# Supplementary material for: Structural basis for ALK2/BMPR2 receptor complex signaling through kinase domain oligomerization
Source: Nat Commun. 2021 Aug 16;12:4950. doi: 10.1038/s41467-021-25248-5 (PMC8368100; doi:10.1038/s41467-021-25248-5)
Supplement: Supplementary file 1 — Supplementary Information [file 41467_2021_25248_MOESM1_ESM.pdf]

Structural basis for ALK2/BMPR2 receptor complex signaling through kinase domain oligomerization

Supplementary Information

Christopher Agnew[1†], Pelin Ayaz[3†], Risa Kashima[1], Hanna S. Loving[4], Prajakta Ghatpande[1], Jennifer E. Kung[1,5], Eric S. Underbakke[4*], Yibing Shan[3*], David E. Shaw[3,6*], Akiko Hata[1], Natalia Jura[1,2*]

[1]Cardiovascular Research Institute, University of California – San Francisco, San Francisco, CA 94158, USA

[2]Department of Cellular and Molecular Pharmacology, University of California – San Francisco, San Francisco, CA 94158, USA

[3]D. E. Shaw Research, New York, NY 10036, USA

[4]Roy J. Carver Department of Biochemistry, Biophysics, and Molecular Biology, Iowa State University, Ames, IA 50011, USA

[5]Current address: Department of Structural Biology, Genentech, Inc., South San Francisco, United States.

[6]Department of Biochemistry and Molecular Biophysics, Columbia University, New York, NY 10032, USA

† Equal contributors

* Correspondence should be addressed to
esu@iastate.edu
Yibing.Shan@DEShawResearch.com,
David.Shaw@DEShawResearch.com,
Natalia.Jura@ucsf.edu (Lead contact)

Supplementary Fig. 1 Agnew, Ayaz et al

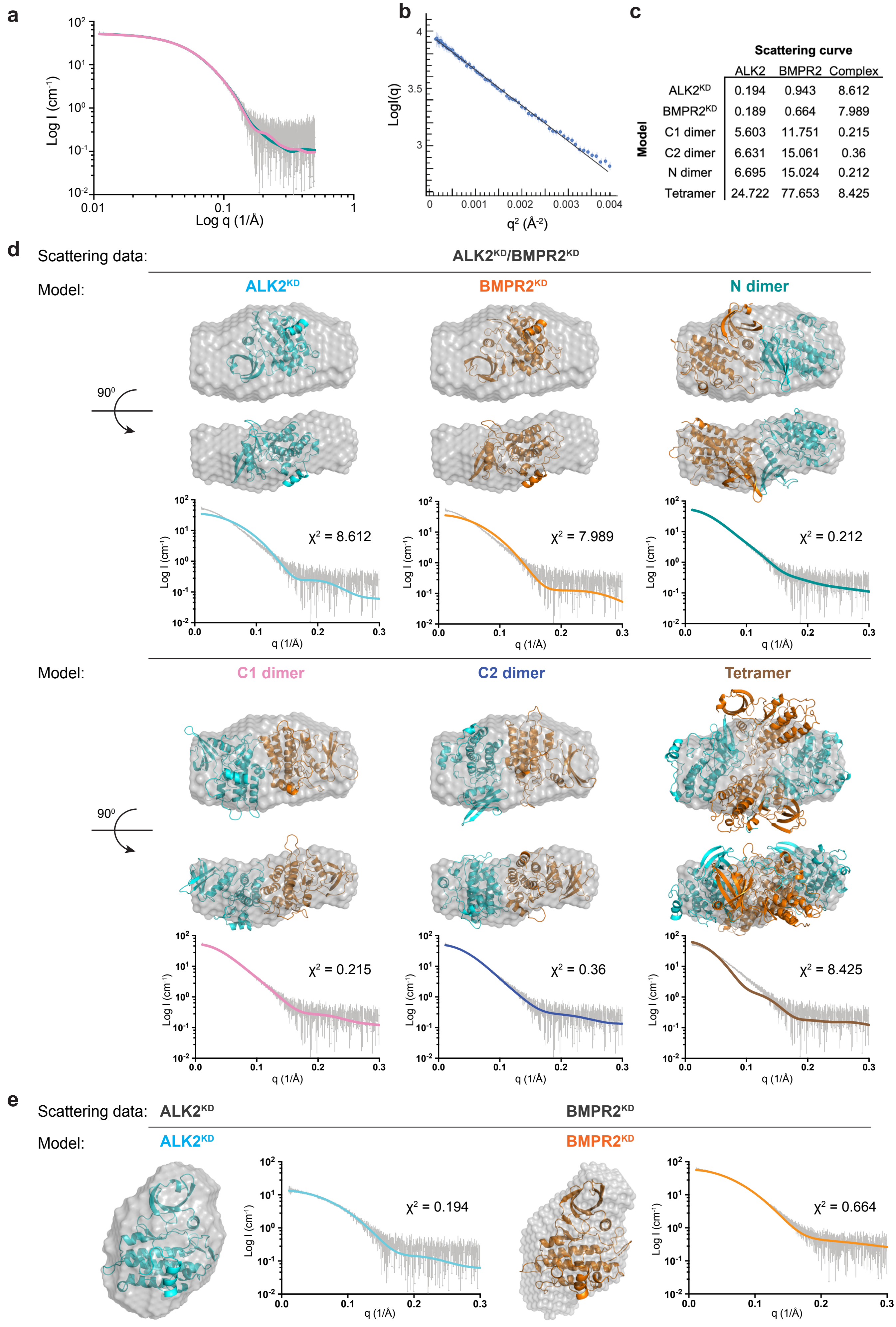

Model	Scattering curve: ALK2	BMPR2	Complex
ALK2KD	0.194	0.943	8.612
BMPR2KD	0.189	0.664	7.989
C1 dimer	5.603	11.751	0.215
C2 dimer	6.631	15.061	0.36
N dimer	6.695	15.024	0.212
Tetramer	24.722	77.653	8.425

Supplementary Figure Legends

Supplementary Fig. 1. SAXS scattering data and modeling.

a Double log plot of the ALK2^{KD}/BMPR2^{KD} complex scattering data highlights the low-angle data. The *CRYSOL* fits calculated for the N dimer model (teal line) and the C1 dimer (pink line) are overlayed.

b Guiner plot of the SAXS low-angle data for the ALK2^{KD}/BMPR2^{KD} complex. The mean of the scattering intensity is plotted +/- the standard deviation.

c Table of χ^2 values reporting on the fit of the crystallographic structures of ALK2^{KD} (PDB 3MTF), BMPR2^{KD} (PDB 3G2F) or the molecular dynamics oligomeric models with the scattering data.

d Scattering data for the ALK2^{KD}/BMPR2^{KD} complex are shown in the lower panel with the *CRYSOL* calculated fit for each model and the χ^2 value. The upper panel corresponds to two orientations rotated ~90° relative to each other showing the superposition of the models to the averaged *ab initio* bead model.

e The scattering data for ALK2^{KD} and BMPR2^{KD} are shown together with the modelled fit and χ^2 value. The *ab initio* bead model for each plot is shown containing the superimposed crystallographic structure.

Supplementary Fig. 2
Agnew, Ayaz et al

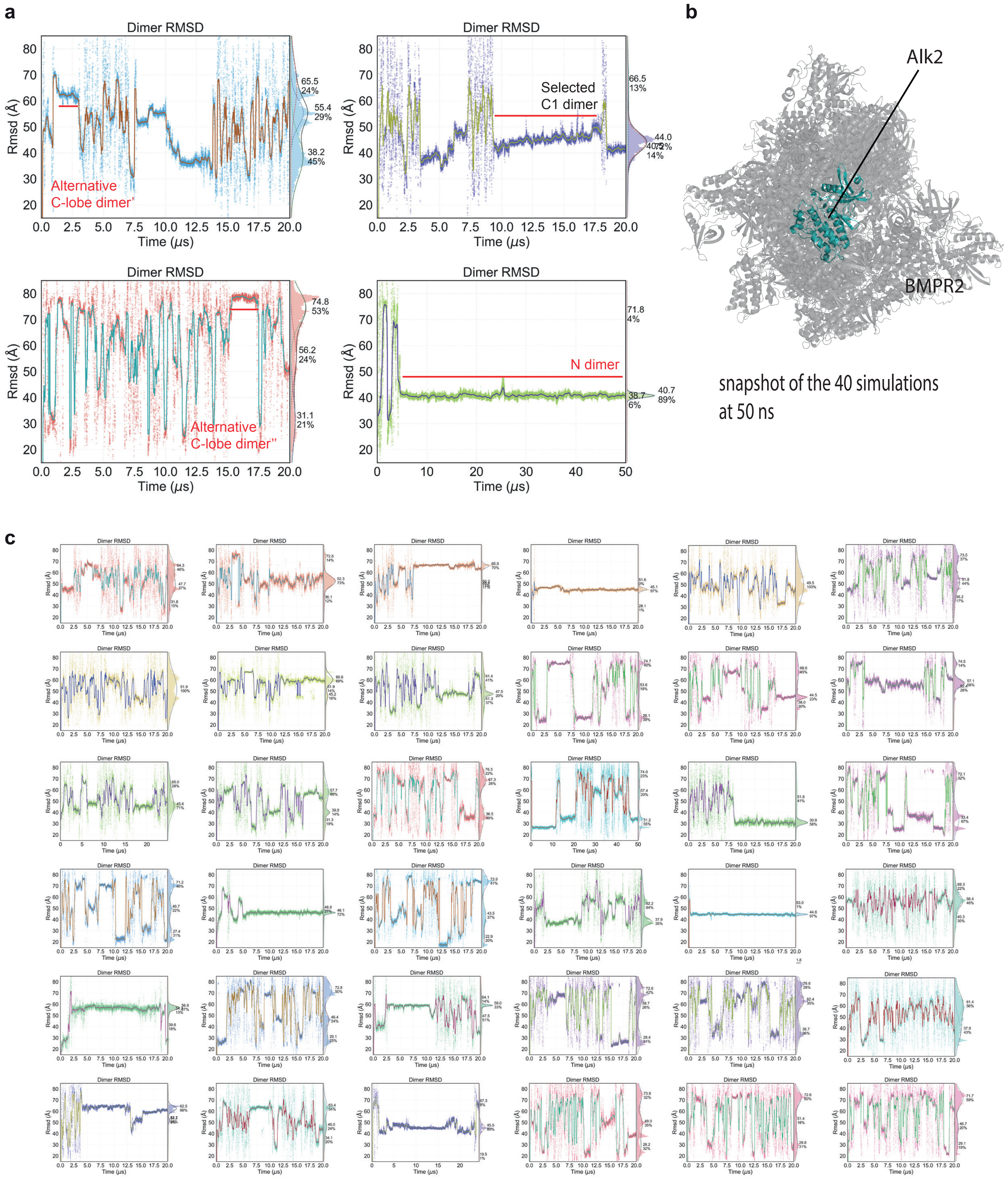

Supplementary Fig. 2. Conformational stability of different C-lobe ALK2/BMPR2 dimers in MD simulations.

a RMSDs of the binding simulations that generated the C1 and N dimers (right-hand panels). The RMSDs were calculated with respect to the initial pose of the simulations; they were calculated for the Cα atoms of ALK2 residues 207–320, 331-359, and 378–499, and BMPR2 residues 198–316, 327–355, and 379–509 (flexible loops were excluded) after superposing the frames using the same ALK2 atom selection. Additional RMSD plots (left-hand panels) correspond to simulations where alternative short-lived C-lobe dimers were observed (labeled in the panels as Alternative C-lobe dimer' and Alternative C-lobe dimer'').

b Frames extracted from the 50 ns time point of each of the 40 binding simulations superposed on ALK2. BMPR2 is distributed almost spherically around ALK2, but it is rendered as partially transparent so that ALK2 is visible.

c RMSDs of the remaining binding simulations (i.e., the 36 not presented in panel **a**) are shown, calculated the same way as in panel **a**. The sampling of a multitude of random unbound poses is evident from the large RMSD fluctuations at the beginning of the simulations.

Supplementary Fig. 3
Agnew, Ayaz et al

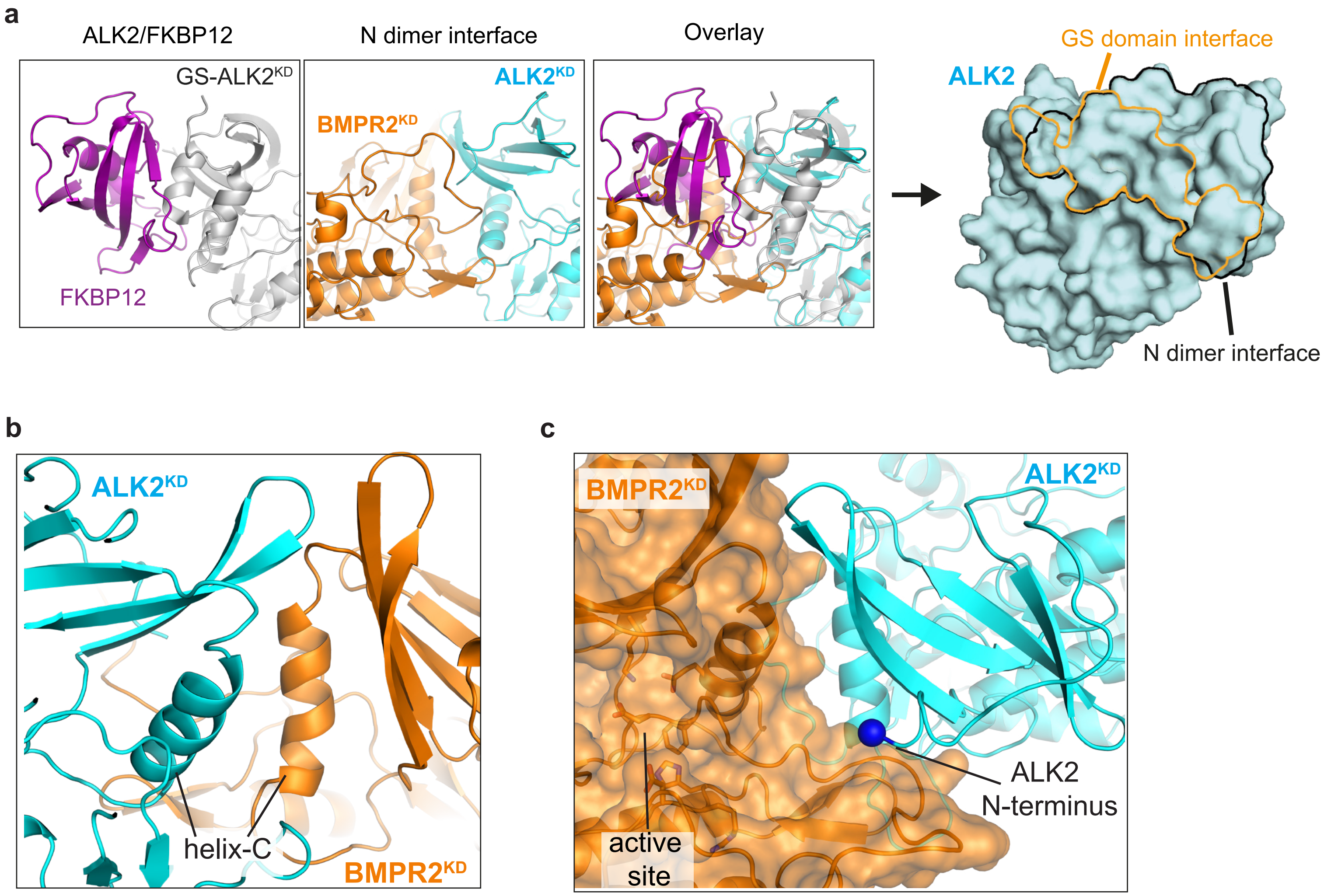

Supplementary Fig. 3. Dimerization of ALK2 and BMPR2 via the N dimer interface competes with GS domain binding to the ALK2 kinase N-lobe.

a Panels on the left show side by side comparison of the inactive ALK2 kinase domain structure stabilized by interaction with the GS domain and binding of the FKBP12 inhibitory protein (PDB: 6I1S) with the ALK2/BMPR2 kinase N dimer interface obtained via our molecular dynamics simulations. Both structures are aligned on the N-lobe of the ALK2 kinase, and overlaid on top of each other in the third panel from the left. In the right-most panel, the BMPR2 kinase binding interface was traced with a black line on the structure of the ALK2 kinase domain based on the structural model of the ALK2/BMPR2 N dimer. The GS domain binding interface on ALK2 was traced with an orange line based on the crystal structure of an inactive ALK2 in the presence of the GS domain and FKBP12 (PDB: 6I1S).

b Zoomed in view of the ALK2/BMPR2 N dimer interface obtained via MD simulations depicting engagement of the helices C of both kinases at the interface. The simulations were conducted in the absence of the GS domain.

c Zoomed in view of the ALK2/BMPR2 N dimer interface obtained via MD simulations indicating the location of the N-terminus of ALK2 in proximity to the BMPR2 active site. The simulations were conducted in the absence of the GS domain.

Supplementary Fig. 4
Agnew, Ayaz et al

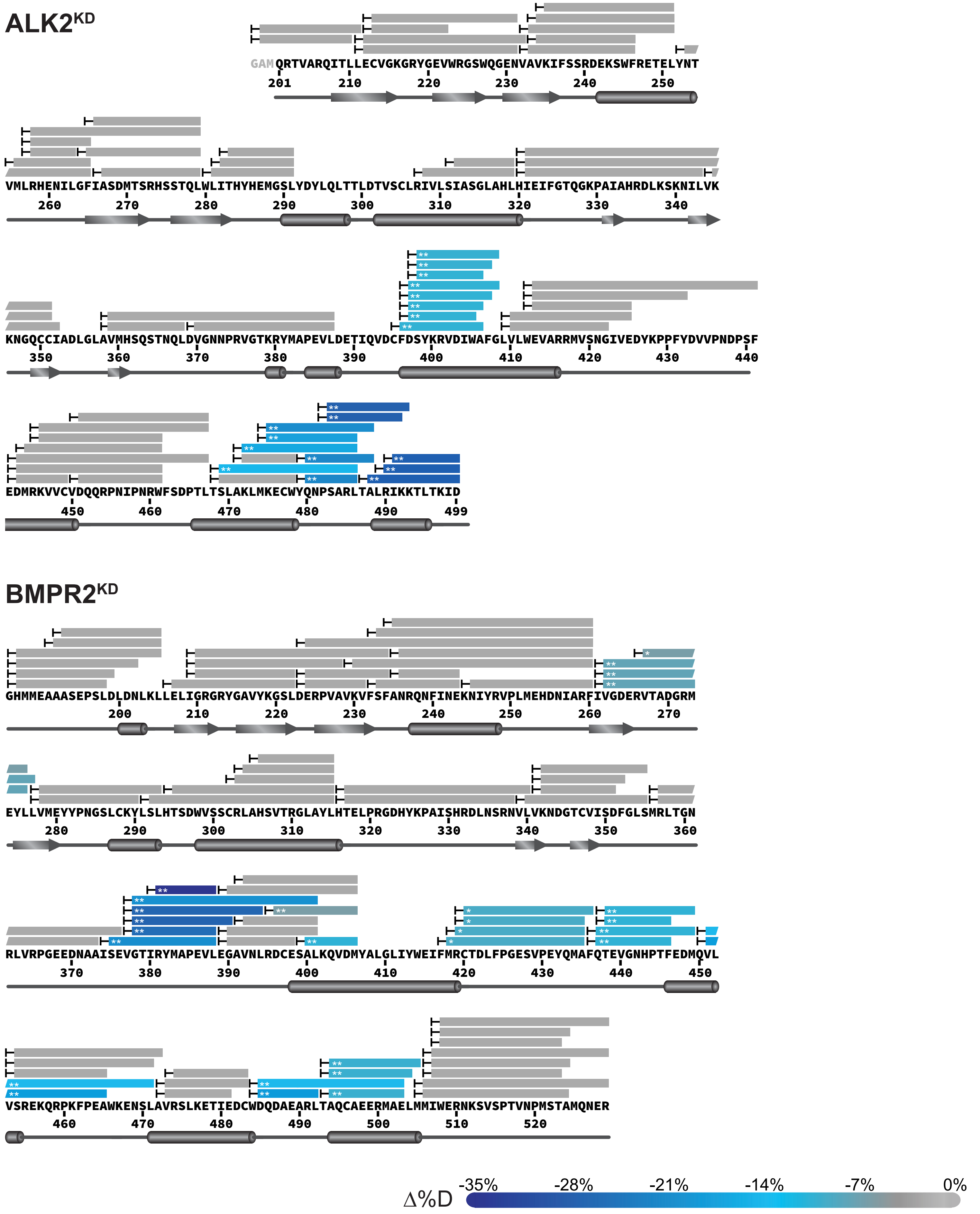

Supplementary Fig. 4. HDX-MS analysis comparing isolated BMPR2^{KD} or ALK2^{KD} vs. the BMPR2^{KD}/ALK2^{KD} complex.

HDX-MS results comparing isolated BMPR2^{KD} or ALK2^{KD} vs. the BMPR2^{KD}/ALK2^{KD} complex are color-coded and mapped above primary sequence with secondary structure annotated below. Coverage is represented as individual bars for each unique peptic peptide. Crossbar lines denote the N-terminal residue of each peptide which exchanges too quickly to measure. Exchange rate perturbations are reported as the average difference in %D incorporation (Δ%D) at time points approximating the midpoint of exchange. Peptides exhibiting significant (** $p<0.005$, * $p<0.01$) exchange rate perturbations between isolated kinases vs. the complex are color coded according to the scale bar (bottom). Significance was assessed with a two-tailed unpaired Student’s t test with n=3 technical replicates representing three independent exchange reactions. Regions exhibiting no significant differences in exchange are colored in grey.

Supplementary Fig. 5
Agnew, Ayaz et al

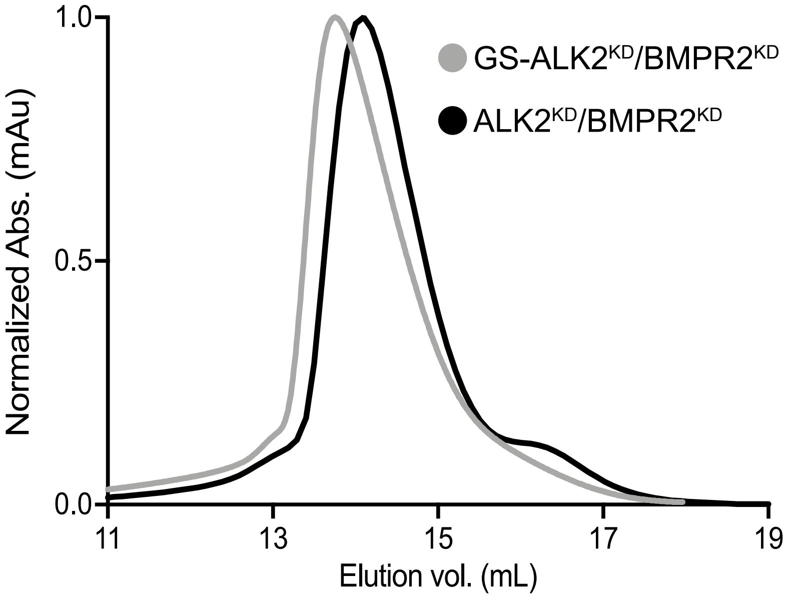

Supplementary Fig. 5. ALK2/BMPR2 kinase dimer forms in solution in the presence of the GS domain.

Size exclusion chromatograms of ALK2^{KD}/BMPR2^{KD} complex (black) and the GS-ALK2^{KD}/BMPR2^{KD} complex (grey) resolved on a Superdex 200 Increase 10/300 GL column.

Supplementary Fig. 6
Agnew, Ayaz et al

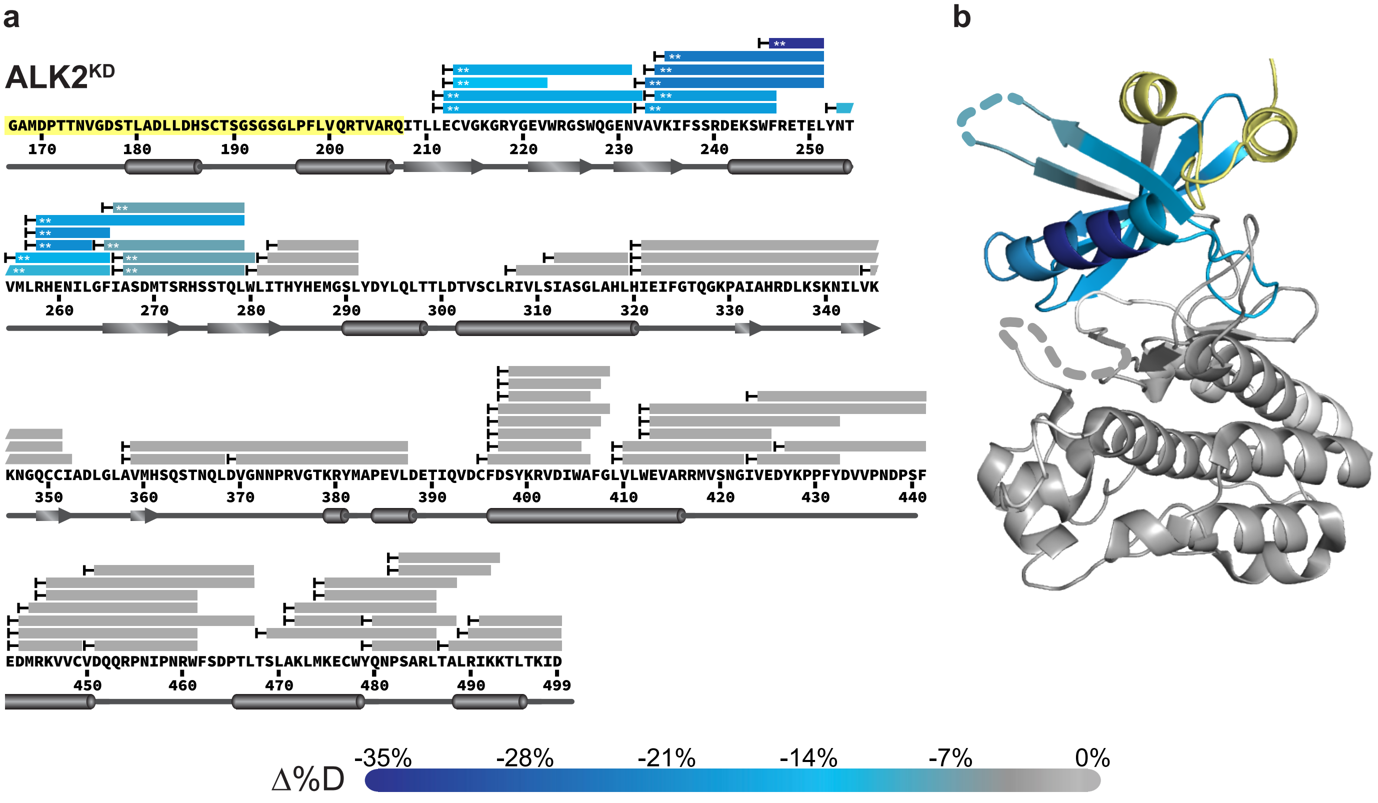

Supplementary Fig. 6. HDX-MS analysis comparing $ALK2^{KD}$ vs $GS\text{-}ALK2^{KD}$.

a HDX-MS results comparing $ALK2^{KD}$ vs. $GS\text{-}ALK2^{KD}$ are color-coded and mapped above primary sequence with secondary structure annotated below. The GS domain is highlighted in yellow. Coverage is represented as individual bars for each unique peptic peptide. Crossbar lines denote the N-terminal residue of each peptide which exchanges too quickly to measure. Exchange rate perturbations are reported as the average difference in %D incorporation (Δ%D) at time points approximating the midpoint of exchange. Peptides exhibiting significant (** $p<0.005$, * $p<0.01$) exchange rate perturbations comparing $ALK2^{KD}$ and $GS\text{-}ALK2^{KD}$ samples are color coded according to the scale bar (bottom). Significance was assessed with a two-tailed unpaired Student's t test with n=3 technical replicates representing three independent exchange timecourses. Regions exhibiting no significant differences in exchange are colored in grey.

b Perturbations were mapped to a model of GS-ALK2 (PDB 3H9R) using color-coding as in **a**. Regions lacking peptide coverage are noted in white.

Supplementary Fig. 7
Agnew, Ayaz et al

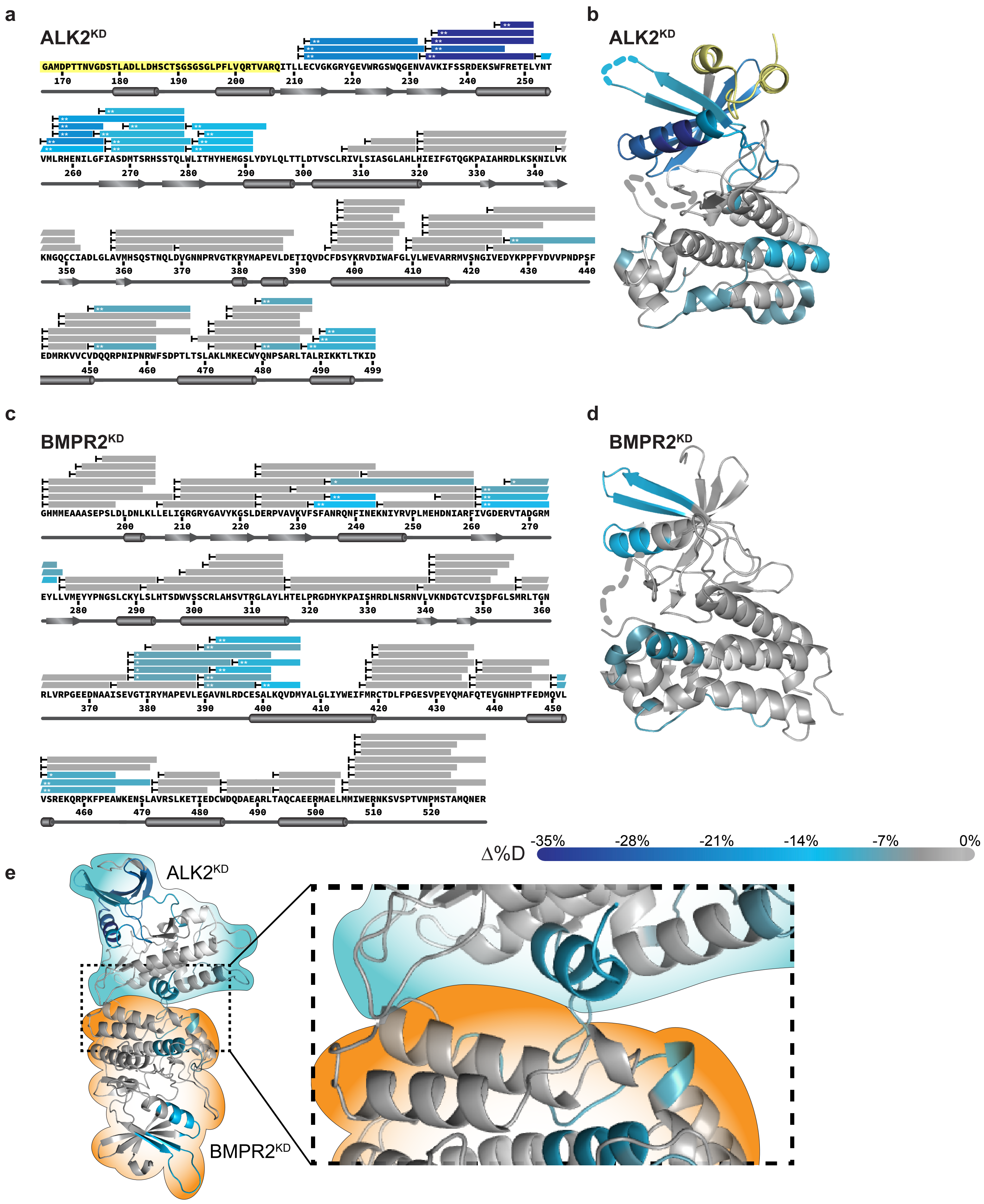

Supplementary Fig. 7. HDX-MS analysis comparing the ALK2^{KD}/BMPR2^{KD} complex vs the BMPR2^{KD}/GS-ALK2^{KD} show GS domain-dependent HDX exchange rate perturbations.

a-d HDX-MS results comparing the ALK2/BMPR2 complex vs GS-ALK2/BMPR2 complex are color-coded and mapped above the primary sequences of ALK2 (**a**) and BMPR2 (**c**). The GS domain is highlighted in yellow. Coverage is represented as individual bars for each unique peptic peptide. Crossbar lines denote the N-terminal residue of each peptide which exchanges too quickly to measure. Exchange rate perturbations are reported as the average difference in %D incorporation (Δ%D) at time points approximating the midpoint of exchange. Peptides exhibiting significant (** $p<0.005$, * $p<0.01$) GS domain-dependent exchange rate perturbations are color coded according to the scale bar (top). Significance was assessed with a two-tailed unpaired Student's t test with n=3 technical replicates representing three independent exchange reactions. Regions exhibiting no significant differences in exchange are colored in grey. GS-dependent perturbations were mapped to structural models of (**b**) ALK2 (PDB: 3H9R) and (**d**) BMPR2 (PDB: 3G2F) using color-coding as in **a** and **c**, respectively. Regions lacking peptide coverage are noted in white.

e Exchange rate perturbations mapped to C1 dimer model. BMPR2^{KD} is outlined in orange, ALK2^{KD} in cyan.

Supplementary Fig. 8
Agnew, Ayaz et al

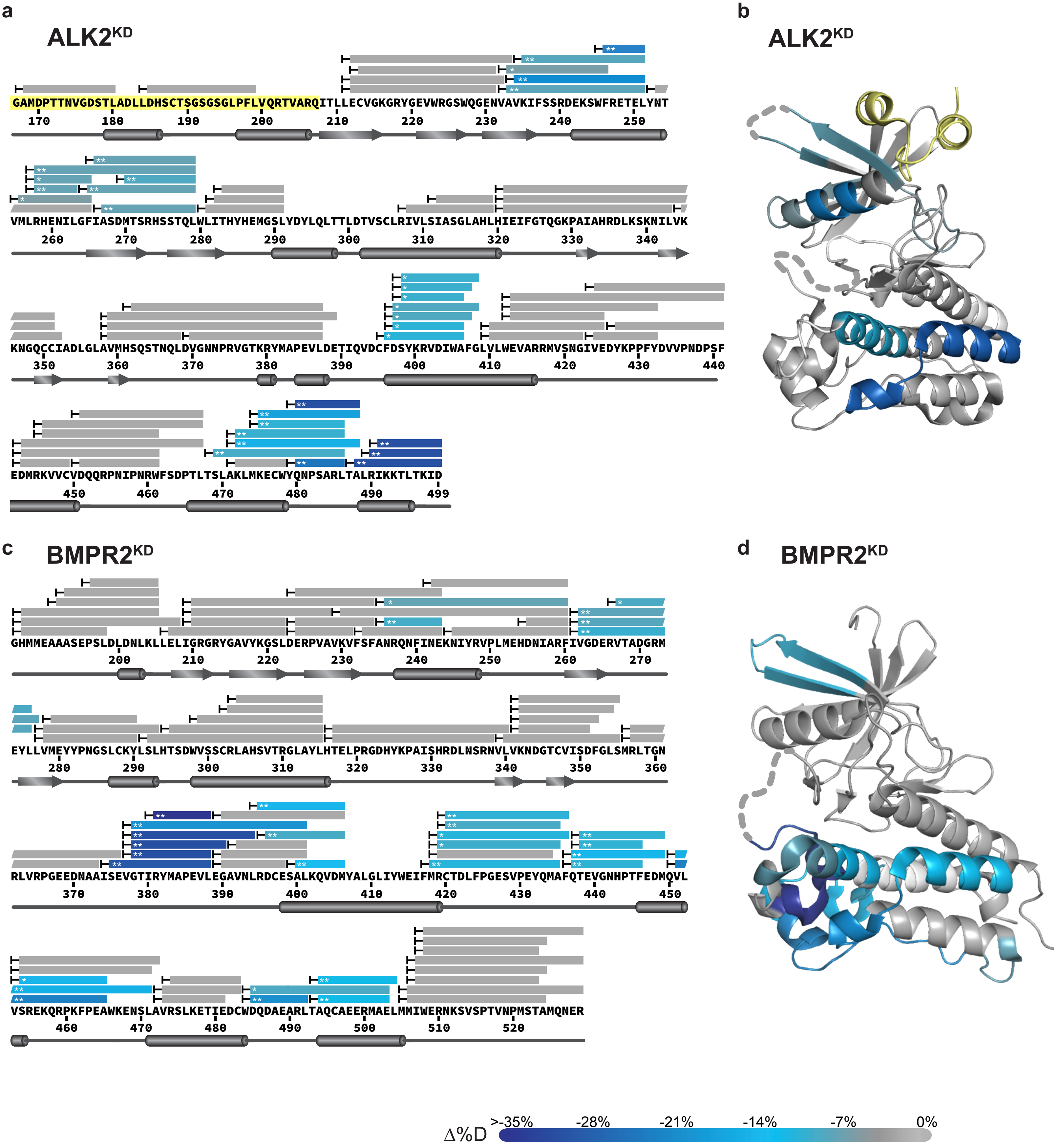

Supplementary Fig. 8. HDX-MS analysis comparing isolated BMPR2^{KD} or GS-ALK2^{KD} vs the BMPR2^{KD}/GS-ALK2^{KD} complex show GS domain-dependent HDX exchange rate perturbations.

a-d HDX-MS results comparing the isolated GS-ALK2 or BMPR2 vs GS-ALK2/BMPR2 complex are color-coded and mapped above the primary sequences of ALK2 (**a**) and BMPR2 (**c**). The GS domain is highlighted in yellow. Coverage is represented as individual bars for each unique peptic peptide. Crossbar lines denote the N-terminal residue of each peptide which exchanges too quickly to measure. Exchange rate perturbations are reported as the average difference in %D incorporation (Δ%D) at time points approximating the midpoint of exchange. Peptides exhibiting significant (** $p<0.005$, * $p<0.01$) exchange rate perturbations are color coded according to the scale bar (top). Significance was assessed with a two-tailed unpaired Student's t test with n=3 technical replicates representing three independent exchange time courses. Regions exhibiting no significant differences in exchange are colored in grey. GS-dependent perturbations were mapped to structural models of (**b**) GS-ALK2 (PDB: 3H9R) and (**d**) BMPR2 (PDB: 3G2F) using color-coding as in **a** and **c**, respectively. Regions lacking peptide coverage are noted in white.

Supplementary Fig. 9
Agnew, Ayaz et al

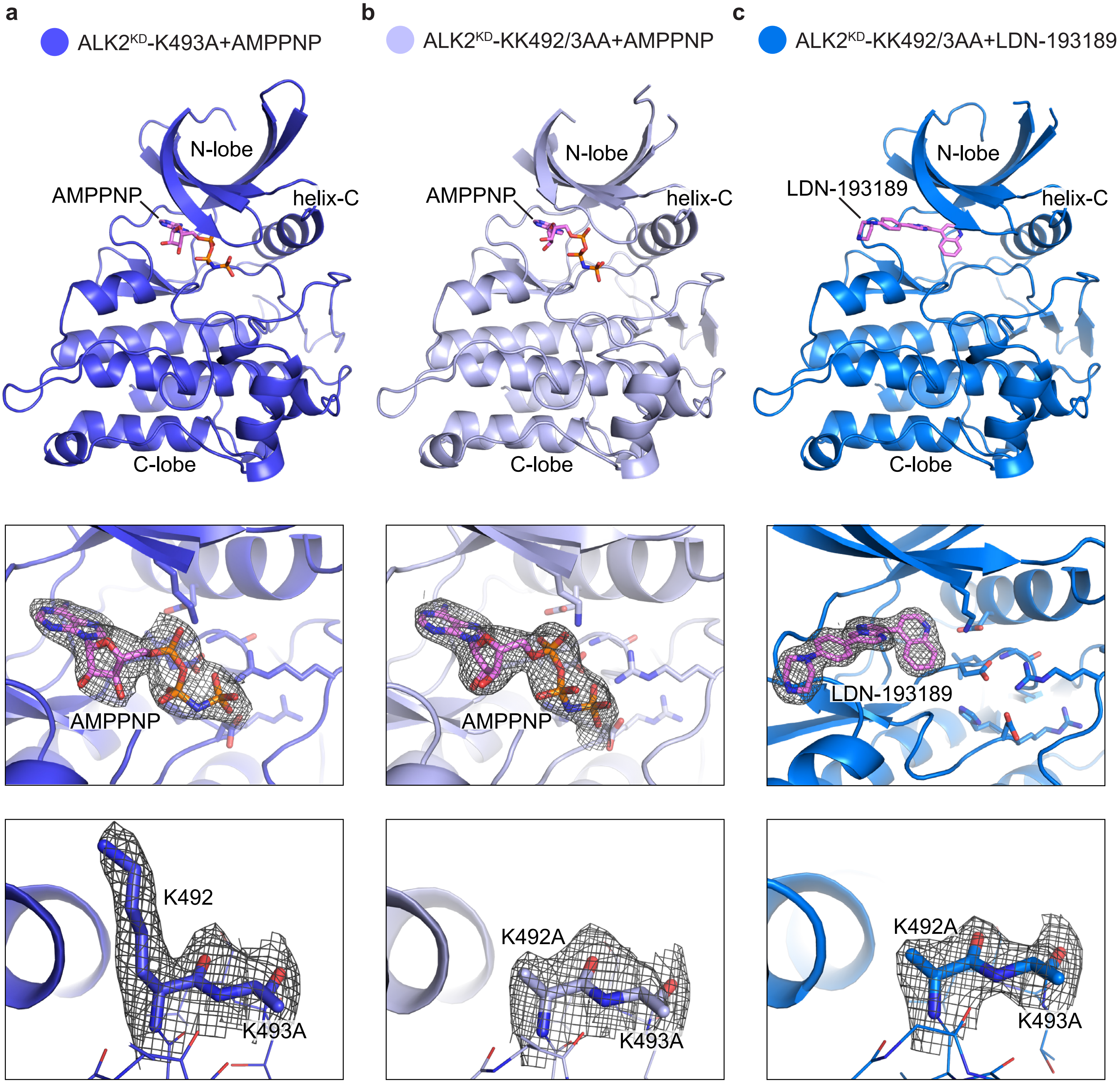

Supplementary Fig. 9. Crystal structures of the ALK2 kinase domain carrying mutations in the C-lobe dimer interface in complex with AMP-PNP and the LDN-193189 inhibitor.

a Top panel shows cartoon representation of the $ALK2^{KD}$ K493A mutant structure in complex with AMP-PNP. Middle panel depicts detailed view of the nucleotide-binding site, showing the electron density (at 1.5 σ above the mean value) around the AMP-PNP molecule. The difference electron density maps shown were calculated using a model of the protein at a stage before the inclusion of AMP-PNP in the refinement. Bottom panel shows zoomed-in view of the C-lobe interface in ALK2 highlighting mutagenesis of K493 to an alanine demonstrated as lack of the electron density (at 1.2 σ above the mean value) around the lysine side chain.

b Top panel shows cartoon representation of the $ALK2^{KD}$ K492A/K493A mutant structure in complex with AMP-PNP. Middle panel depicts detailed view of the nucleotide-binding site, showing the electron density (at 1.5 σ above the mean value) around the AMP-PNP molecule. The difference electron density maps shown were calculated using a model of the protein at a stage before the inclusion of AMP-PNP in the refinement. Bottom panel shows zoomed-in view of the C-lobe interface in ALK2 highlighting mutagenesis of K492 and K493 to alanines demonstrated as lack of the electron density (at 1.2 σ above the mean value) around the lysine side chains.

c To panels shows cartoon representation of the $ALK2^{KD}$ K492A/K493A mutant structure in complex with kinase inhibitor: LDN-193189. Middle panel depicts detailed view of the nucleotide-binding site, showing the electron density (at 1.5 σ above the mean value) around the LDN-193189 inhibitor molecule. The difference electron density maps shown were calculated using a model of the protein at a stage before the inclusion of LDN-193189 in the refinement. Bottom panel shows zoomed-in view of the C-lobe interface in ALK2 highlighting mutagenesis of K492 and K493 to alanines demonstrated as lack of the electron density (at 1.2 σ above the mean value) around the lysine side chains.

Supplementary Fig. 10
Agnew, Ayaz et al

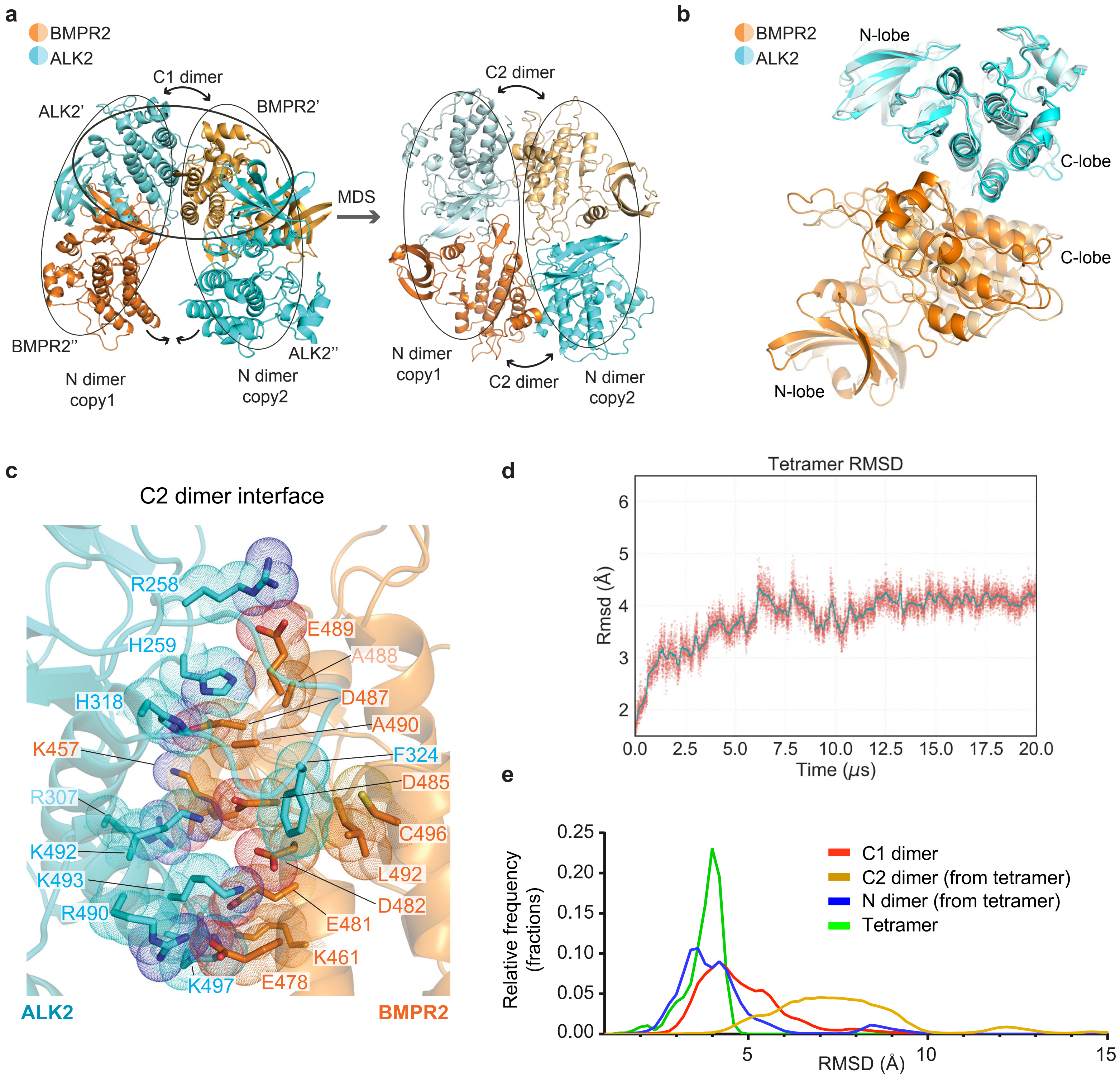

Supplementary Fig. 10. The structural model of the ALK2/BMPR2 kinase tetramer.

a The ALK2/BMPR2 kinase tetramer was assembled using the ALK2'/BMPR2' C1 dimer as a template. The N-dimer interface was used to allow ALK2' to interact with a second BMPR2" molecule and to allow BMPR2' to interact with a second ALK2".

b Two copies of the C:C dimer extracted from the final tetramer model are superposed to show that even when it was not enforced, the two C:C interfaces are equivalent in the tetramer.

c A zoomed-in view of the C:C dimer interface in the tetramer showing positions of the charged residues within the C-lobes of the BMPR2 and ALK2 kinases involved in electrostatic interactions.

d RMSD of the final tetramer model in a 20-μs MD simulation. The RMSD was calculated for the Cα atoms after superposing the frames to the initial pose of the simulation using the Cα atoms. Details of the simulation are summarized in the Supplementary Table 4.

e Normalized distribution of the RMSD values over the trajectories of the individual systems plotted for the MD simulations of the C1 dimer and compared to: (i) the final tetramer and (ii) the N dimer and C2 dimer extracted from the final tetramer model and subjected to MD simulations. The RMSD data are reported in Supplementary Fig. 13. Details of the simulations are summarized in Supplementary Table 4.

Supplementary Fig. 11
Agnew, Ayaz et al

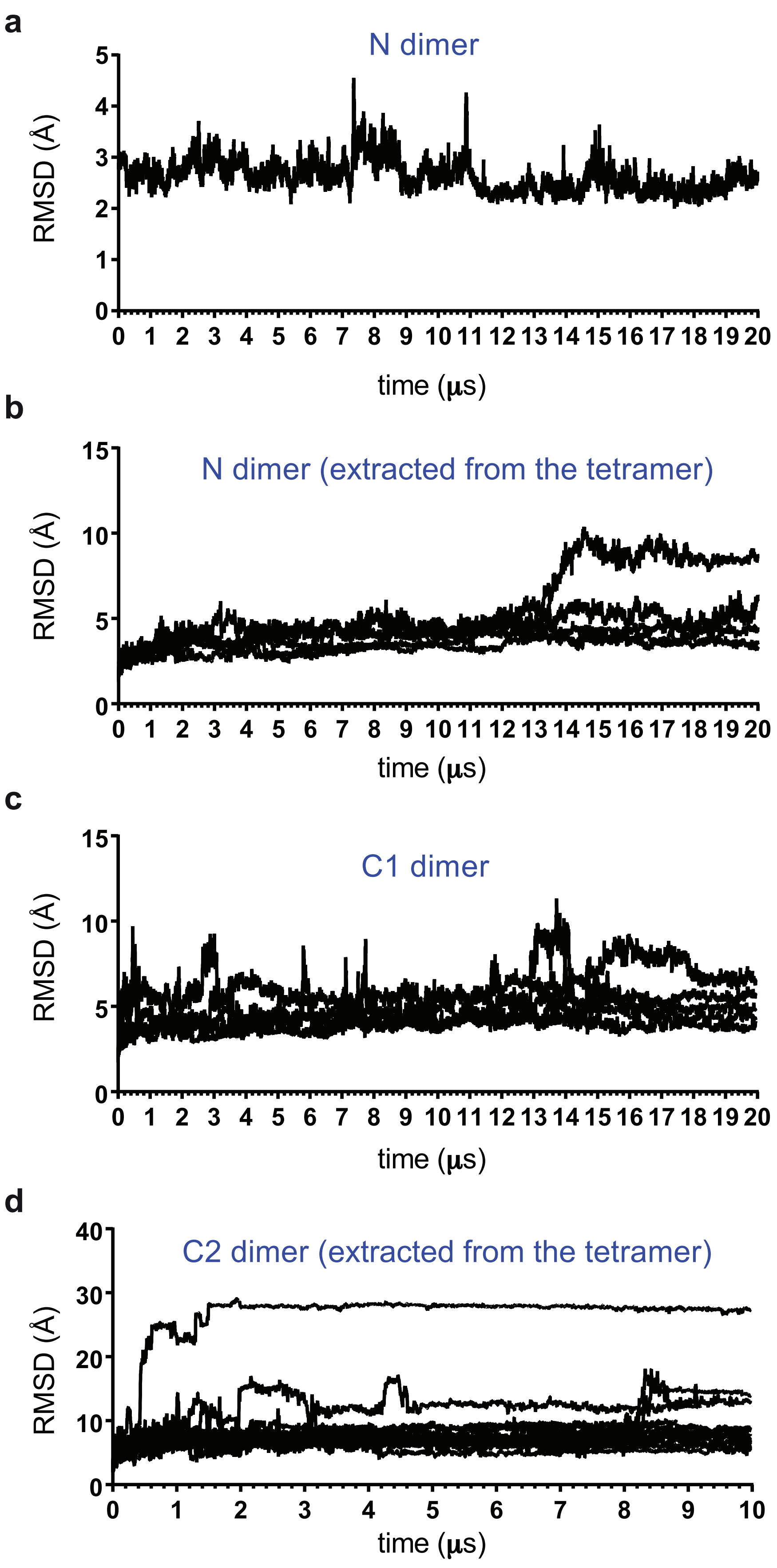

Supplementary Fig. 11. Individual RMSD plots for assessing the conformational stability of the dimer models. RMSD with respect to the initial poses was calculated using the Cα atoms after alignment using the Cα atoms. Details of the simulation are summarized in the Supplementary Table 4. **a** Simulation initiated from the N dimer generated by a spontaneous binding simulation; **b** Simulations initiated from the N dimer extracted from a simulation of the tetramer model; **c** Simulation initiated from the C1 dimer generated by a spontaneous binding simulation; **d** Simulations initiated from a C2 dimer extracted from a simulation of the tetramer model.

Supplementary Fig. 12
Agnew, Ayaz et al

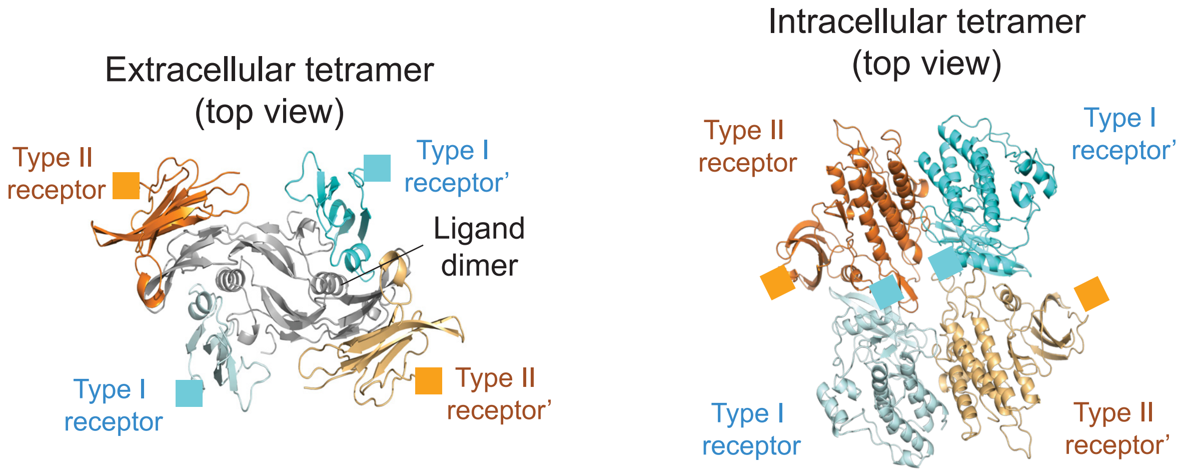

Supplementary Fig. 12. Comparison of the ALK2/BMPR2 kinase tetramer architecture with the ligand bound type I/type II extracellular domain hetero-tetramer.

Left panel - crystal structure of a BMP receptor extracellular domain tetramer composed of two type I BMPR1 receptors - in cyan, two type II ACVR2B receptors in cyan and a dimeric BMP2 ligand in grey (PDB: 2H64). The C-termini of the proteins are marked by respectively colored boxes. Right panel – our MD model of the ALK2/BMPR2 kinase tetramer. The kinase N-termini are marked as colored boxes.

Supplementary Fig. 13
Agnew, Ayaz et al

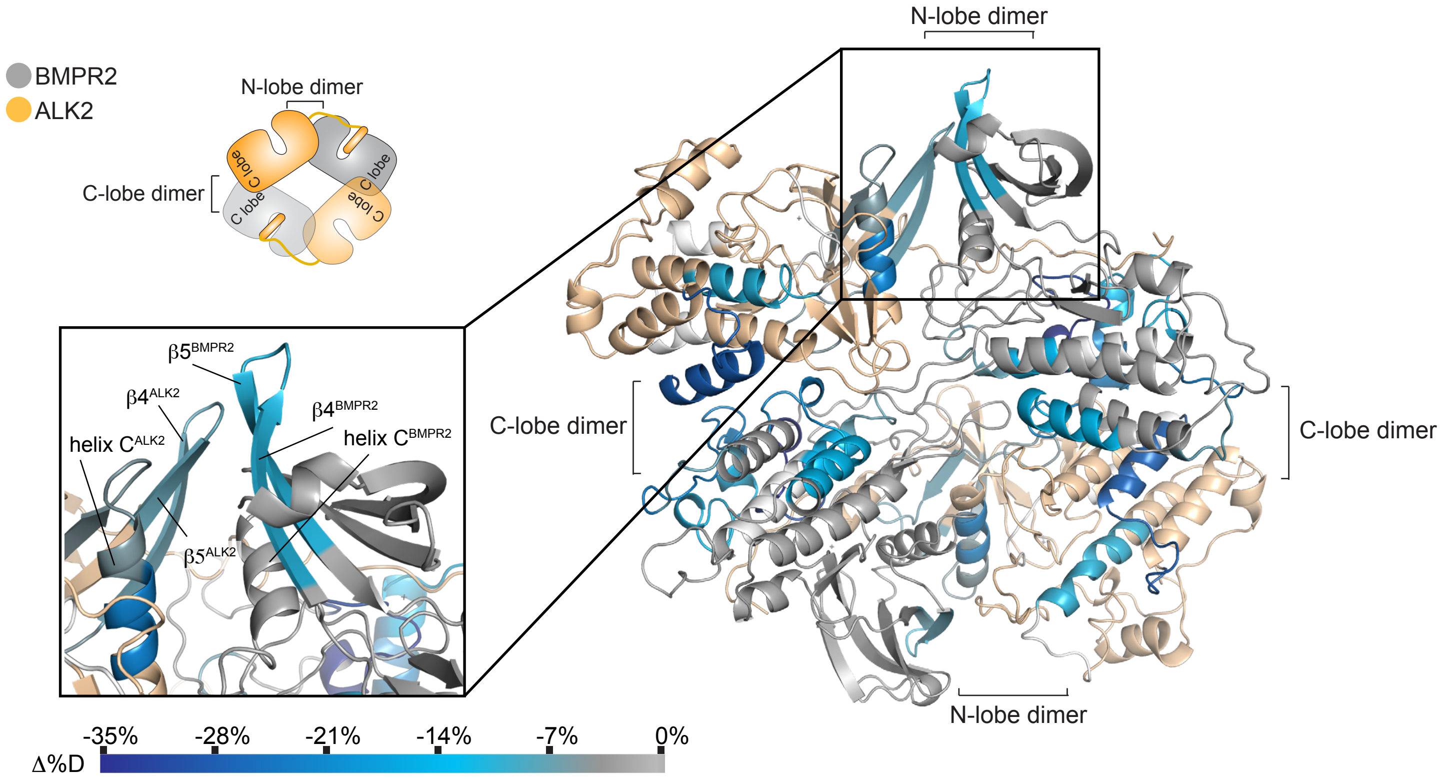

Supplementary Fig. 13. Exchange rate perturbations of GS-Alk2 and BMPR2 kinases induced by complex formation mapped to the tetramer architecture.

HDX-MS results comparing isolated GS-ALK2 and BMPR2 kinase constructs vs. GS-ALK2/BMPR2 complex are color-coded and mapped to the tetramer architecture model. Exchange rate perturbations are reported as the average difference in %D incorporation (Δ%D) at time points approximating the midpoint of exchange. Peptides exhibiting significant (** $p<0.005$, * $p<0.01$) exchange rate perturbations are color coded according to the scale bar (bottom). Significance was assessed with a two-tailed unpaired Student's t test. Regions exhibiting no significant differences in exchange are colored in grey for BMPR2 and pale orange for ALK2. Regions lacking peptide coverage are noted in white.

Supplementary Figure 14
Agnew, Ayaz et al

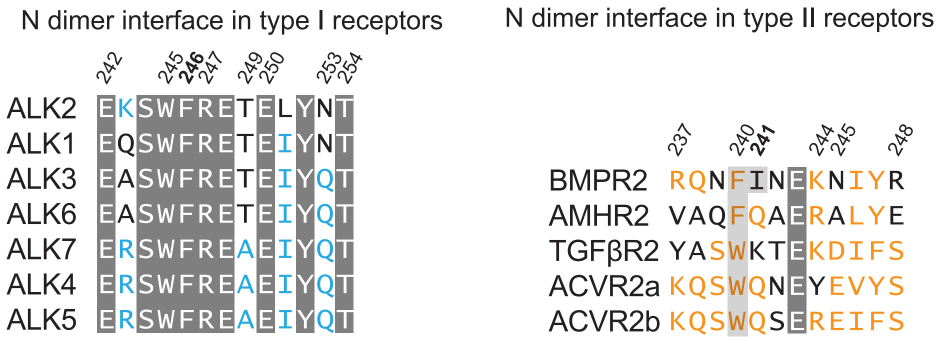

Supplementary Fig. 14. Comparison of the ALK2/BMPR2 kinase tetramer architecture with the ligand bound type I/type II extracellular domain hetero-tetramer.

Sequence alignment of the regions within the human TGF-β superfamily of receptors that correspond to the N dimer interface. Identical residues are highlighted by a grey background and highly conserved residues are colored in blue in type I receptors, and in orange in type II receptors.

Supplementary Figure 15
Agnew, Ayaz et al

a

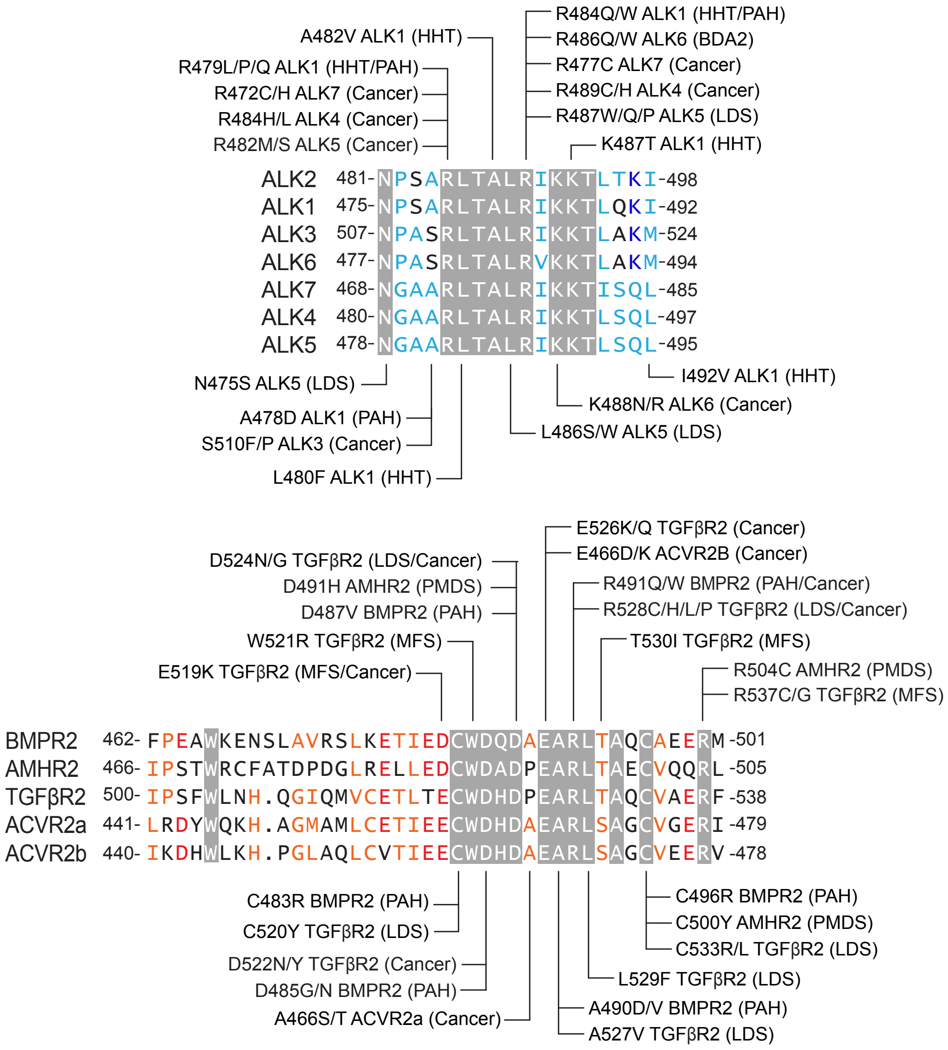

b

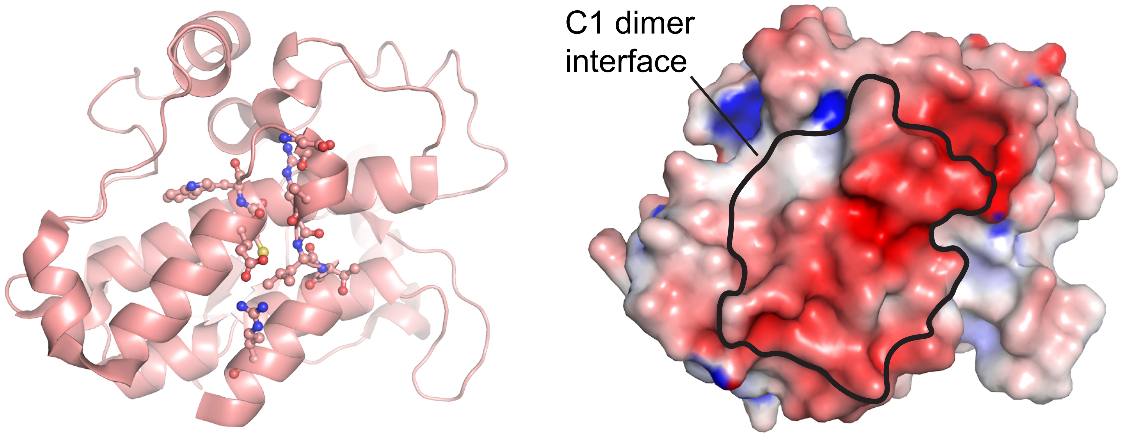

Supplementary Fig. 15. Mapping of TGF-β superfamily mutations to the C dimer interface.

a Sequence alignment of the region within the kinase domains of human TGF-β superfamily of receptors that correspond to the C dimer interface. Identical residues are highlighted by a grey background. Similar residues are colored as light blue in type I and light orange in type II receptors. Disease mutations are marked for individual receptors on the alignment. The relevant disorder names are indicated in brackets and represent following abbreviations: Loeys-Dietz syndrome (LDS), Pulmonary arterial hypertension (PAH), Hereditary hemorrhagic telangiectasia (HHT), Brachydactyly A2 (BDA2), Marfan syndrome (MFS) and persistent Müllerian duct syndrome (PMDS). Cancer mutations were cataloged by using cBioPortal and COSMIC databases when they were identified in two or more independent samples.

b Mutations in the TbRII kinase domain that cause Marfan syndrome map to the C dimer interface. In the left panel, mutations causing Marfan syndrome that localize to the C-lobe of the TbRII kinase are shown as sticks on the structure of the TbRII kinase domain (PDB: 5QIN). The structure is in the same orientation in the right panel, with depicted electrostatic surface potential (APBS), to demonstrate overlap between the C1 dimer interface and the localization of mutations.

Supplementary Table 1
Agnew, Ayaz et al

	BSA (Å ^{2})	#H-bonds	#salt bridges	
Swimming Simulations				
	565.8	5	0	
	404.3	10	3	
	709.8	5	1	
	1266.6	15	8	
	1016.0	7	2	
N dimer	1262.2	8	5	N-N interaction
	940.8	6	0	
	719.5	6	6	
	266.6	1	2	
	857.8	6	0	
	511.3	4	2	
	861.9	2	0	
	568.1	3	4	
	991.5	10	3	
	584.1	2	1	
C1 dimer	861.3	7	7	C-C interaction
	1123.8	13	1	
	928.8	8	4	
C1 dimer final	1092	14	22	C-C interaction
Tetramer				
ALK2a-BMPR2a	2396	1	27	N-N interaction
ALK2b-BMPR2b	2352	5	15	N-N interaction
ALK2a-BMPR2b	809	1	15	C-C interaction
ALK2b-BMPR2b	677	0	19	C-C interaction
BMPR2a-BMPR2b	573	0	2	

Supplementary Table 1. The detailed properties of identified protein-protein interfaces are summarized. Interface properties, burried surface area (BSA), number of hydrogen bonds and number of salt bridges were calculated using the PISA software integrated in the CCP4 suite (Winn et al, 2011).

Supplementary Table 2
Agnew, Ayaz et al

	BMPR2-D485G ADP	ALK2-K493A AMPPNP	ALK2-KK492/3AA AMPPNP	ALK2-KK492/3AA LDN-193189
PDB code	6UNP	6UNQ	6UNR	6UNS
Resolution range (Å)	45.33 – 2.3 (2.382 – 2.3)	49.07 – 2.4 (2.486 – 2.4)	46.14 – 2.2 (2.279 – 2.2)	45.97 – 2.3 (2.382 – 2.3)
Space group	$P\ 3_2\ 3\ 1$	$C\ 2\ 2\ 2_1$	$C\ 2\ 2\ 2_1$	$P\ 2\ 2_1\ 2_1$
Unit cell (Å)	138.5, 138.5, 109.9 90º, 90º, 120º	59.5, 86.9, 138 90º, 90º, 90º	59.1, 87.1, 138.9 90º, 90º, 90º	60, 83.6, 138 90º, 90º, 90º
Total reflections	108706 (10716)	27264 (2673)	37086 (3643)	63217 (6188)
Unique reflections	54353 (5358)	14208 (1406)	18574 (1825)	31609 (3094)
Multiplicity	19.2 (12.4)	3.6 (3.4)	7.2 (7.3)	12.8 (13.5)
Completeness (%)	99.93 (99.91)	98.51 (98.66)	99.24 (98.63)	99.92 (99.90)
Mean I/sigma(I)	16.74 (1.39)	21.84 (5.16)	11.02 (1.95)	8.88 (1.73)
Wilson B-factor (Å^2)	48.71	46.16	42.40	34.35
R-merge	0.02912 (0.5051)	0.01684 (0.115)	0.03018 (0.3567)	0.05145 (0.417)
$CC_{1/2}$	0.999 (0.707)	1 (0.987)	1 (0.942)	1 (0.908)
Reflections used in refinement	54327 (5353)	14147 (1398)	18464 (1804)	31599 (3094)
Reflections used in R-free	2714 (295)	678 (67)	889 (94)	1624 (164)
R-work	0.1885	0.2178	0.2435	0.2123
R-free	0.2112	0.2815	0.3025	0.2614
No. of non-hydrogen atoms	4994	2325	2236	4594
macromolecules	4718	2287	2185	4522
ligands	182	35	39	62
solvent	94	3	12	10
Protein residues	605	289	286	578
RMSD (bonds, Å)	0.008	0.008	0.003	0.008
RMSD (angles, º)	1.11	1.12	0.75	0.97
Ramachandran favored (%)	96.53	94.4	92.86	97.17
Ramachandran outliers (%)	0.0	0.0	0.71	0.35
Clashscore	3.04	8.3	6.02	5.87
Average B-factor (Å^2)	59.92	71.48	74.05	37.84
macromolecules	59.79	71.57	73.78	37.96
ligands	66.99	67.61	92.51	29.42
solvent	52.69	47.33	63.74	37.21

Supplementary Table 2. X-ray crystallographic data refinement and statistics. Statistics for the highest-resolution shells are shown in parentheses. RMSD stands for root mean square deviation from ideal geometry.

Supplementary Table 3
Agnew, Ayaz et al

Primer Name	Primer Sequence
BMPR2-KD_pET28a_For	GGCGAAAACCTGTACTTCCAGGGCCATATGATGGAGGCAGCAGCATCCGAACCC
BMPR2-KD_pET28a_Rev	GTGCTCGAGTGCGGCCGCAAGCTTTTAGCGTTCATTCTGCATAGCAGTAGACATTGGATTG
BMPR2_I241E_For	CCTTTGCAAACCGTCAGAATTTTGAGAACGAAAAGAACATTTACAGAGTGCC
BMPR2_I241E_Rev	GGCACTCTGTAAATGTTCTTTTCGTTCTCAAAATTCTGACGGTTTGCAAAGG
BMPR2_E464R_For	CAGAGACCCAAGTTCCCAAGAGCCTGGAAAGAAAATAGCCTGGCA
BMPR2_E464R_Rev	TGCCAGGCTATTTTCTTTCCAGGCTCTTGGGAACTTGGGTCTCTG
BMPR2_D487R_E489R_For	GTTGGGACCAGAGGGCAAGGGCTCGGCTTACTGCACAGTGTGCTGAGGAAAGG
BMPR2_D487R_E489R_Rev	CCTTTCCTCAGCACACTGTGCAGTAAGCCGAGCCCTTGCCCTCTGGTCCCAAC
BMPR2_E478R_E481R_D482R_For	AGGACAATCAGAAGATGTTGGGACCAGGATGCAGAGGCTCGGCTTACTGCACAGTGTG
BMPR2_E478R_E481R_D482R_Rev	TCTTCTGATTGTCCTCTTGAGTGACCTCACTGCCAGGCTATTTTCTTTCCAGGCTTCTGG
BMPR2_D485G_For	CGAAGACTGTTGGGGCCAGGATGCAGAGG
BMPR2_D485G_Rev	CCTCTGCATCCTGGCCCCAACAGTCTTCG
ALK2-KD_pFastBac_For	AAAACCTGTATTTTCAGGGCGCCATGGGGCAAAGAACAGTGGCTCGCCAG
ALK2-KD_pFastBac_Rev	CTCGACAAGCTTGGTACCGCATGCCTCGAGTTAATCAATTTTGGTCAAAGTCTTTTTGATACGCAG
ALK2_F246R_For	CCGTGATGAGAAGTCATGGAGGAGGGAAACGGAATTGTAC
ALK2_F246R_Rev	GTACAATTCCGTTTCCCTCCTCCATGACTTCTCATCACGG
ALK2_R485E_For	CTGGTATCAAAATCCATCCGCAGAGCTCACAGCACTGCGTATCAAAA
ALK2_R485E_Rev	TTTTGATACGCAGTGCTGTGAGCTCTGCGGATGGATTTTGATACCAG
ALK2_R485E_R490E_For	CATCCGCAGAGCTCACAGCACTGGAGATCAAAAAGACTTTGACCAAAAT
ALK2_R485E_R490E_Rev	ATTTTGGTCAAAGTCTTTTTGATCTCCAGTGCTGTGAGCTCTGCGGATG
ALK2_K492A_For	GCAAGACTCACAGCACTGCGTATCGCAAAGACTTTGACCAAAATTG
ALK2_K492A_Rev	CAATTTTGGTCAAAGTCTTTGCGATACGCAGTGCTGTGAGTCTTGC
ALK2_K493A_For	CACAGCACTGCGTATCAAAGCGACTTTGACCAAAATTG
ALK2_K493A_Rev	CAATTTTGGTCAAAGTCGCTTTGATACGCAGTGCTGTG
ALK2_K492A_K493A_For	CCGCAAGACTCACAGCACTGCGTATCGCAGCGACTTTGACCAAAATTG
ALK2_K492A_K493A_Rev	CAATTTTGGTCAAAGTCGCTGCGATACGCAGTGCTGTGAGTCTTGCGG
ALK2_K497E_pFastBac_For	CAAAAAGACTTTGACCGAGATTGATTAACTCGAGGCATGCGGTACCAAG
ALK2_K497E_pFastBac_Rev	CTTGGTACCGCATGCCTCGAGTTAATCAATCTCGGTCAAAGTCTTTTTG
ALK2_K497E_pcDNA_For	GCGTATCAAAAAGACTTTGACCGAAATTGATAATTCCCTCGAC
ALK2_K497E_pcDNA_Rev	GTCGAGGGAATTATCAATTTCGGTCAAAGTCTTTTTGATACGC

Supplementary Table 4
Agnew, Ayaz et al

**Data-collection parameters**			
Instrument	Beamline 4-2 at SSRL with Dectris Pilatus3 X 1M		
Wavelength (Å)	1.13		
q range ($Å^{-1}$)	0.01 – 0.3.00		
Exposure time	1 sec		
Concentration	100 μM		
Temperature (K)	293		
**Structural parameters**	**ALK2^{KD}**	**BMPR2^{KD}**	**ALK2^{KD}/ BMPR2^{KD}**
I(0) (cm^{-1}) [from *P*(r)]	14.00 ± 0.26	61.07 ± 0.17	53.17 ± 0.46
R_g (Å) [from *P*(r)]	21.56 ± 0.64	23.48 ± 0.23	31.46 ± 0.41
I(0) (cm^{-1}) [from Guiner plot]	14.30± 0.28	60.84 ± 0.16	53.51 ± 0.58
R_g (Å) [from Guiner plot]	20.90 ± 0.57	23.49 ± 0.23	31.08 ± 0.51
D_{max} (Å)	70.57	75.74	98.04
Porod volume estimate ($Å^3$)	36,400	52,300	106,228
Dry volume estimate from sequence ($Å^3$)	40,100	42,800	90,500
Molecular mass determination			
Partial specific volume (cm^3 g^{-1})	0.7425	0.7425	0.7425
Contrast (10^{10} cm^{-2})	2.809	2.809	2.809
Molecular mass M_r [from *I*(0)]	33,100	38,525	74,325
Calculated M_r from sequence	34,604	39,204	73,789
Software employed			
Data processing	SasTool		
Ab initio analysis	DAMMIN		
Validation and averaging	DAMAVER		
Computation of model intensities	CRYSOL		
Three-dimensional graphics representations	PyMOL		

Supplemental Table 4. Data-collection and scattering derived parameters for the Small angle X-ray scattering (SAXS) analysis, collected at the Stanford synchrotron radiation lightsource BioSAXS beamline BL4-2

Supplementary Table 5
Agnew, Ayaz et al

Simulation length	Box size	Number of atoms	Referenced figure	System description	Motivation
40 X 20 µs	~107 Å	~120K	Fig. 2a Panels 1,2<br>Suppl Fig. 3	ALK2 residues 202-499, BMPR2 residues 197-510	ALK2 BMPR2 unbiased association
20 µs	~107 Å	~120K	Fig. 2b<br>Suppl Fig. 15a	ALK2 residues 202-499, BMPR2 residues 197-510	Assesment of the stability of the N dimer
2 µs	~100 Å	~100K	Fig. 2a Panel 3	ALK2 residues 183-499, BMPR2 residues 197-510 and ATP	Refinement of the N dimer after homology modelling of the GS domain*
10 µs	~105 Å	~120K	Fig. 2a Panel 3	ALK2 residues 202-499, BMPR2 residues 197-510 and ATP	Refinement of the C1 dimer
15 X 2 µs	~125 Å	~200K	Fig. 7a<br>Suppl Fig. 10a	2 X ALK2 residues 183-499**, 2 X BMPR2 residues 197-510 and ATP	Generation of the tetramer model
20 µs	~125 Å	~200K	Fig. 7a<br>Suppl Fig. 10d,e	2 X ALK2 residues 183-499**, 2 X BMPR2 residues 197-510 and ATP	Refinement of the tetramer model
20 x 10 µs	~112 Å	~140K	Suppl Fig. 10e<br>Supp Fig. 15d	ALK2 residues 202-499, BMPR2 residues 197-510 and ATP	Assesment of the stability of the C2 dimer as a dimer (extracted from final tetramer)
5 x 20 µs	~133 Å	~230K	Fig. 2b<br>Suppl Fig. 10e<br>Suppl Fig. 15c	ALK2 residues 202-499, BMPR2 residues 197-510 and ATP	Assesment of the stability of the C1 dimer
5 x 20 µs	~125 Å	~200K	Suppl Fig. 10e<br>Suppl Fig. 15b	ALK2 residues 183-499**, BMPR2 residues 197-510 and ATP	Assessment of the stability of the N dimer (extracted from final tetramer)

Supplementary Table 5. List and specifications of conducted molecular dynamics simulations, including references to the figures in which they are described. (*) denotes simulation of the N-lobe dimer run after modeling of the GS domain to the substrate-binding pocket of BMPR2 using a structure of the PKA kinase bound to a substrate peptide (Zheng et al., 1993). The refined N-lobe dimer model containing the GS domain then used in simulations to generate the tetramer model. (**) marks cases in which distance restraints were implemented between ALK2 residues 189, 191, 193 and BMPR2 residues 378, 380, 342 to ensure GS domain stability in the substrate-binding pocket of BMPR2.

Supplementary Table 6
Agnew, Ayaz et al

TABLE 6A

HDX-MS Data Summary Table

Data Set	$BMPR2^{KD}$	$ALK2^{KD}$	$BMPR2^{KD}$:$ALK2^{KD}$
HDX reaction details	50 mM HEPES, 50 mM KCl, 5 mM DTT, pD 7.60, 93% D_2O, 23 °C	50 mM HEPES, 50 mM KCl, 5 mM DTT, pD 7.60, 93% D_2O, 23 °C	50 mM HEPES, 50 mM KCl, 5 mM DTT, pD 7.60, 93% D_2O, 23 °C
HDX time course (sec)	15, 180, 900	15, 180, 900	15, 180, 900
HDX control samples	undeuterated controls; internal maximally-labeled estimation		
Back-exchange (mean / IQR)	13% / 8%		
# of Peptides	82	78	160
Sequence coverage	97%	91%	94%
Average peptide length / Redundancy	16.7 (3.5)	15.3 (4.1)	16.0 (3.8)
Replicates	3 (technical)	3 (technical)	3 (technical)
Repeatability (%D, average standard deviation)	0.97%	1.10%	0.68%
Significance testing	two-tailed, unpaired t test, p<0.005 at time point(s) approximating the middle range of exchange		

TABLE 6B

HDX-MS Data Summary Table

Data Set	$ALK2^{KD}$	$GS\text{-}ALK2^{KD}$
HDX reaction details	50 mM HEPES, 50 mM KCl, 5 mM DTT, pD 7.60, 93% D_2O, 23 °C	50 mM HEPES, 50 mM KCl, 5 mM DTT, pD 7.60, 93% D_2O, 23 °C
HDX time course (sec)	15, 180, 900	15, 180, 900
HDX control samples	undeuterated controls	
Back-exchange (mean / IQR)	not determined	
# of Peptides	67	67
Sequence coverage	91%	91%
Average peptide length / Redundancy	15.9 (3.5)	15.9 (3.5)
Replicates	3 (technical)	3 (technical)
Repeatability (%D, average standard deviation)	1.01%	0.69%
Significance testing	two-tailed, unpaired t test, $p<0.005$ at time point(s) approximating the middle range of exchange	

TABLE 6C

HDX-MS Data Summary Table

Data Set	$BMPR2^{KD}$	$GS\text{-}ALK2^{KD}$	$BMPR2^{KD}:GS\text{-}ALK2^{KD}$
HDX reaction details	50 mM HEPES, 50 mM KCl, 5 mM DTT, pD 7.60, 93% D_2O, 23 °C	50 mM HEPES, 50 mM KCl, 5 mM DTT, pD 7.60, 93% D_2O, 23 °C	50 mM HEPES, 50 mM KCl, 5 mM DTT, pD 7.60, 93% D_2O, 23 °C
HDX time course (sec)	15, 180, 900	15, 180, 900	15, 180, 900
HDX control samples	undeuterated controls; internal maximally-labeled estimation		
Back-exchange (mean / IQR)	15% / 4%		
# of Peptides	86	69	155
Sequence coverage	97%	87%	94%
Average peptide length / Redundancy	16.4(3.6)	15.6(3.0)	16.2(3.6)
Replicates	3 (technical)	3 (technical)	3 (technical)
Repeatability (%D, average standard deviation)	1.0%	1.0%	0.7%
Significance testing	two-tailed, unpaired t test, p<0.005 at time point(s) approximating the middle range of exchange		

TABLE 6D

HDX-MS Data Summary Table

Data Set	BMPR2^{KD}:GS-ALK2^{KD}	BMPR2^{KD}:ALK2^{KD}
HDX reaction details	50 mM HEPES, 50 mM KCl, 5 mM DTT, pD 7.60, 93% D_2O, 23.5 °C	50 mM HEPES, 50 mM KCl, 5 mM DTT, pD 7.60, 93% D_2O, 23.5 °C
HDX time course (sec)	15, 180, 900	15, 180, 900
HDX control samples	undeuterated controls; internal maximally-labeled estimation	
Back-exchange (mean / IQR)	15% / 5%	
# of Peptides	143	143
Sequence coverage	95%	95%
Average peptide length / Redundancy	16.2 (3.6)	16.2 (3.6)
Replicates	3 (technical)	3 (technical)
Repeatability (%D, average standard deviation)	0.73%	1.18%
Significance testing	two-tailed, unpaired t test, p<0.005 at time point(s) approximating the middle range of exchange	

Supplementary Table 6. HDX-MS Data Summary Table. HDX analysis was performed by automated computational processing using HDX Workbench software and manual curation of each peptide, state, and time point. A summary of the data analysis is presented in the standardized format recommended by HDX community guidelines (Masson et al., 2019). Back exchange is estimated from peptides derived from the disordered N-terminus. Peptide count and redundancy is calculated based on one representative charge state per peptic product.

References:

Winn, M.D., Ballard, C.C., Cowtan, K.D., Dodson, E.J., Emsley, P., Evans, P.R., Keegan, R.M., Krissinel, E.B., Leslie, A.G., McCoy, A., *et al.* (2011). Overview of the CCP4 suite and current developments. Acta Crystallogr D Biol Crystallogr. *67*, 235-42

Zheng, J., Knighton, D.R., ten Eyck, L.F., Karlsson, R., Xuong, N., Taylor, S.S., SowadskiJ.M. (1993). Crystal structure of the catalytic subunit of cAMP-dependent protein kinase complexed with MgATP and peptide inhibitor. Biochemistry 32, 2154-61.

Masson, G.R., Burke, J.E., Ahn, N.G., Anand, G.S., Borchers, C., Brier, S., Bou-Assaf, G.M., Engen, J.R., Englander, S.W., Faber, J., *et al.* (2019). Recommendations for performing, interpreting and reporting hydrogen deuterium exchange mass spectrometry (HDX-MS) experiments. Nature Methods *16*, 595-602.
