# Supplementary material for: Structural basis for ALK2/BMPR2 receptor complex signaling through kinase domain oligomerization
Source: Nat Commun. 2021 Aug 16;12:4950. doi: 10.1038/s41467-021-25248-5 (PMC8368100; doi:10.1038/s41467-021-25248-5)
Supplement: Supplementary file 3 — Description of Additional Supplementary Files [file 41467_2021_25248_MOESM3_ESM.pdf]

Description of Additional Supplementary Files

File Name: Supplementary Data 1
Description: Structural coordinates for the MD-derived C1 dimer model.

File Name: Supplementary Data 2
Description: Structural coordinates for the MD-derived BMPR2/ALK2 kinase domain tetramer.

File Name: Supplementary Data 3
Description: H/D exchange uptake plots of $ALK2^{KD}$ and $BMPR2^{KD}$ peptic peptides. Relative percent deuterium exchange across sample time points are plotted as pairwise comparisons of kinase samples. Exchange time courses were performed in triplicate as technical replicates of three independent exchange time courses. Error bars represent standard deviations (n=3). Peptide charge states were analyzed independently, and one charge state is reported per peptide.
