# Supplementary material for: Structural basis for ALK2/BMPR2 receptor complex signaling through kinase domain oligomerization
Source: Nat Commun. 2021 Aug 16;12:4950. doi: 10.1038/s41467-021-25248-5 (PMC8368100; doi:10.1038/s41467-021-25248-5)

Alk2 or BMPR2 vs. Alk2:BMPR2 complex - HDX-MS Uptake Plots

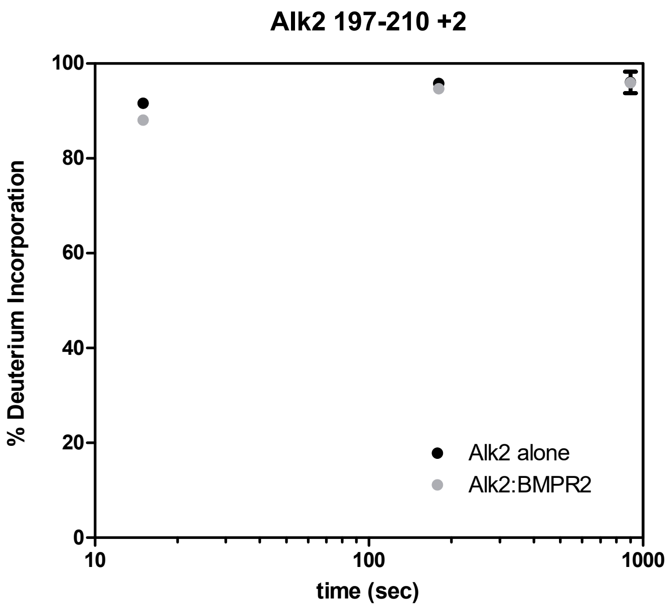

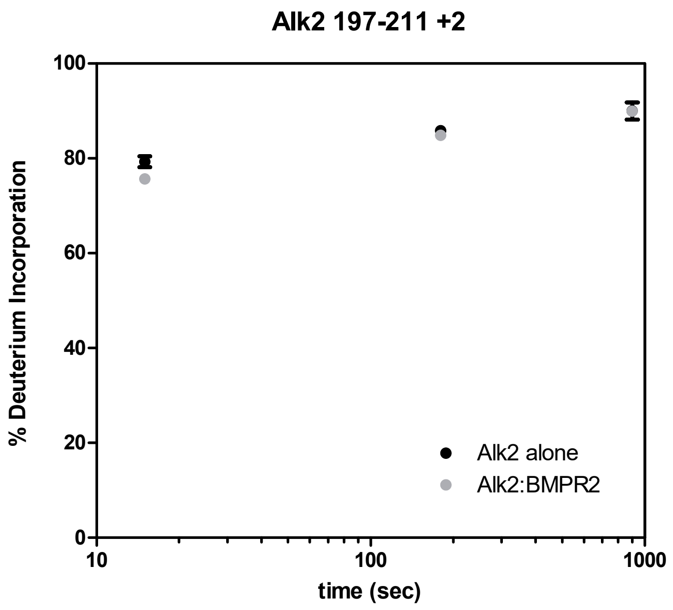

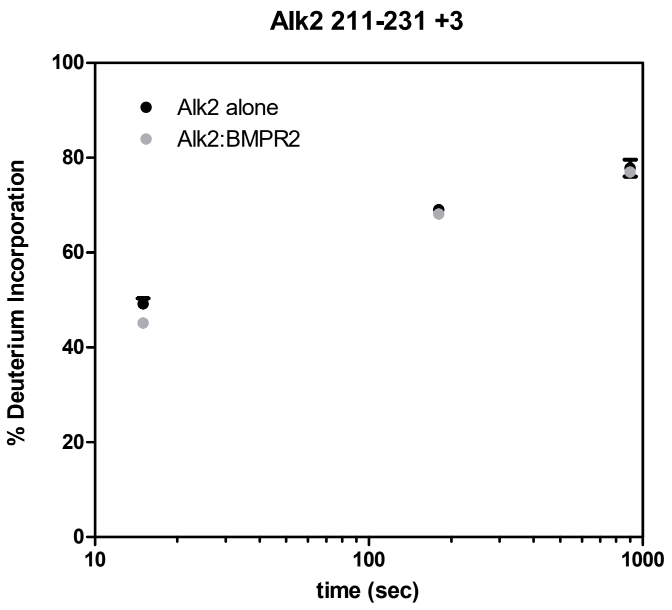

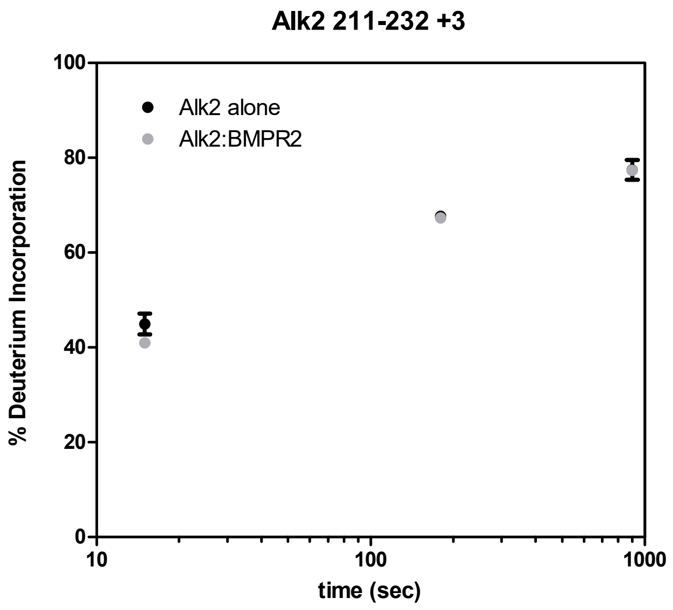

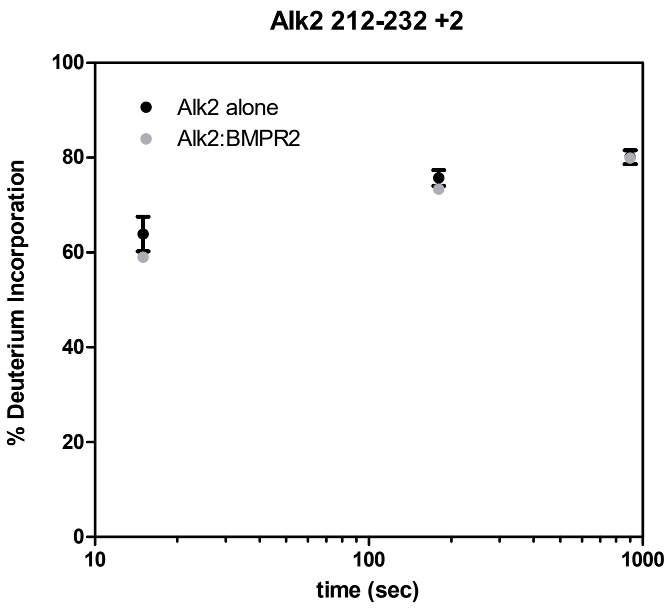

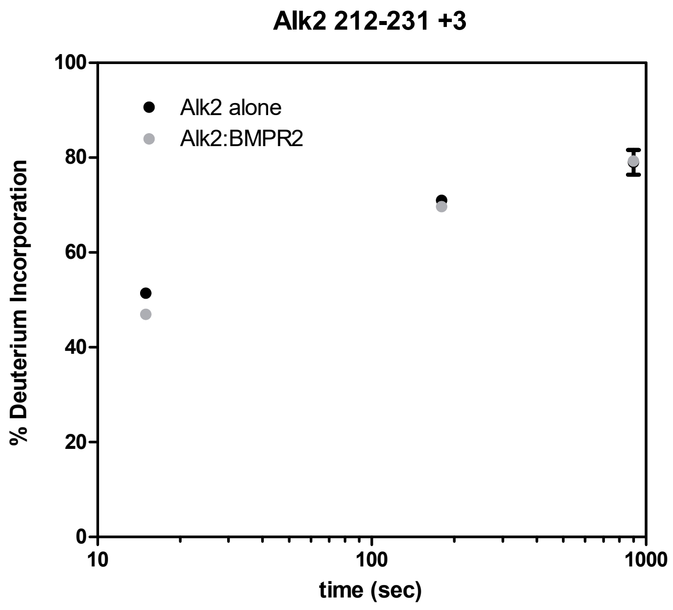

Alk2 or BMPR2 vs. Alk2:BMPR2 complex - HDX-MS Uptake Plots

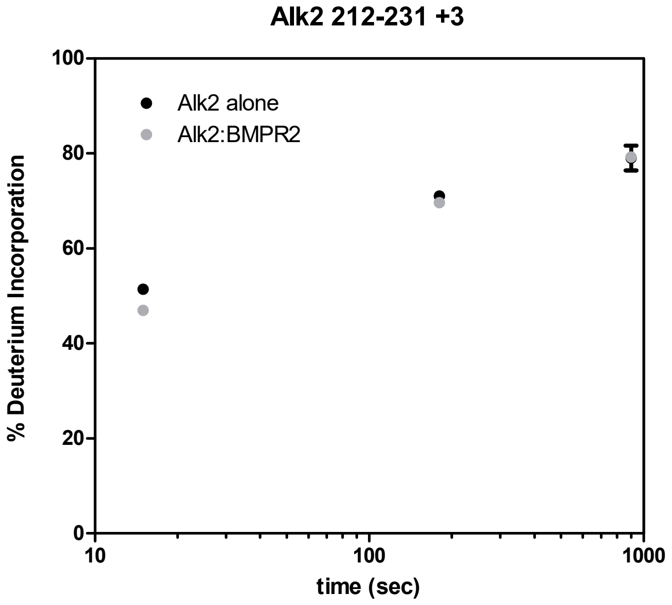

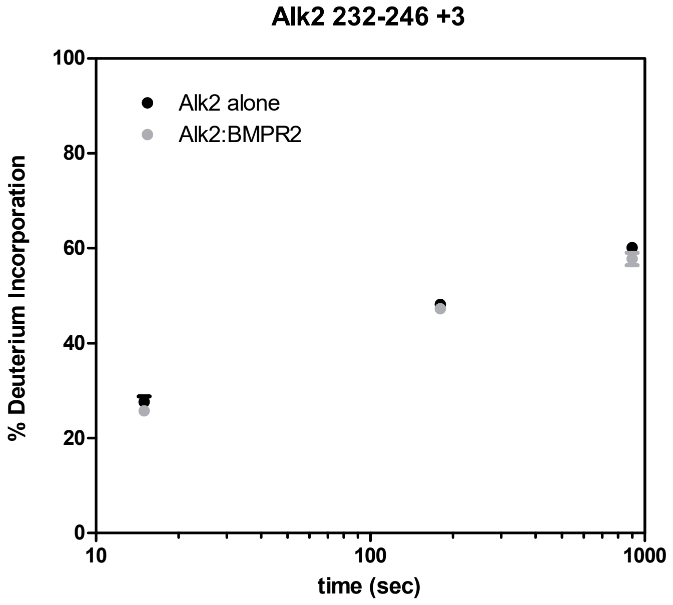

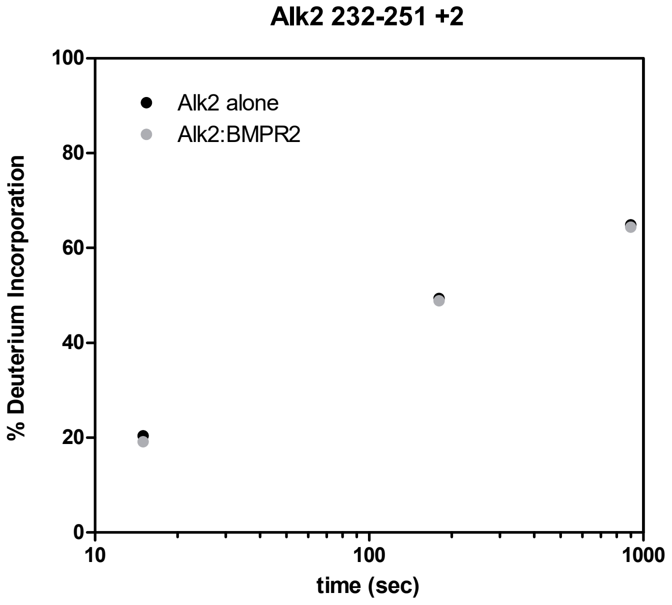

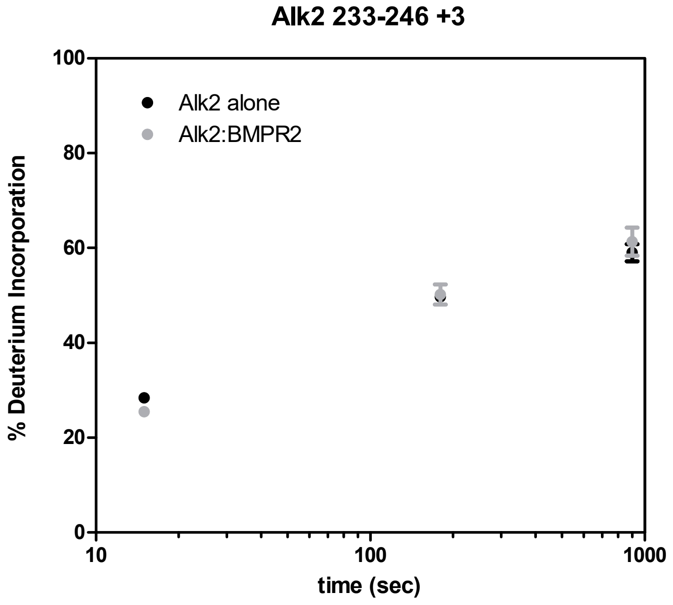

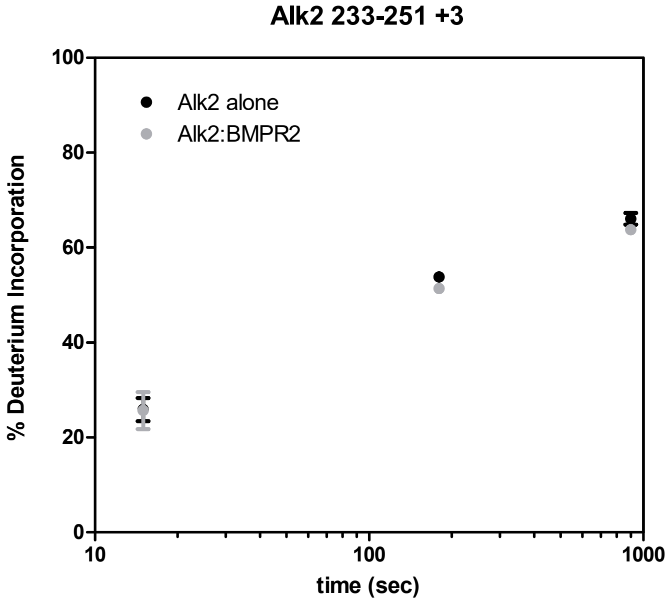

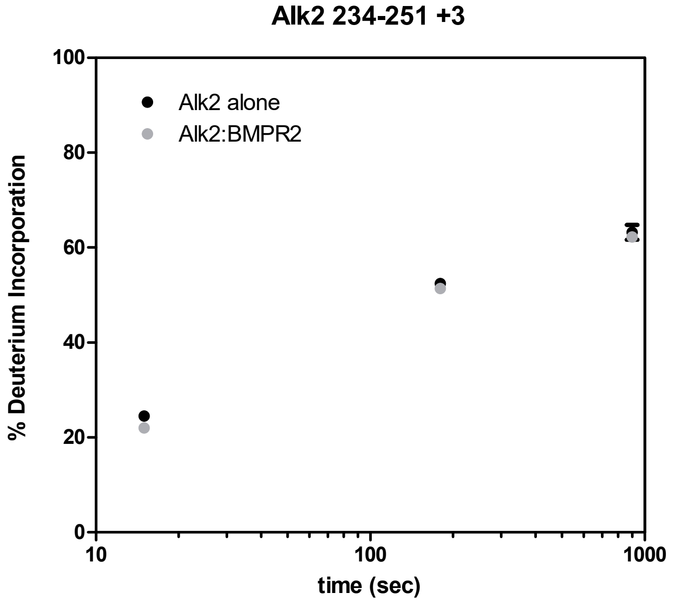

Alk2 or BMPR2 vs. Alk2:BMPR2 complex - HDX-MS Uptake Plots

Alk2 252-265 +2

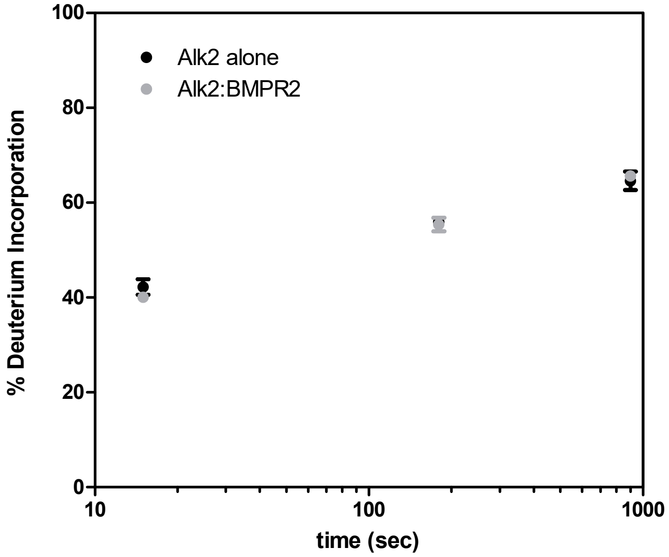

Alk2 255-265 +2

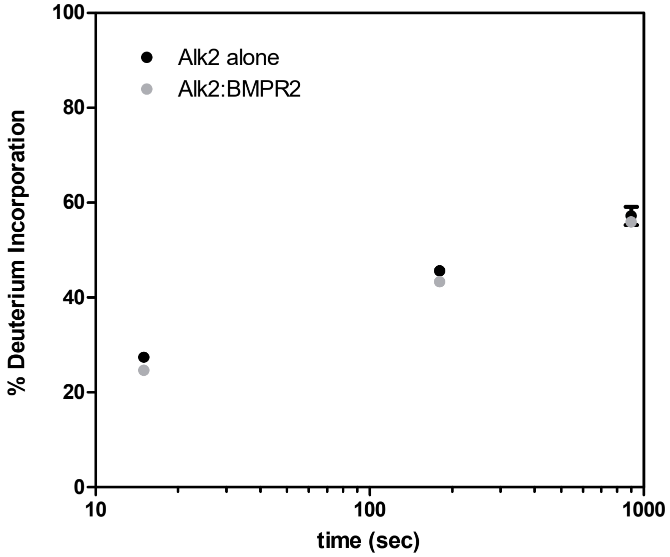

Alk2 257-263 +2

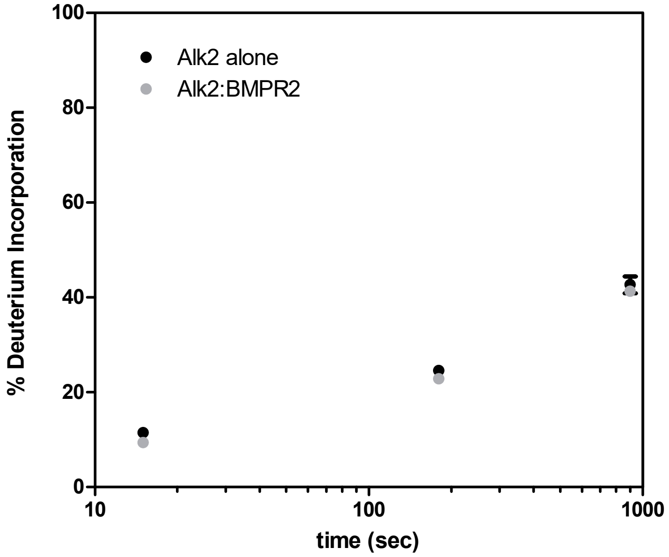

Alk2 257-265 +2

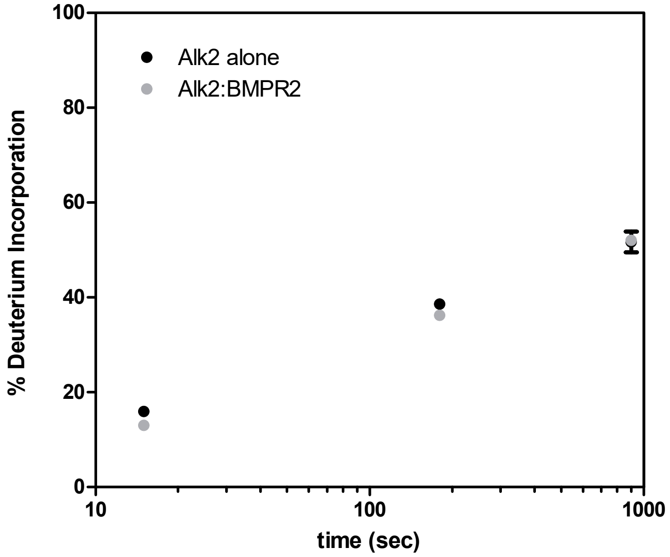

Alk2 257-279 +3

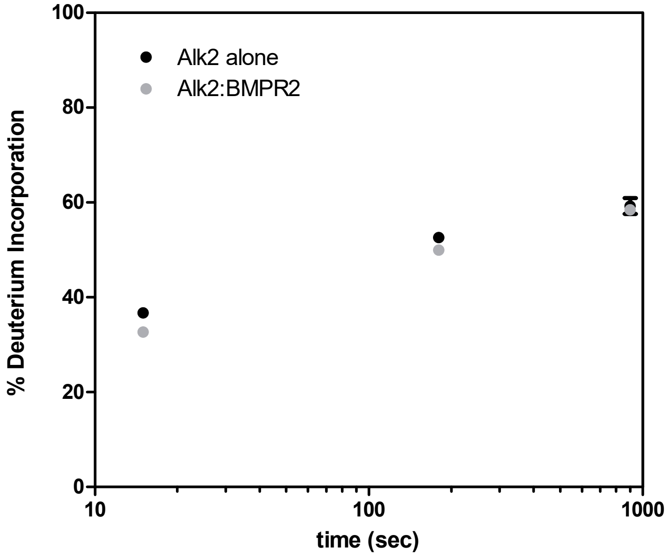

Alk2 264-279 +2

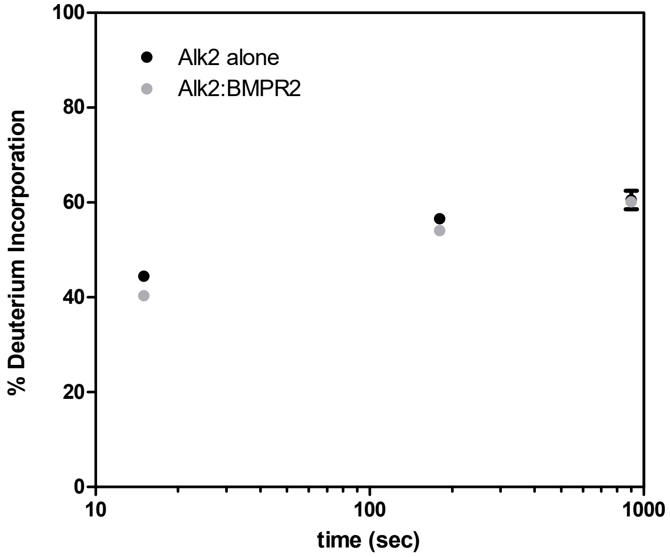

Alk2 or BMPR2 vs. Alk2:BMPR2 complex - HDX-MS Uptake Plots

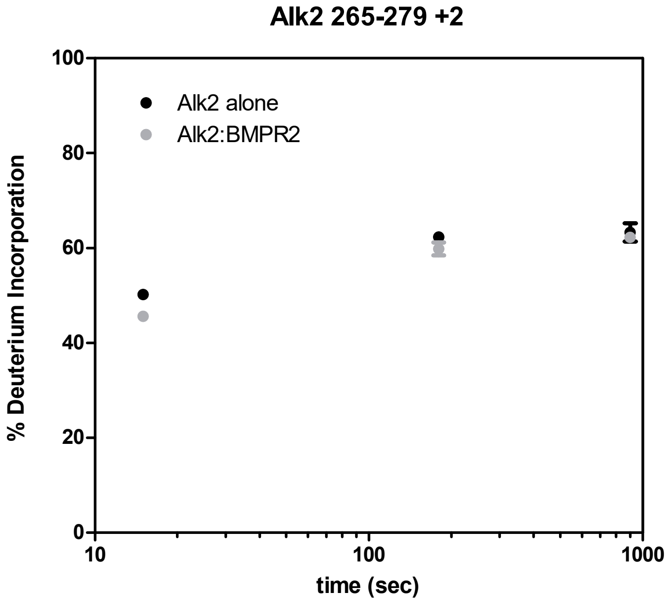

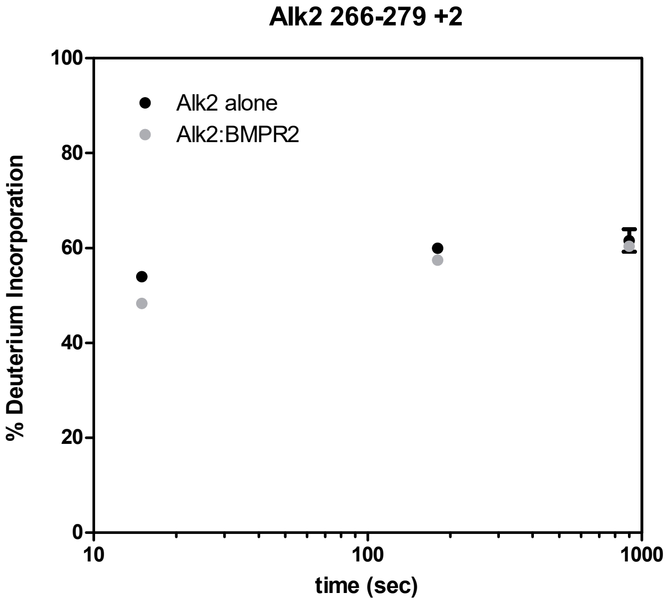

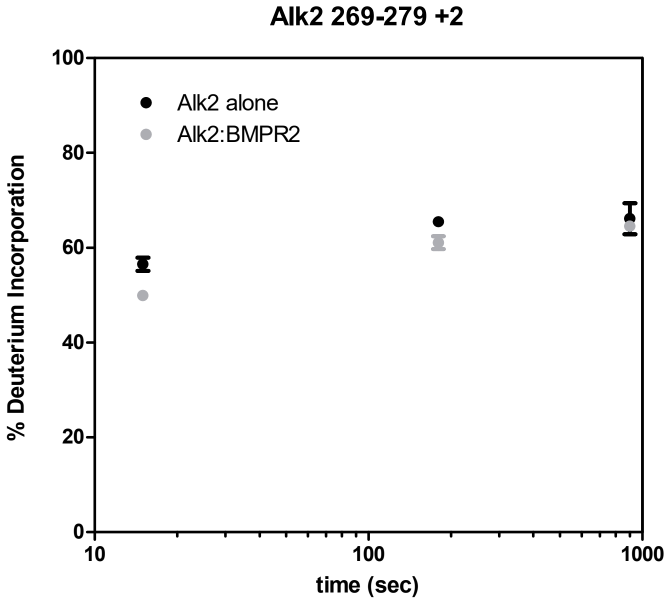

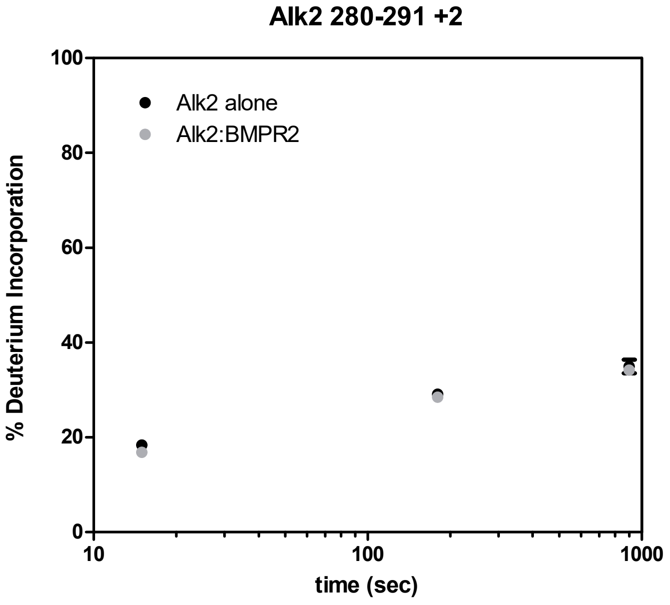

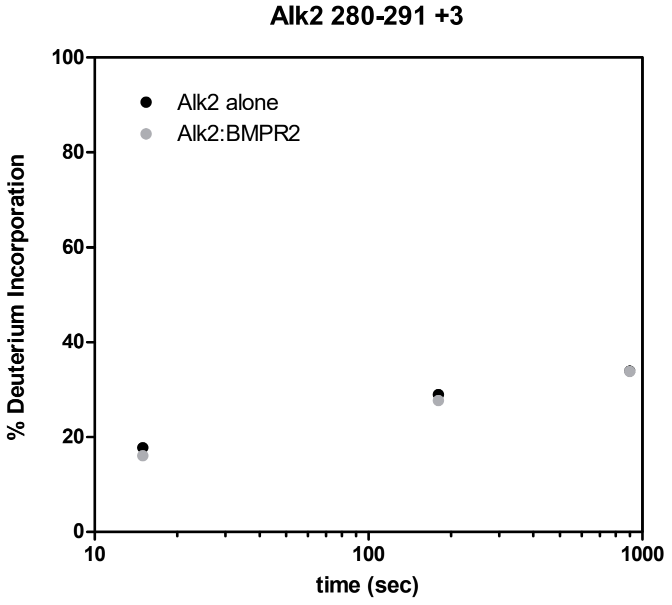

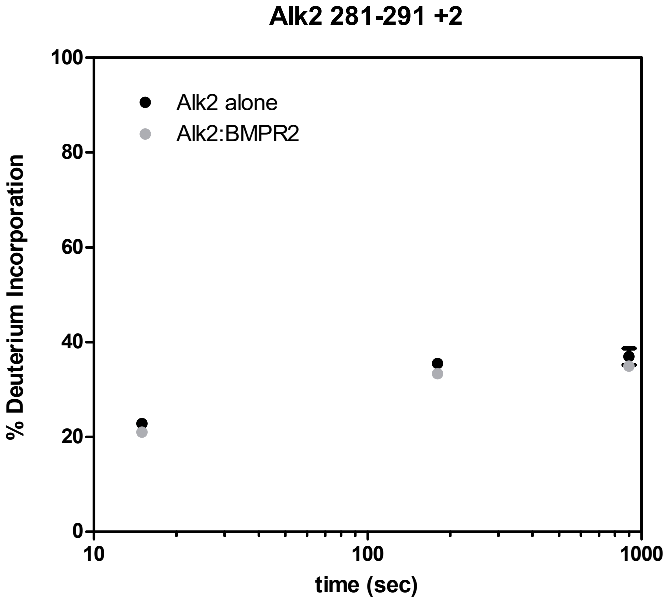

Alk2 or BMPR2 vs. Alk2:BMPR2 complex - HDX-MS Uptake Plots

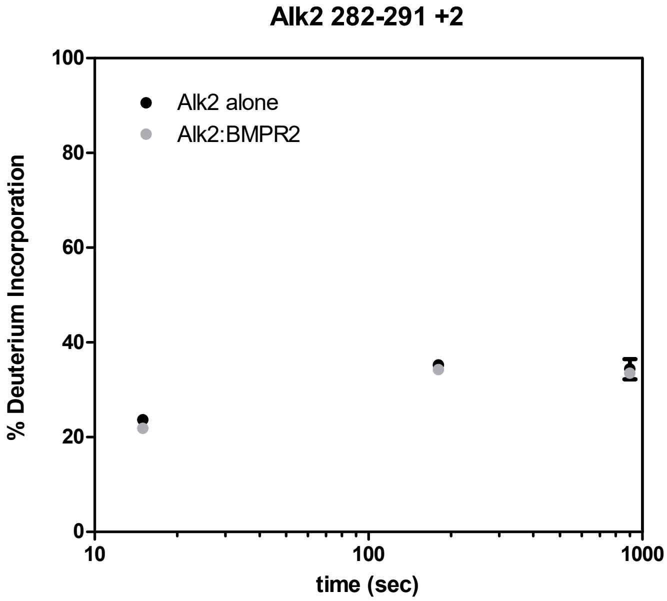

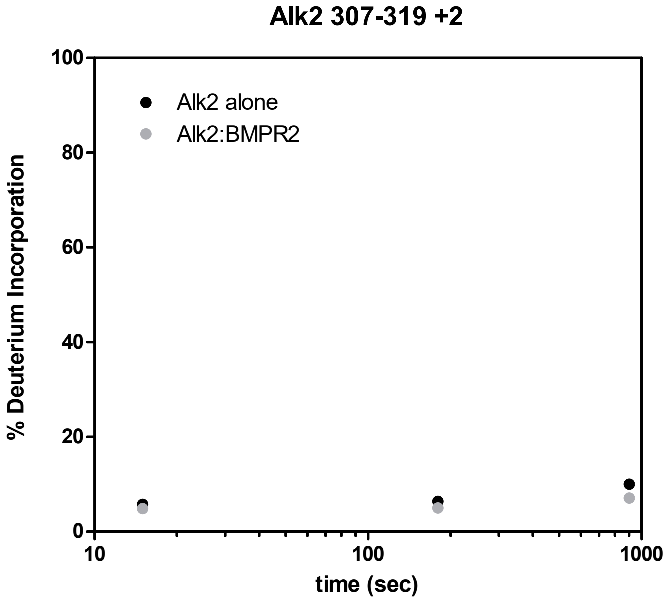

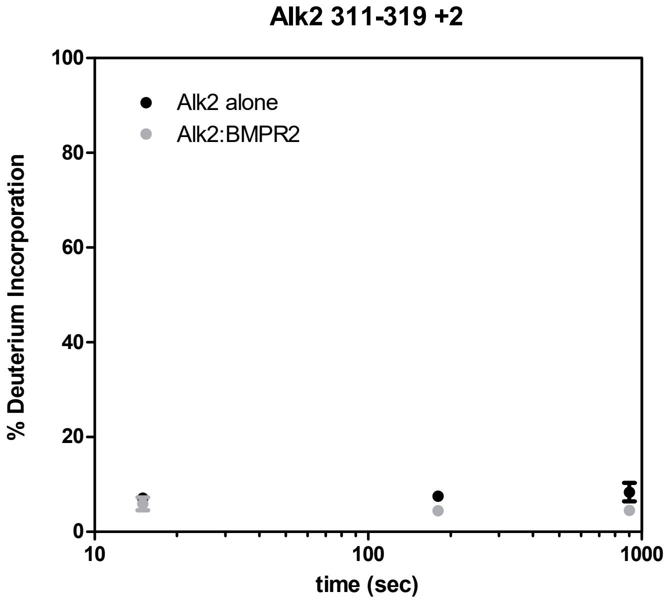

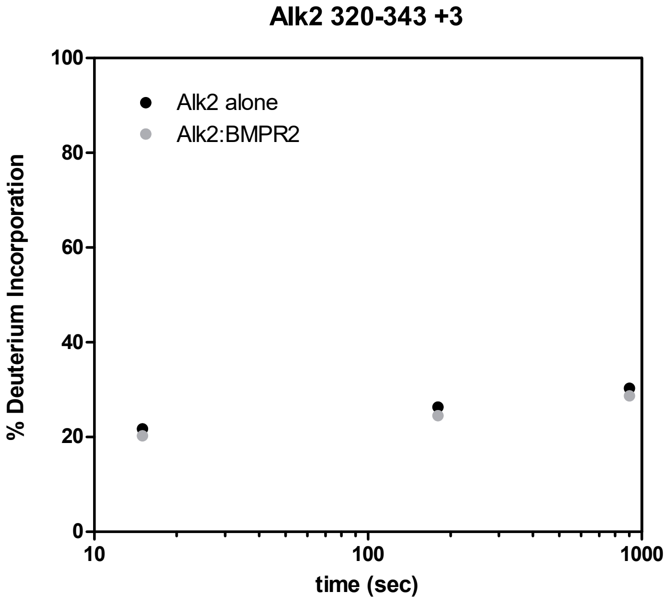

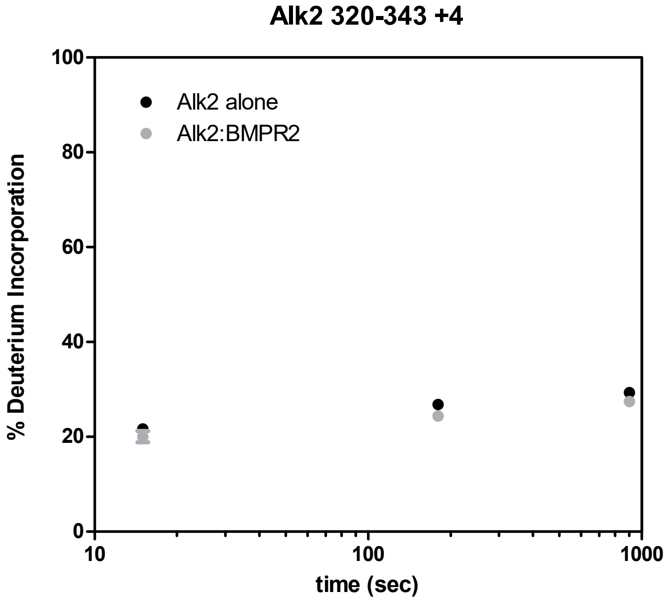

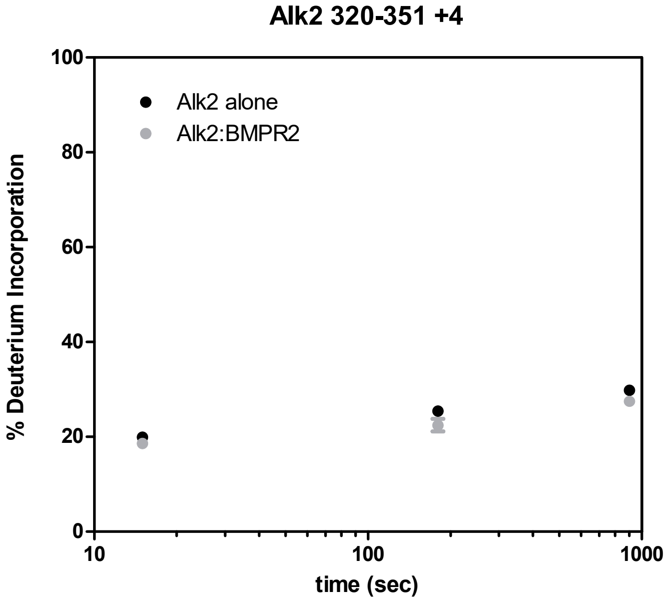

Alk2 or BMPR2 vs. Alk2:BMPR2 complex - HDX-MS Uptake Plots

Alk2 320-352 +4

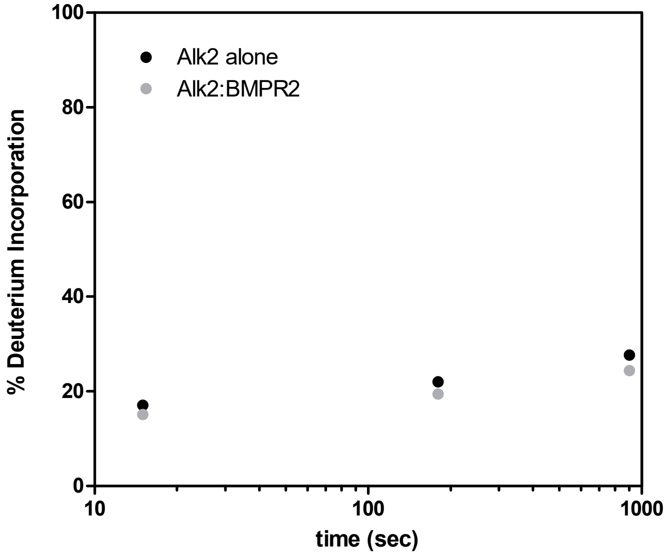

Alk2 344-352 +2

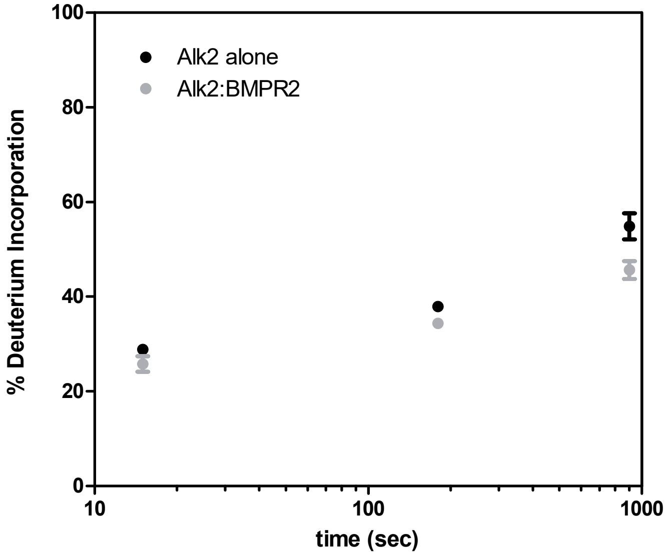

Alk2 358-368 +2

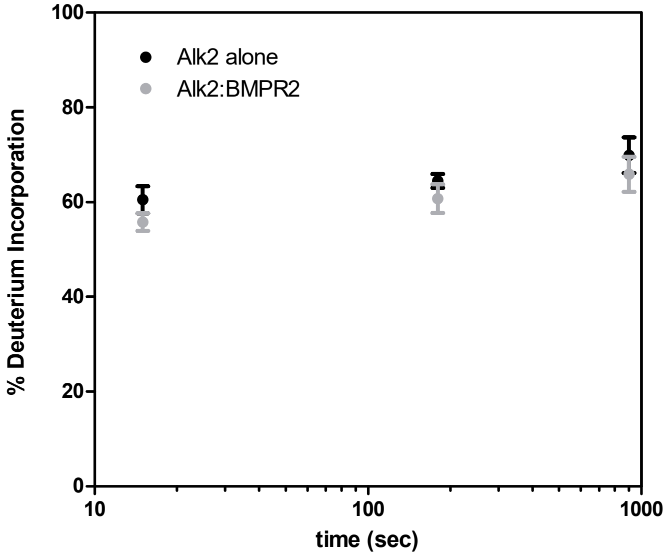

Alk2 358-387 +4

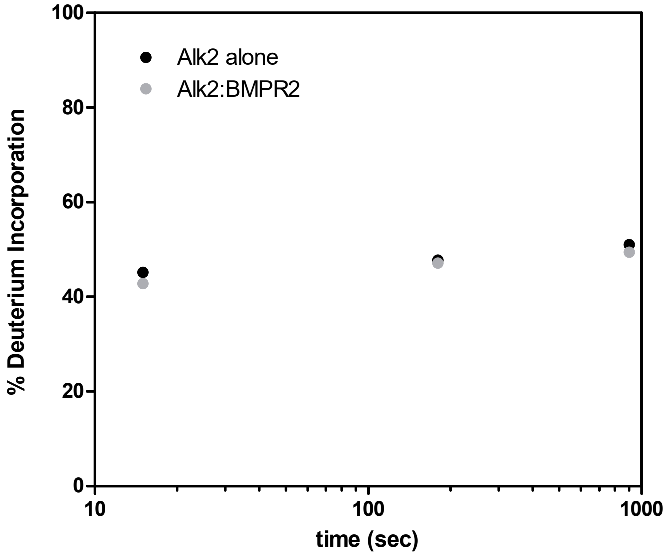

Alk2 369-387 +3

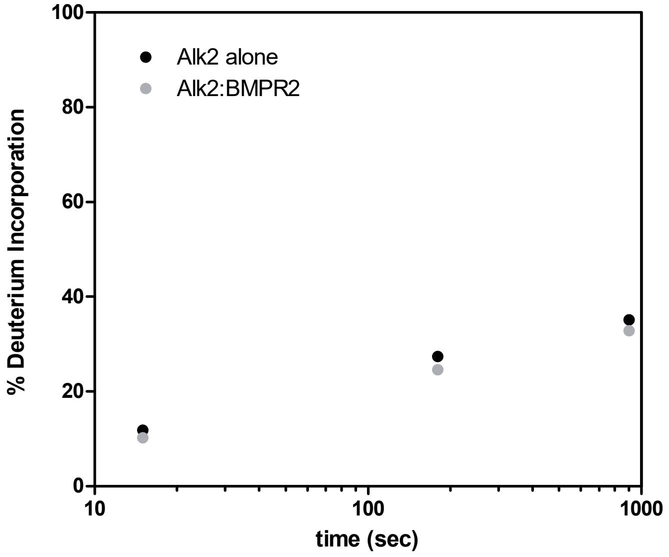

Alk2 395-406 +2

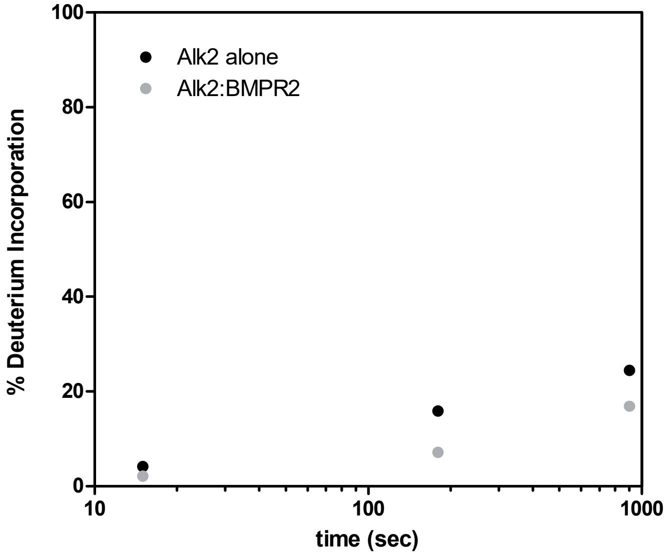

Alk2 or BMPR2 vs. Alk2:BMPR2 complex - HDX-MS Uptake Plots

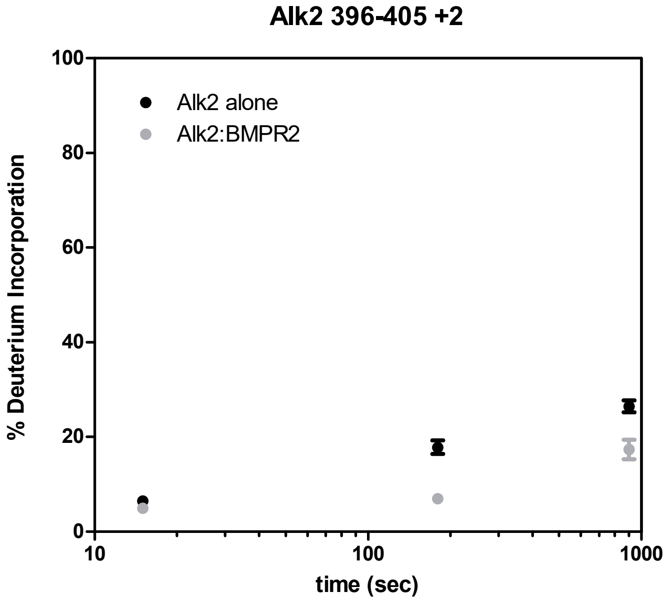

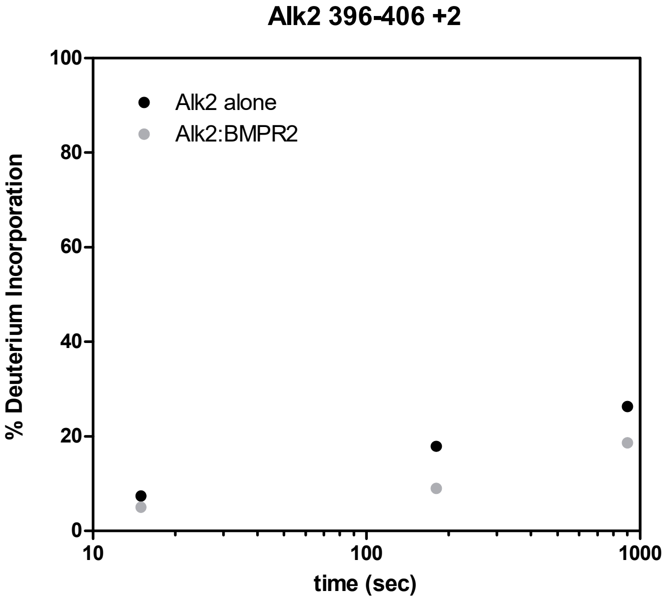

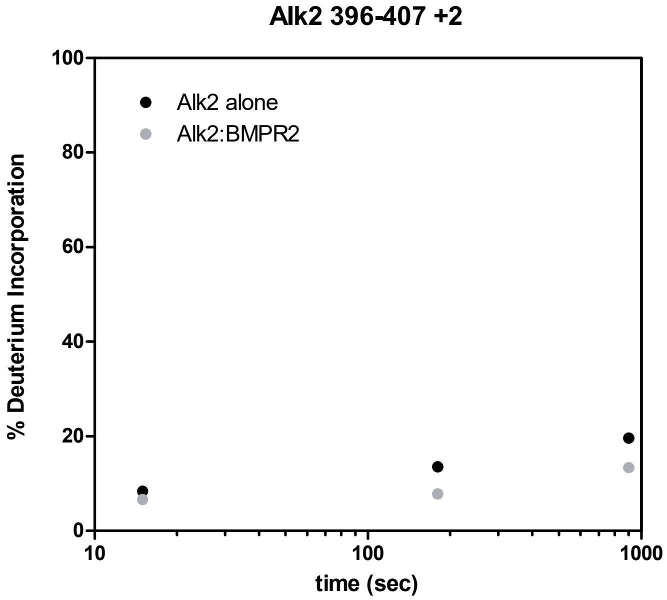

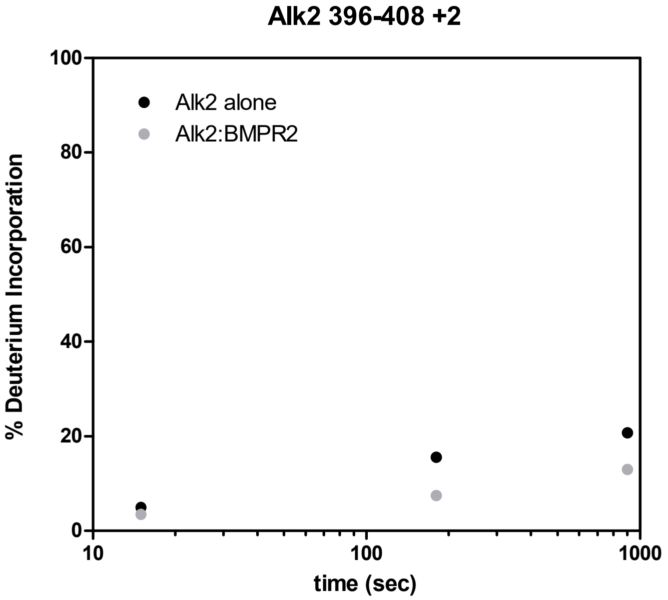

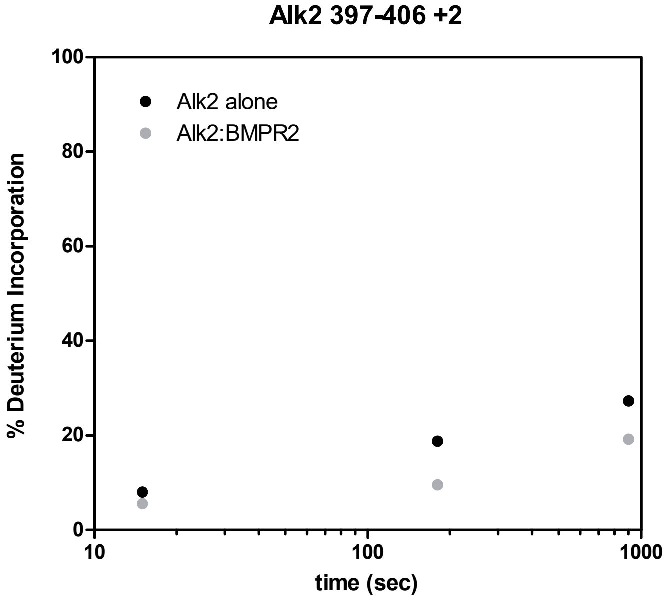

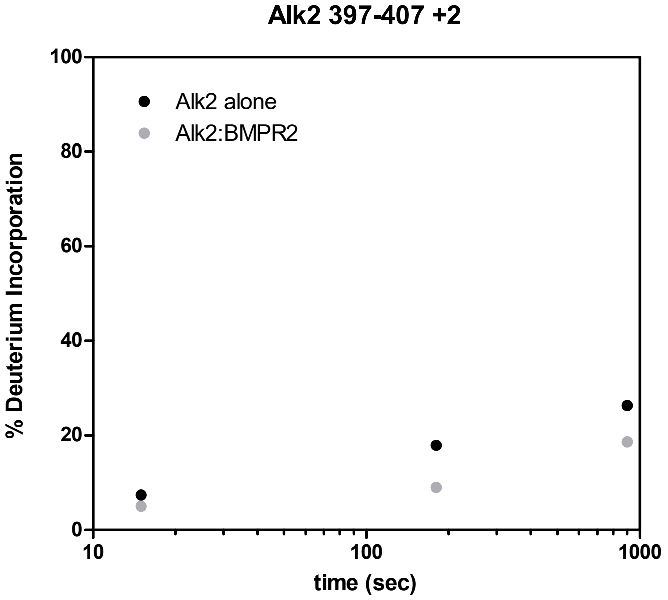

Alk2 or BMPR2 vs. Alk2:BMPR2 complex - HDX-MS Uptake Plots

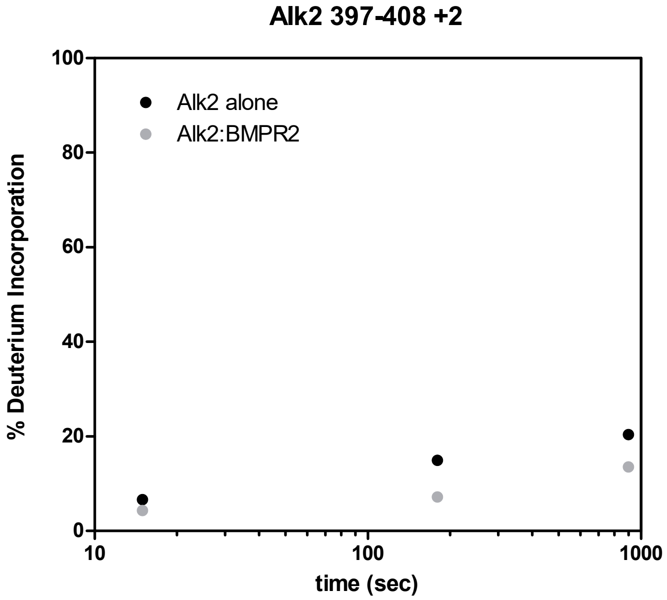

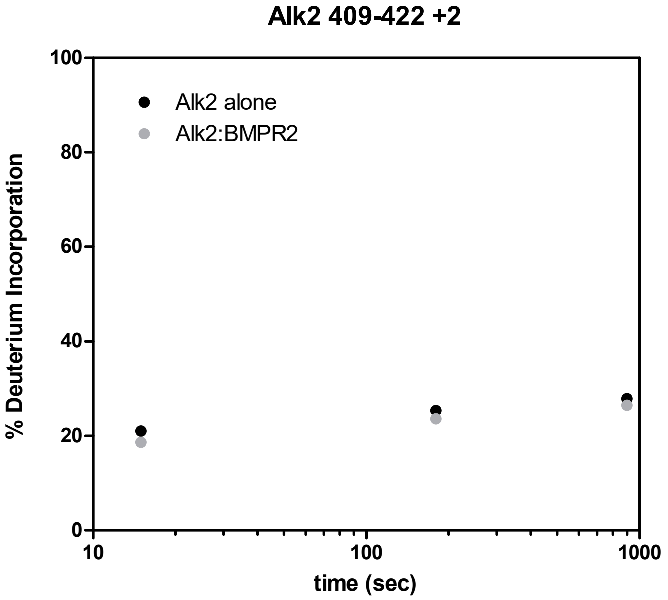

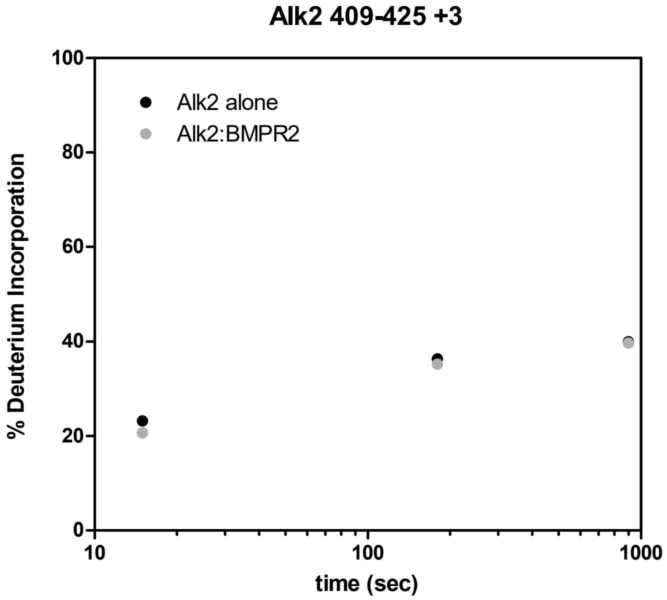

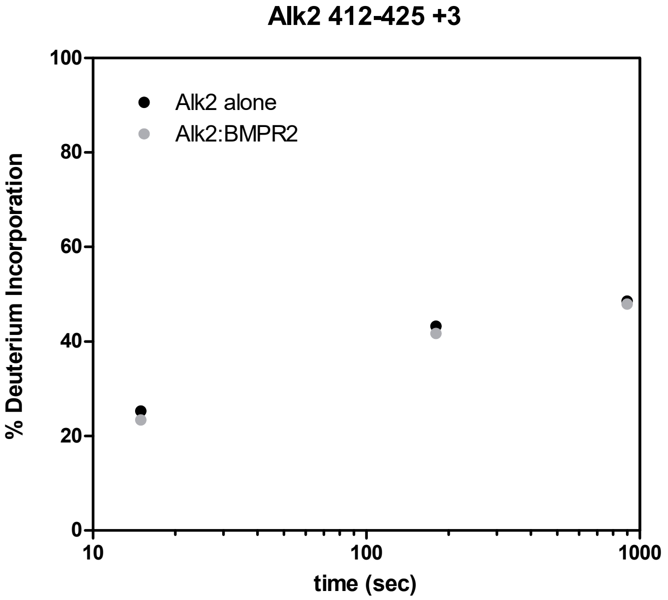

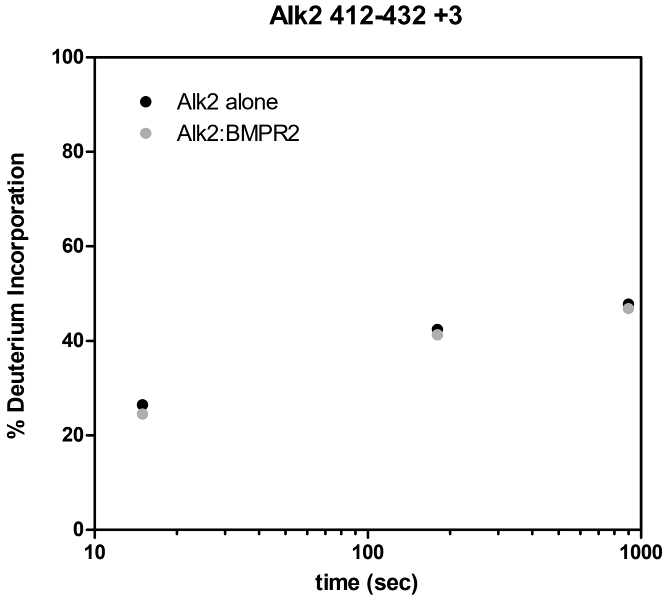

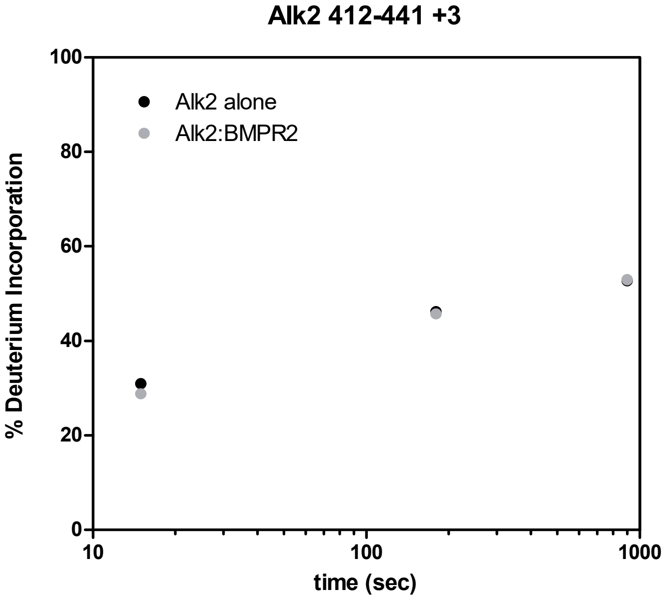

Alk2 or BMPR2 vs. Alk2:BMPR2 complex - HDX-MS Uptake Plots

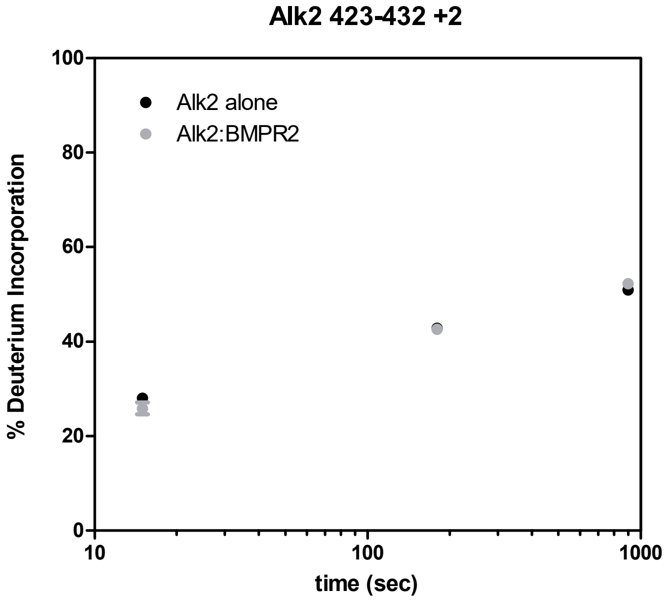

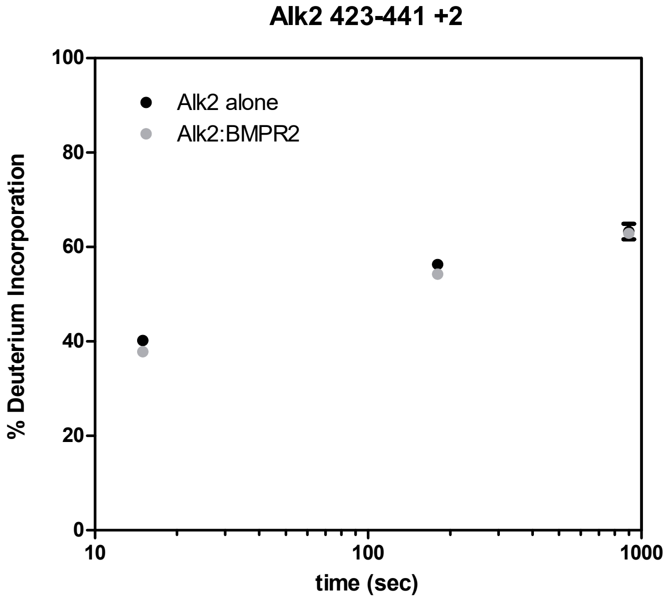

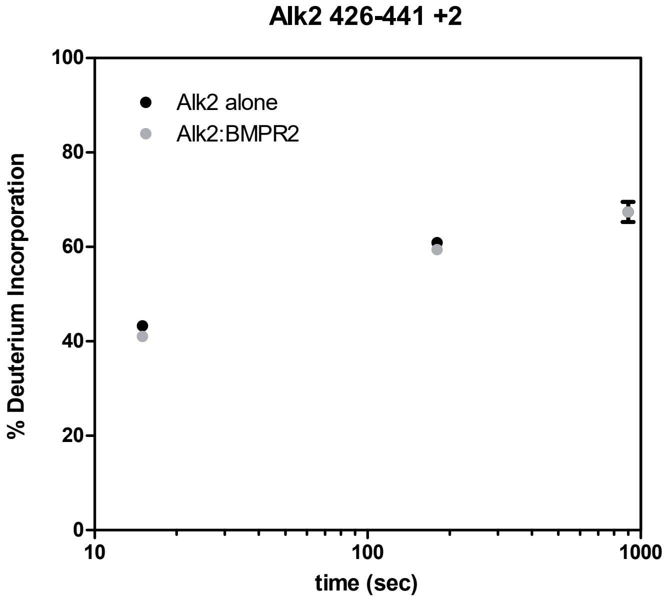

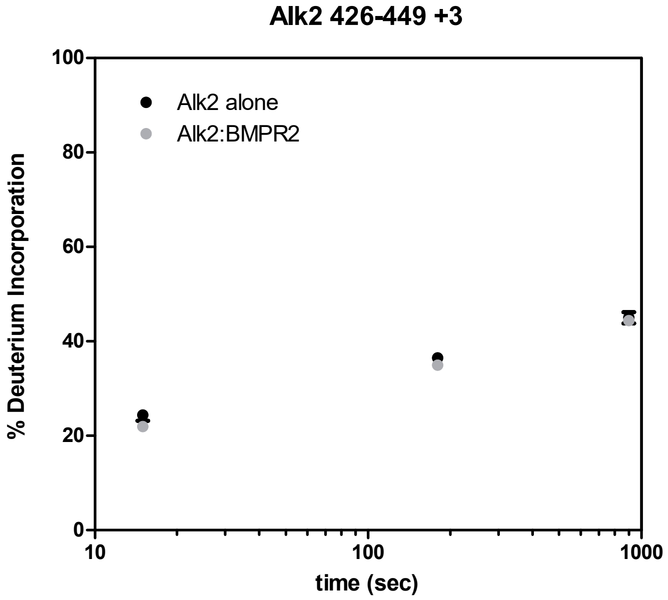

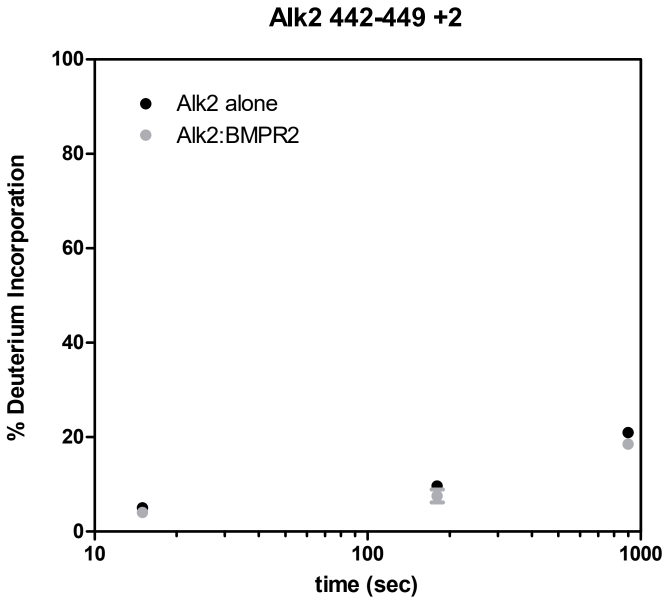

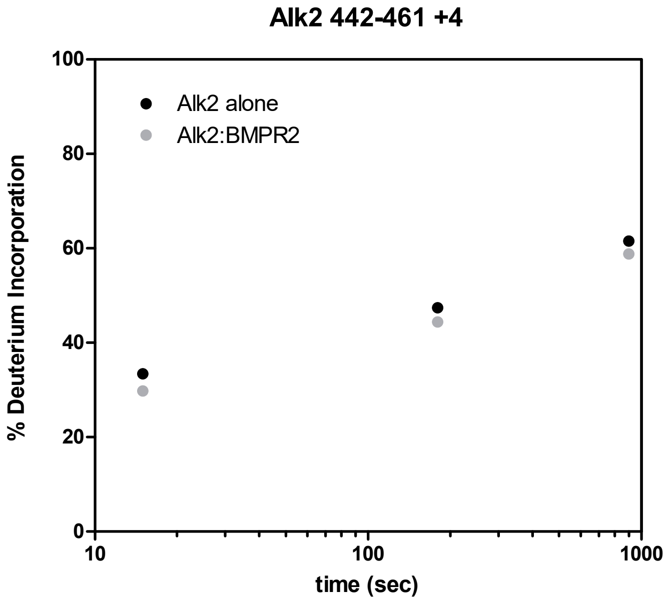

Alk2 or BMPR2 vs. Alk2:BMPR2 complex - HDX-MS Uptake Plots

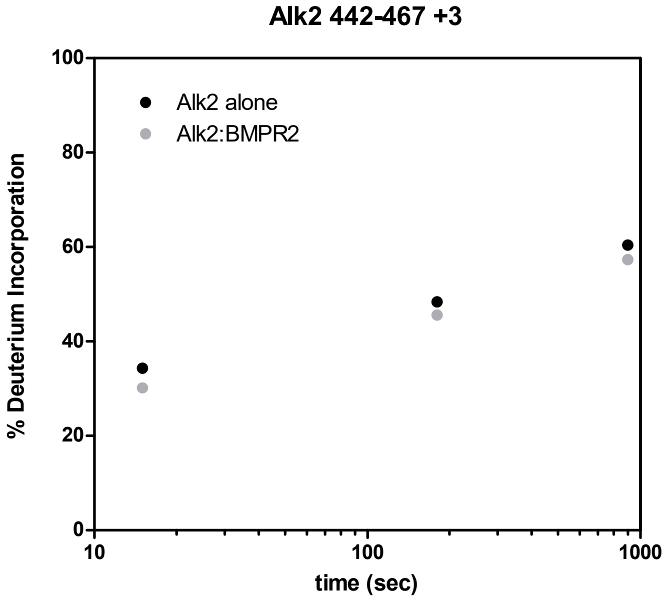

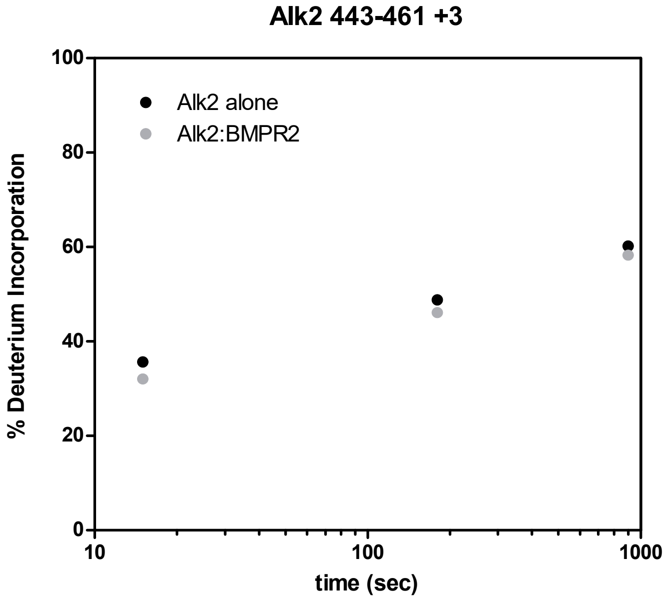

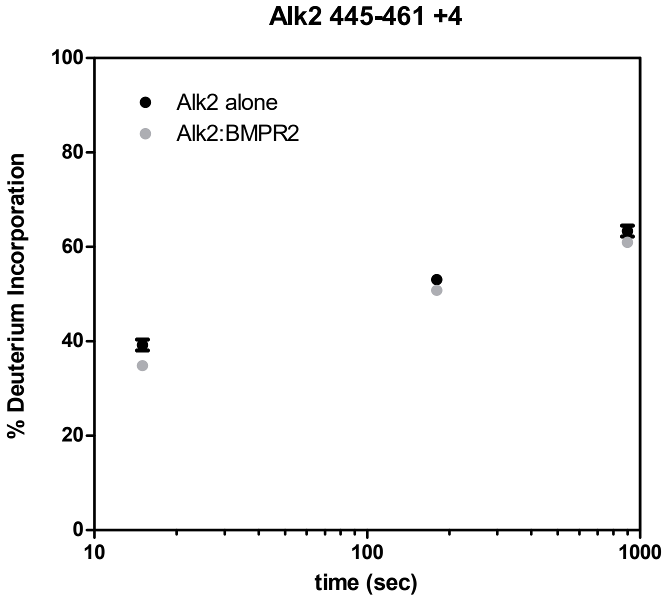

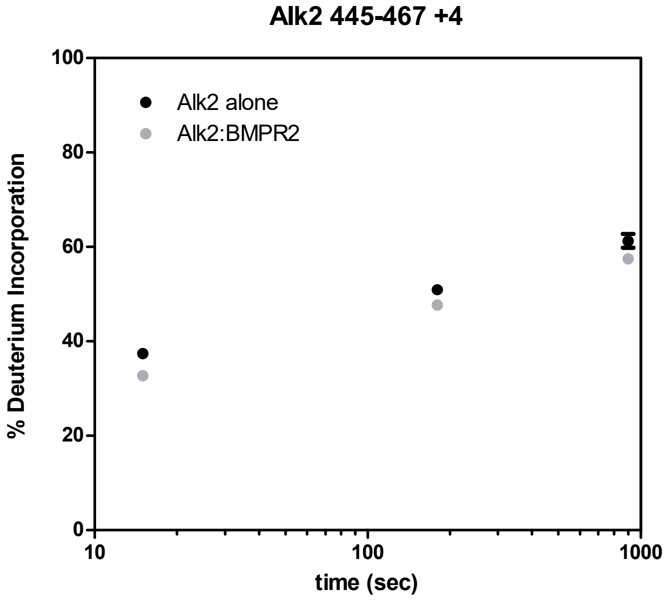

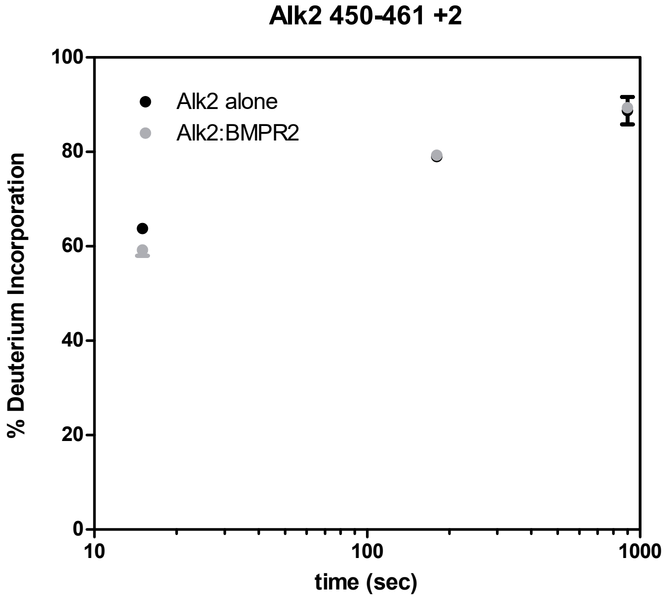

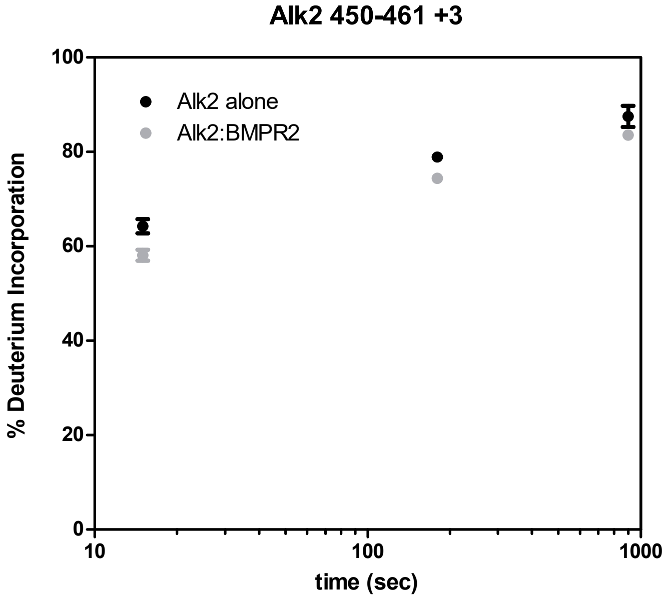

Alk2 or BMPR2 vs. Alk2:BMPR2 complex - HDX-MS Uptake Plots

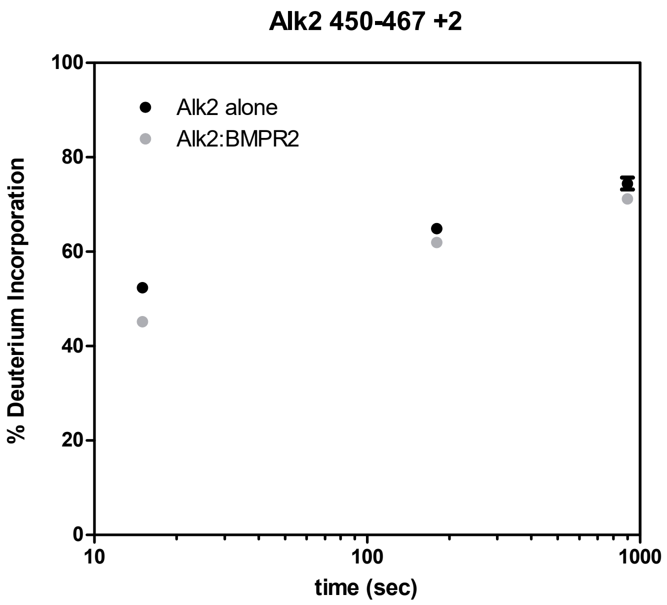

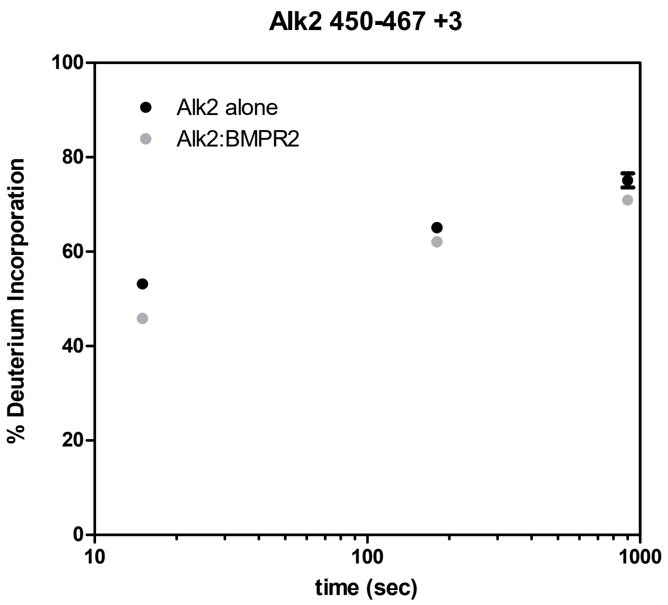

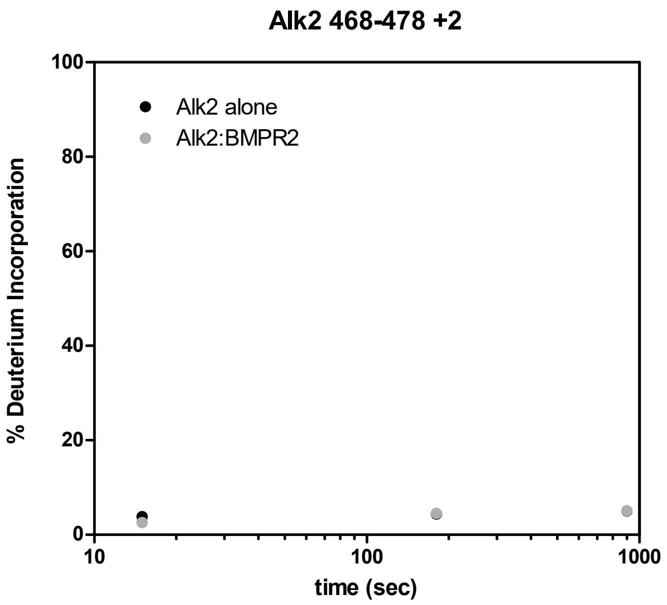

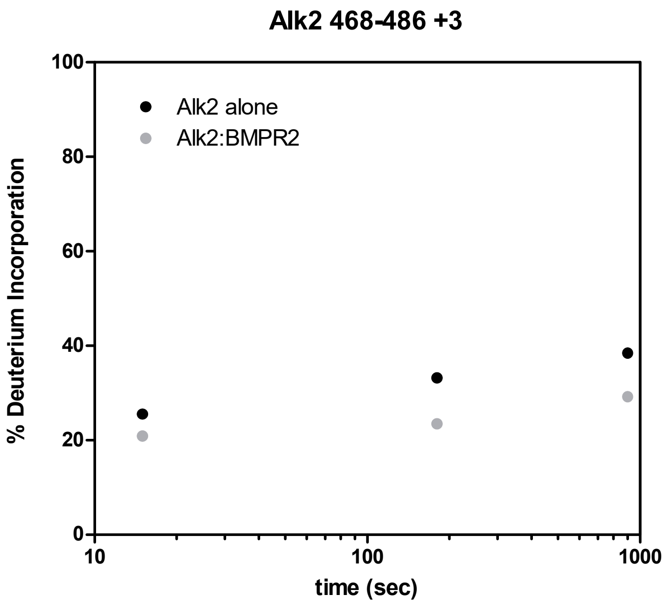

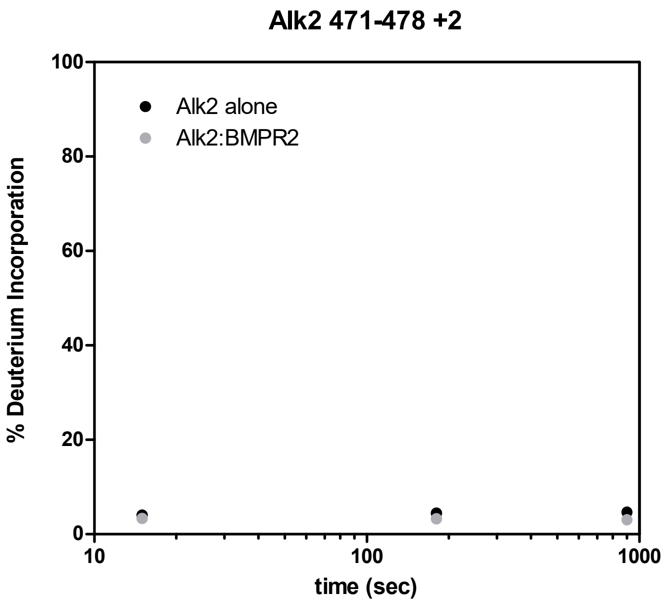

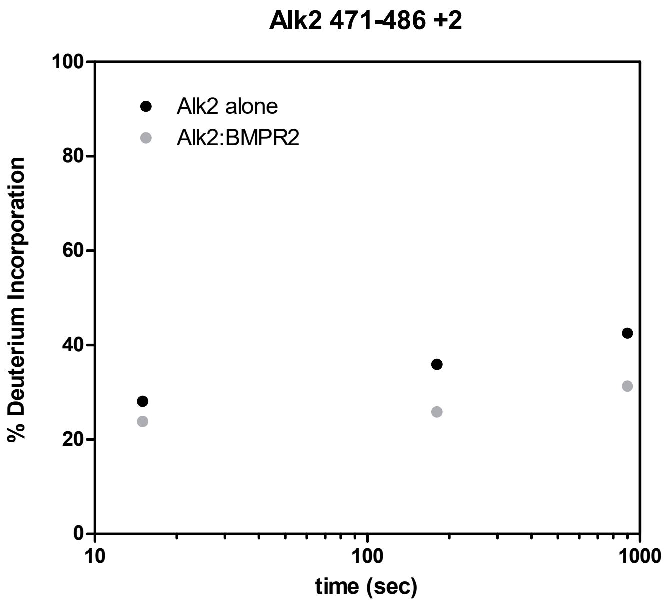

Alk2 or BMPR2 vs. Alk2:BMPR2 complex - HDX-MS Uptake Plots

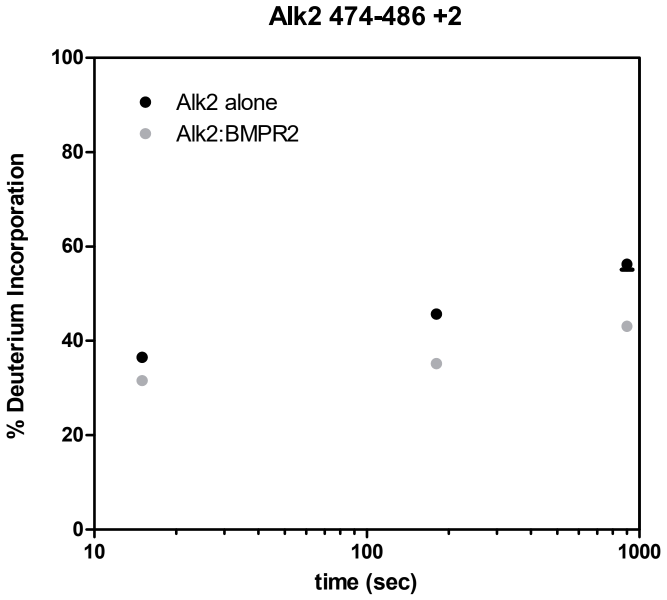

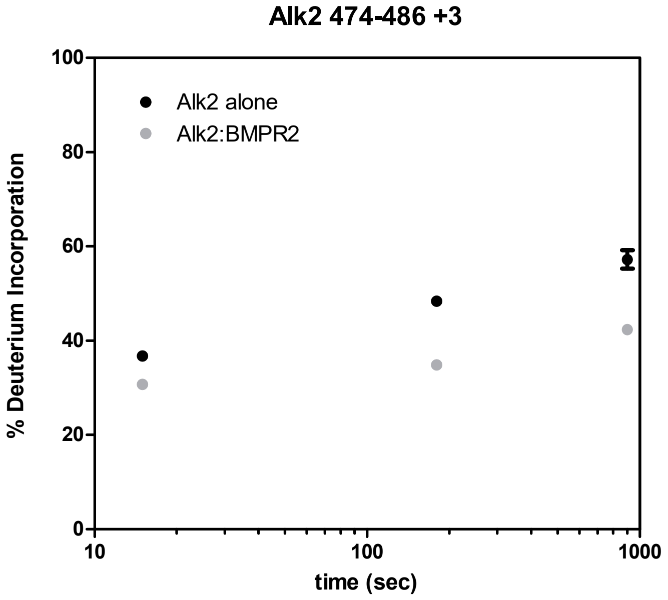

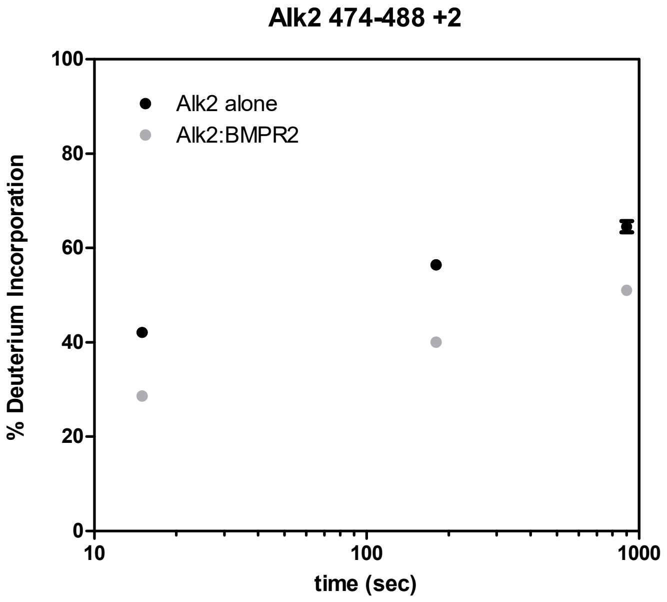

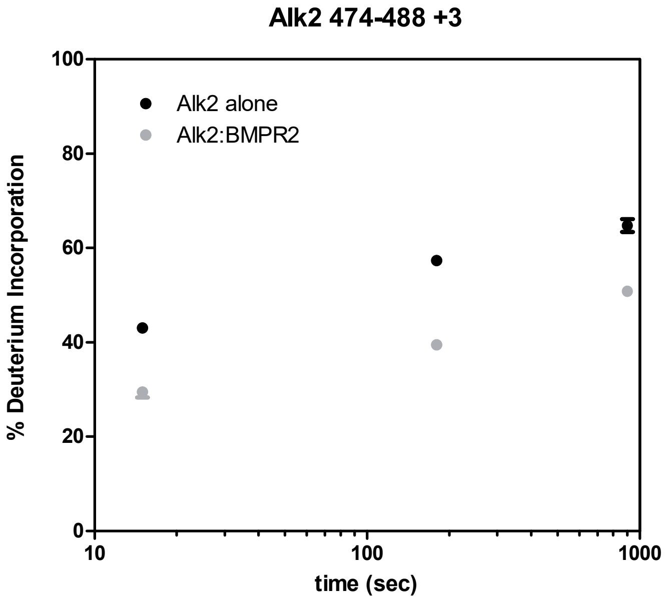

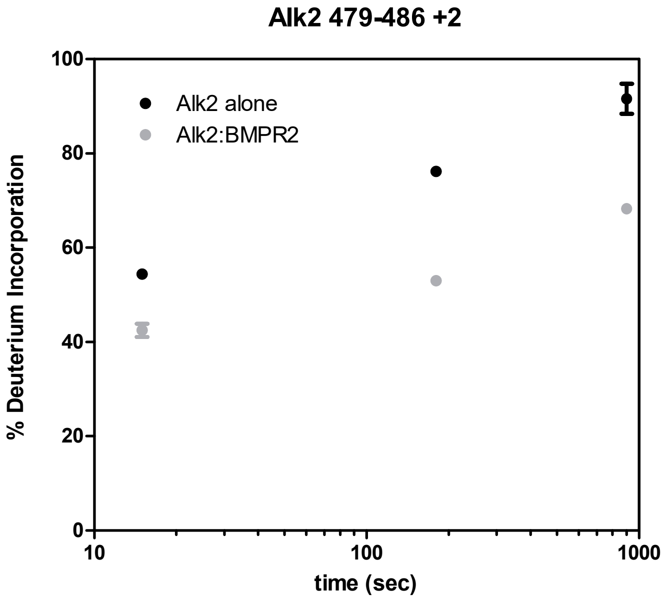

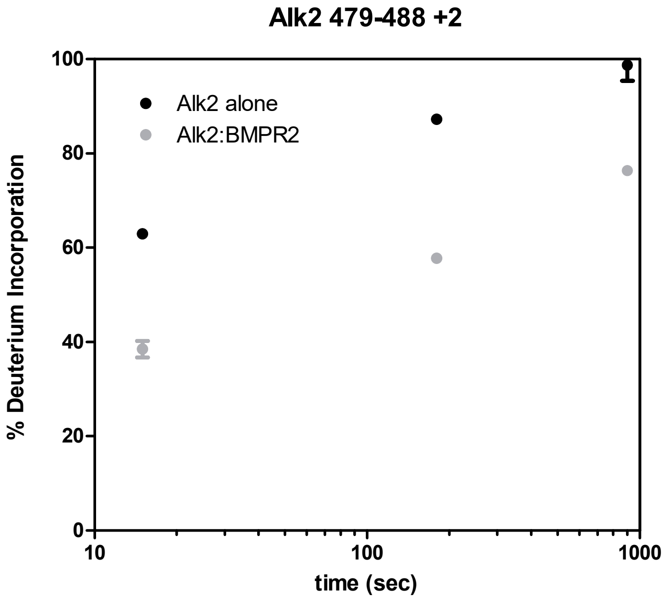

Alk2 or BMPR2 vs. Alk2:BMPR2 complex - HDX-MS Uptake Plots

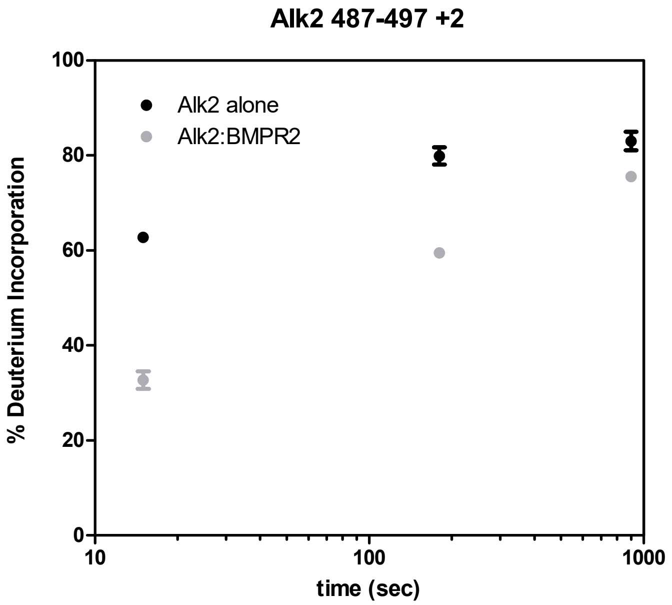

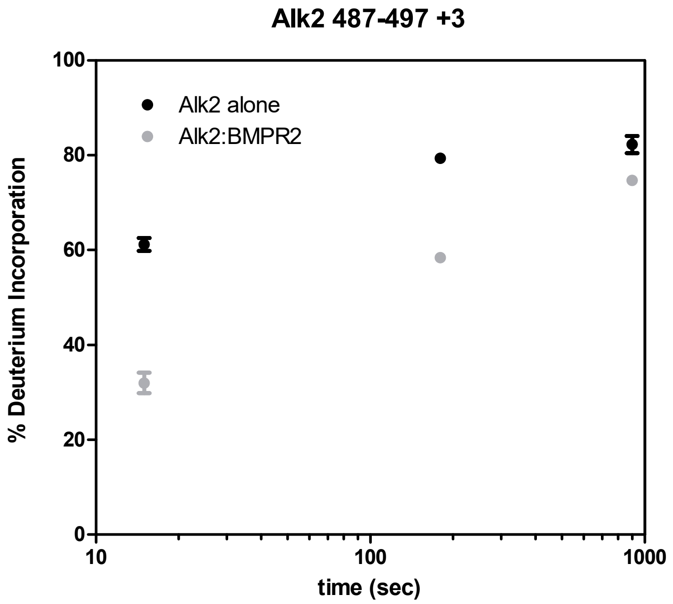

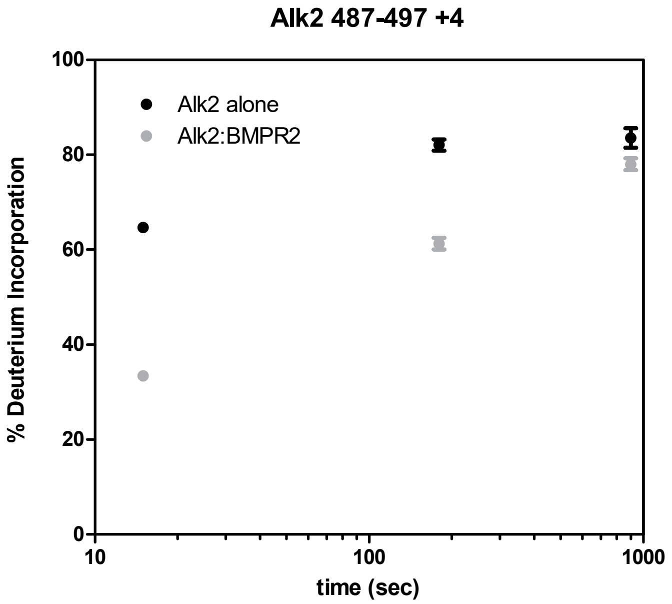

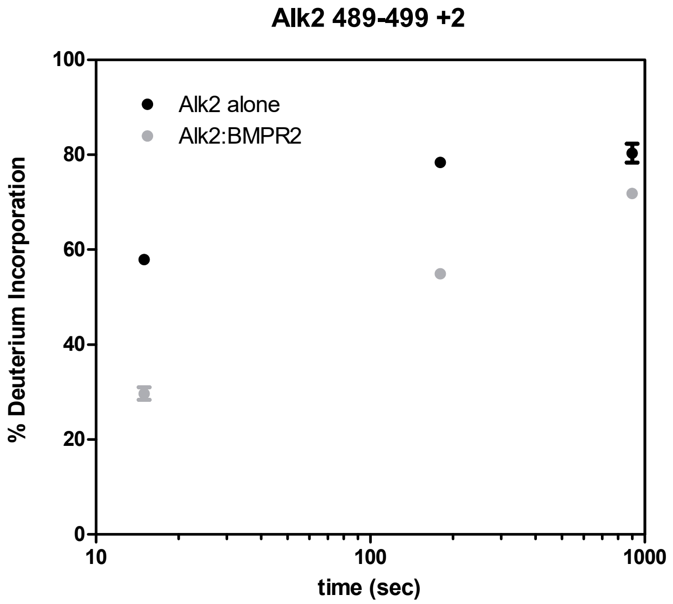

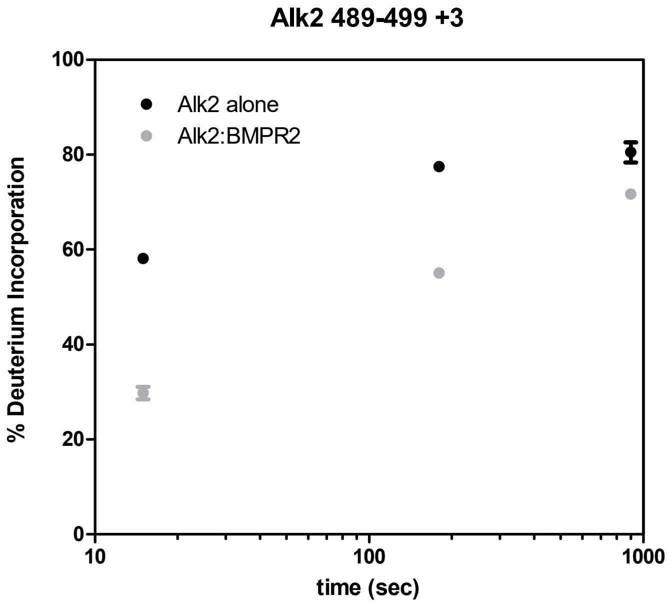

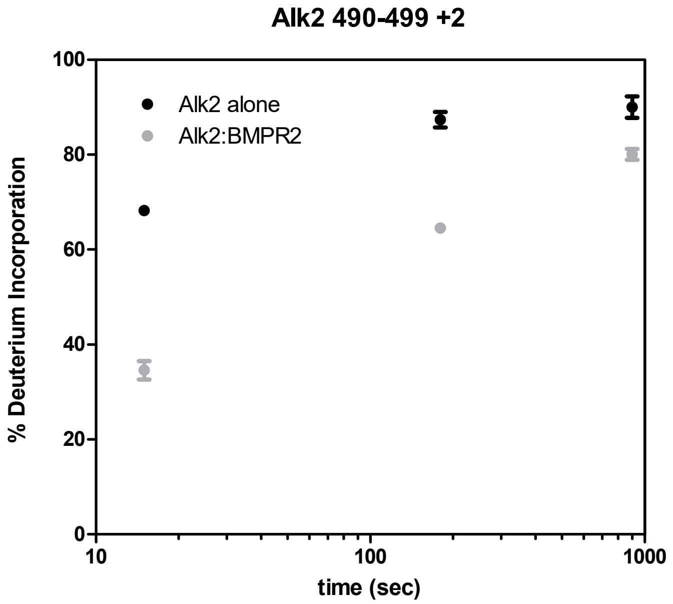

Alk2 or BMPR2 vs. Alk2:BMPR2 complex - HDX-MS Uptake Plots

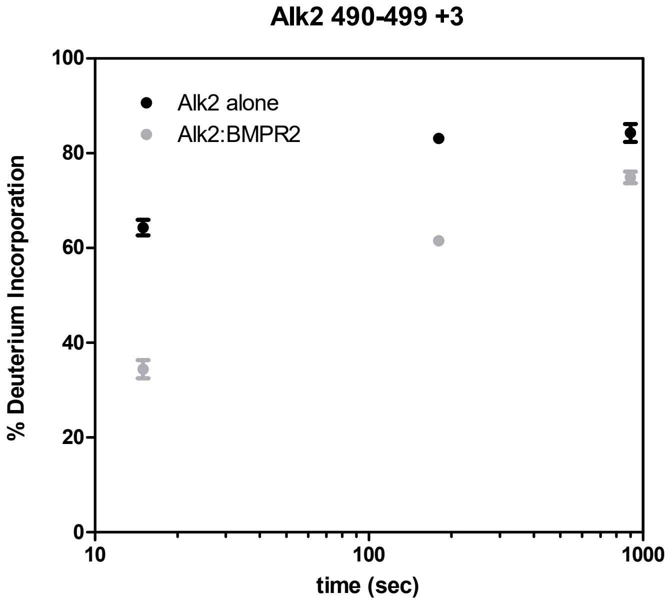

Alk2 or BMPR2 vs. Alk2:BMPR2 complex - HDX-MS Uptake Plots

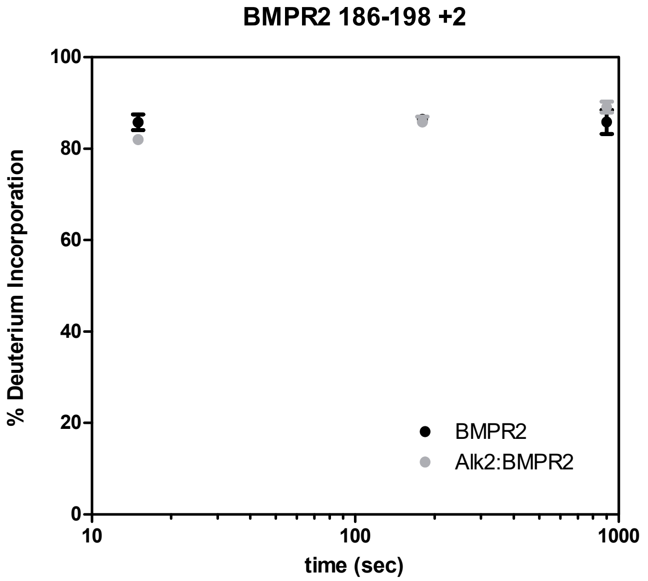

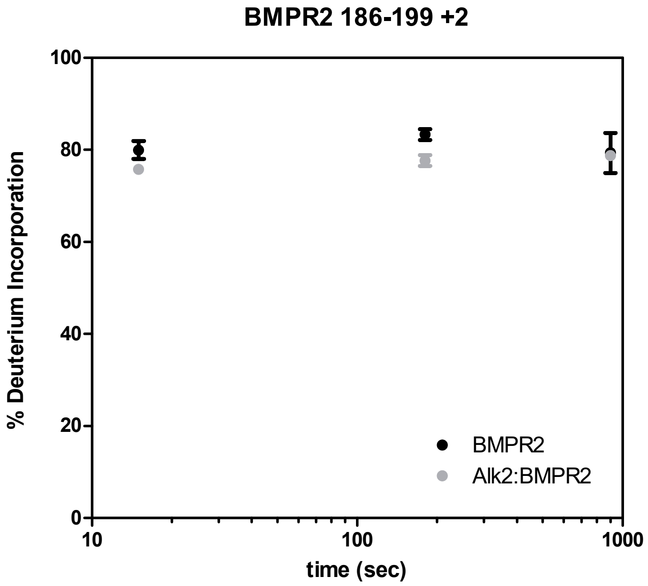

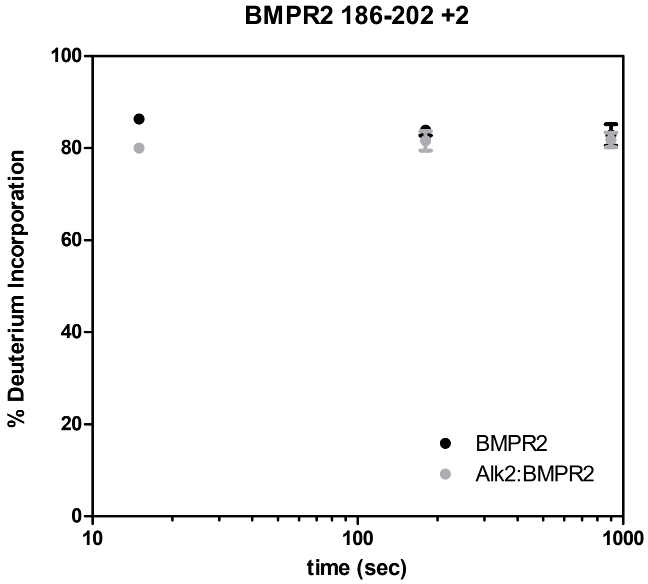

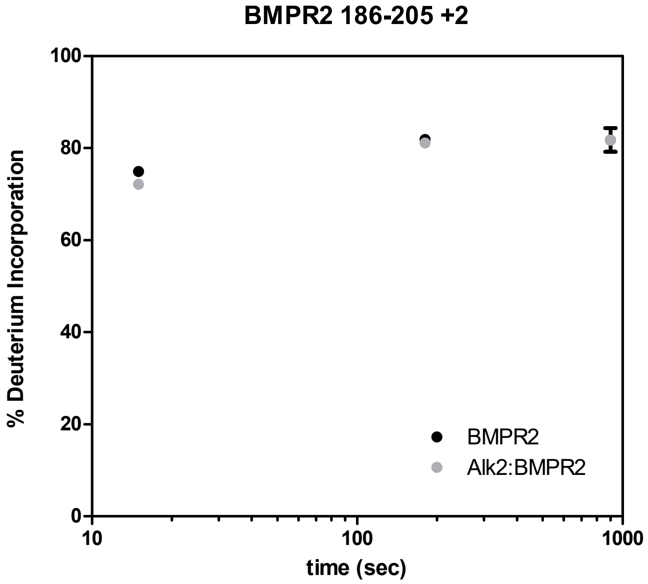

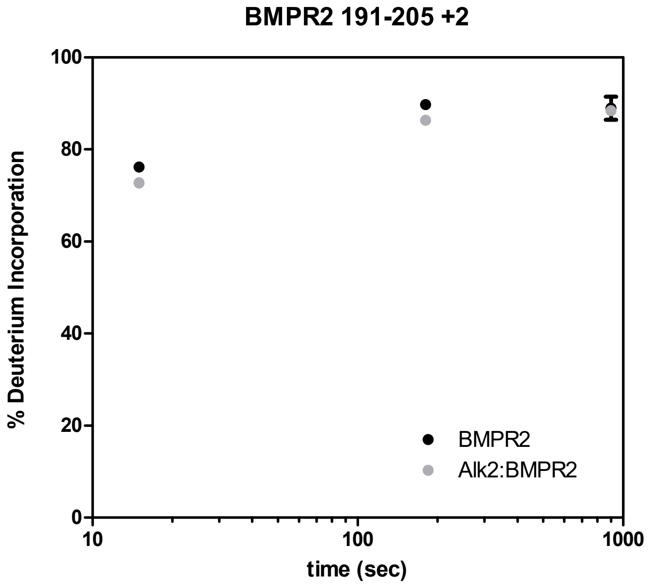

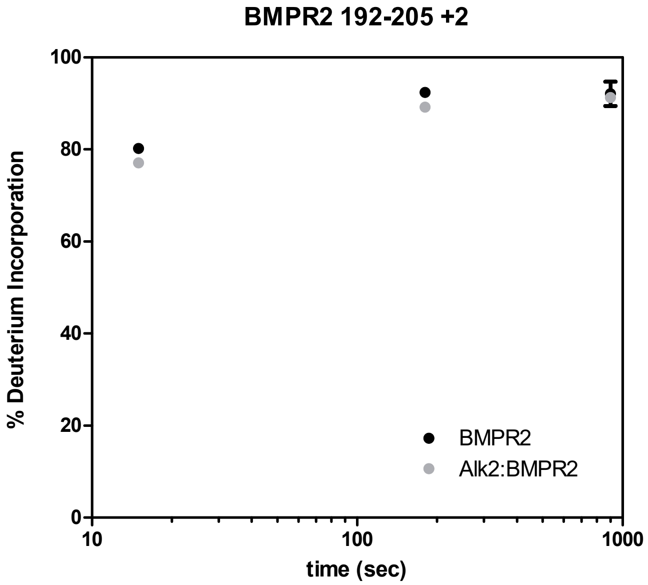

Alk2 or BMPR2 vs. Alk2:BMPR2 complex - HDX-MS Uptake Plots

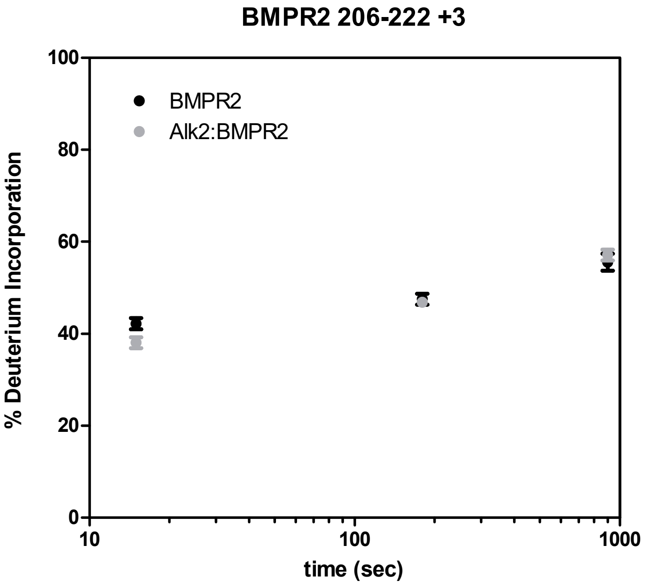

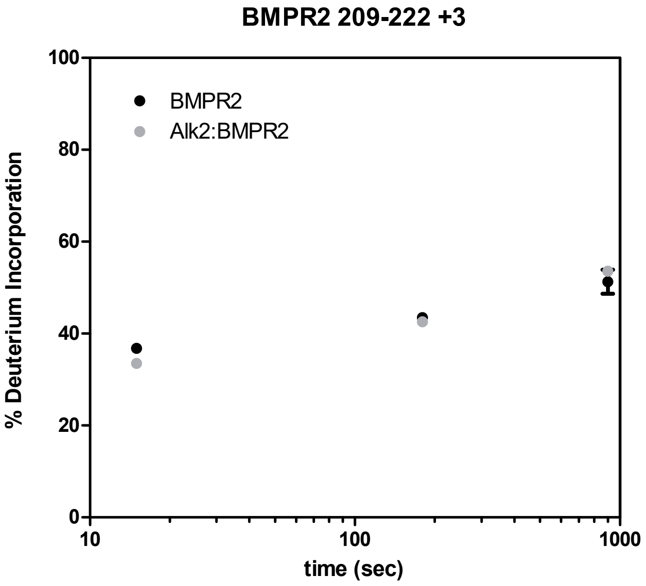

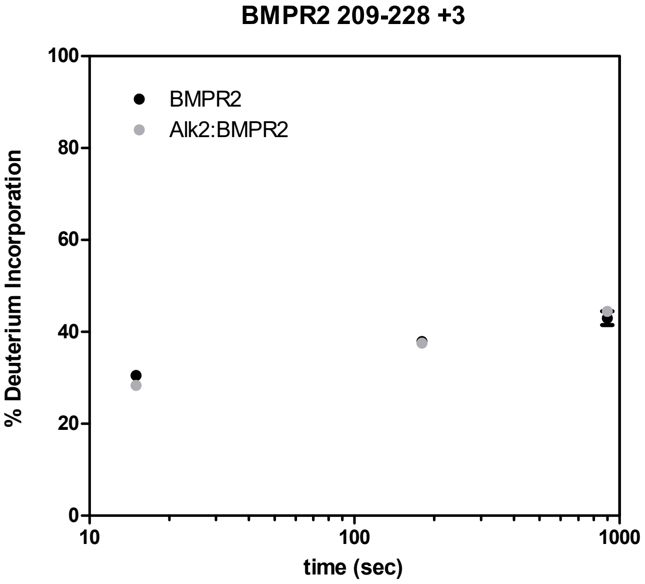

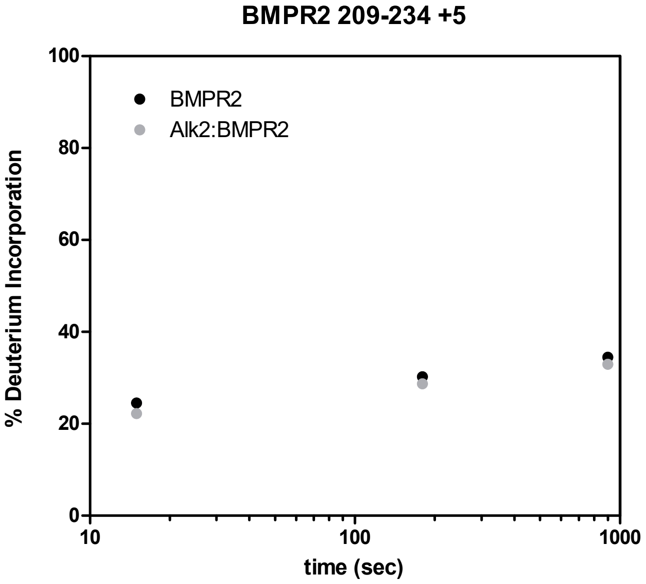

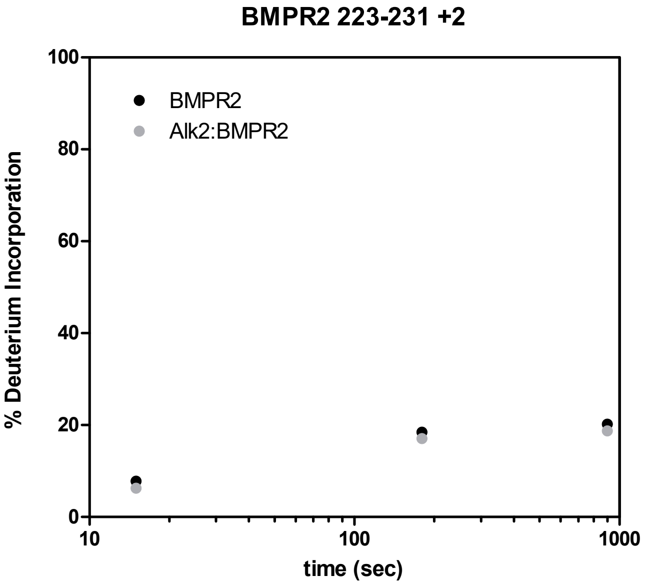

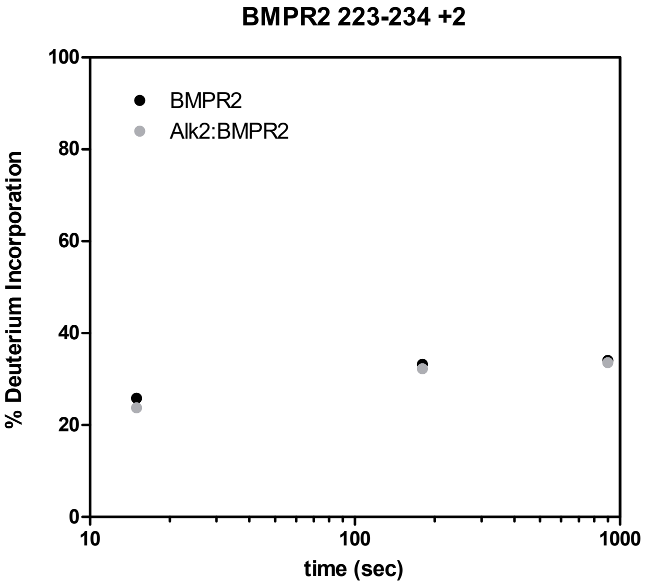

Alk2 or BMPR2 vs. Alk2:BMPR2 complex - HDX-MS Uptake Plots

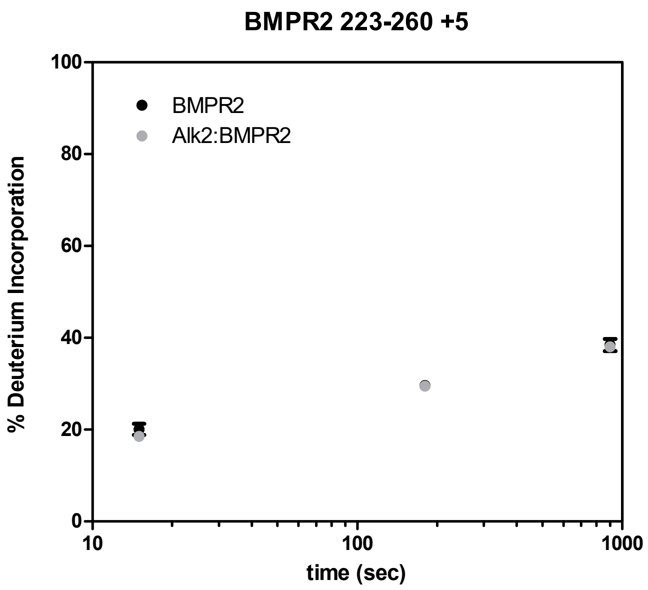

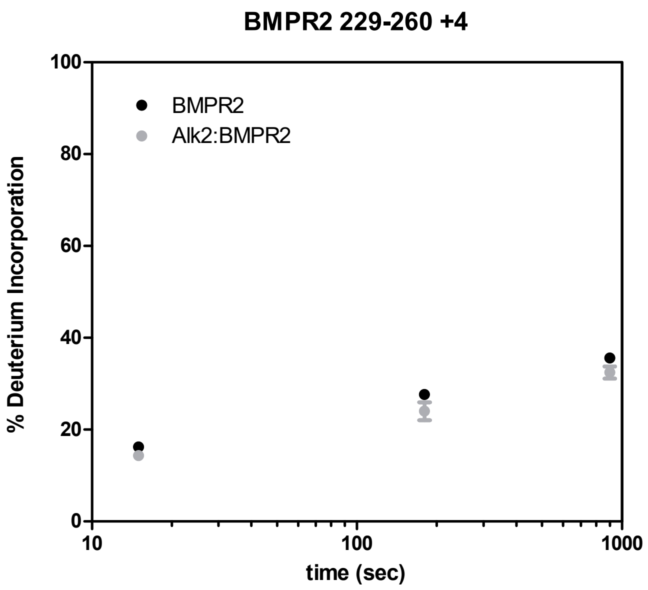

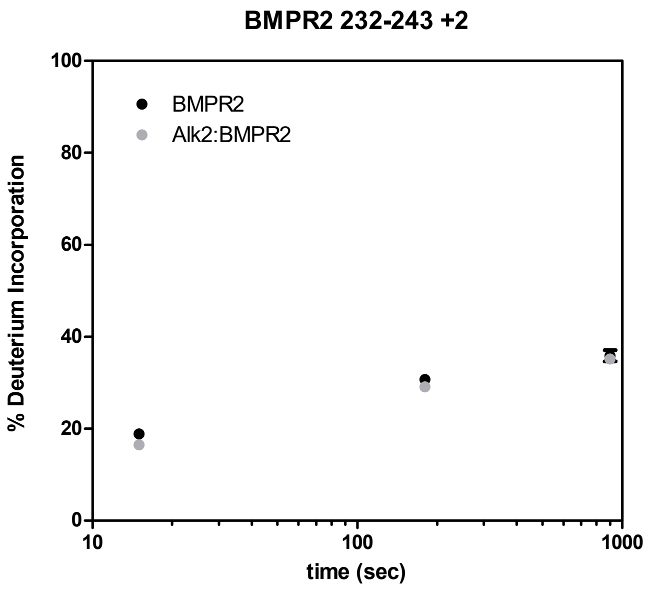

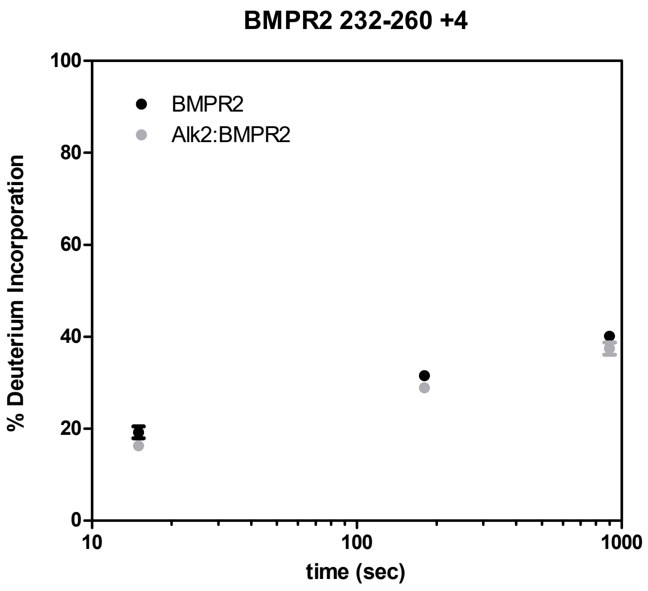

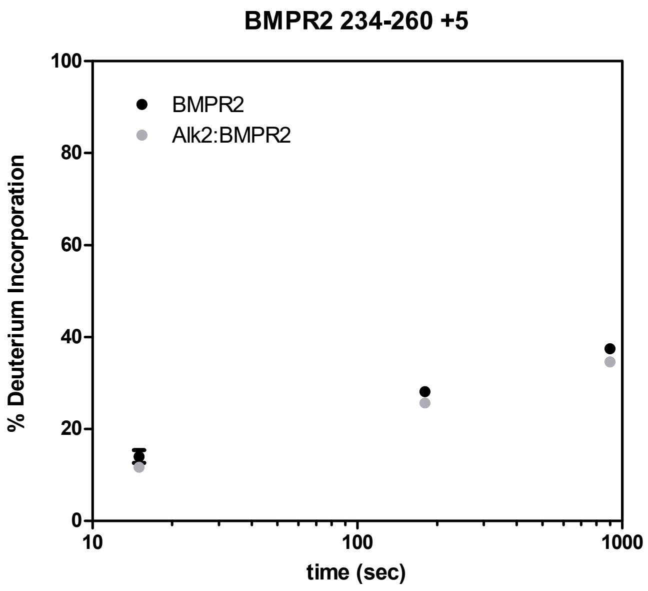

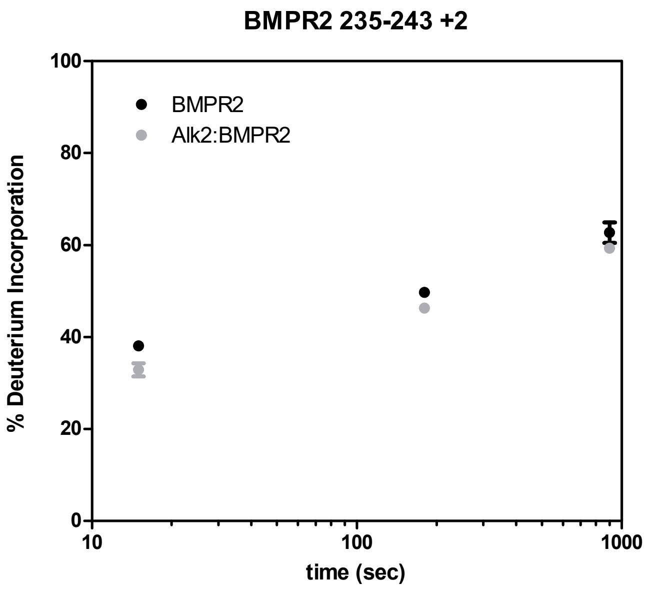

Alk2 or BMPR2 vs. Alk2:BMPR2 complex - HDX-MS Uptake Plots

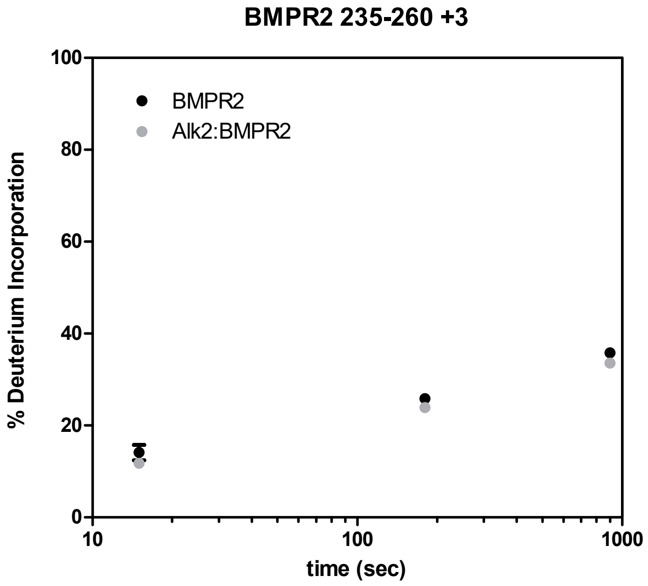

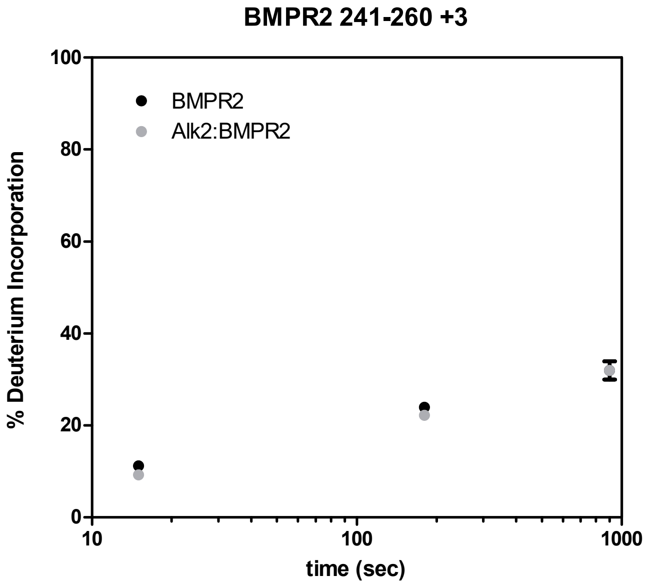

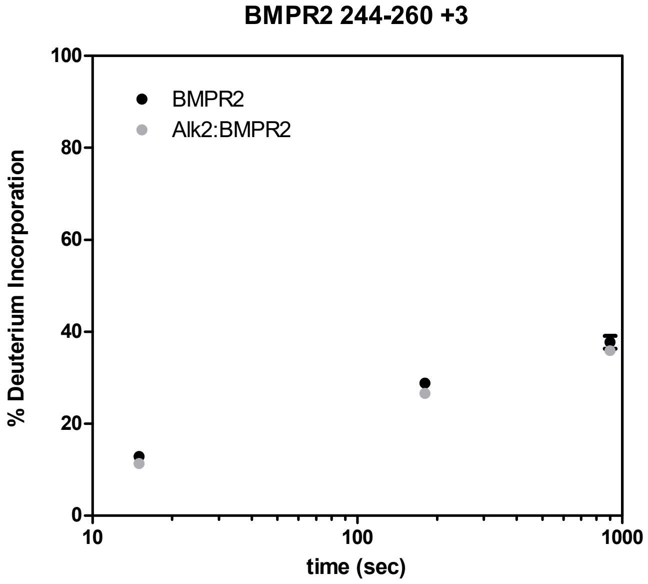

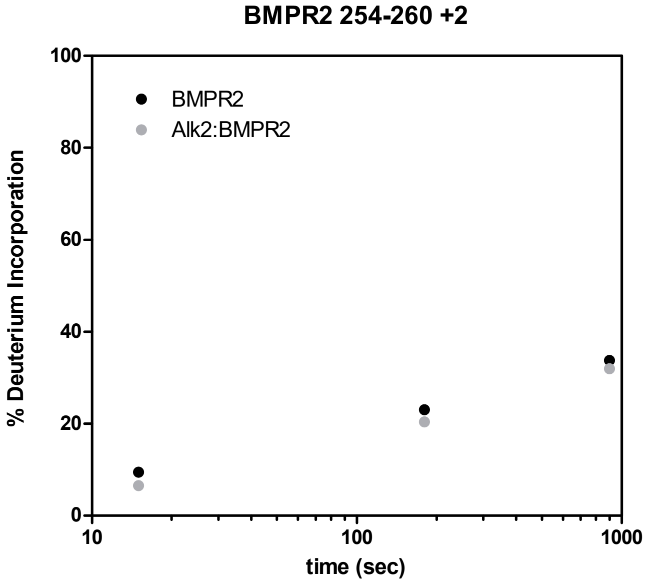

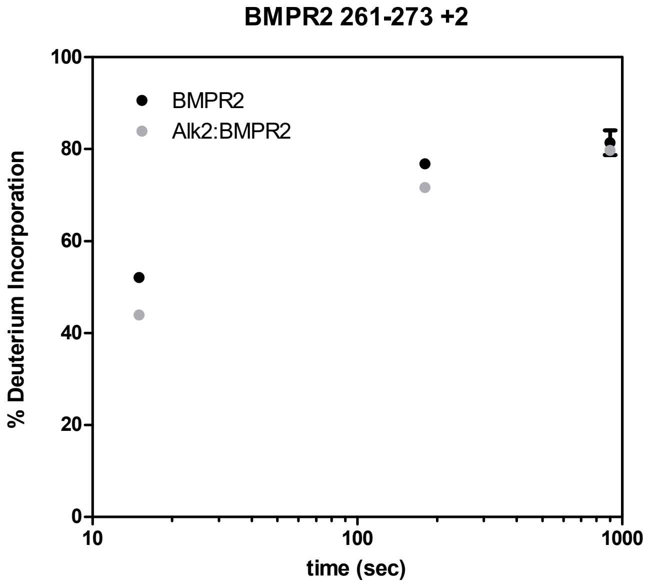

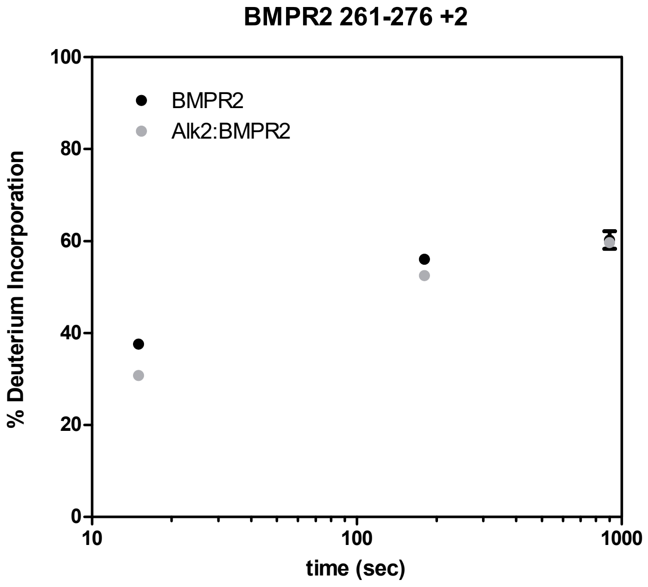

Alk2 or BMPR2 vs. Alk2:BMPR2 complex - HDX-MS Uptake Plots

BMPR2 261-276 +3

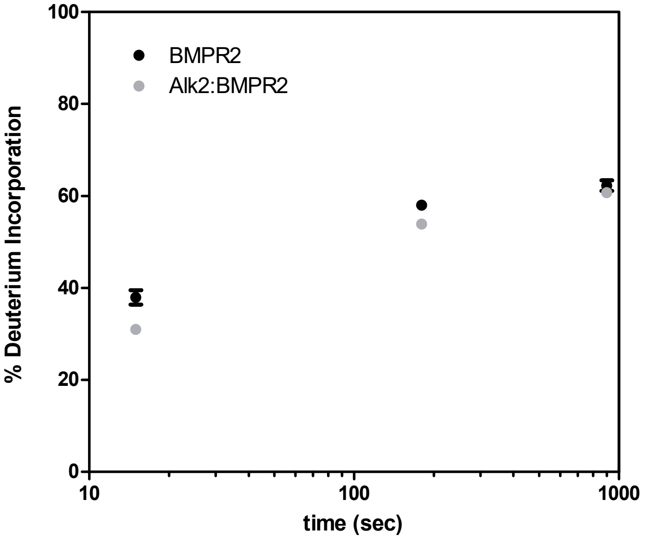

BMPR2 261-277 +3

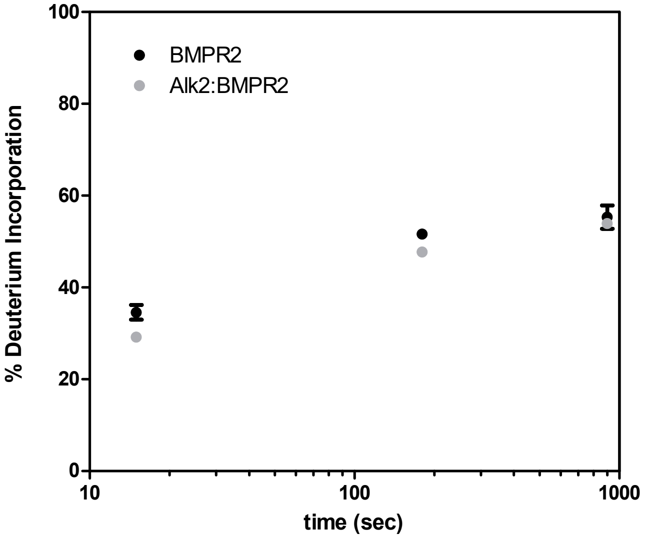

BMPR2 266-276 +2

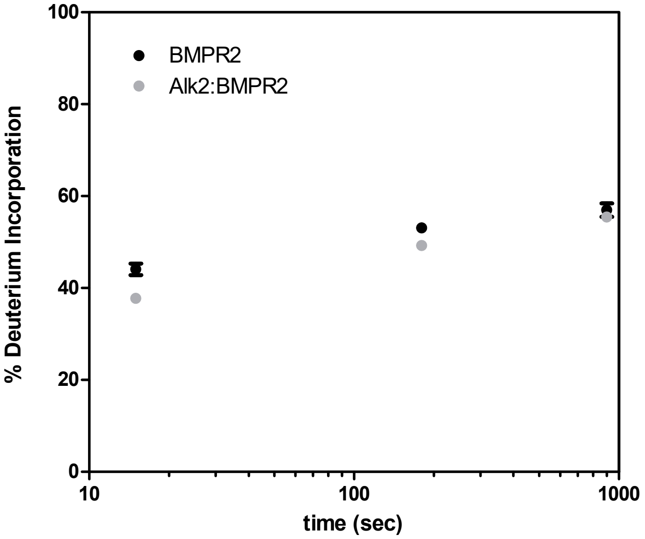

BMPR2 277-290 +2

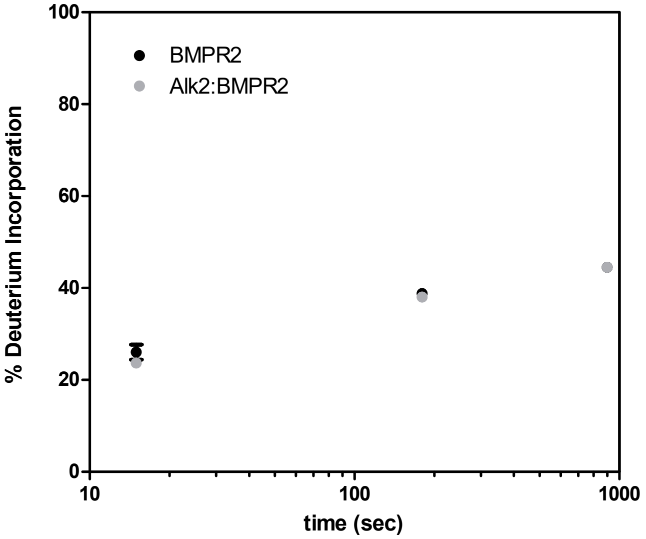

BMPR2 277-293 +2

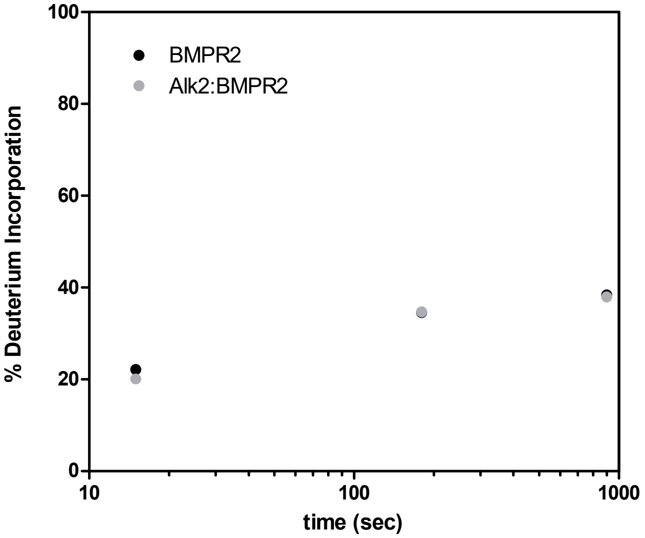

BMPR2 291-315 +3

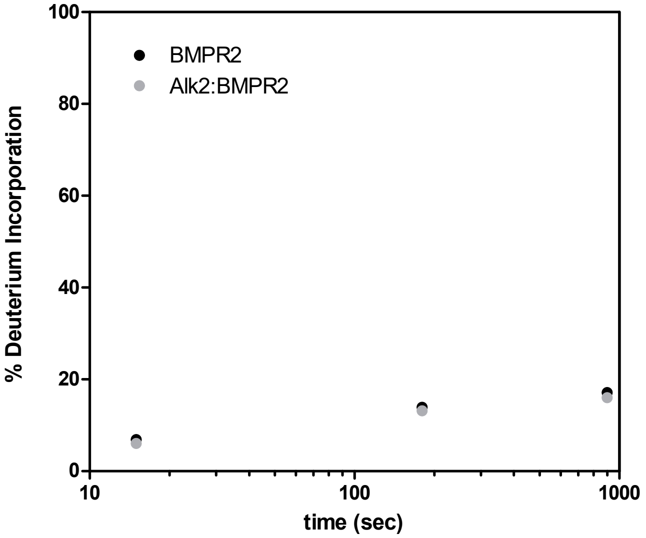

Alk2 or BMPR2 vs. Alk2:BMPR2 complex - HDX-MS Uptake Plots

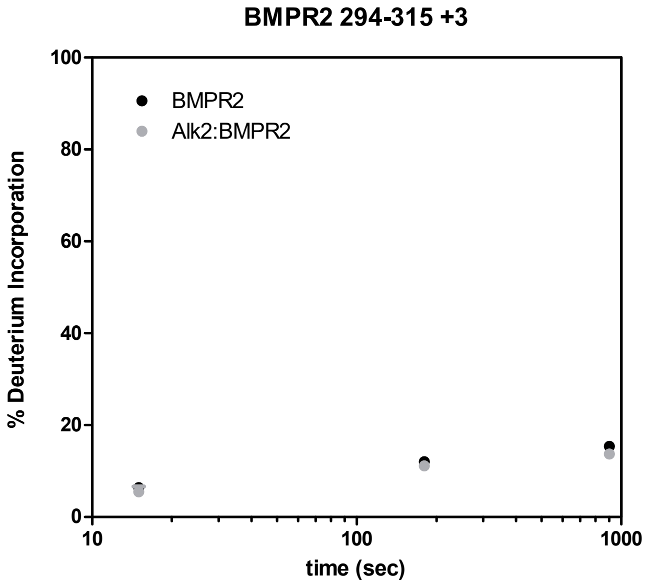

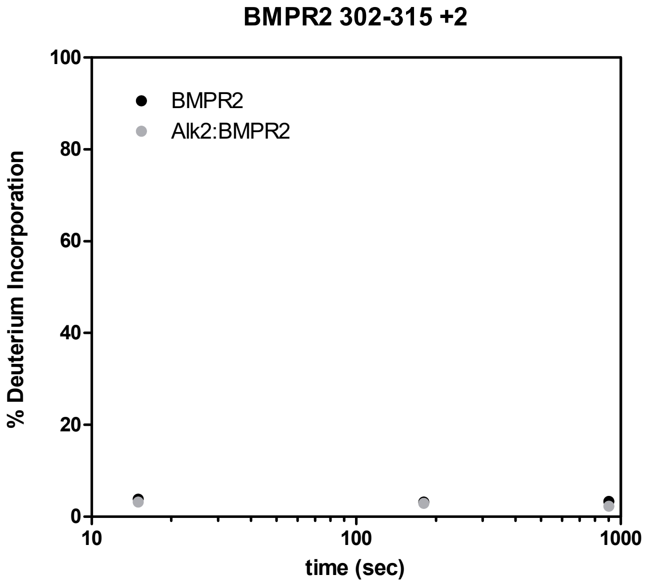

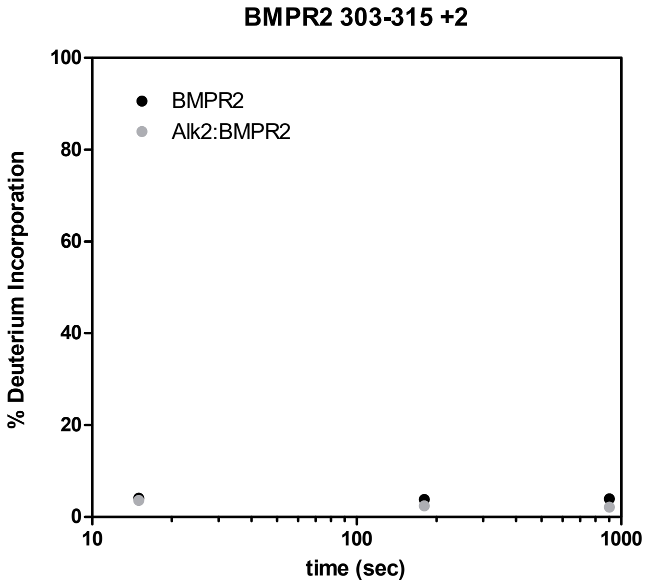

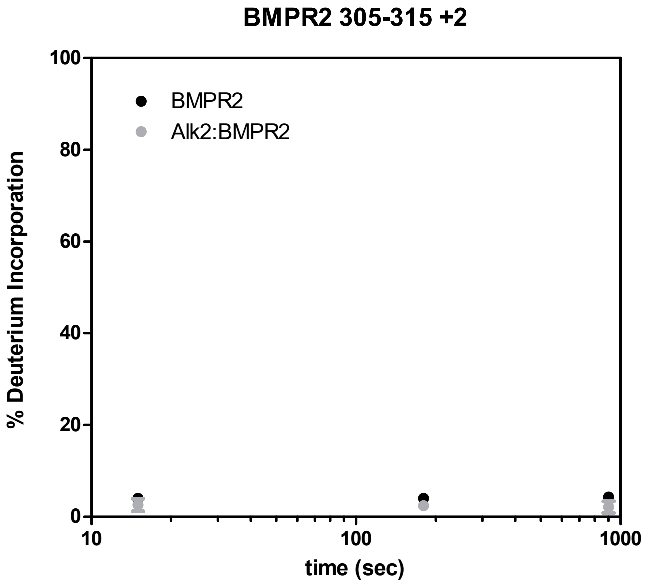

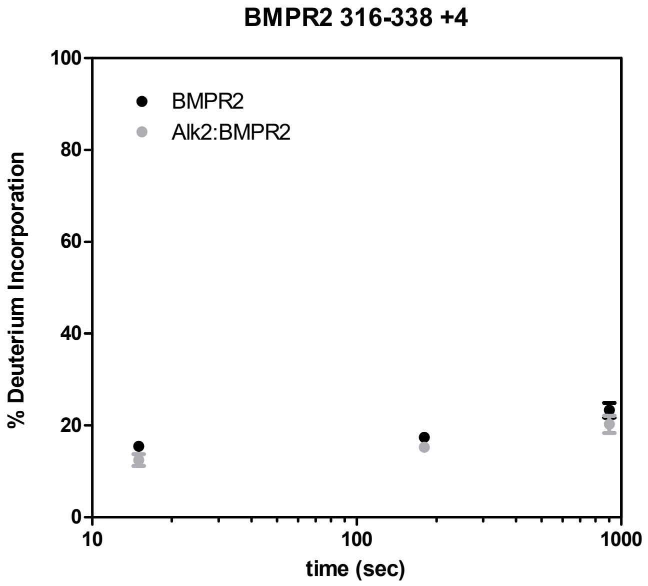

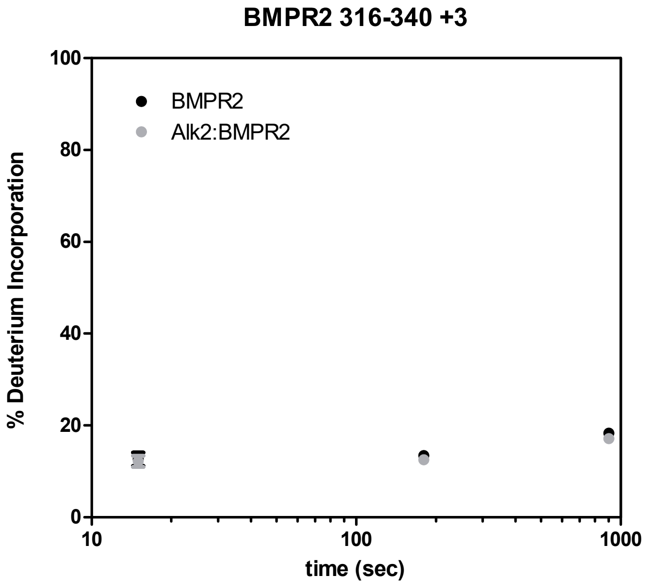

Alk2 or BMPR2 vs. Alk2:BMPR2 complex - HDX-MS Uptake Plots

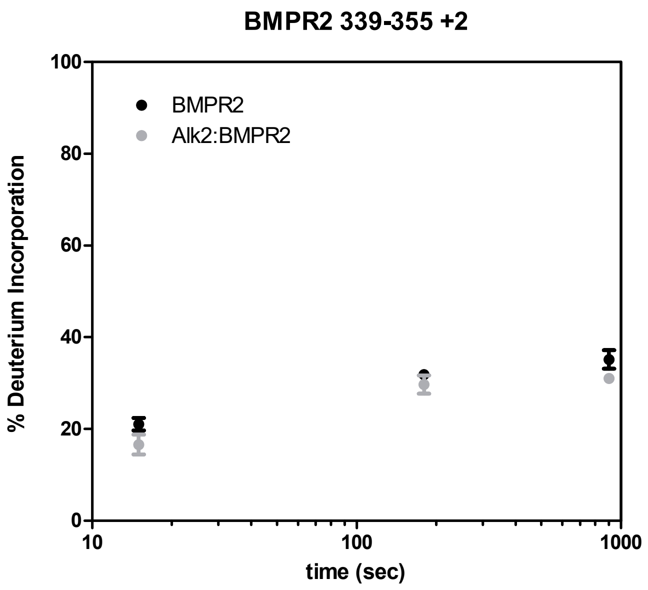

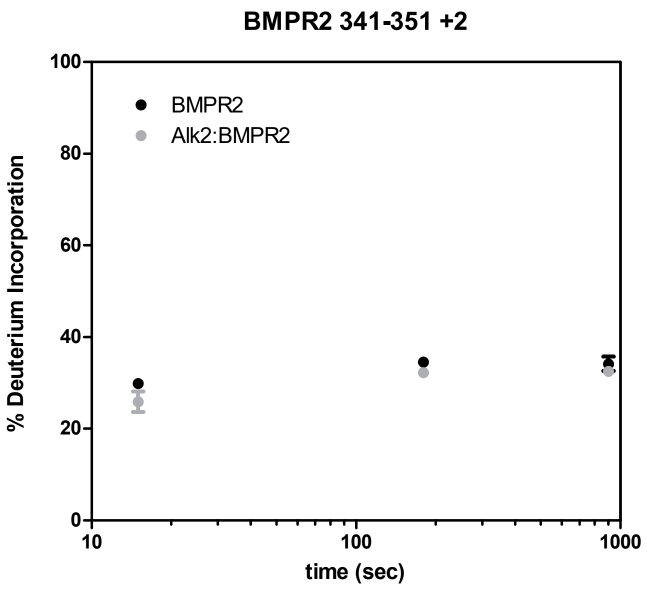

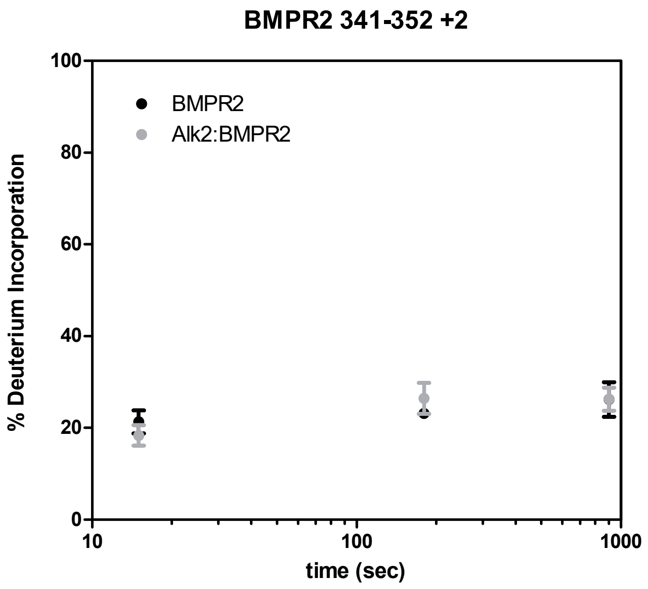

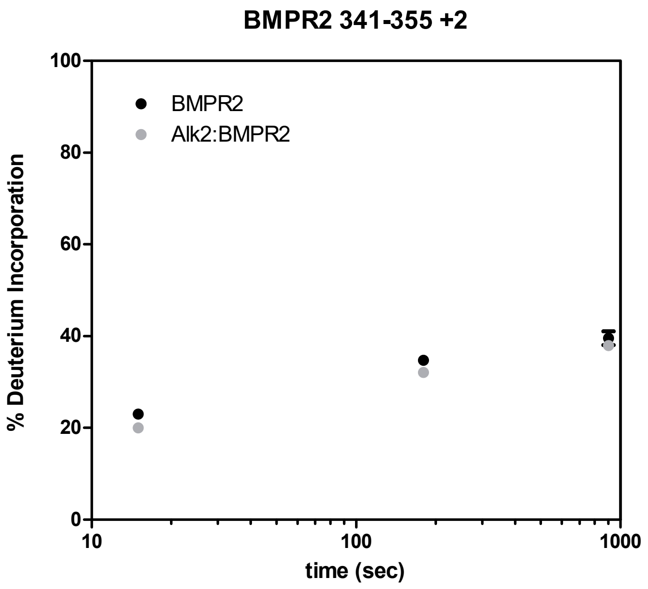

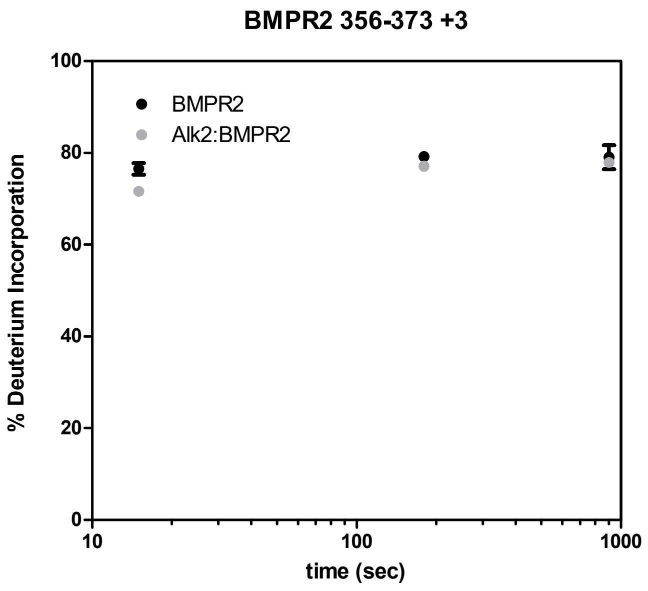

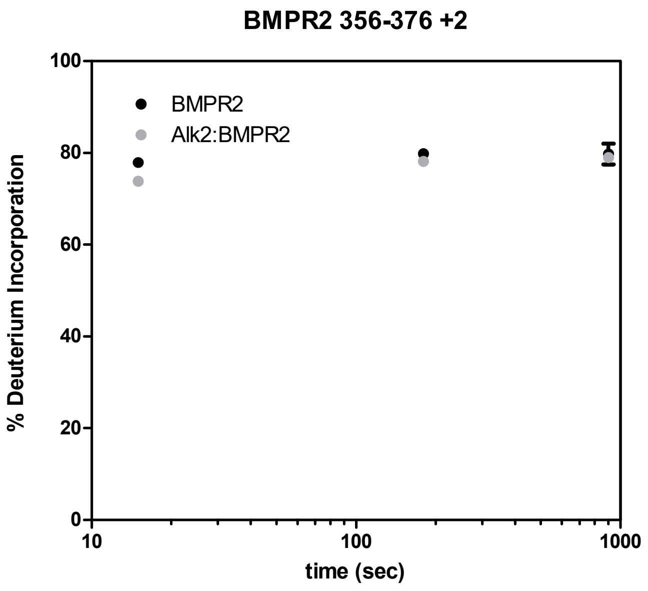

Alk2 or BMPR2 vs. Alk2:BMPR2 complex - HDX-MS Uptake Plots

BMPR2 374-388 +2

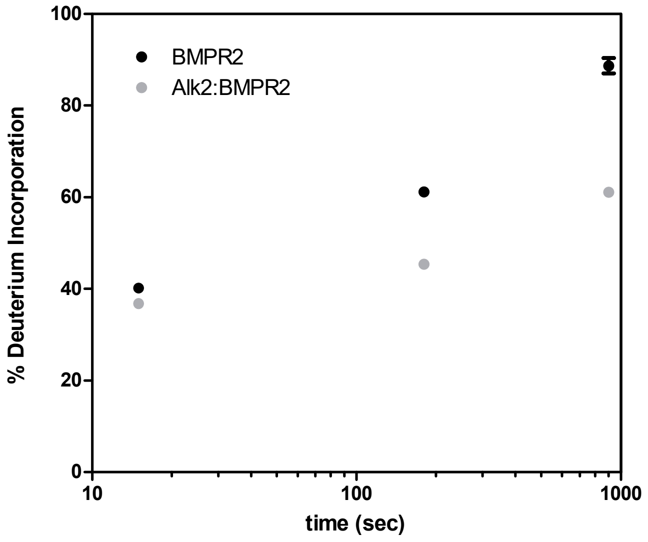

BMPR2 377-388 +2

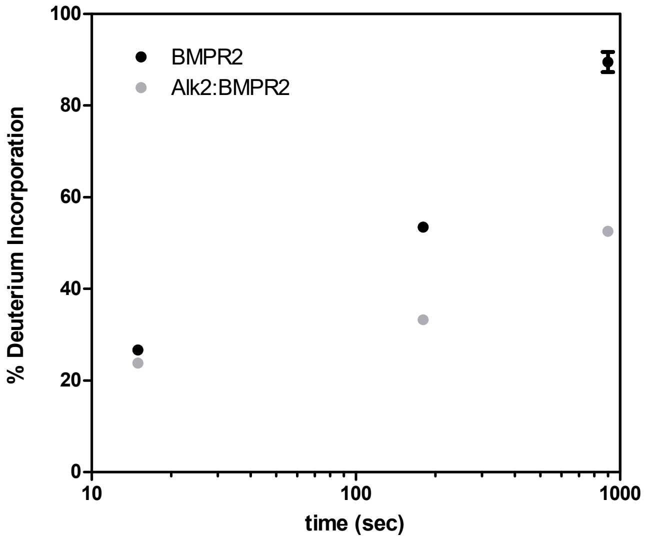

BMPR2 377-390 +2

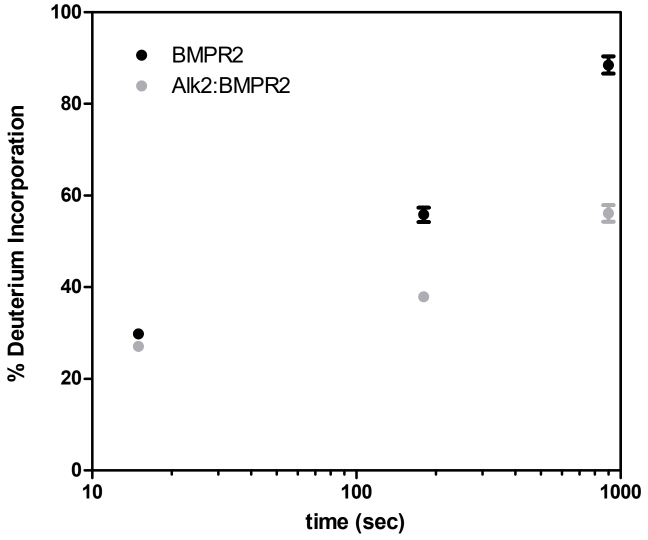

BMPR2 377-394 +2

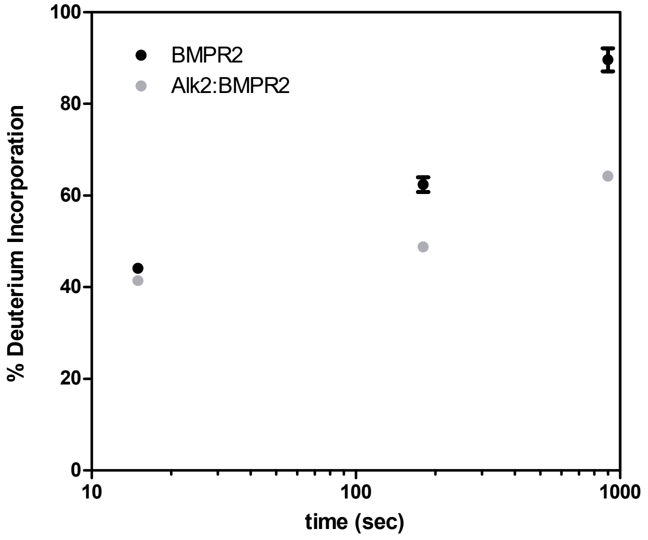

BMPR2 377-401 +3

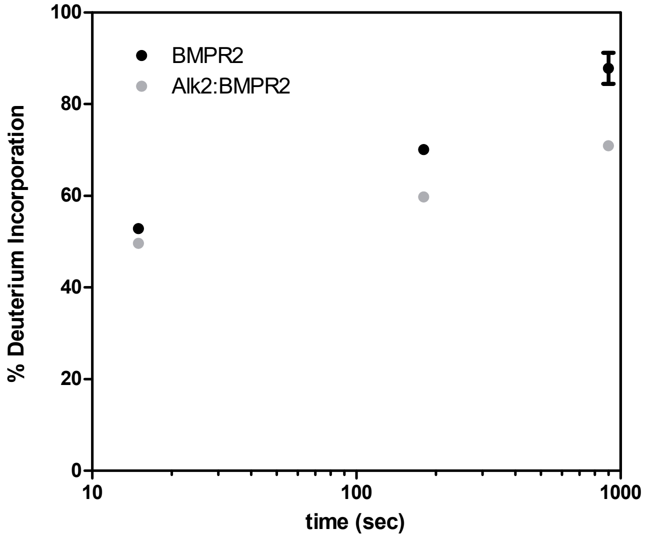

BMPR2 380-388 +2

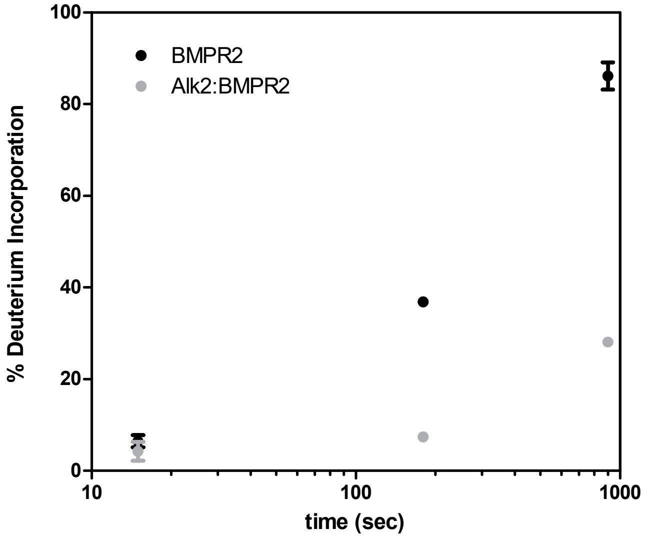

Alk2 or BMPR2 vs. Alk2:BMPR2 complex - HDX-MS Uptake Plots

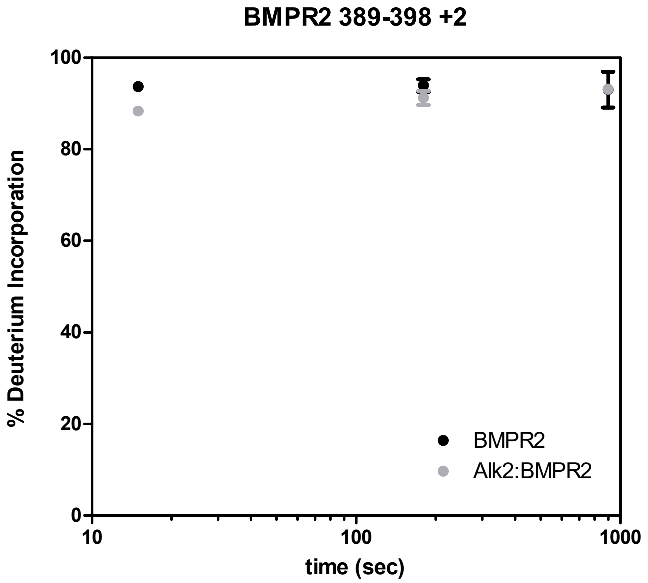

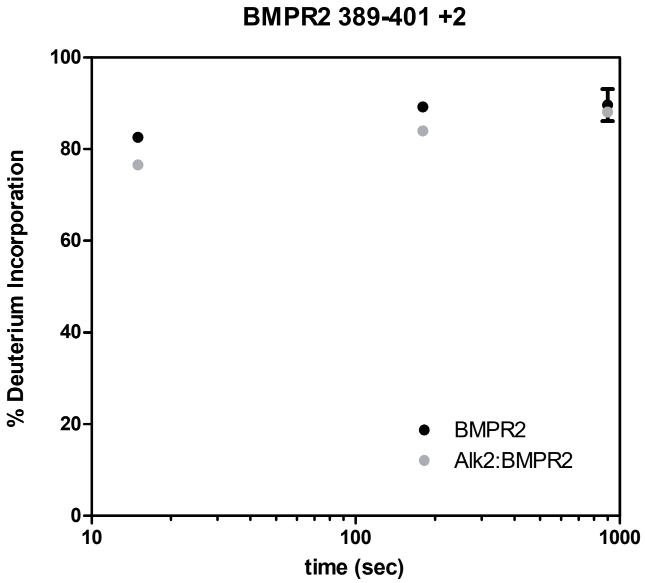

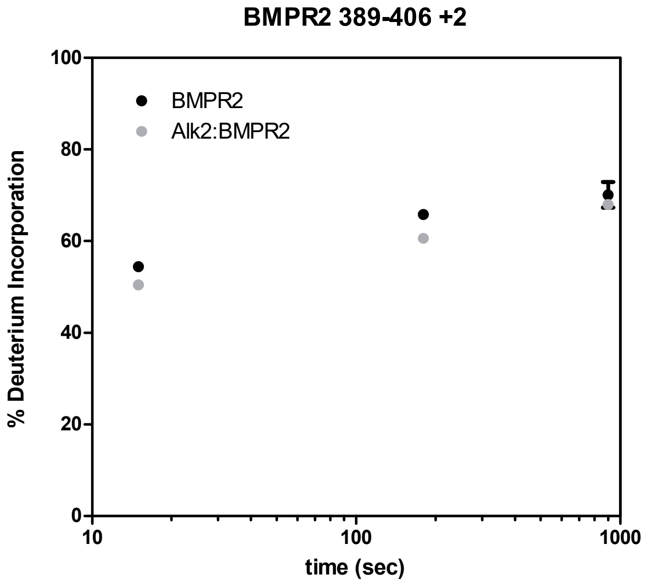

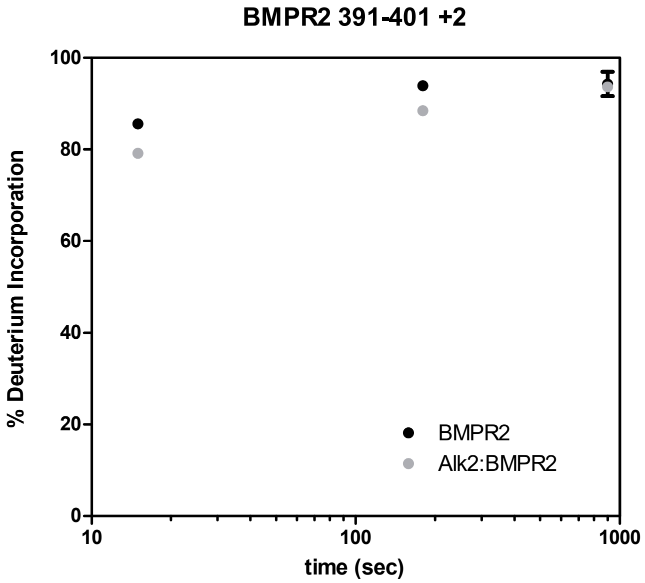

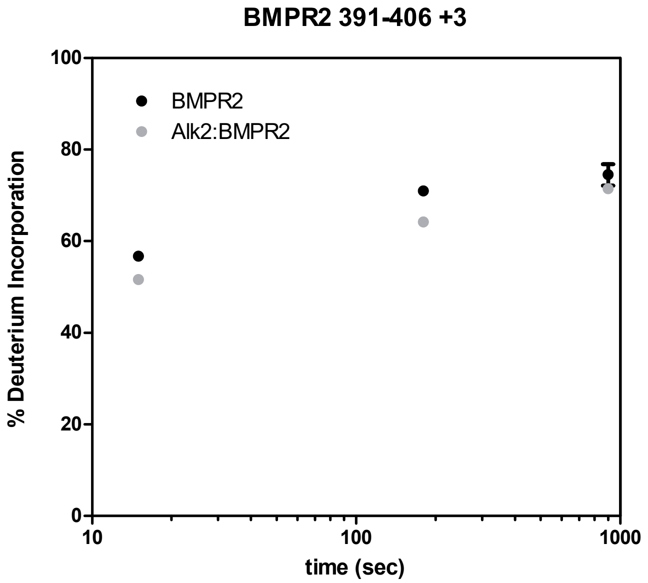

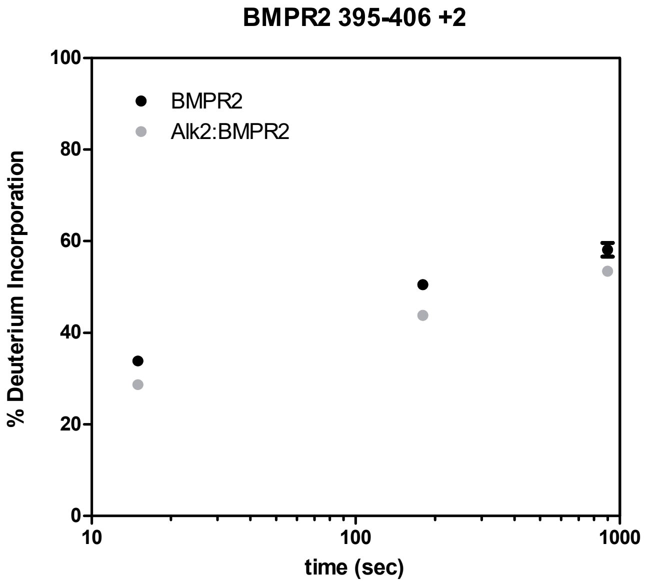

Alk2 or BMPR2 vs. Alk2:BMPR2 complex - HDX-MS Uptake Plots

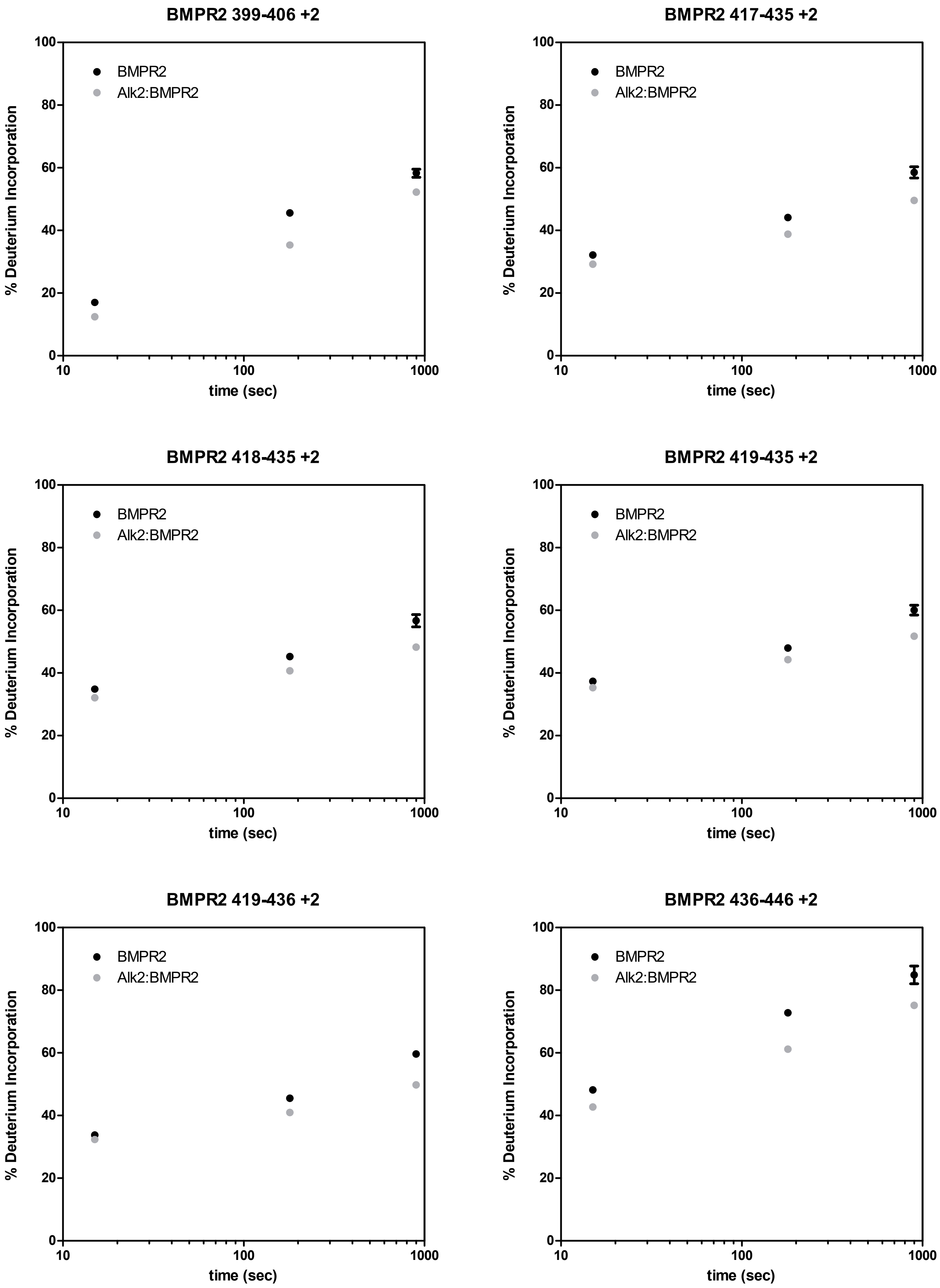

Alk2 or BMPR2 vs. Alk2:BMPR2 complex - HDX-MS Uptake Plots

BMPR2 436-449 +2

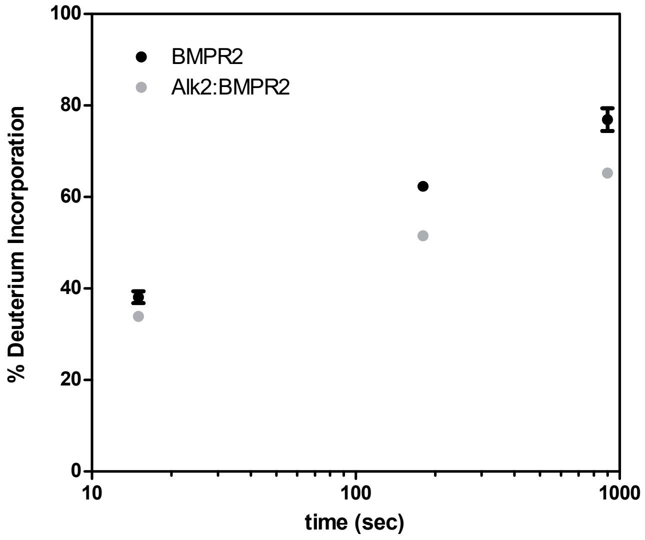

BMPR2 437-446 +2

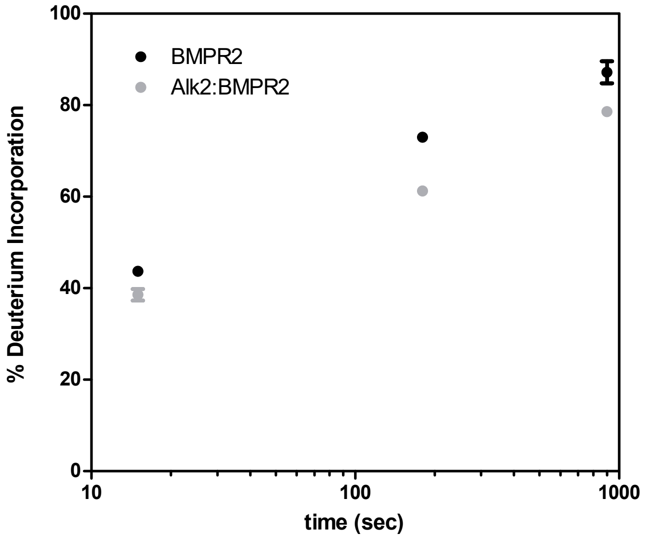

BMPR2 437-449 +2

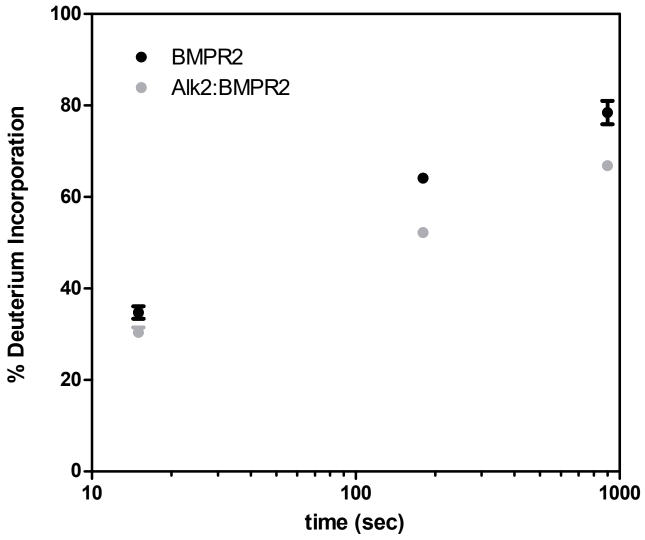

BMPR2 450-465 +3

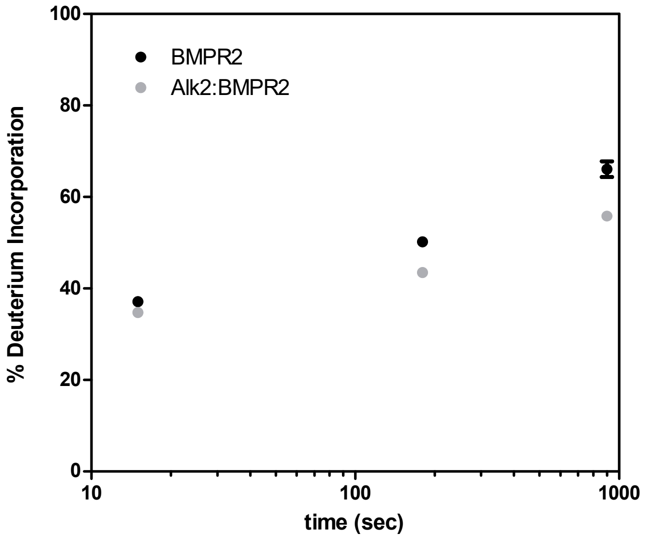

BMPR2 450-471 +4

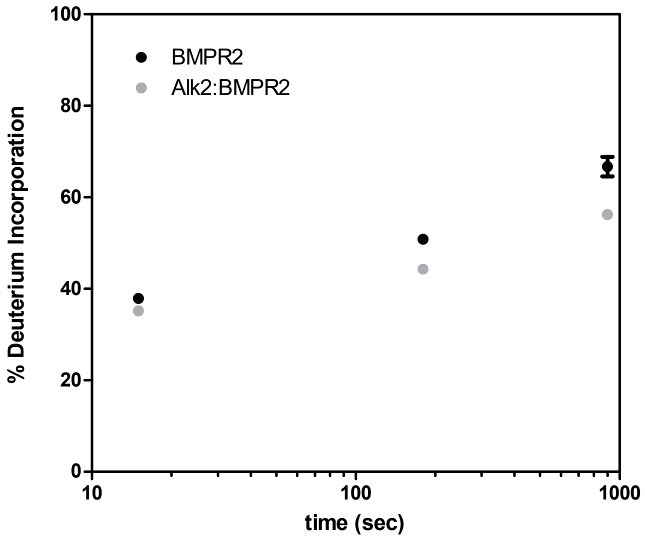

BMPR2 453-465 +3

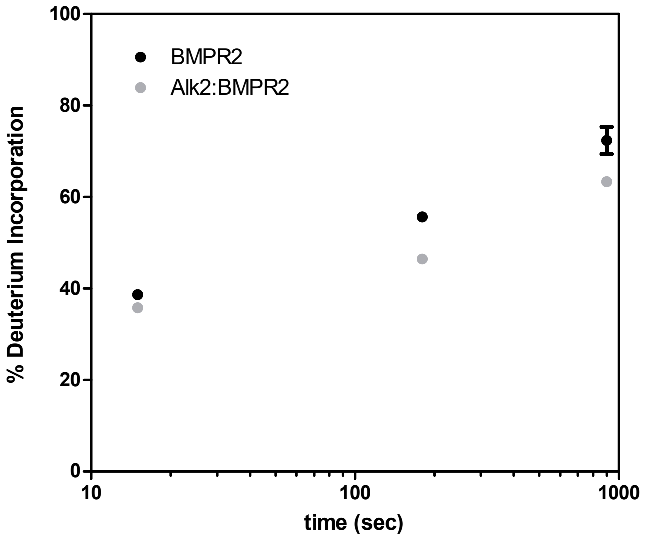

Alk2 or BMPR2 vs. Alk2:BMPR2 complex - HDX-MS Uptake Plots

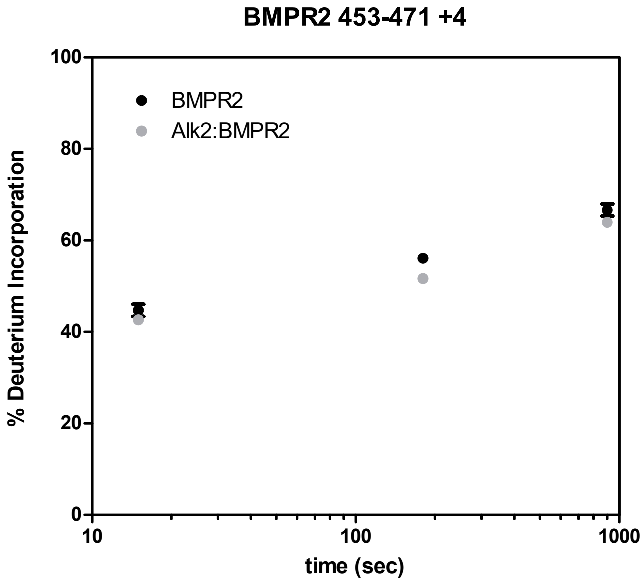

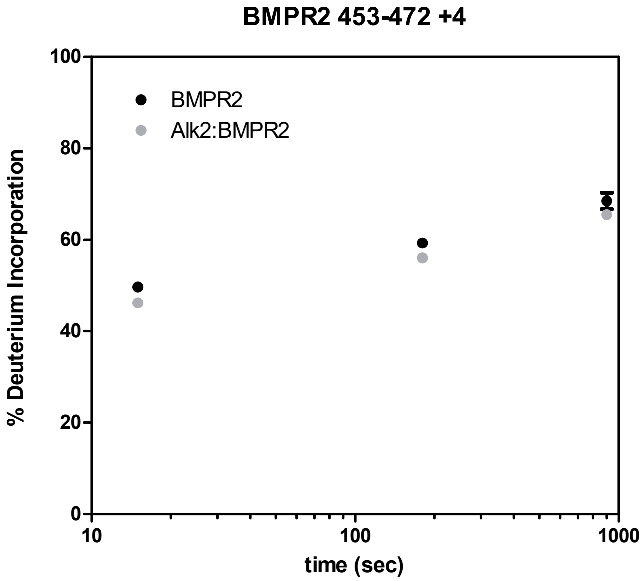

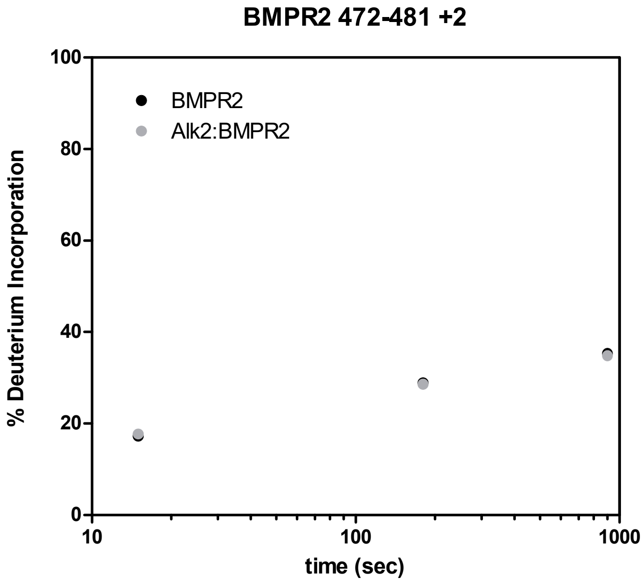

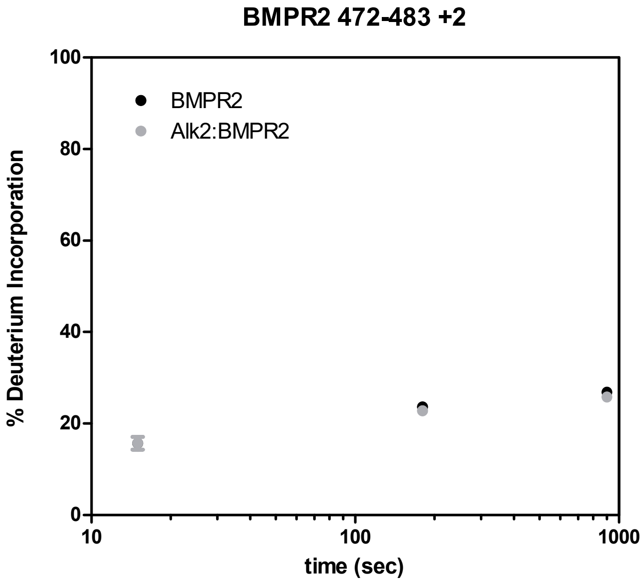

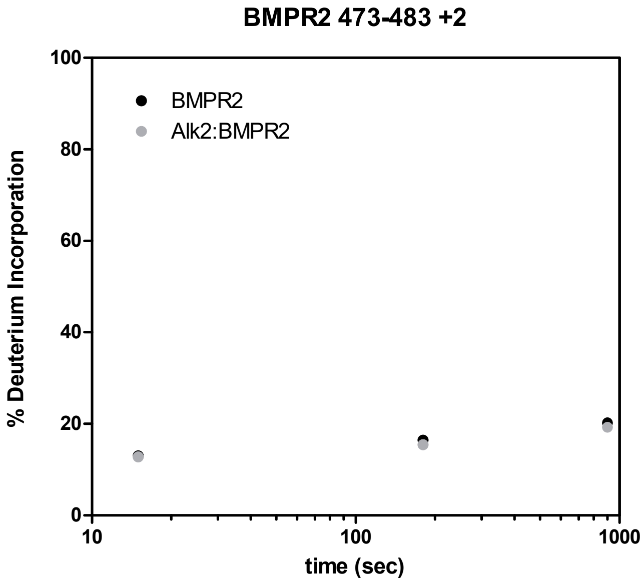

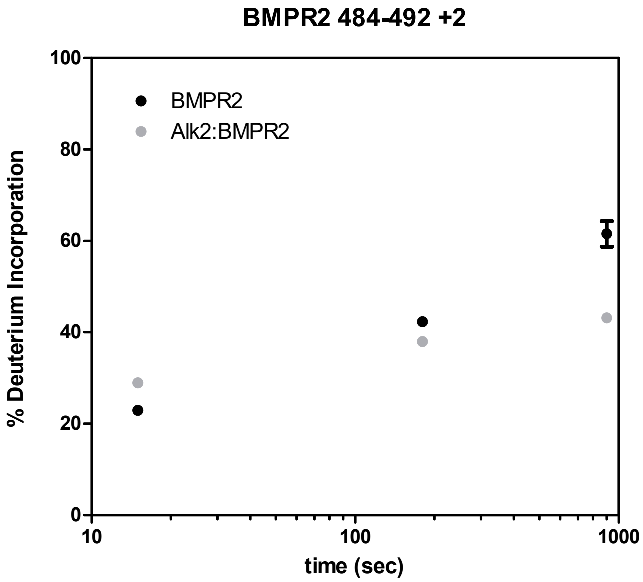

Alk2 or BMPR2 vs. Alk2:BMPR2 complex - HDX-MS Uptake Plots

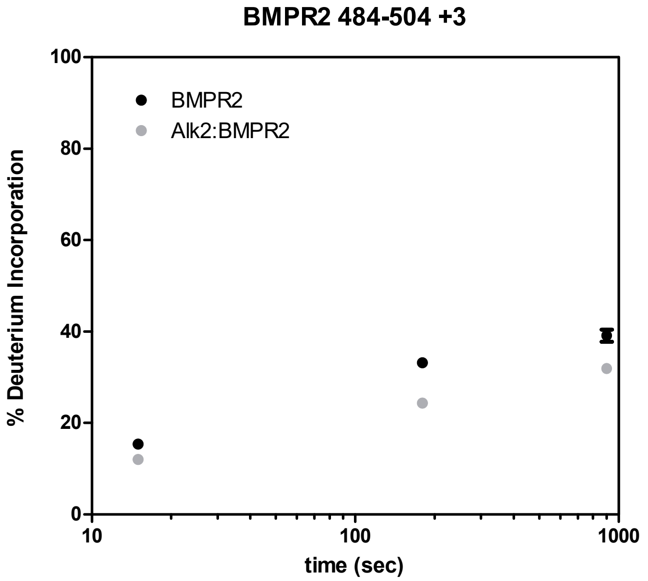

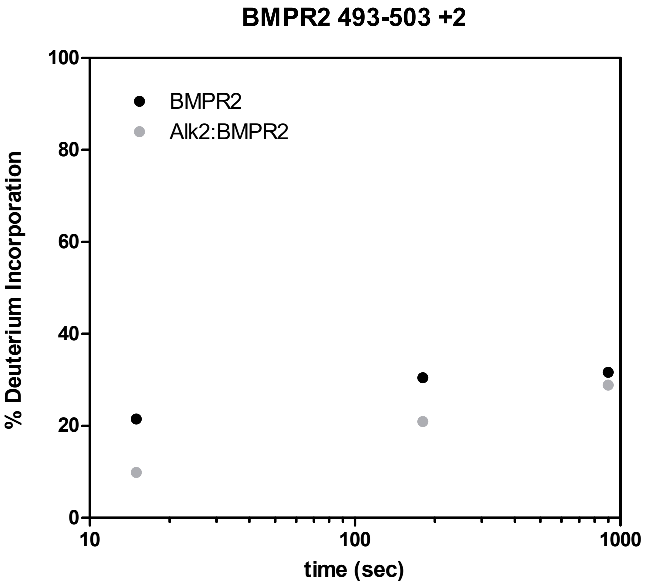

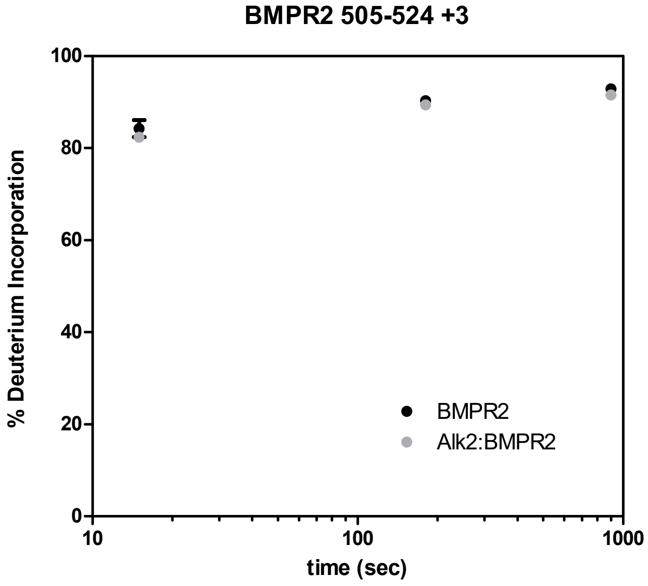

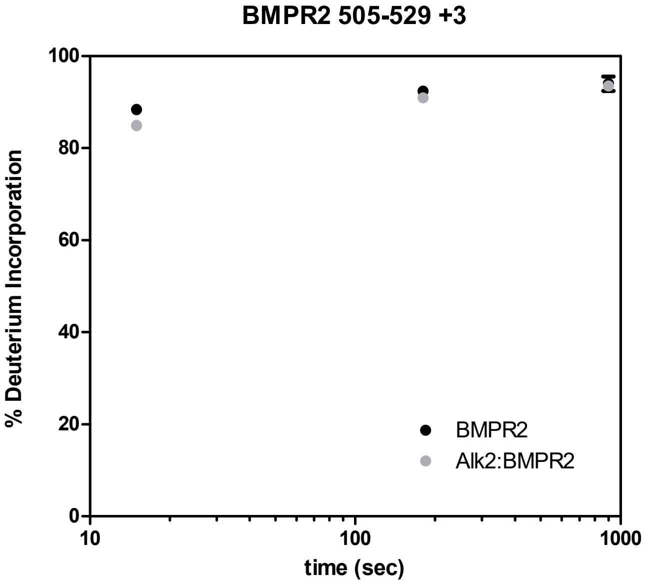

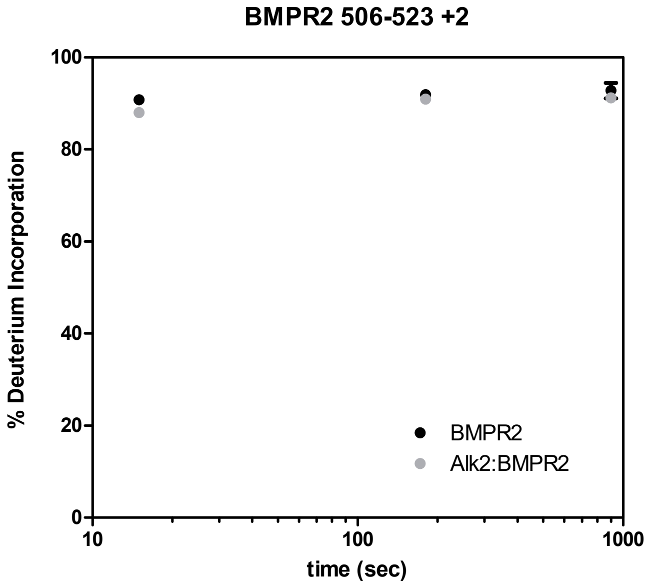

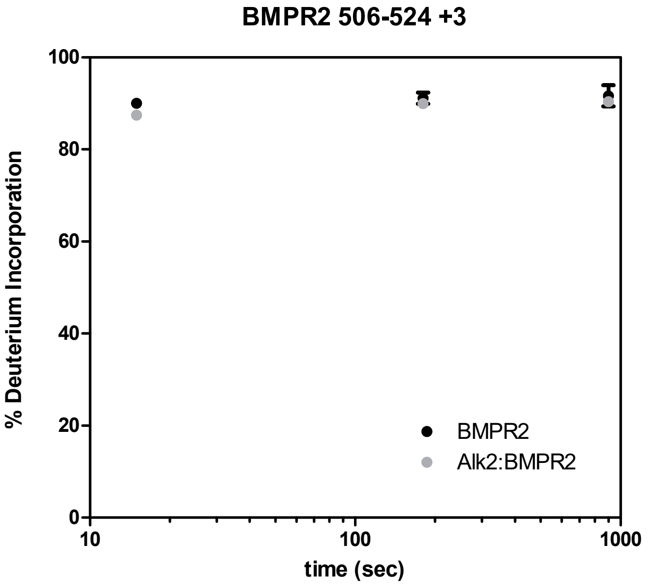

Alk2 or BMPR2 vs. Alk2:BMPR2 complex - HDX-MS Uptake Plots

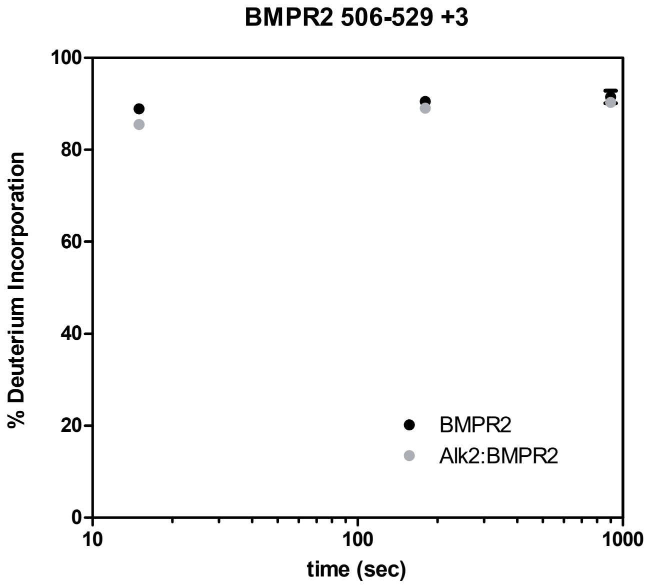

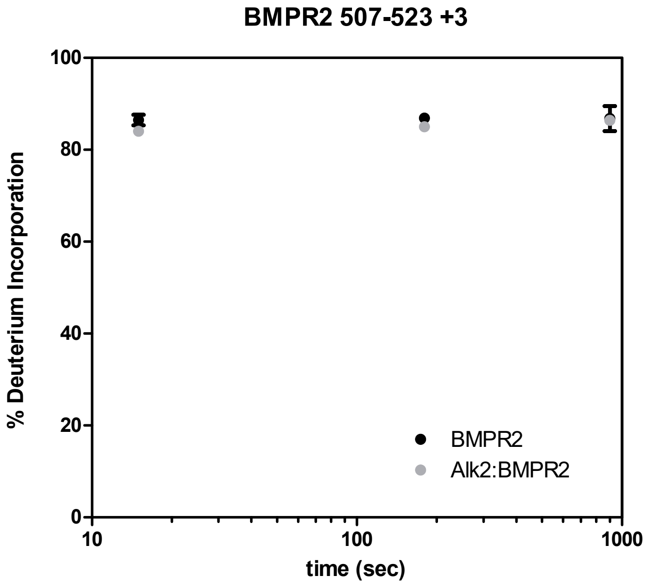

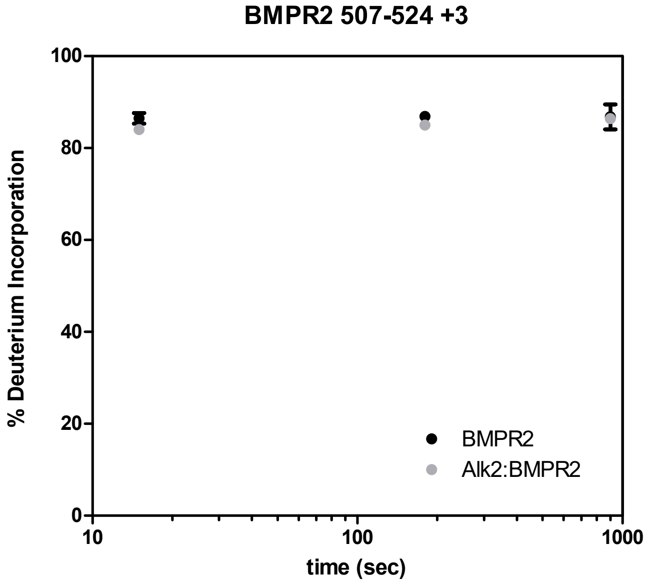

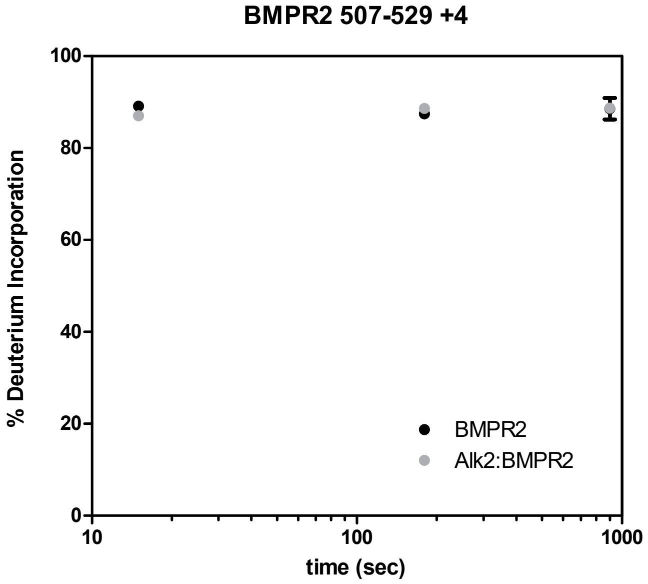

Alk2 vs. GS-Alk2 - HDX-MS Uptake Plots

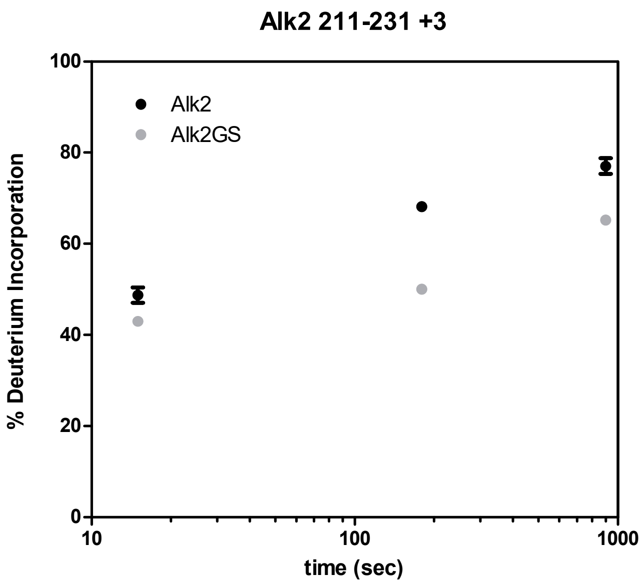

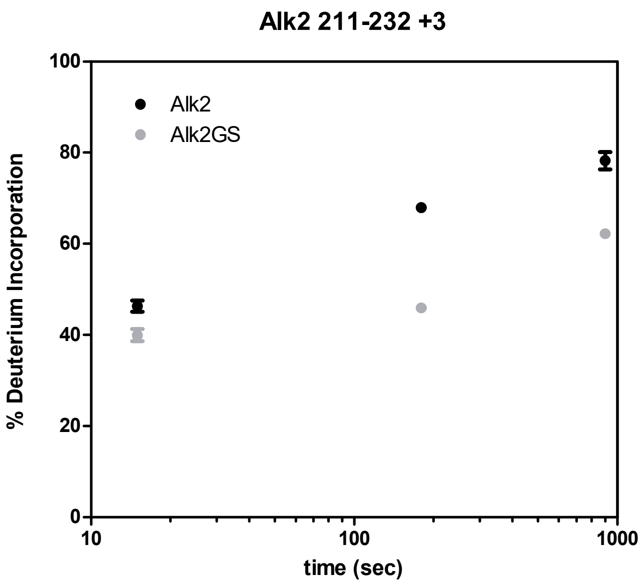

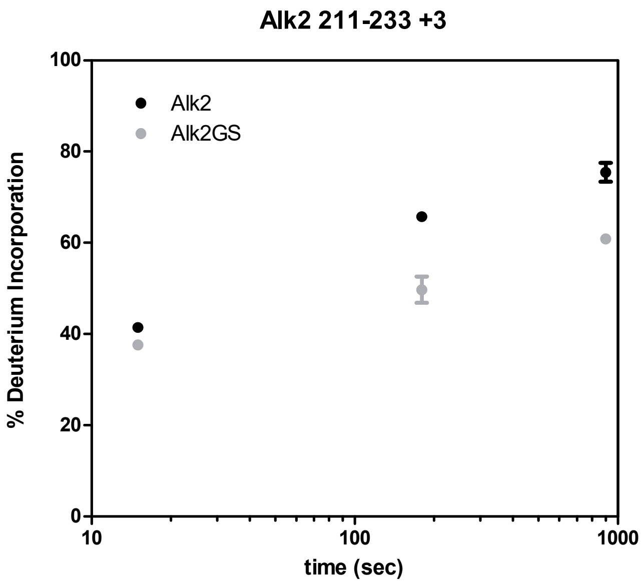

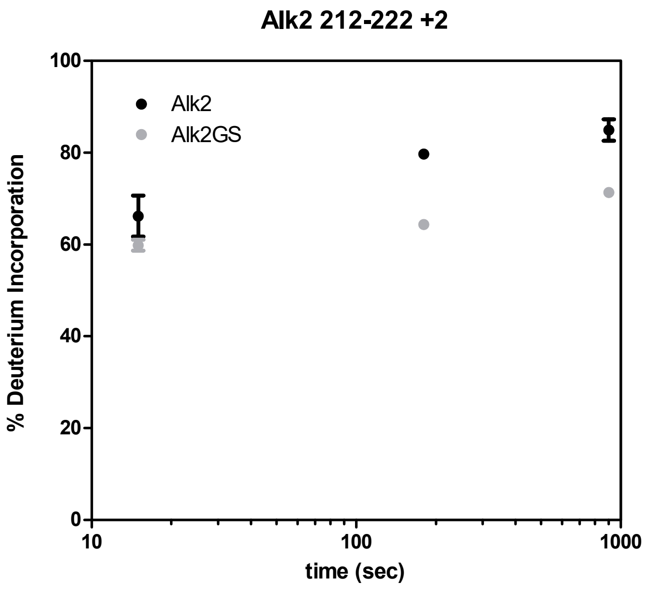

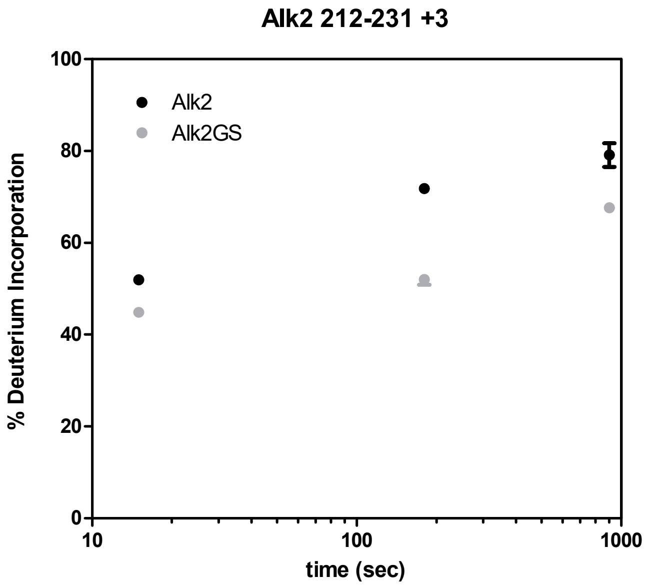

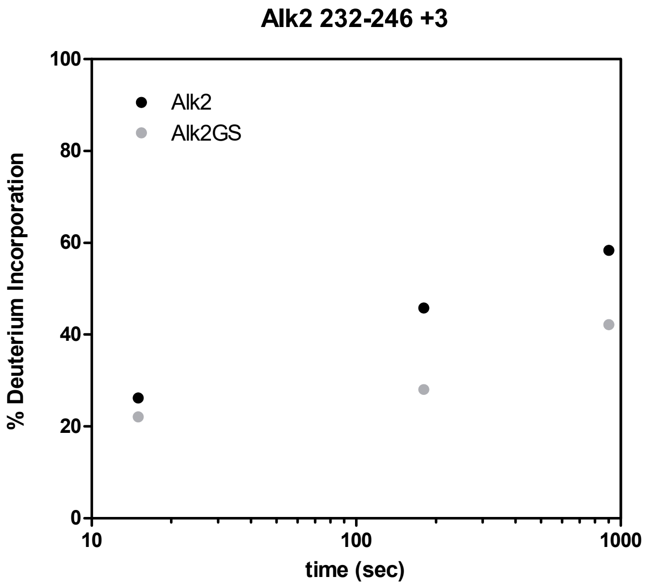

Alk2 vs. GS-Alk2 - HDX-MS Uptake Plots

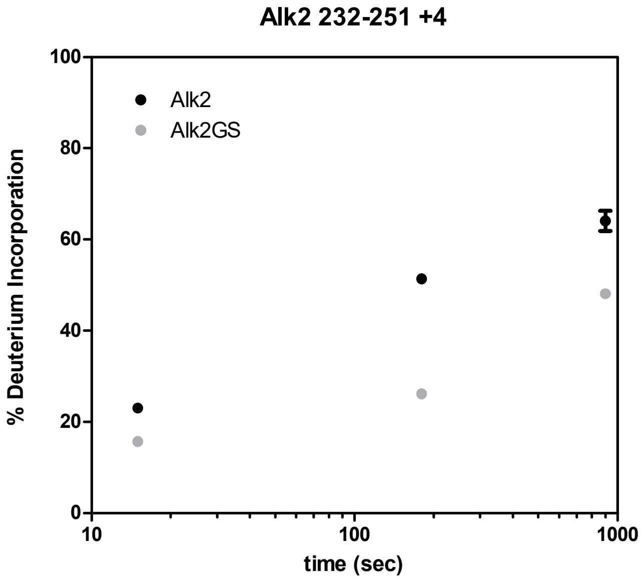

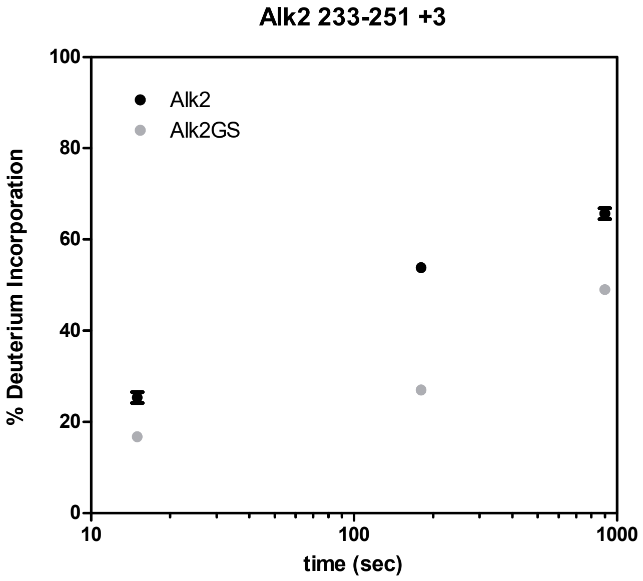

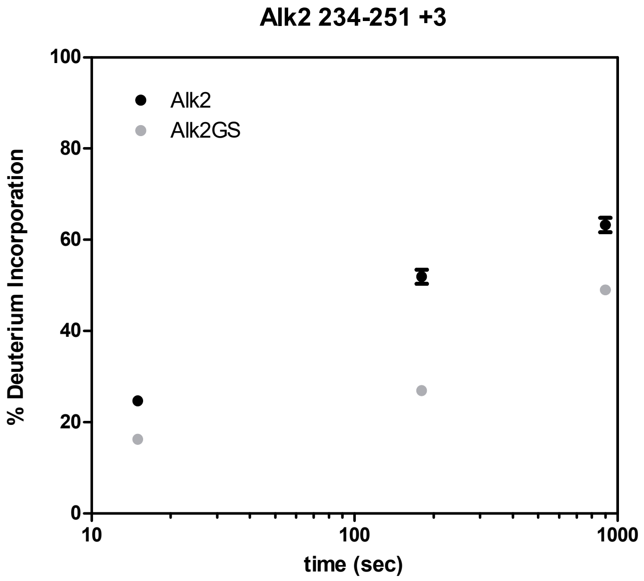

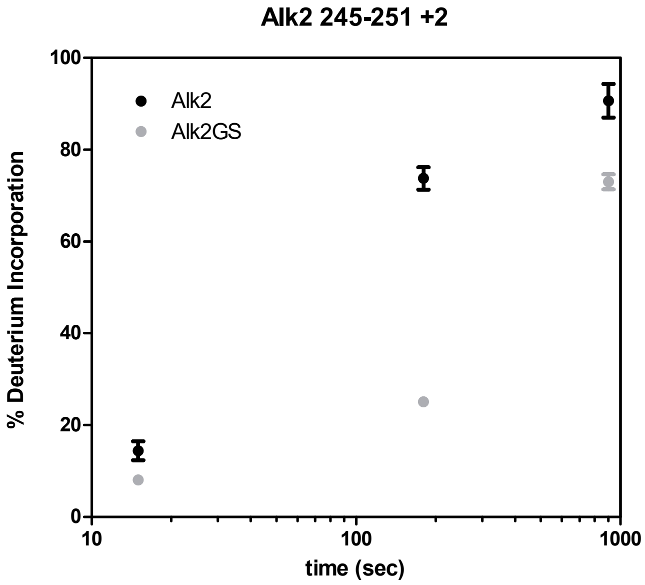

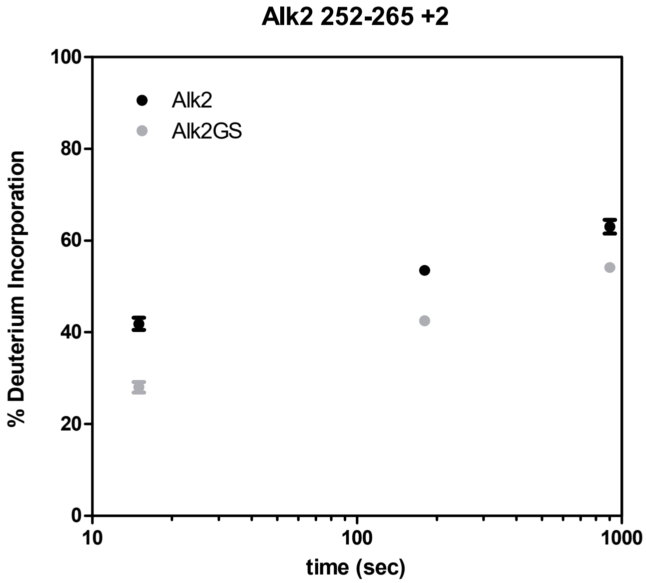

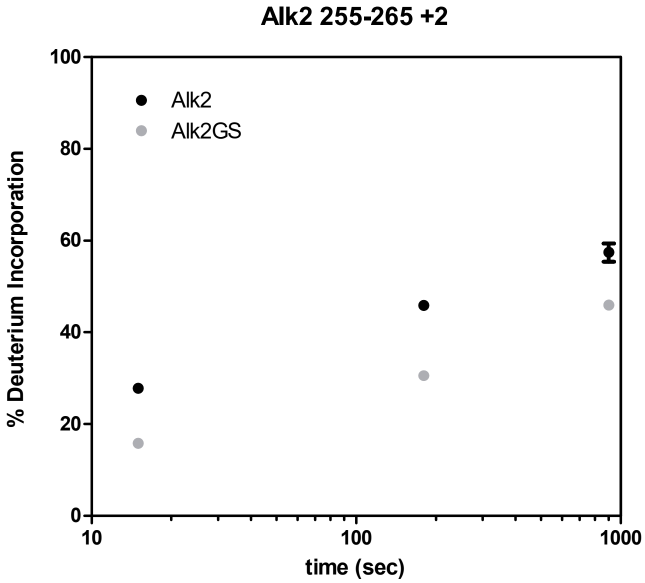

Alk2 vs. GS-Alk2 - HDX-MS Uptake Plots

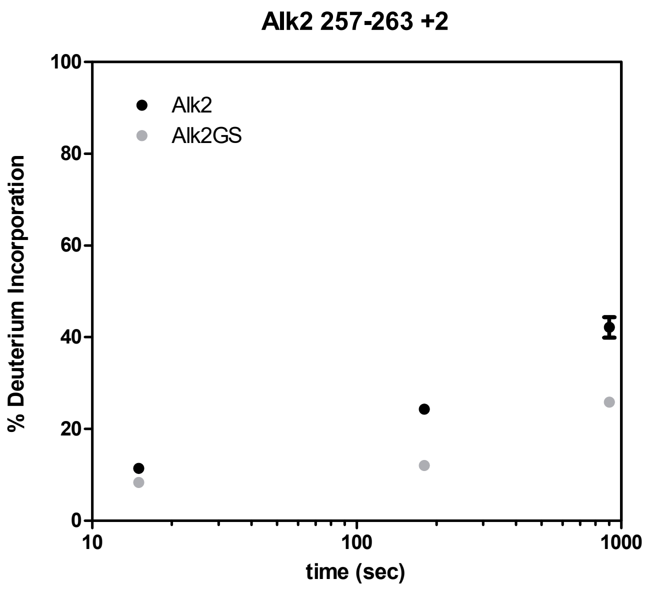

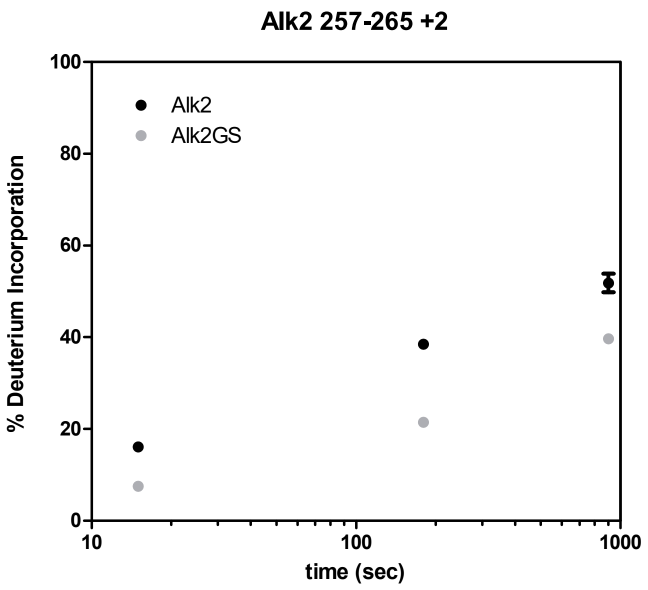

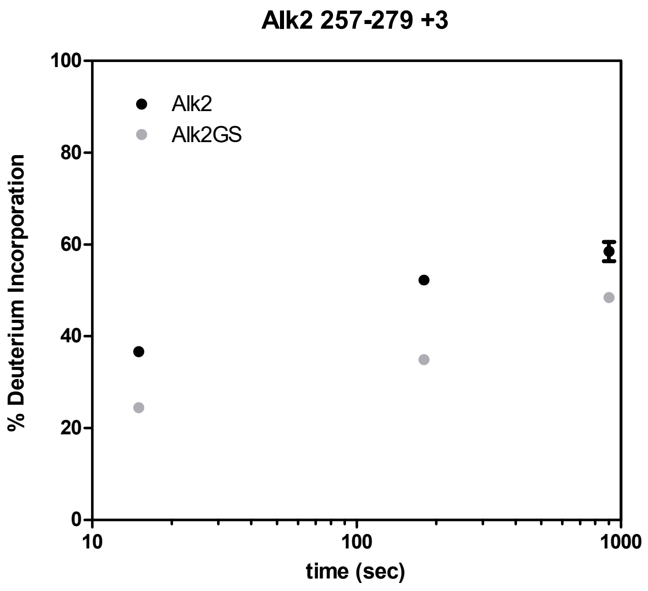

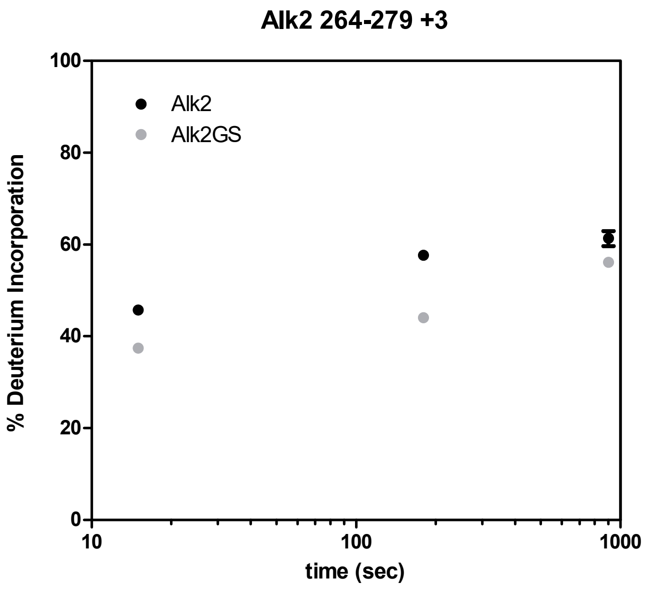

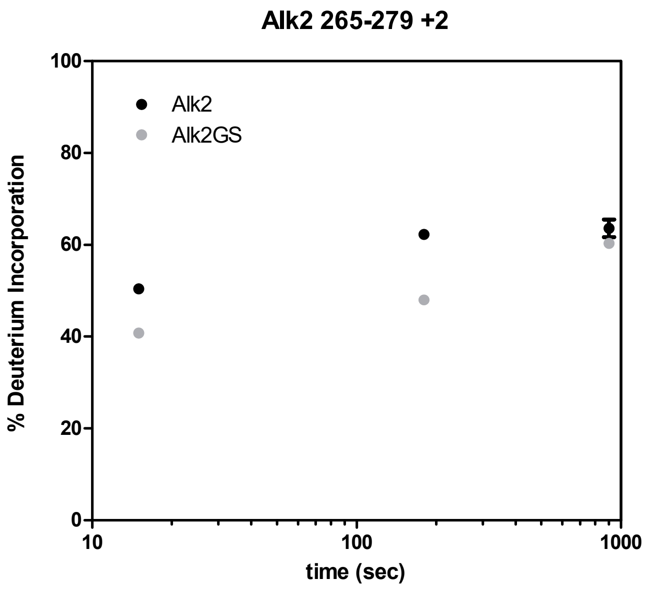

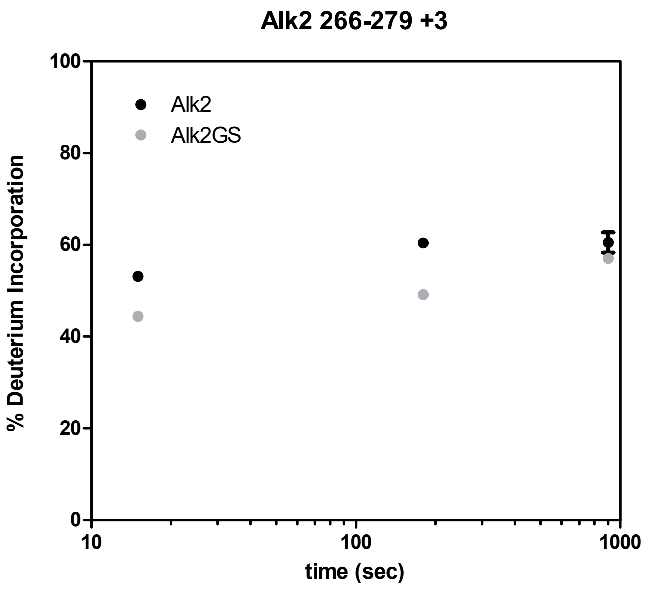

Alk2 vs. GS-Alk2 - HDX-MS Uptake Plots

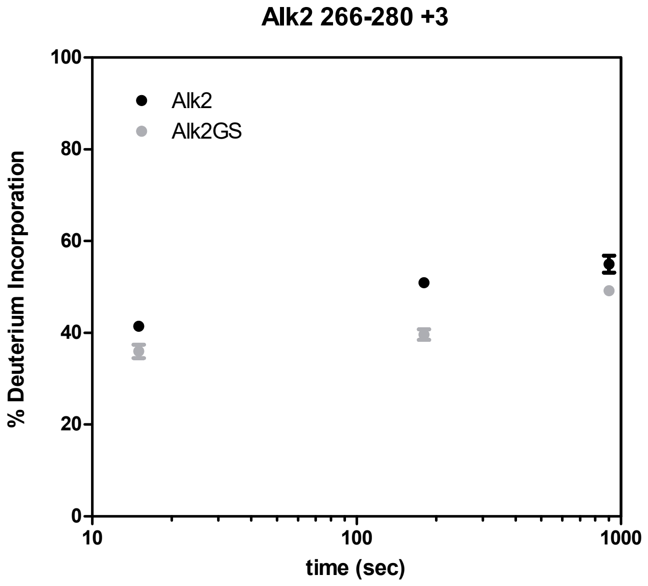

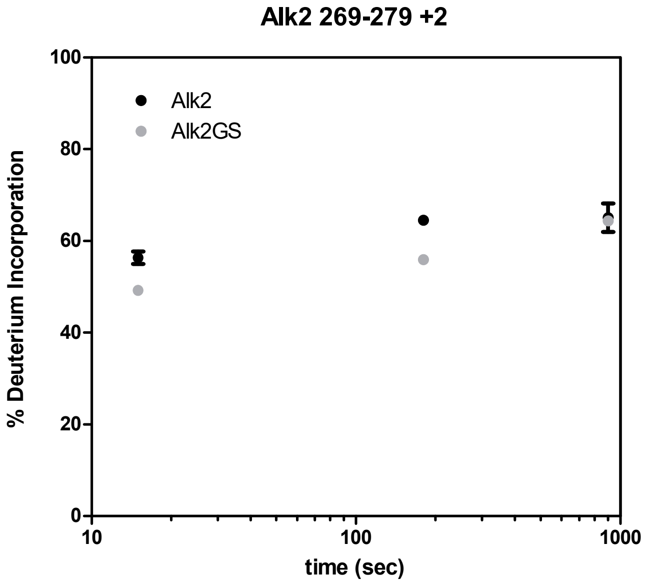

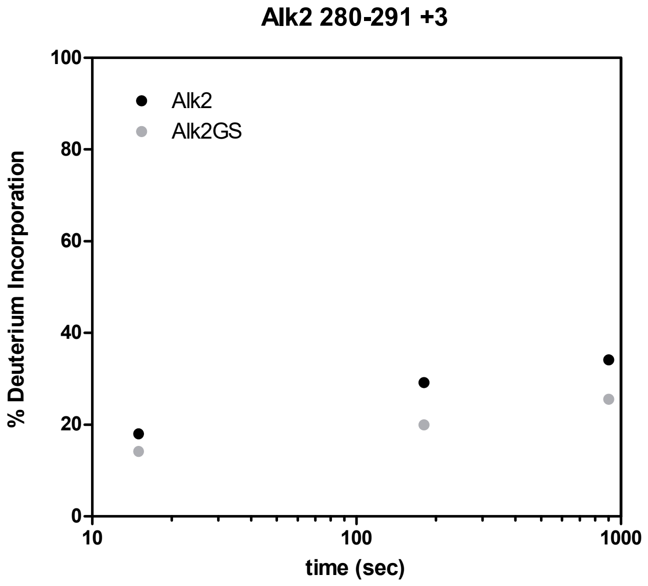

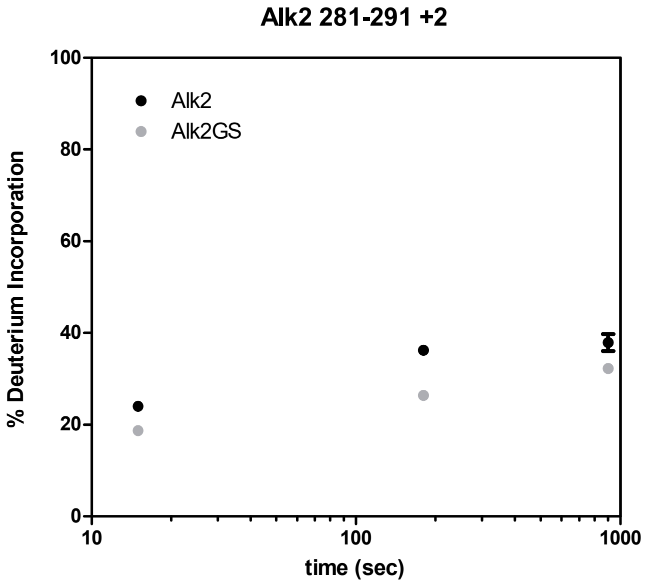

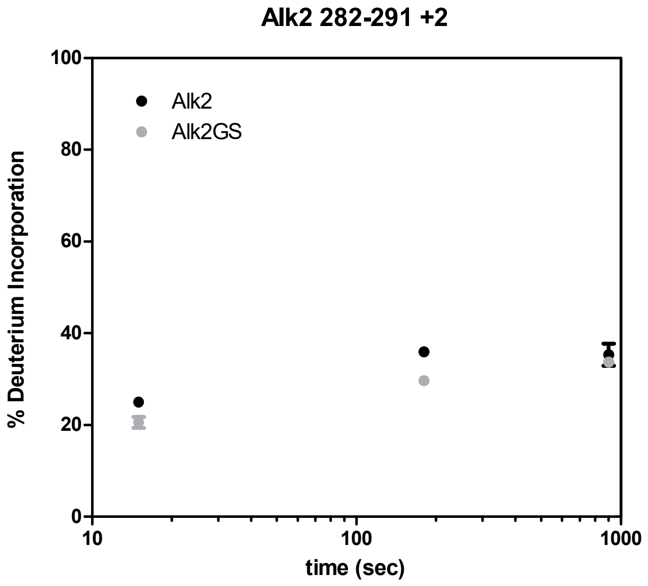

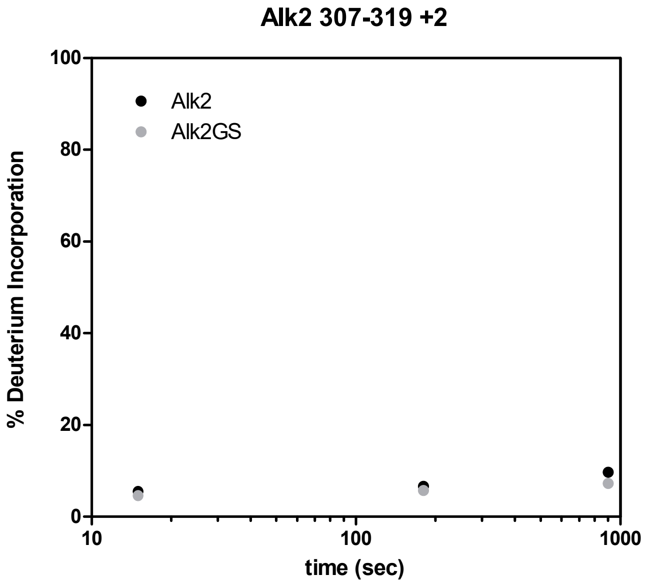

Alk2 vs. GS-Alk2 - HDX-MS Uptake Plots

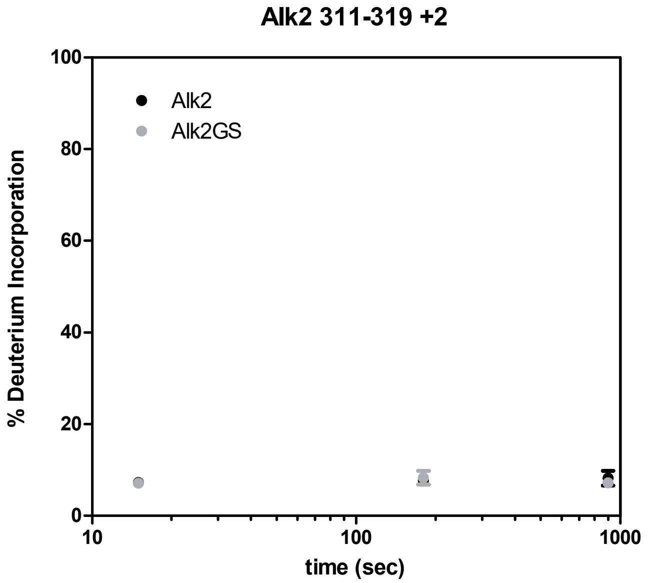

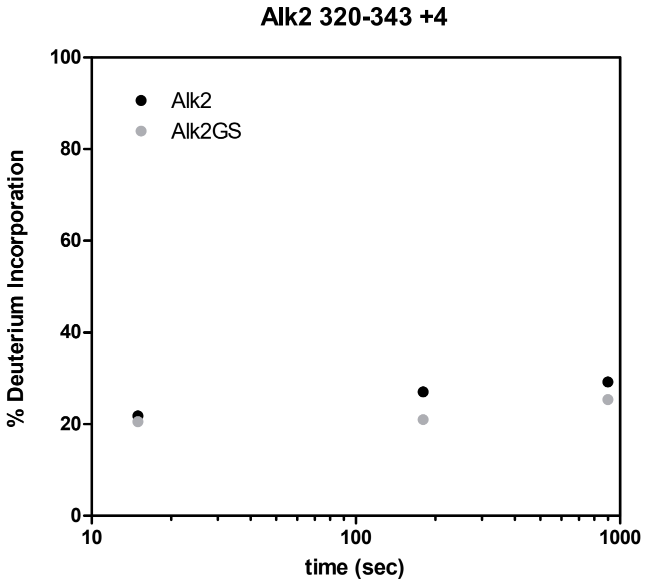

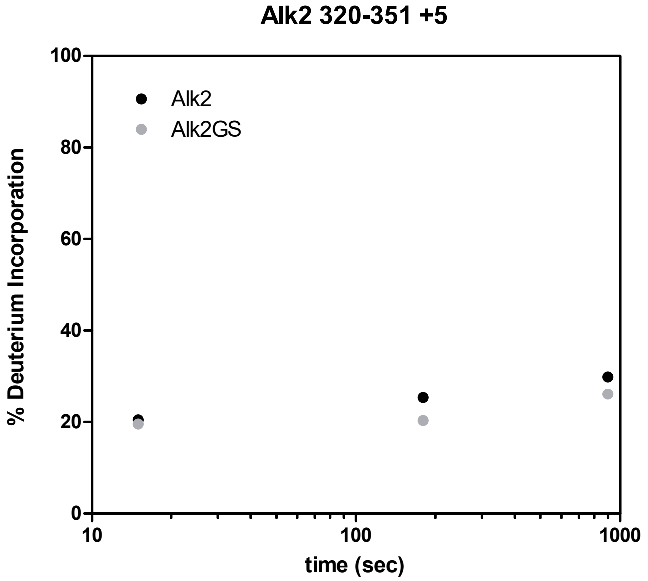

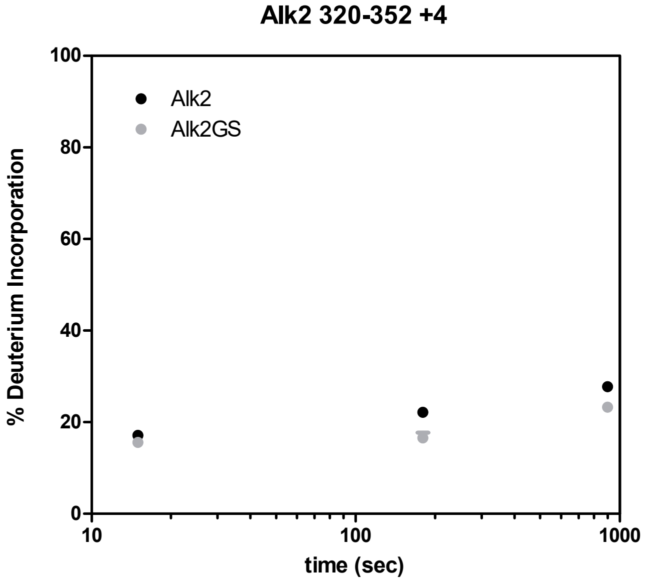

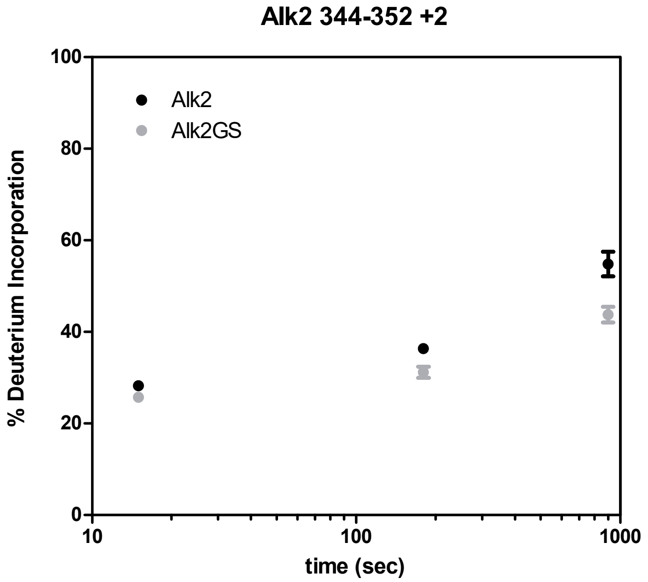

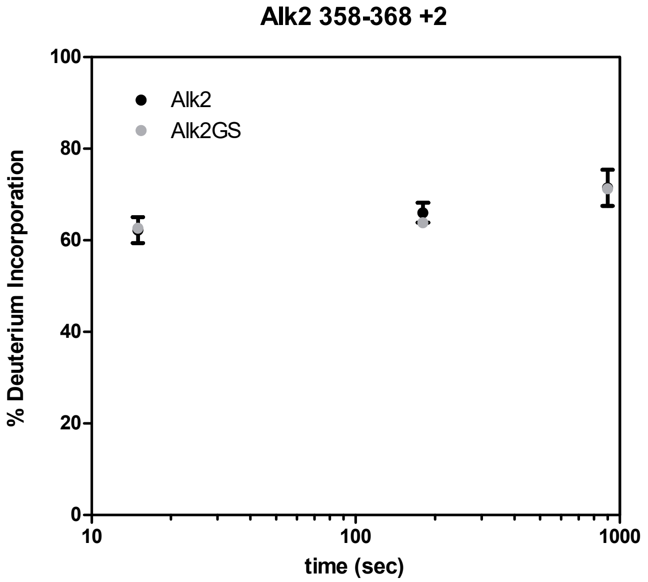

Alk2 vs. GS-Alk2 - HDX-MS Uptake Plots

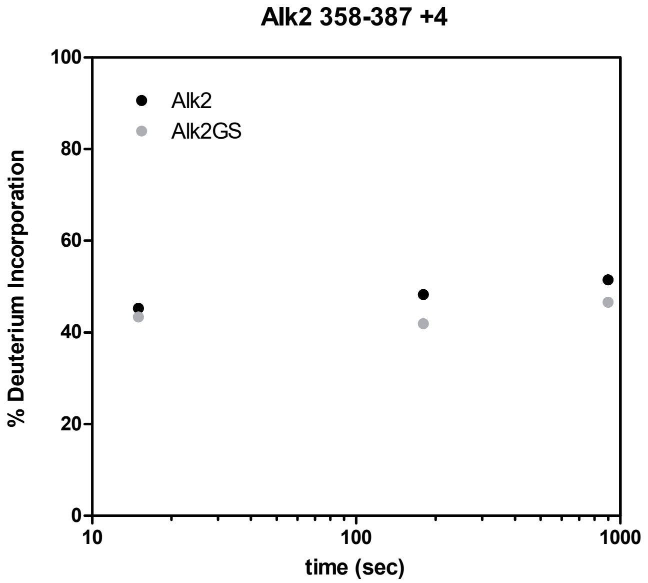

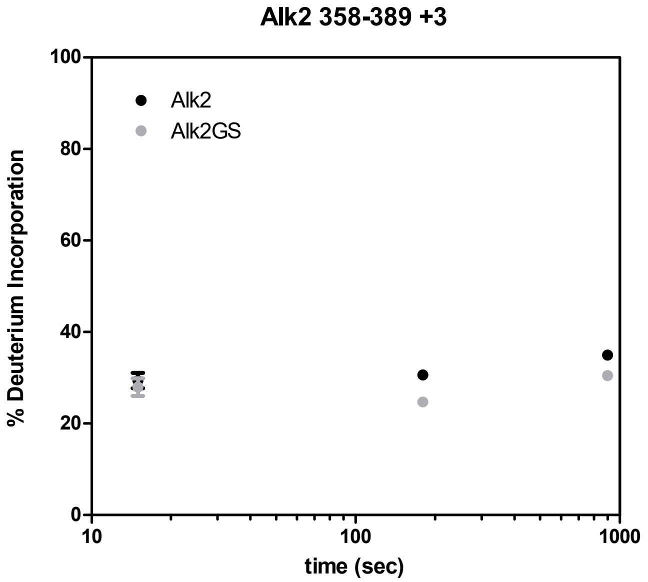

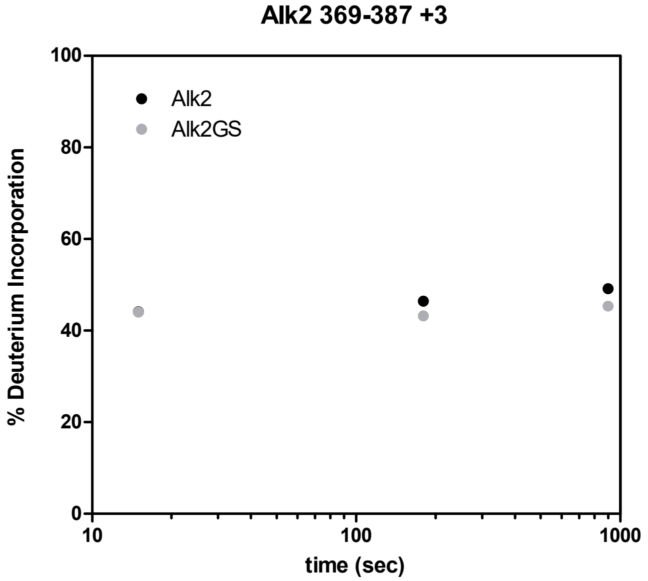

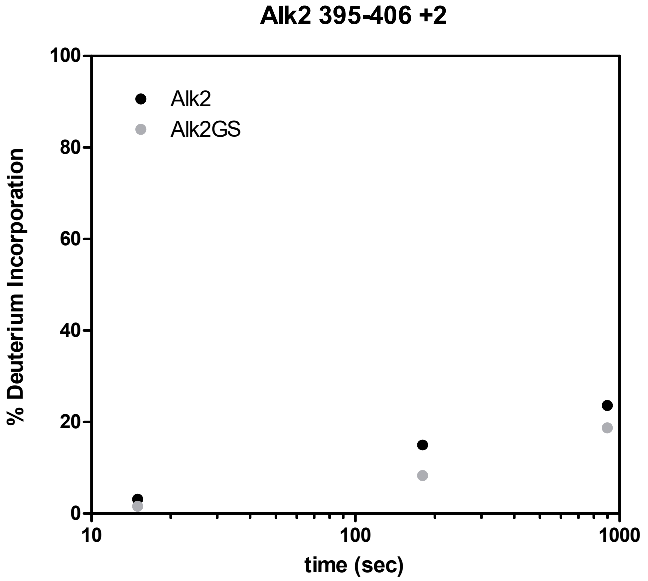

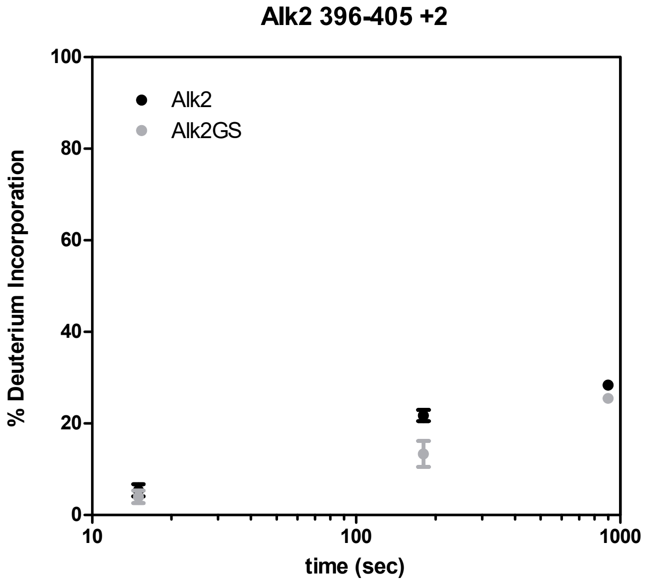

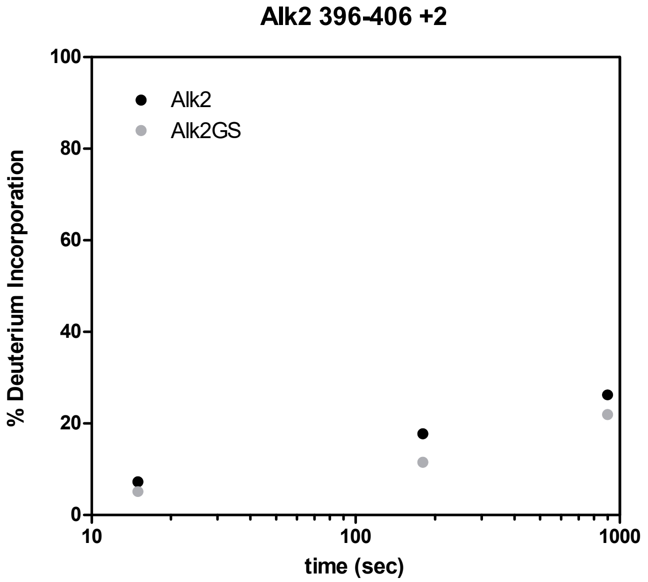

Alk2 vs. GS-Alk2 - HDX-MS Uptake Plots

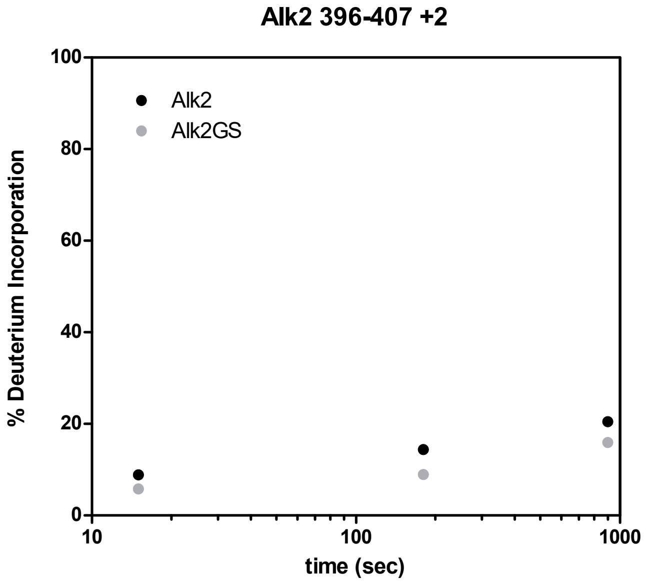

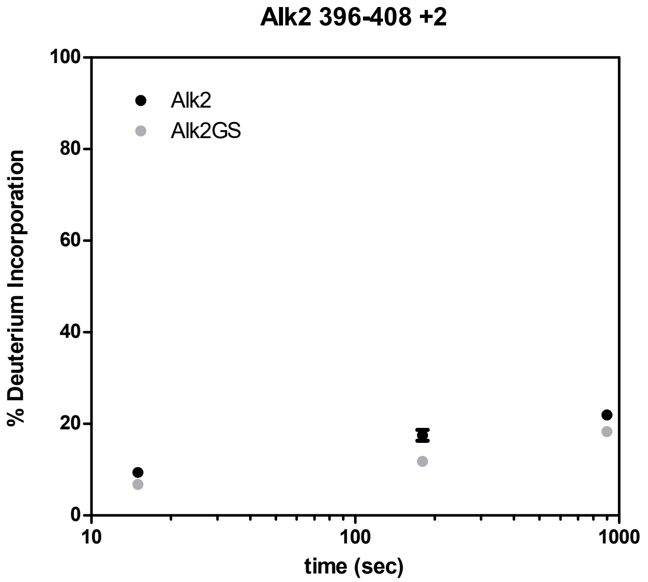

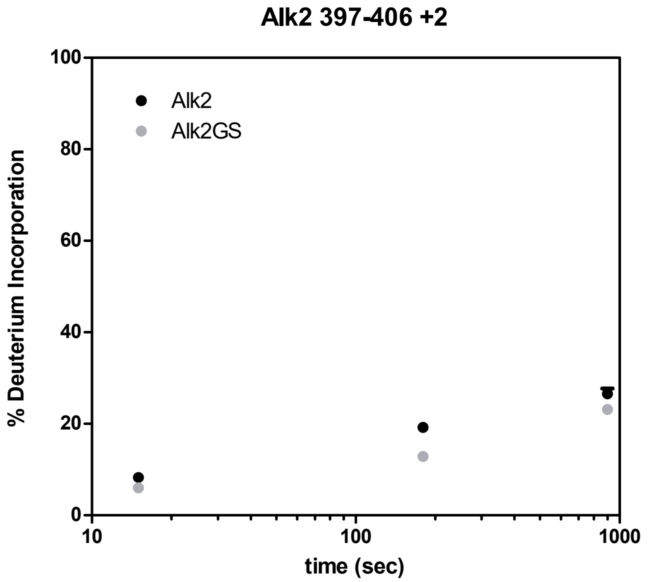

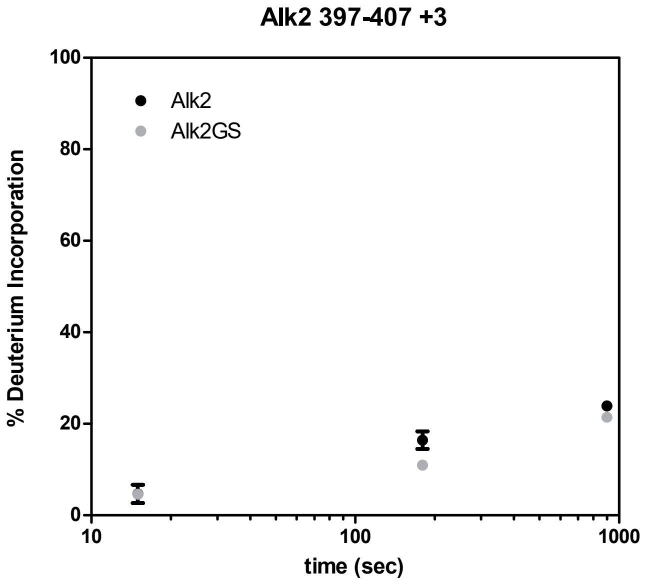

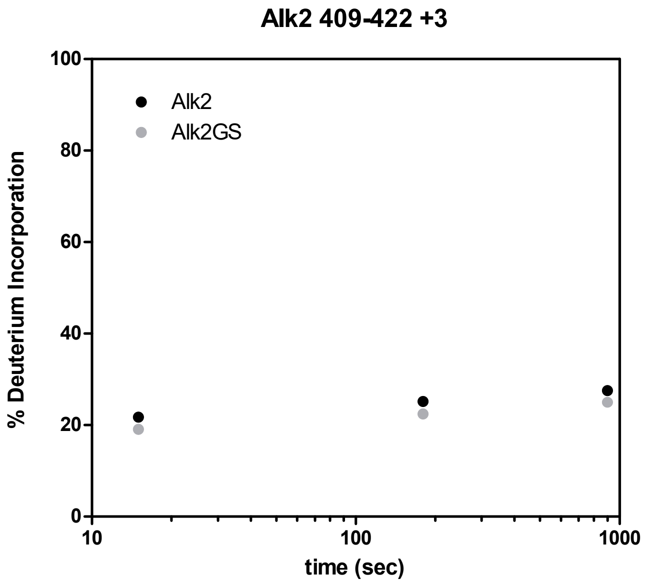

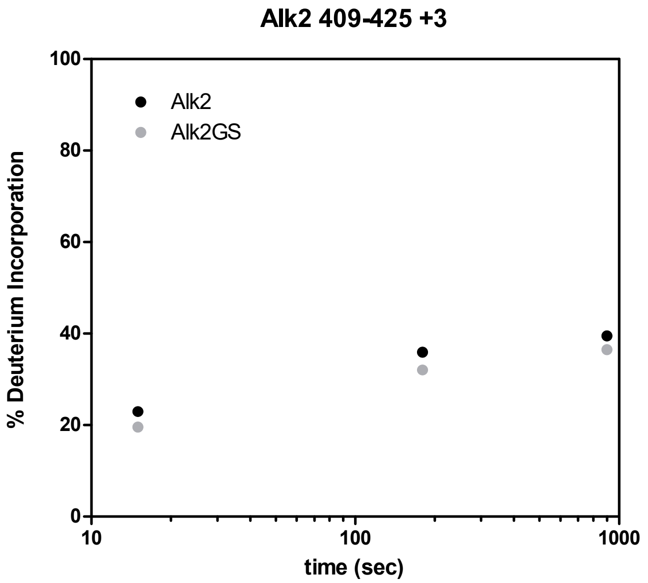

Alk2 vs. GS-Alk2 - HDX-MS Uptake Plots

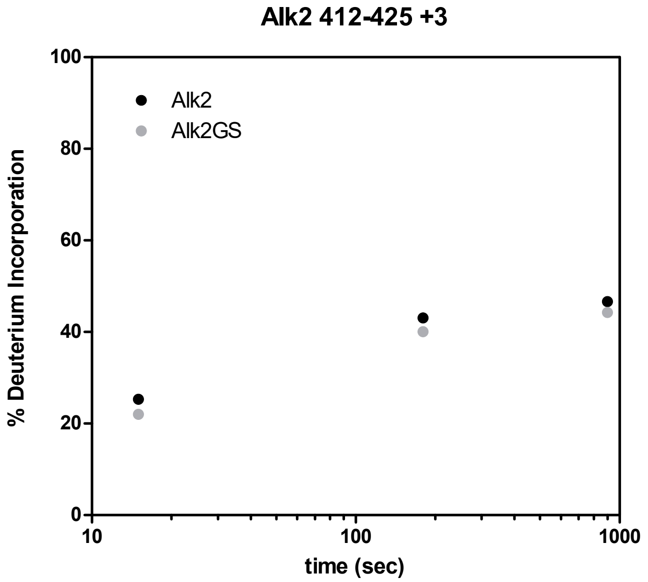

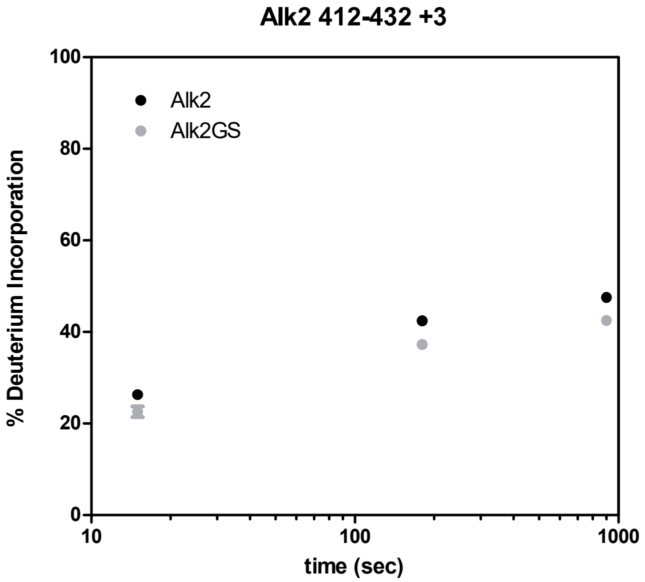

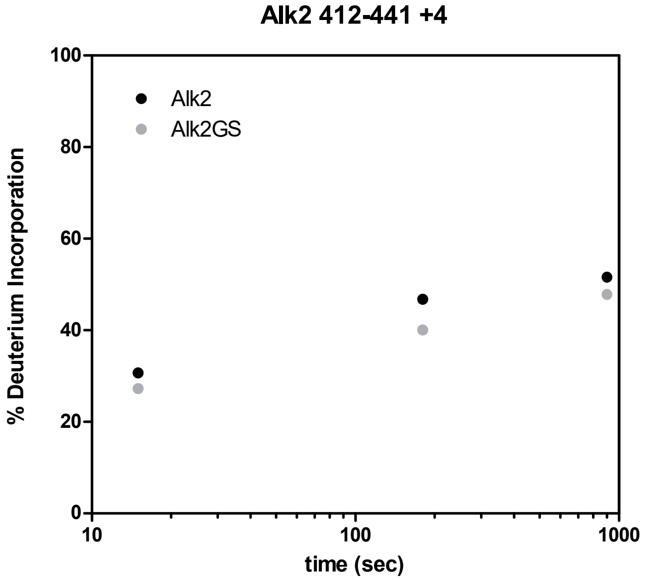

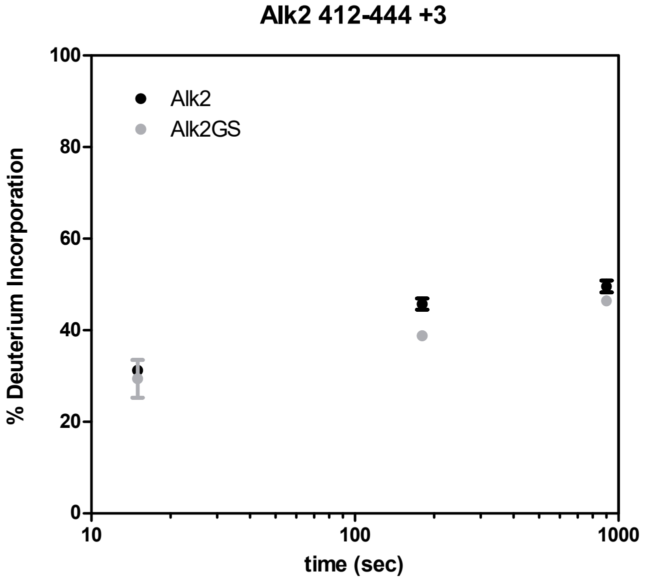

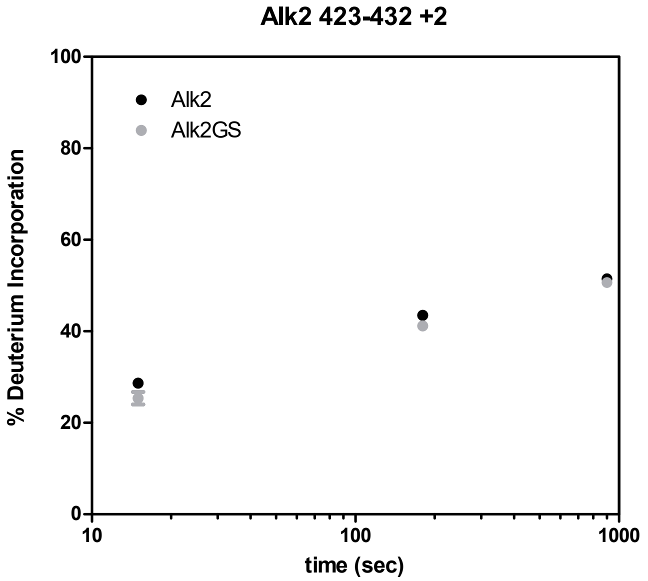

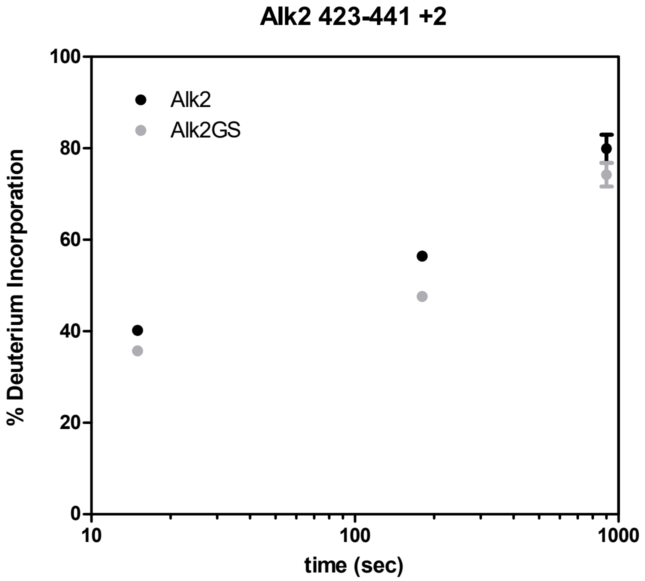

Alk2 vs. GS-Alk2 - HDX-MS Uptake Plots

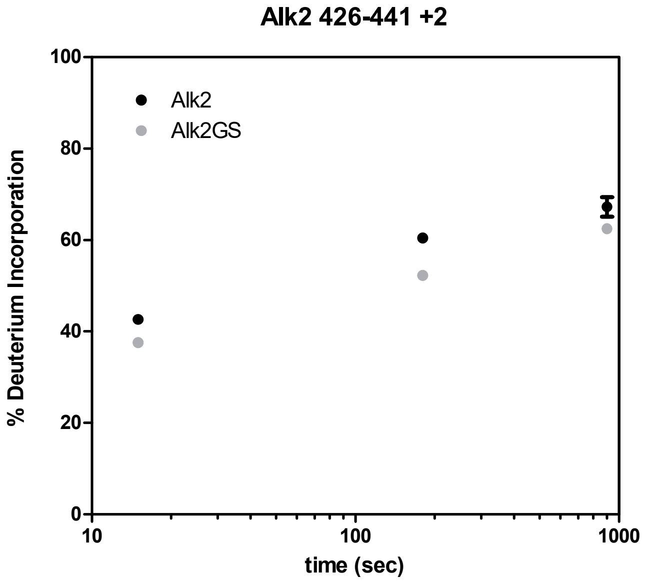

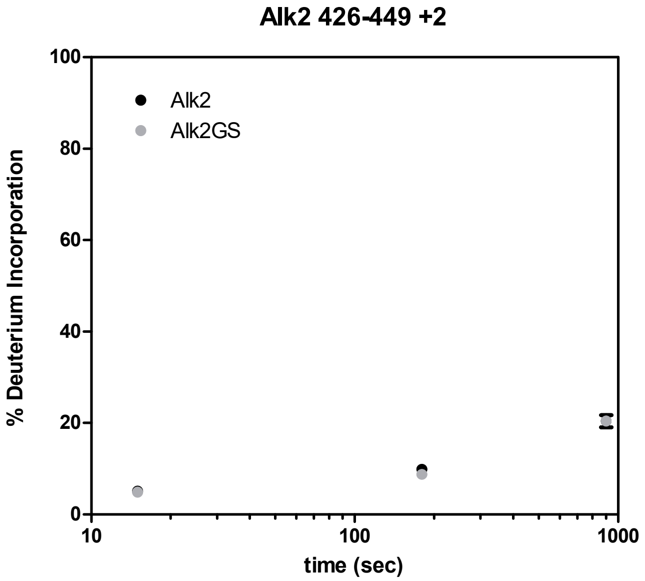

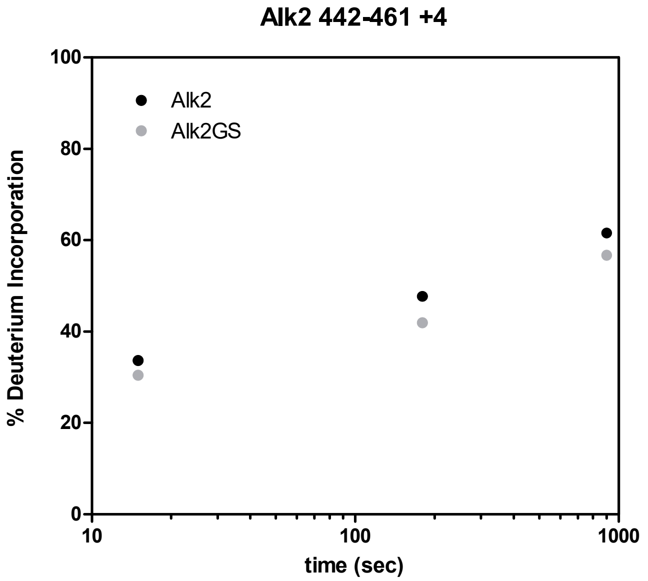

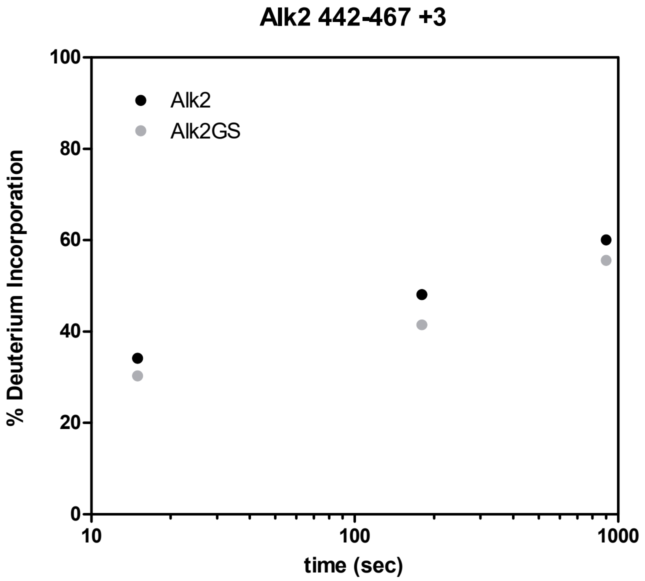

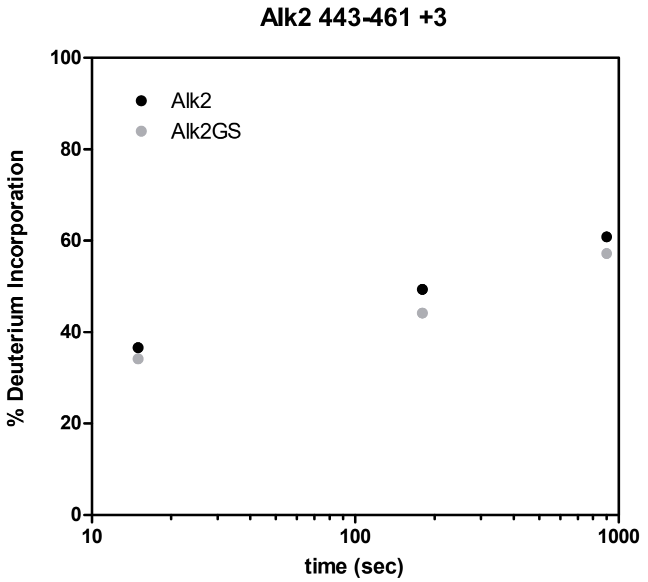

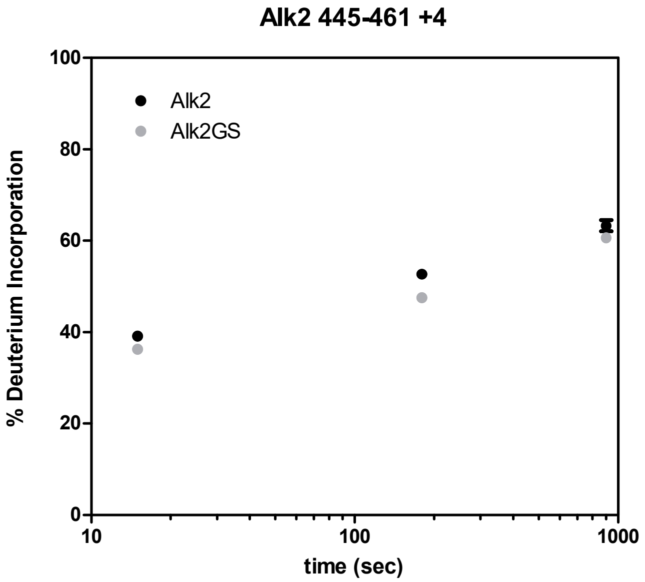

Alk2 vs. GS-Alk2 - HDX-MS Uptake Plots

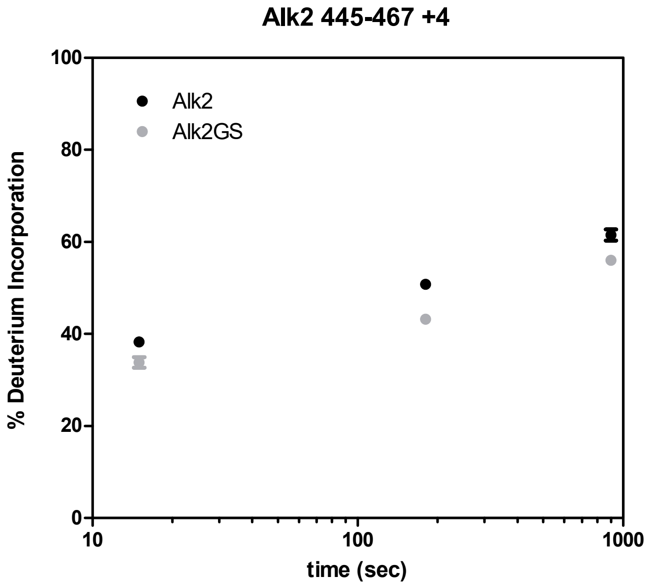

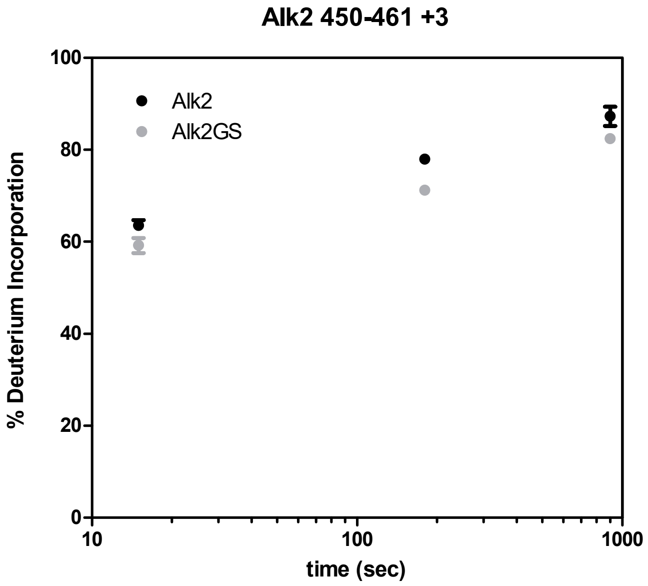

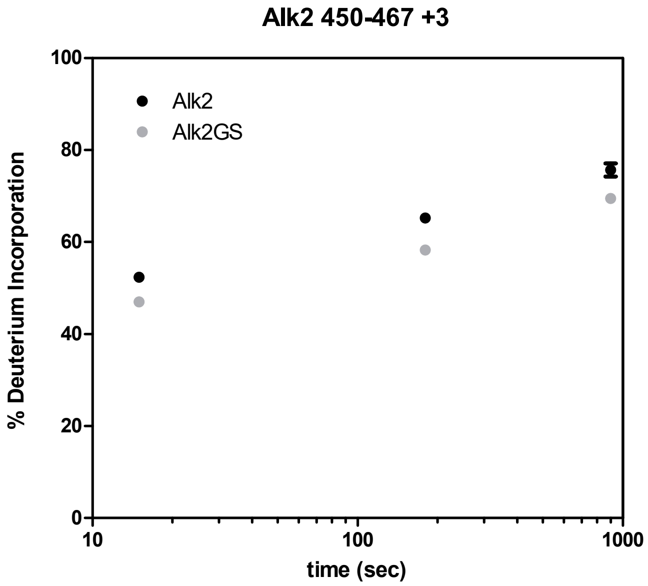

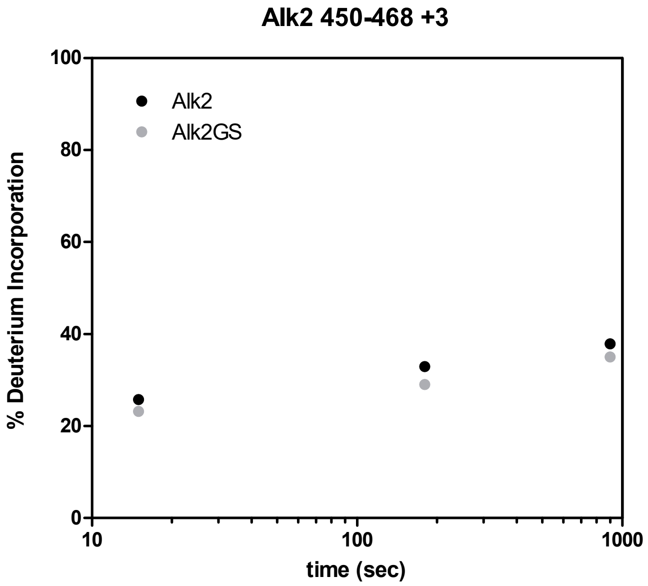

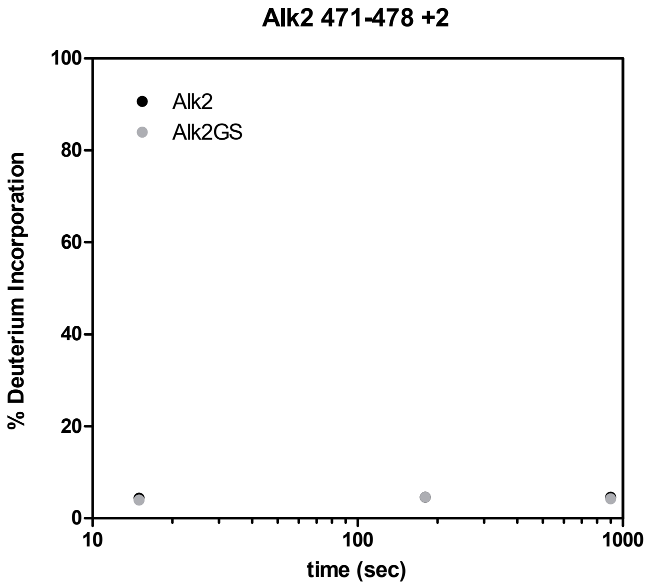

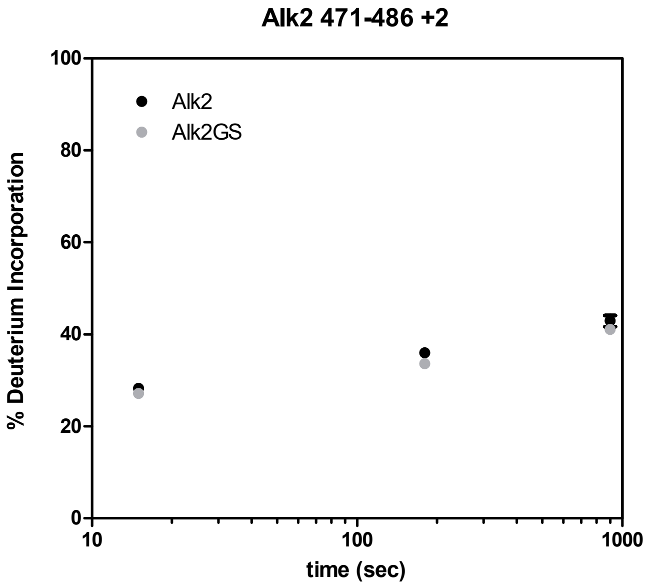

Alk2 vs. GS-Alk2 - HDX-MS Uptake Plots

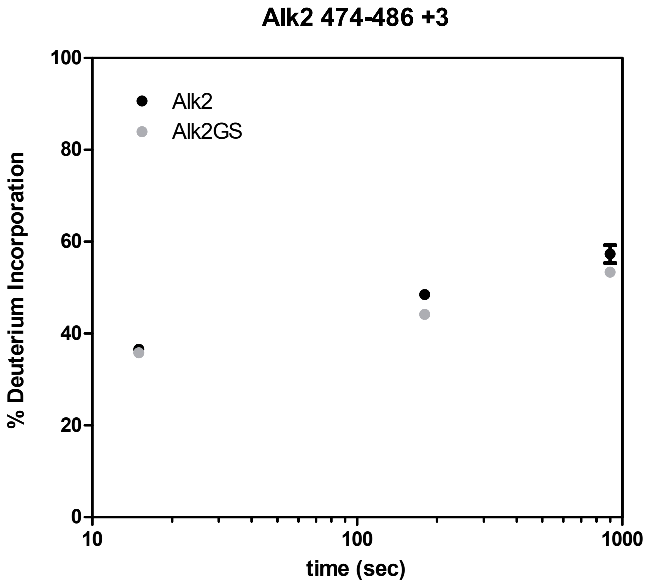

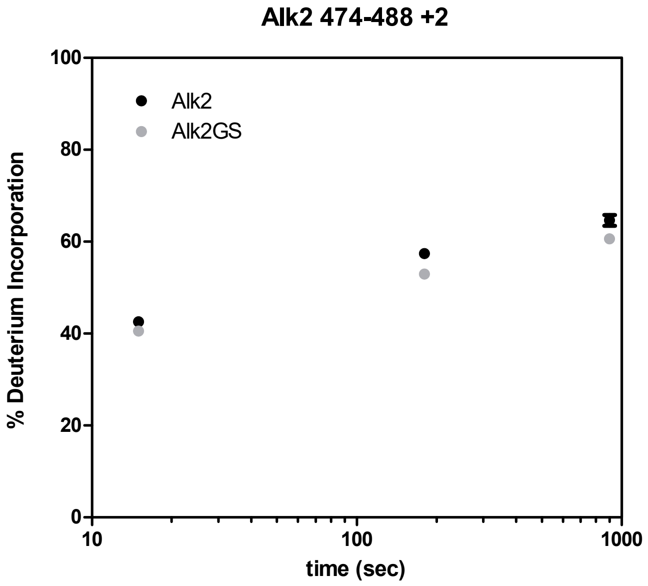

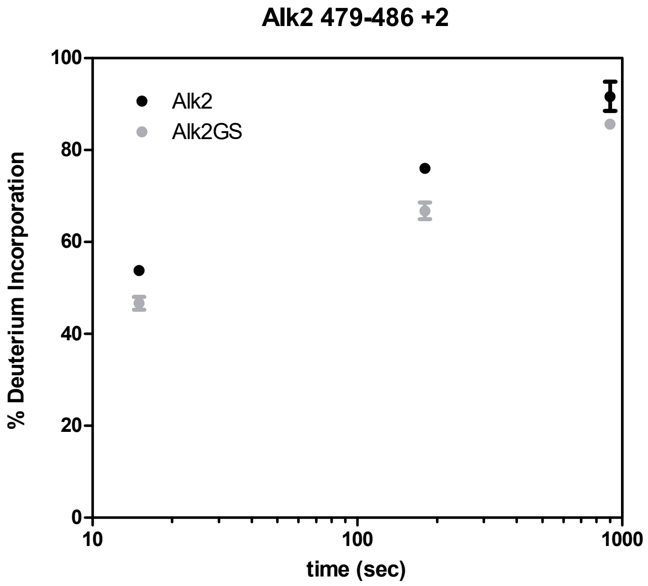

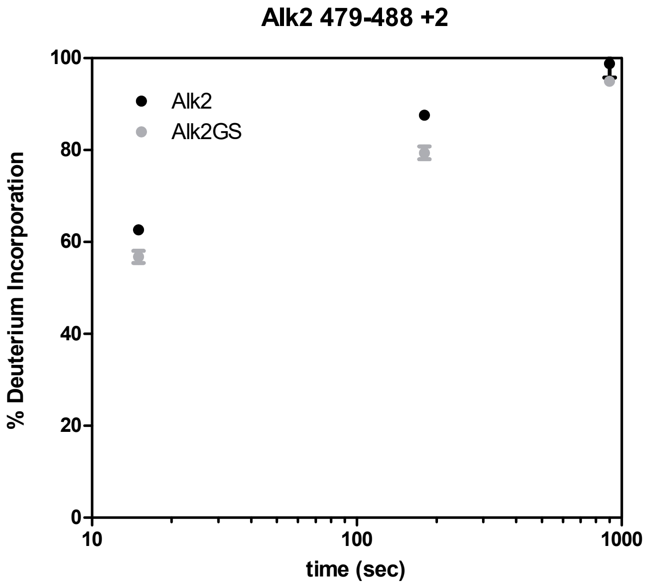

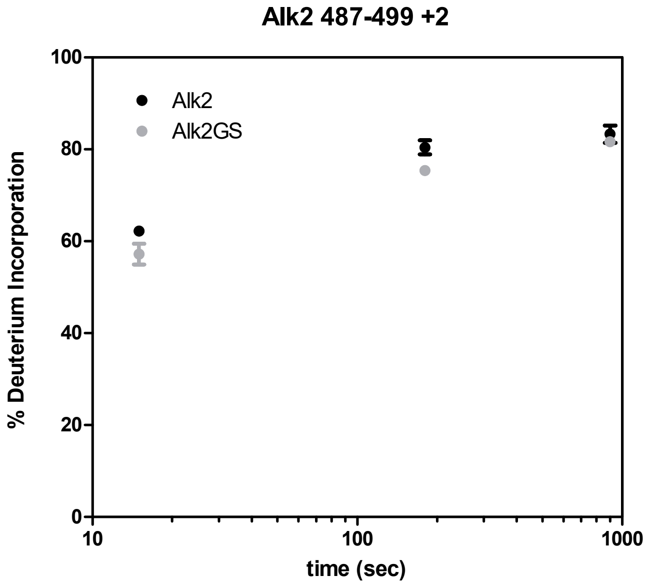

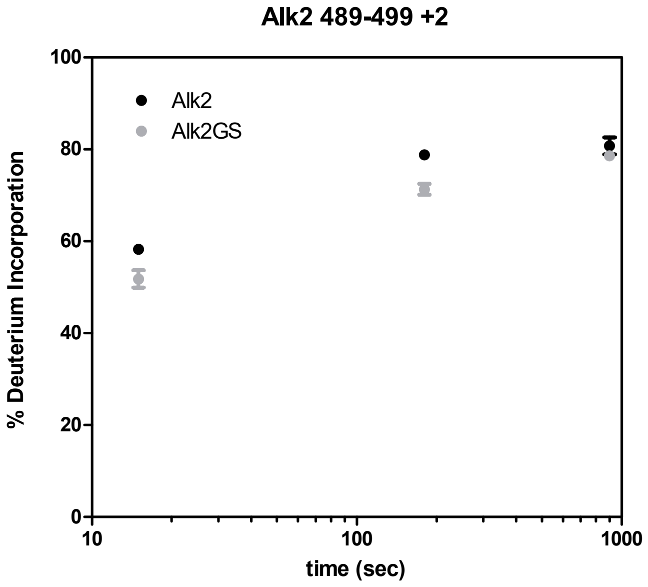

Alk2 vs. GS-Alk2 - HDX-MS Uptake Plots

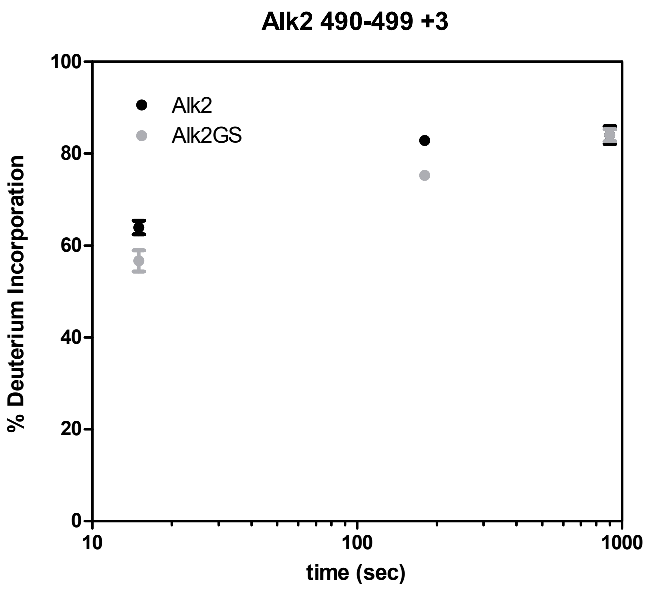

Alk2:BMPR2 complex vs. GS-Alk2:BMPR2 complex - HDX-MS Uptake Plots

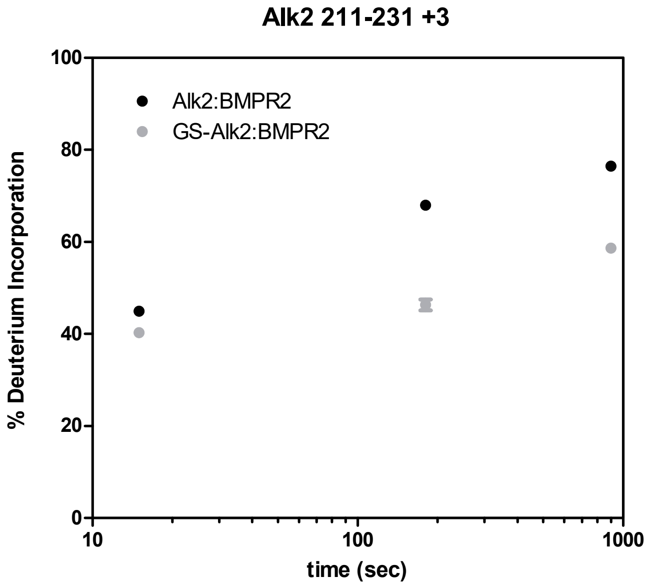

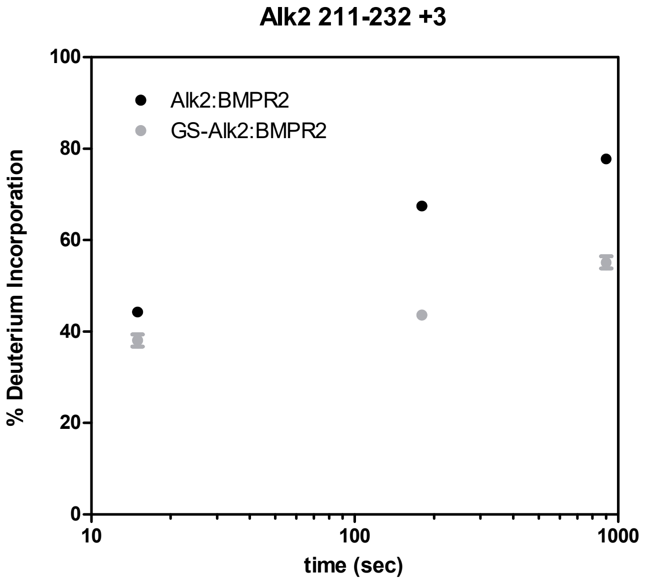

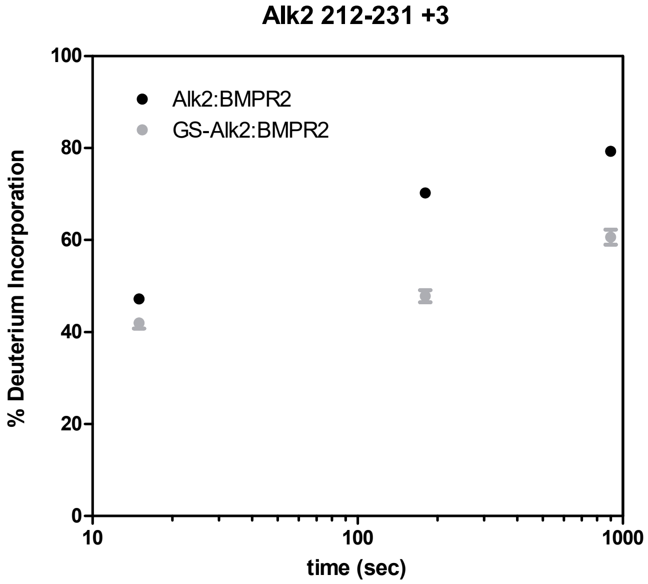

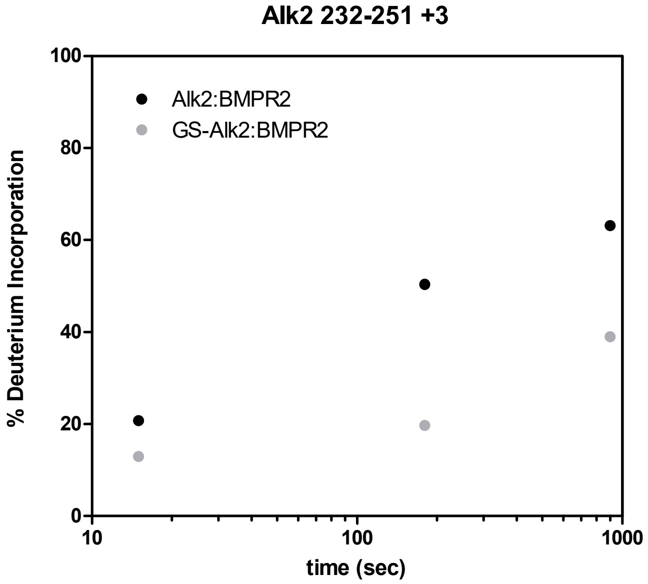

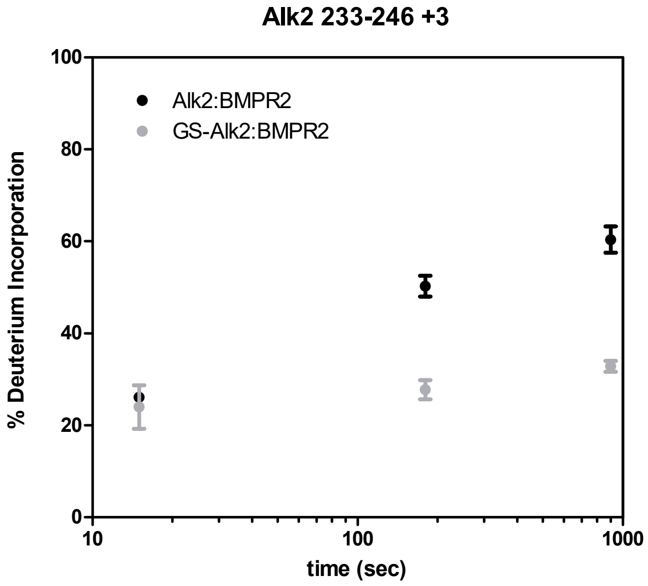

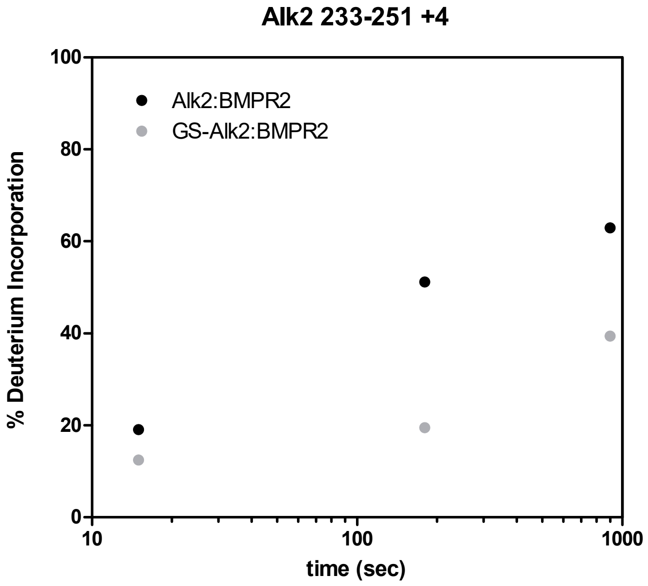

Alk2:BMPR2 complex vs. GS-Alk2:BMPR2 complex - HDX-MS Uptake Plots

Alk2 234-251 +3

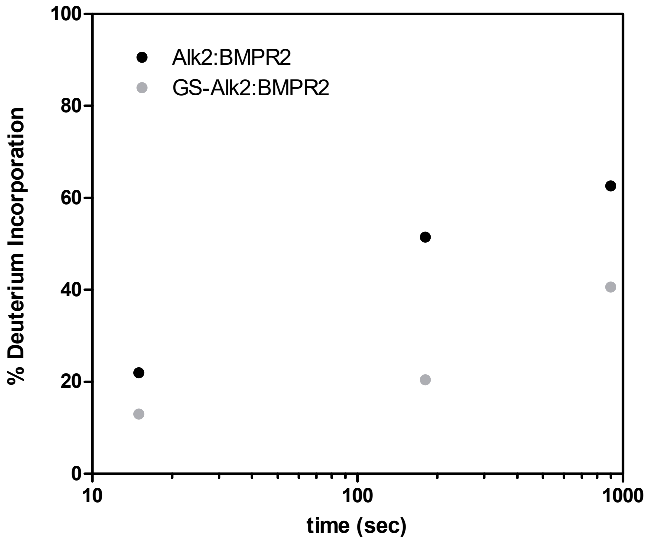

Alk2 245-251 +2

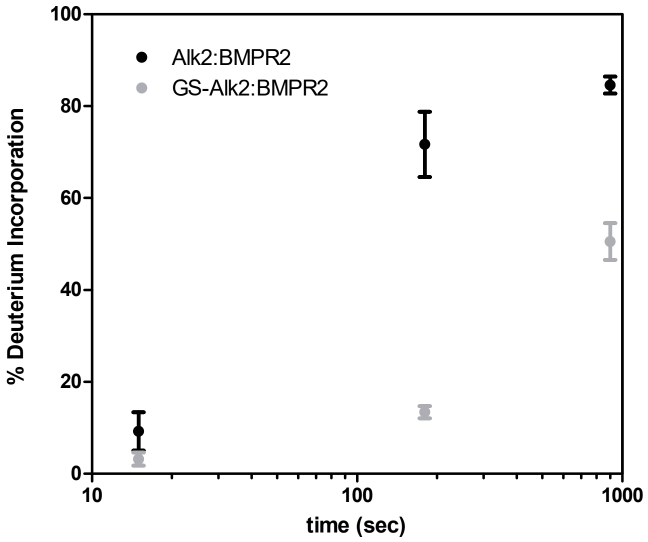

Alk2 252-265 +2

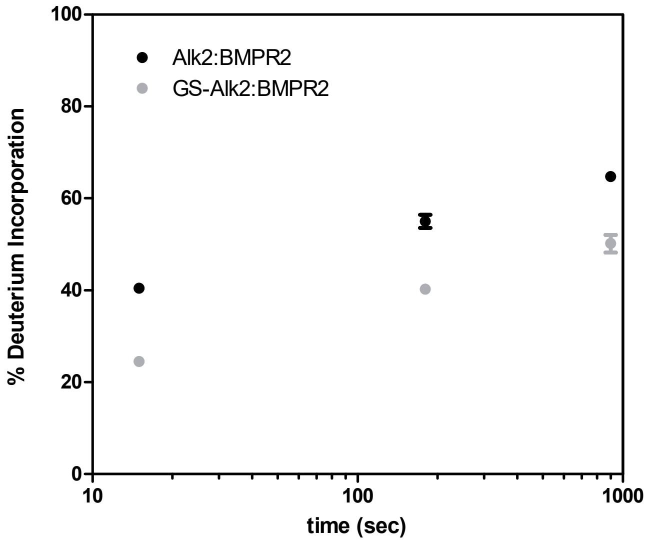

Alk2 255-265 +2

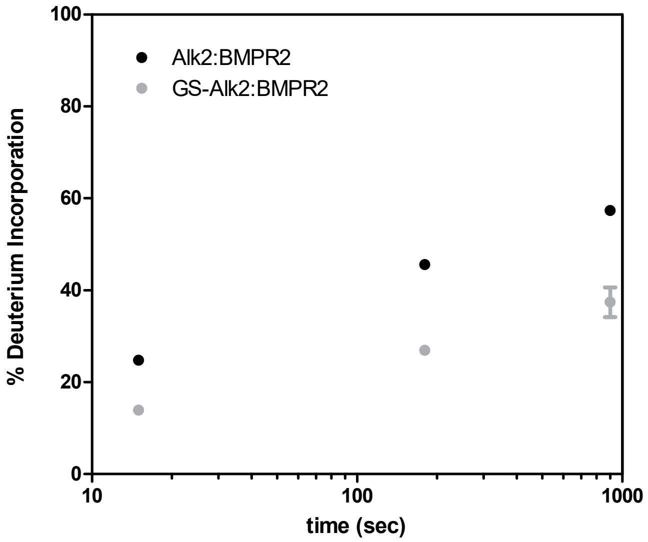

Alk2 257-263 +2

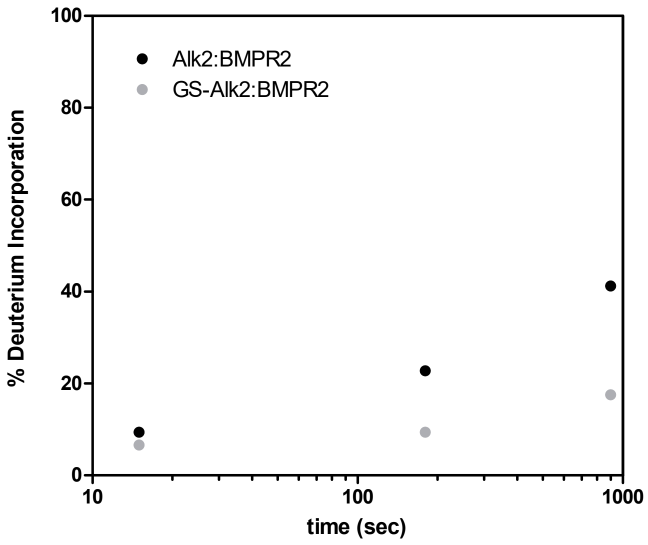

Alk2 257-265 +2

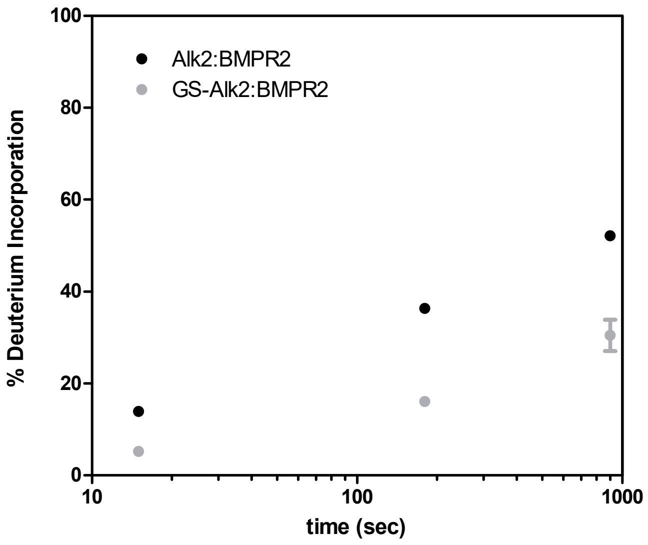

Alk2:BMPR2 complex vs. GS-Alk2:BMPR2 complex - HDX-MS Uptake Plots

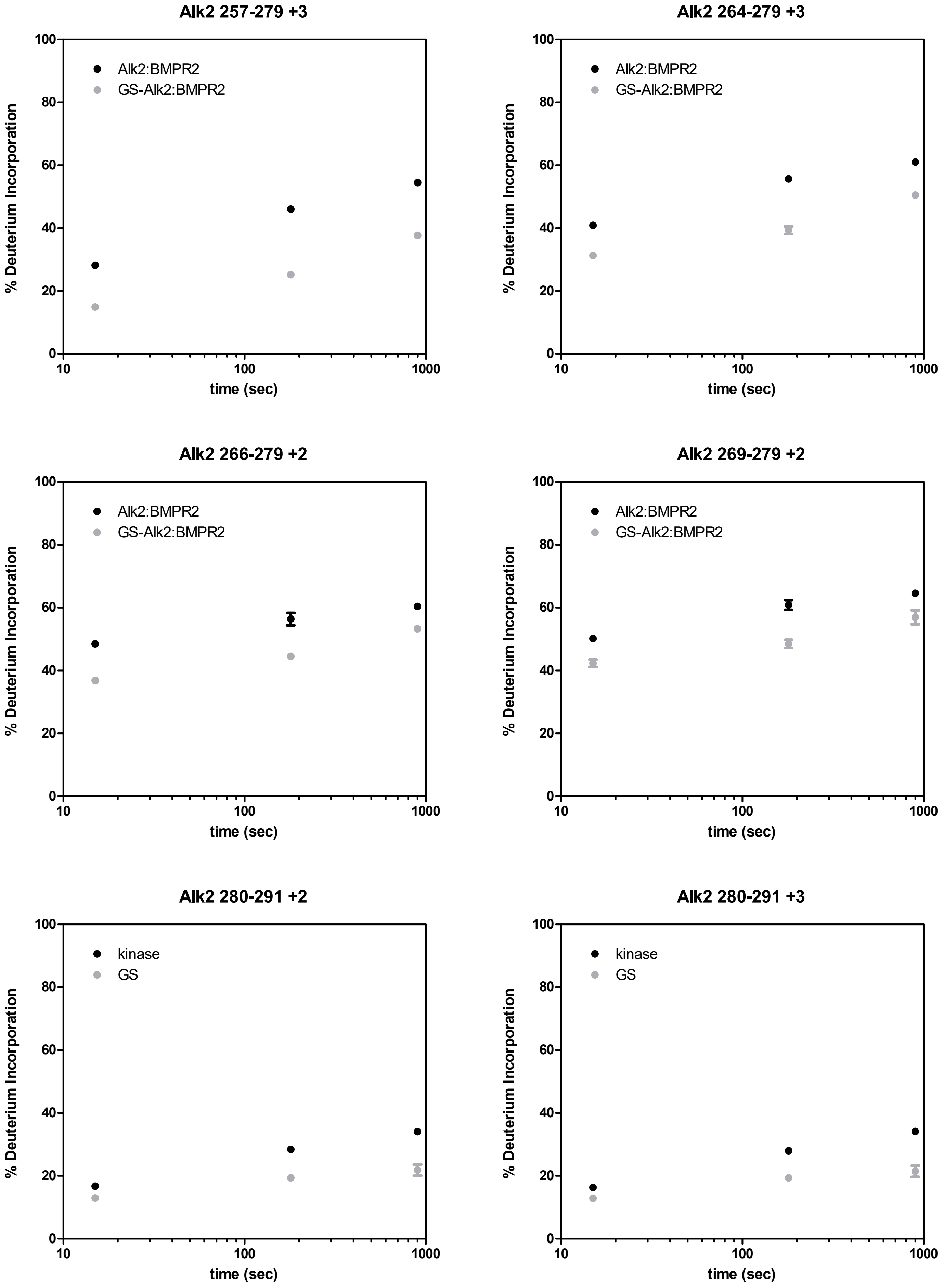

Alk2:BMPR2 complex vs. GS-Alk2:BMPR2 complex - HDX-MS Uptake Plots

Alk2 281-291 +2

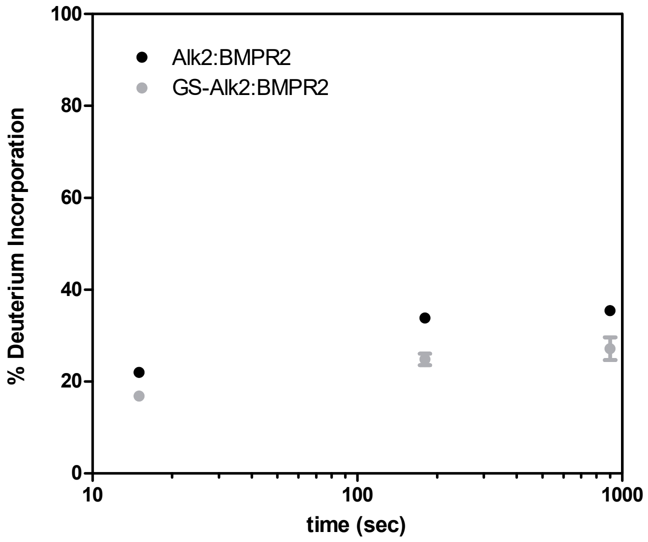

Alk2 282-291 +2

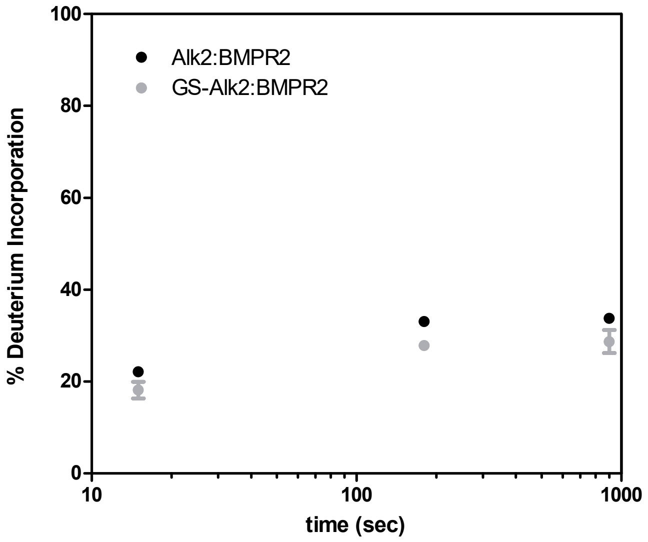

Alk2 307-319 +2

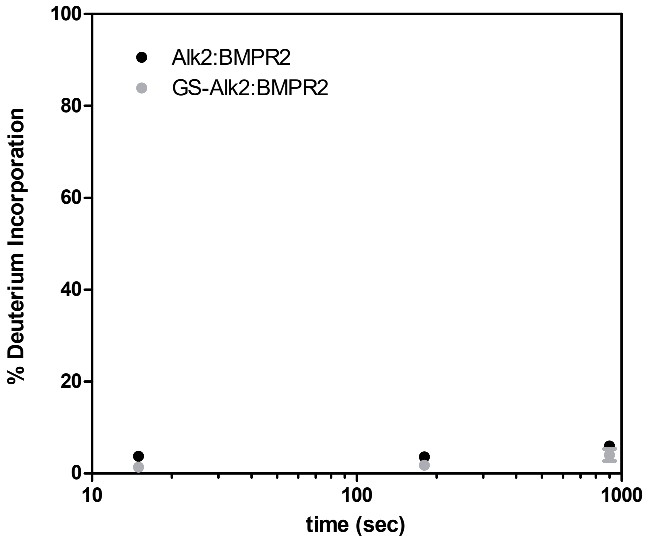

Alk2 311-319 +2

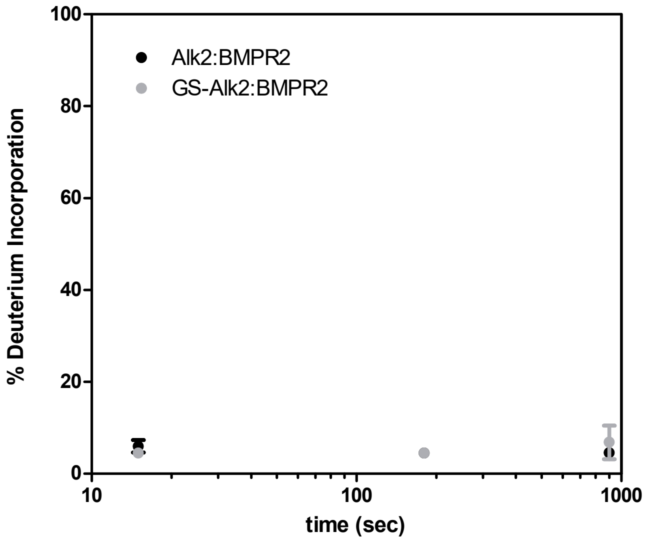

Alk2 320-343 +4

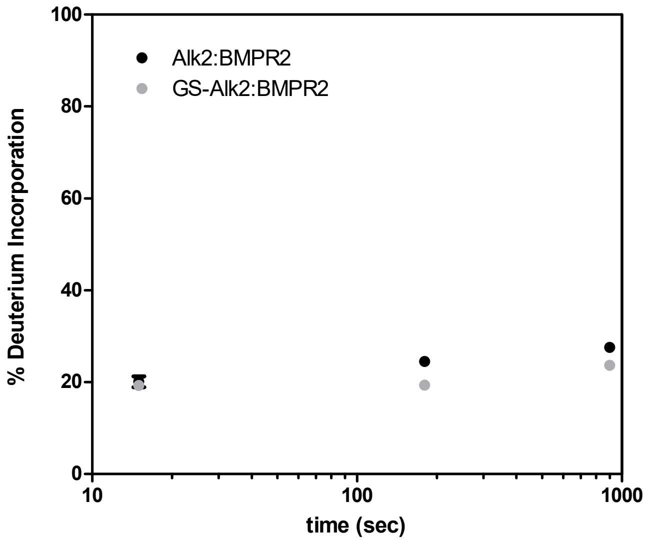

Alk2 320-351 +5

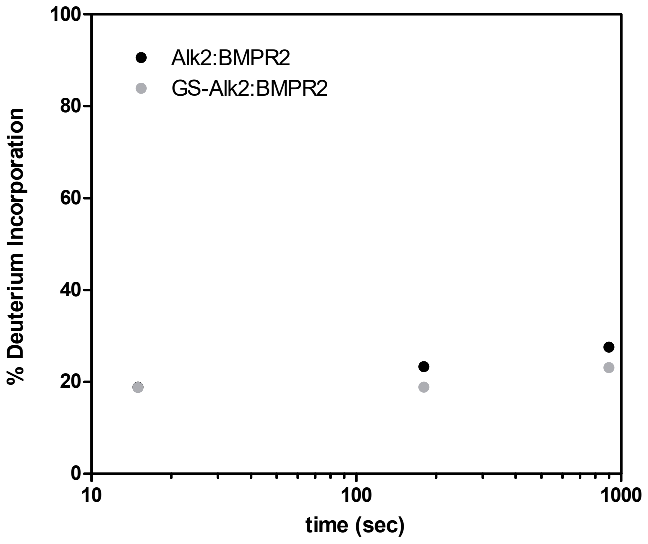

Alk2:BMPR2 complex vs. GS-Alk2:BMPR2 complex - HDX-MS Uptake Plots

Alk2 358-368 +2

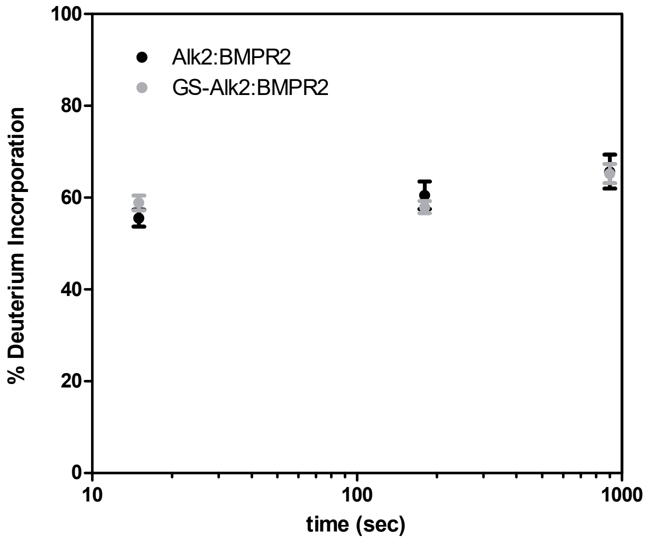

Alk2 358-387 +2

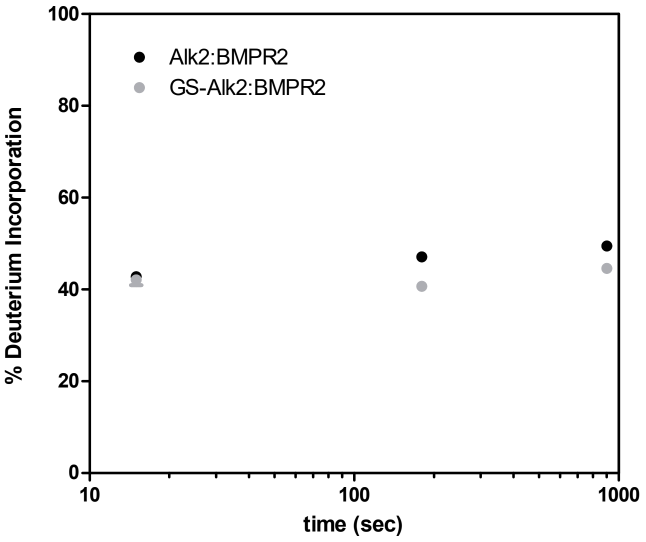

Alk2 358-389 +3

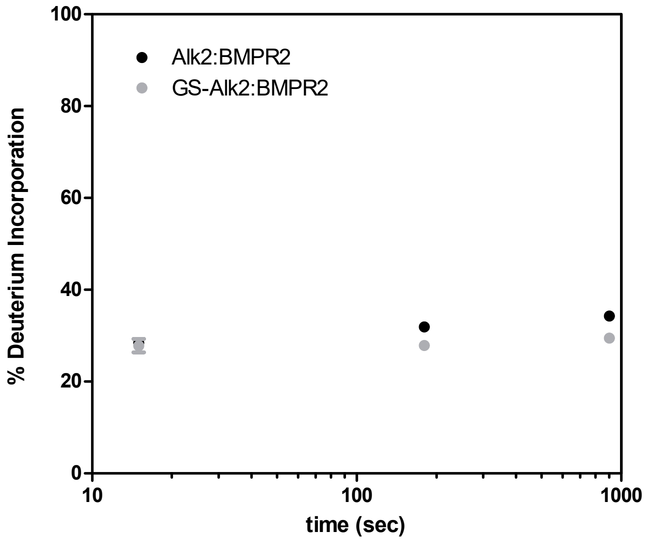

Alk2 369-387 +3

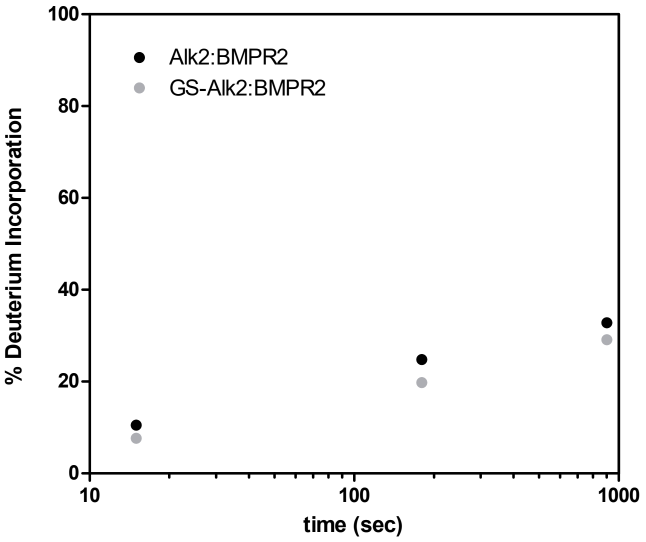

Alk2 395-406 +2

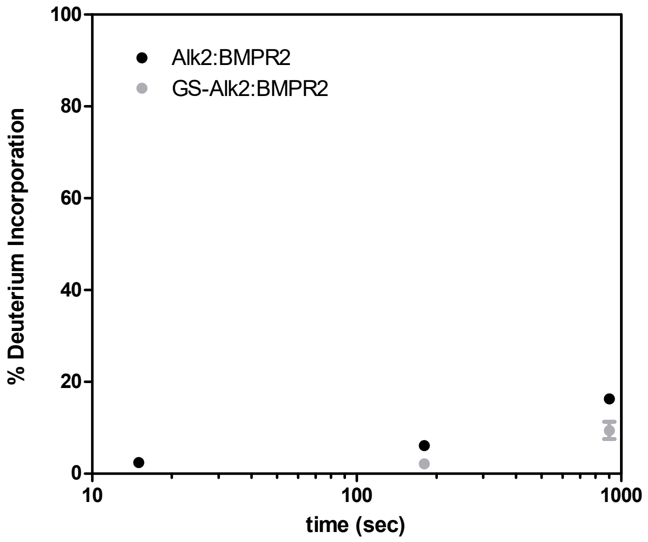

Alk2 396-406 +2

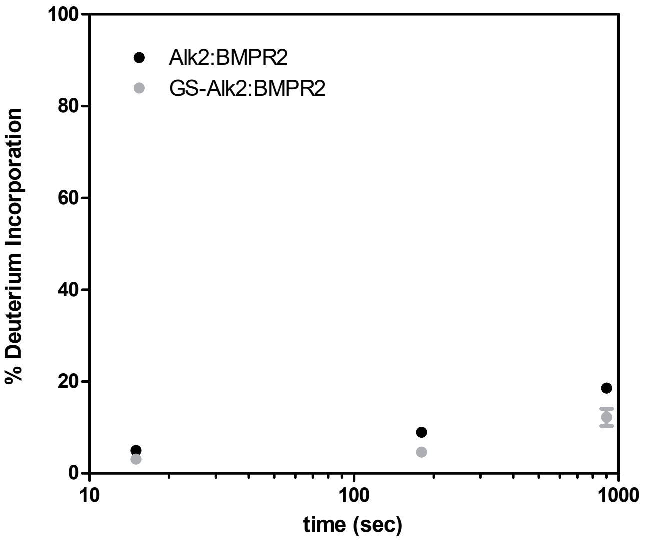

Alk2:BMPR2 complex vs. GS-Alk2:BMPR2 complex - HDX-MS Uptake Plots

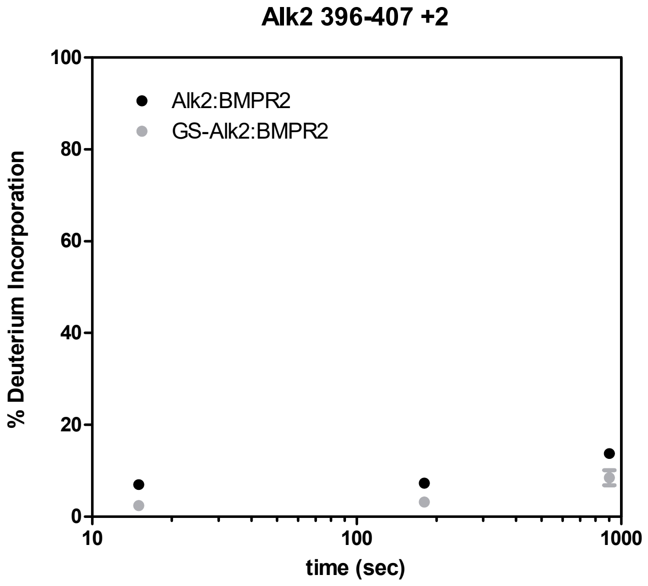

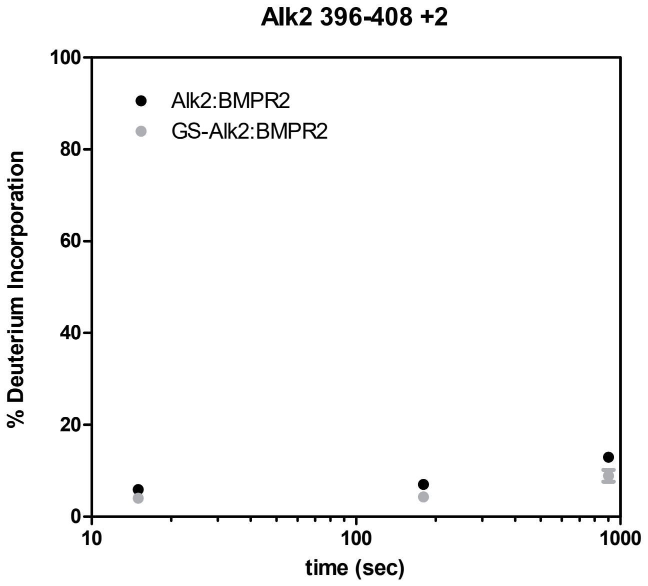

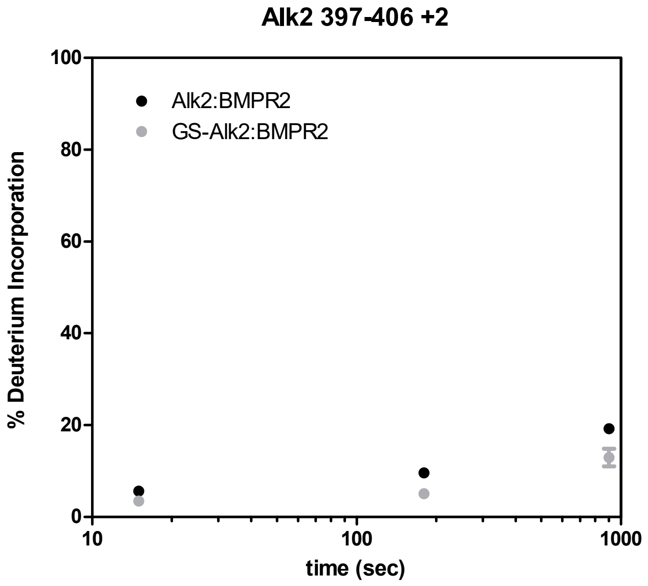

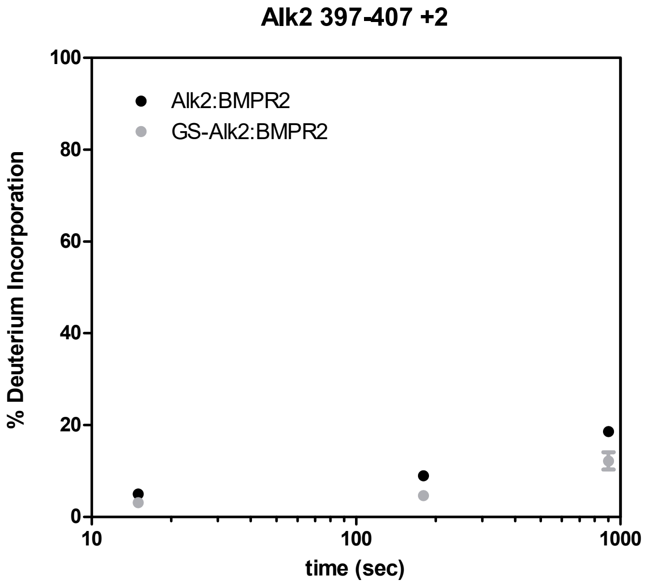

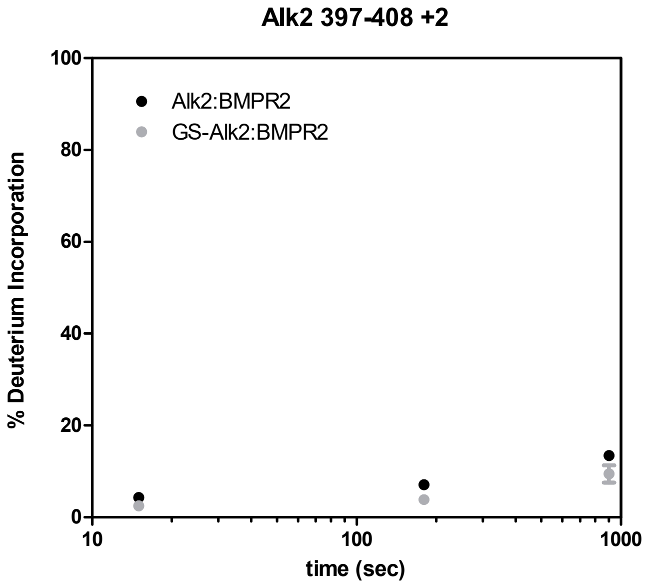

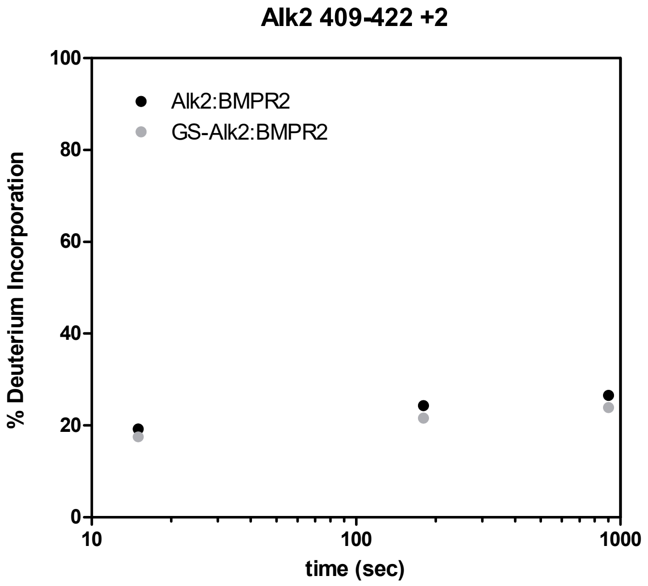

Alk2:BMPR2 complex vs. GS-Alk2:BMPR2 complex - HDX-MS Uptake Plots

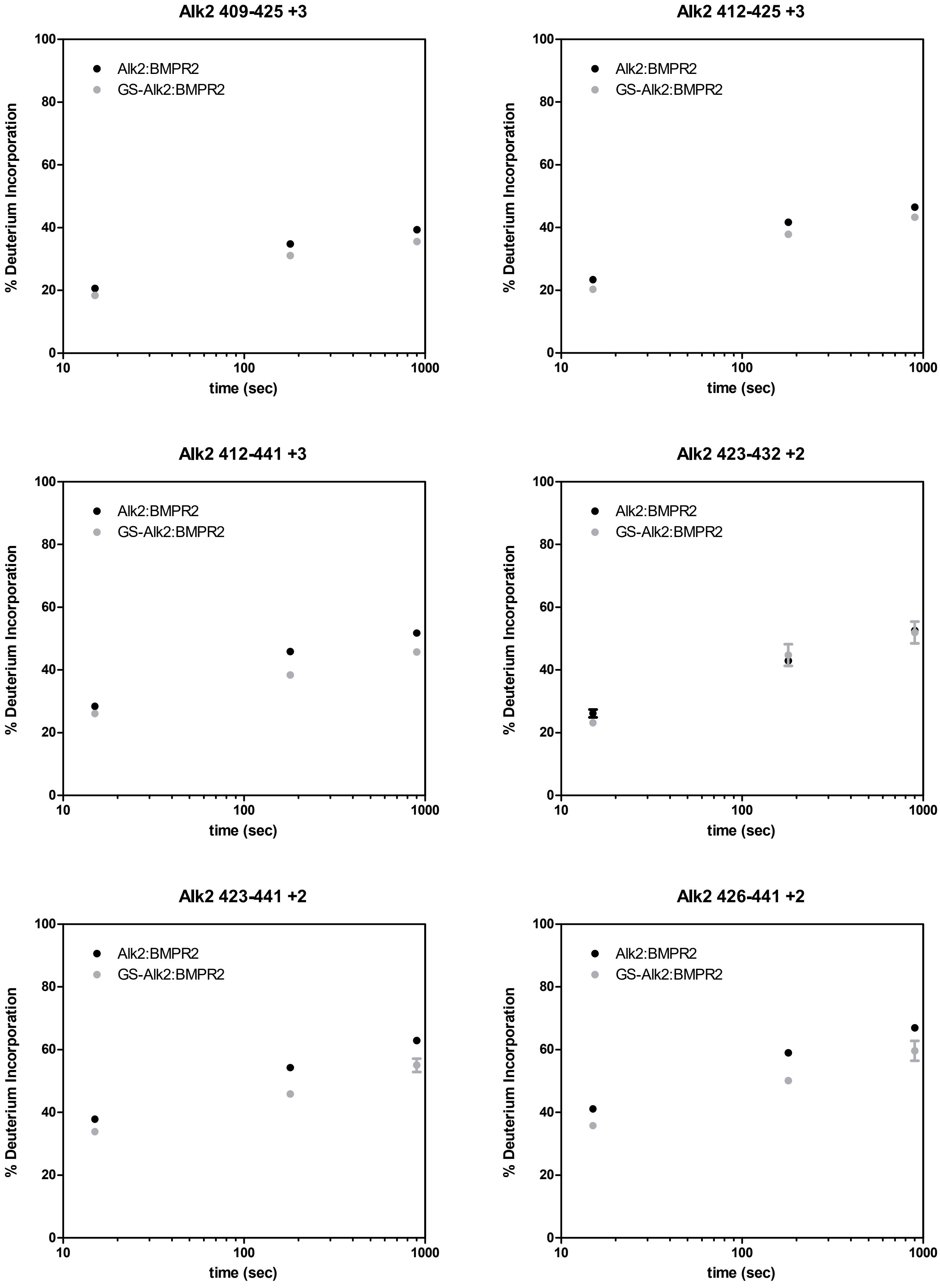

Alk2:BMPR2 complex vs. GS-Alk2:BMPR2 complex - HDX-MS Uptake Plots

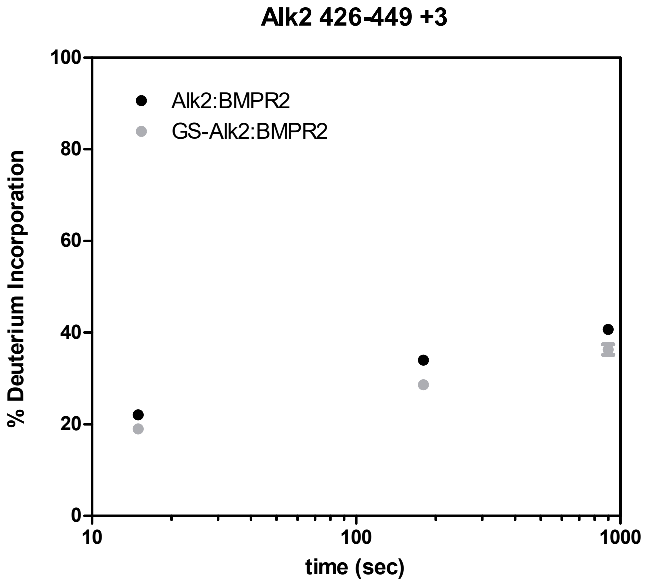

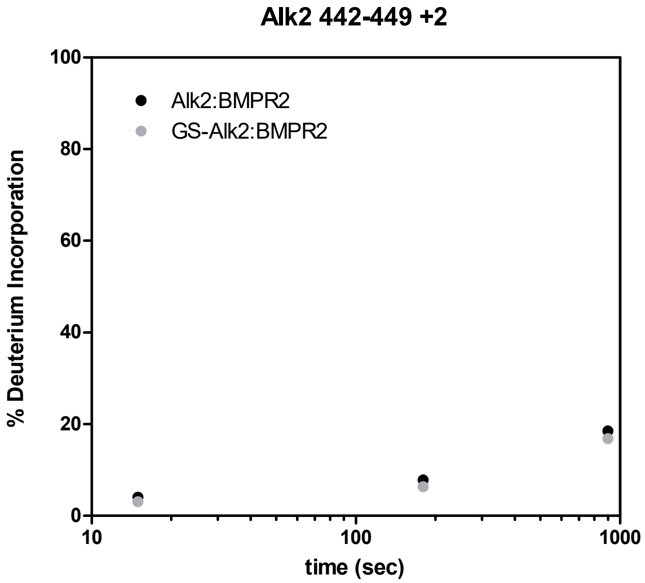

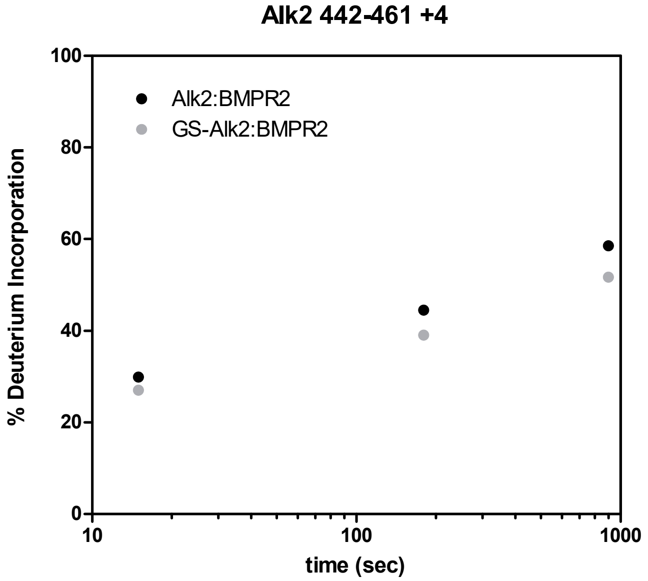

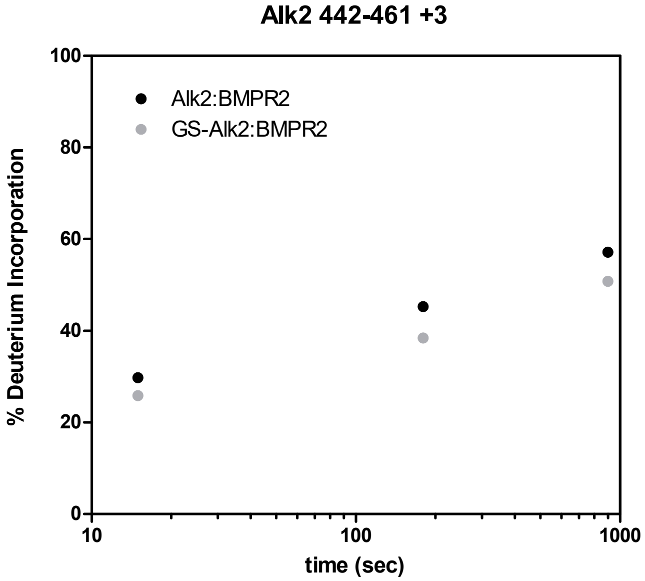

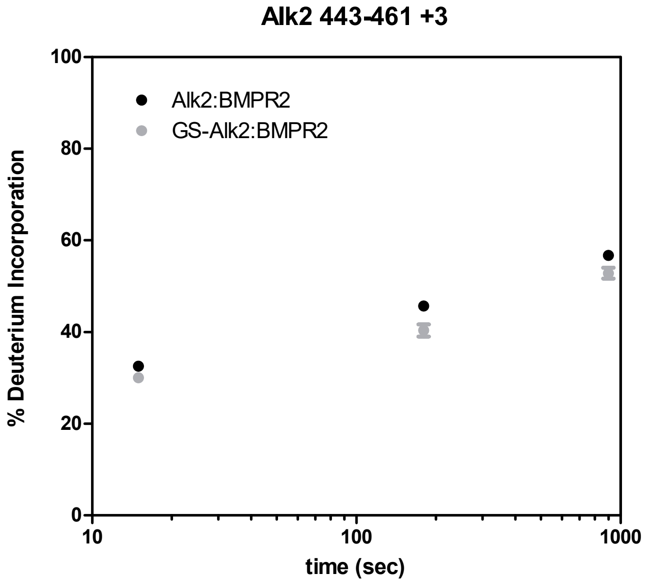

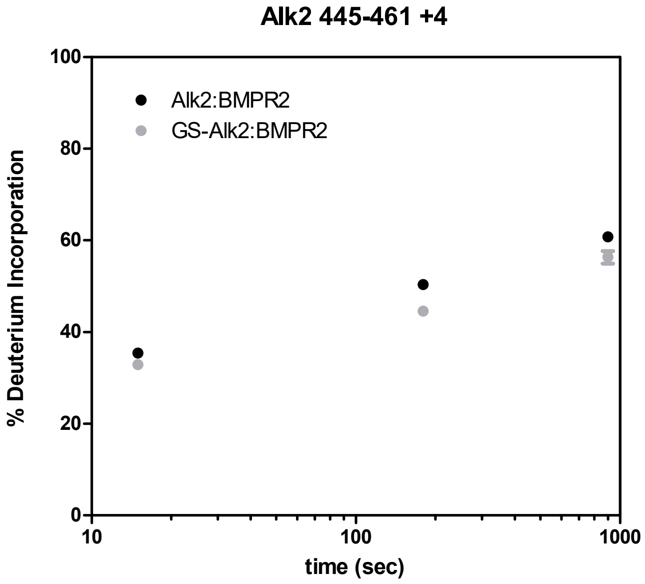

Alk2:BMPR2 complex vs. GS-Alk2:BMPR2 complex - HDX-MS Uptake Plots

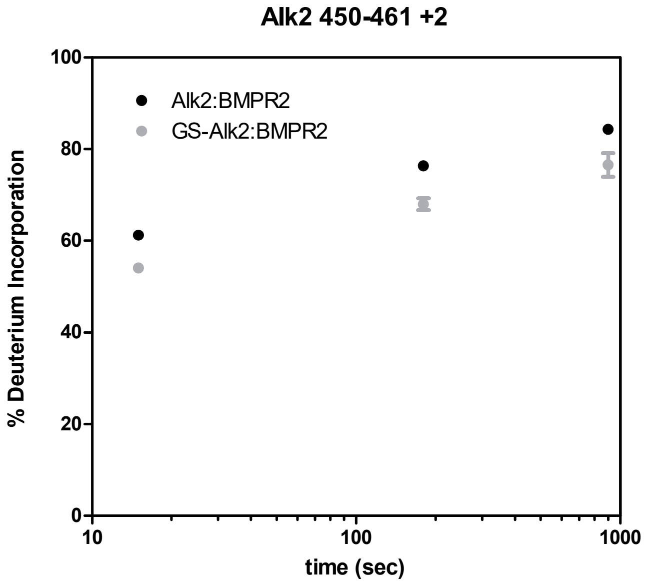

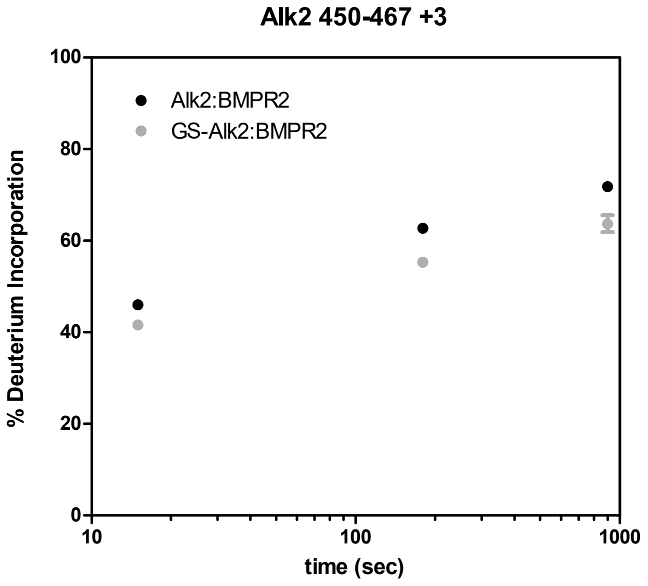

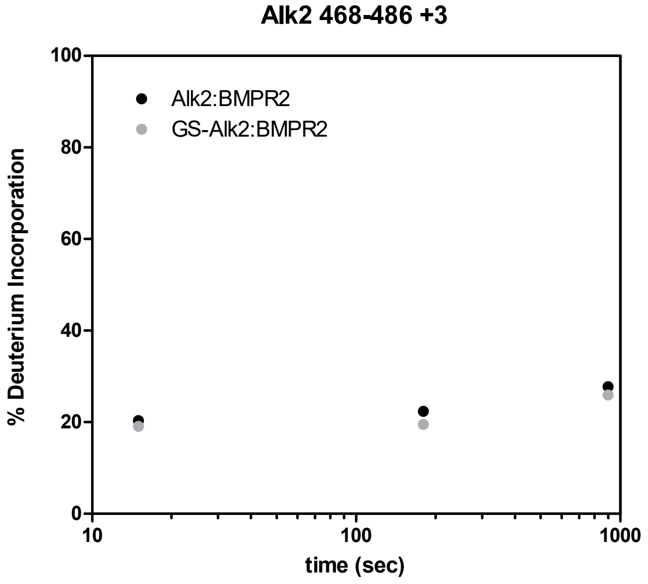

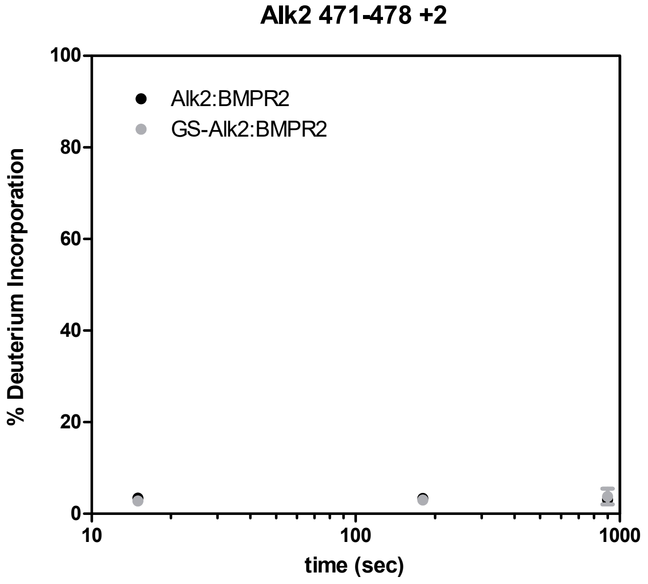

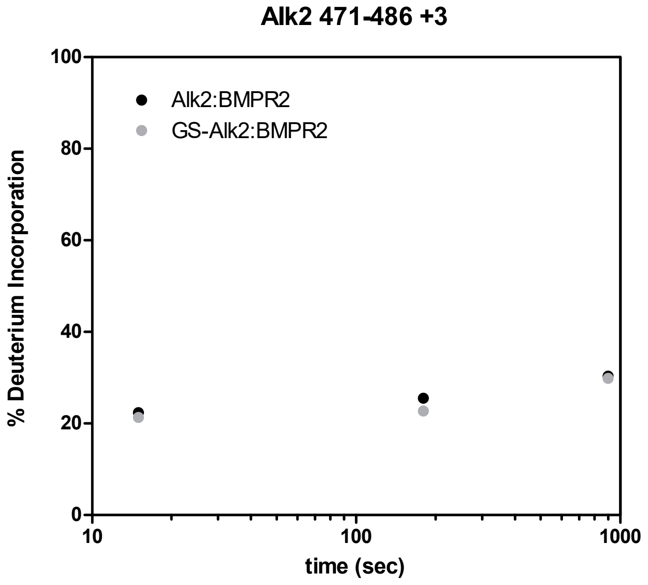

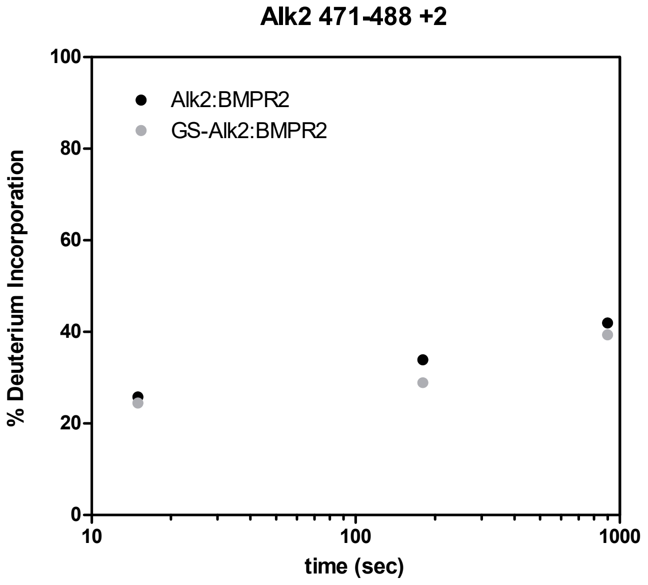

Alk2:BMPR2 complex vs. GS-Alk2:BMPR2 complex - HDX-MS Uptake Plots

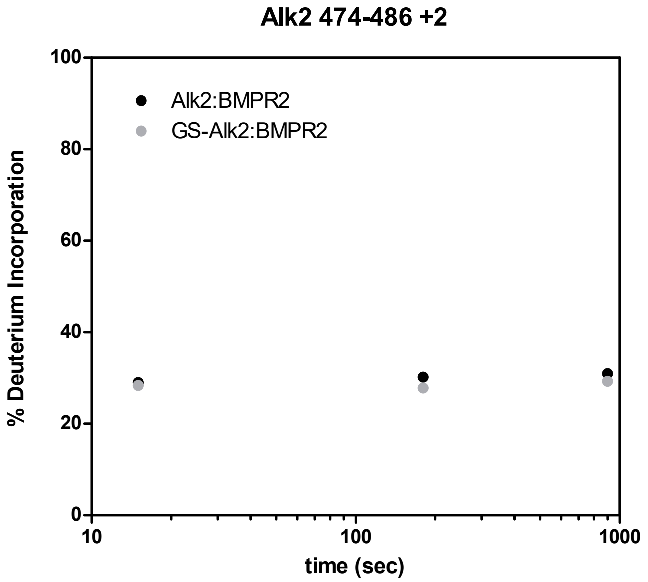

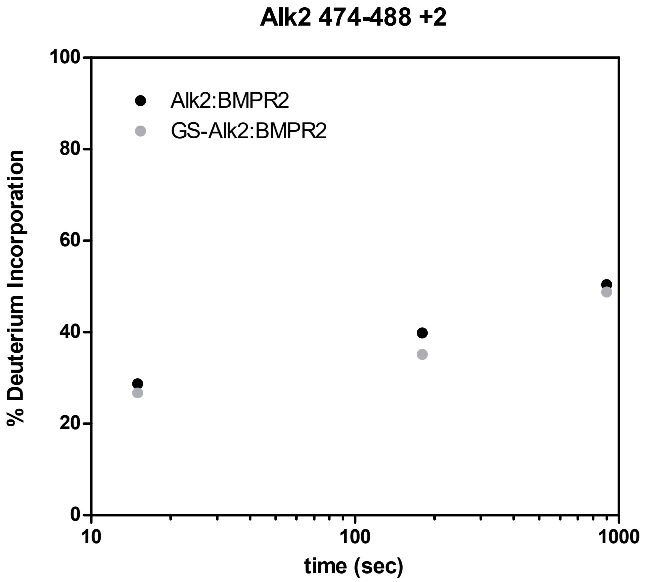

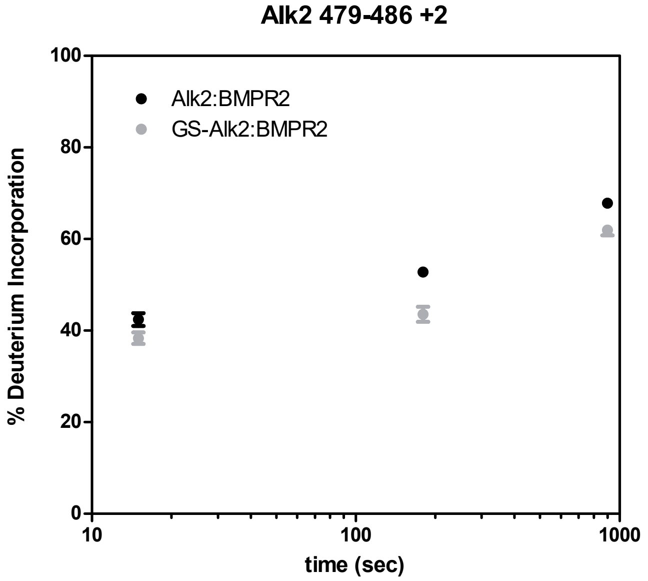

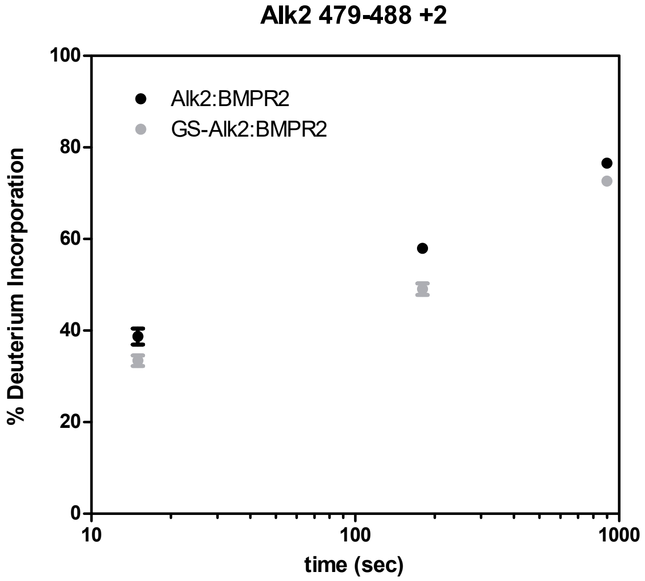

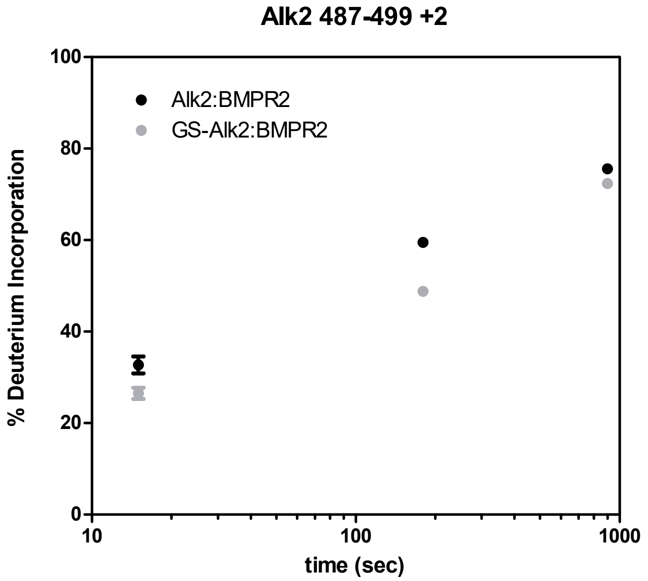

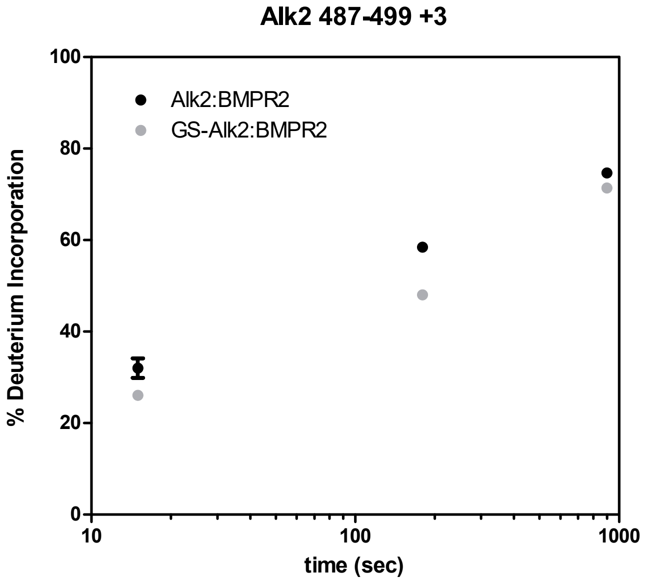

Alk2:BMPR2 complex vs. GS-Alk2:BMPR2 complex - HDX-MS Uptake Plots

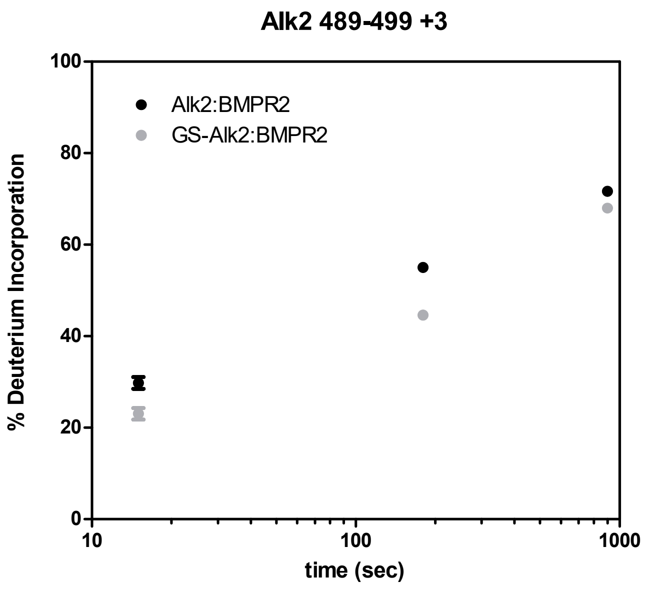

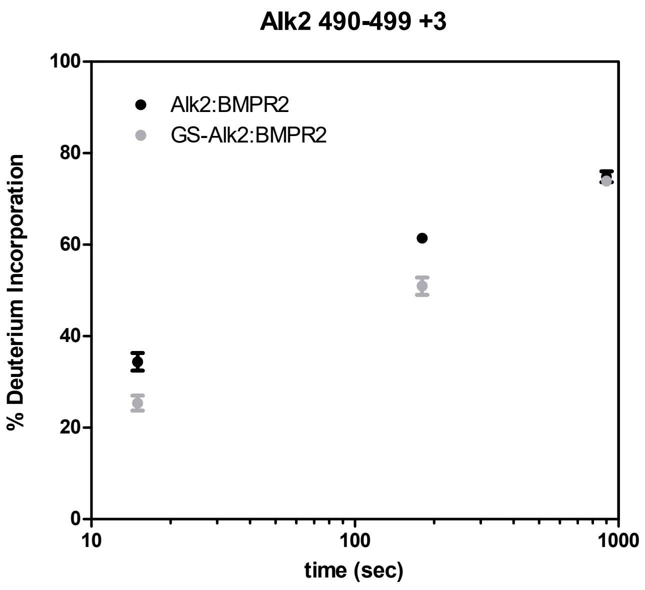

Alk2:BMPR2 complex vs. GS-Alk2-BMPR2 complex - HDX-MS Uptake Plots

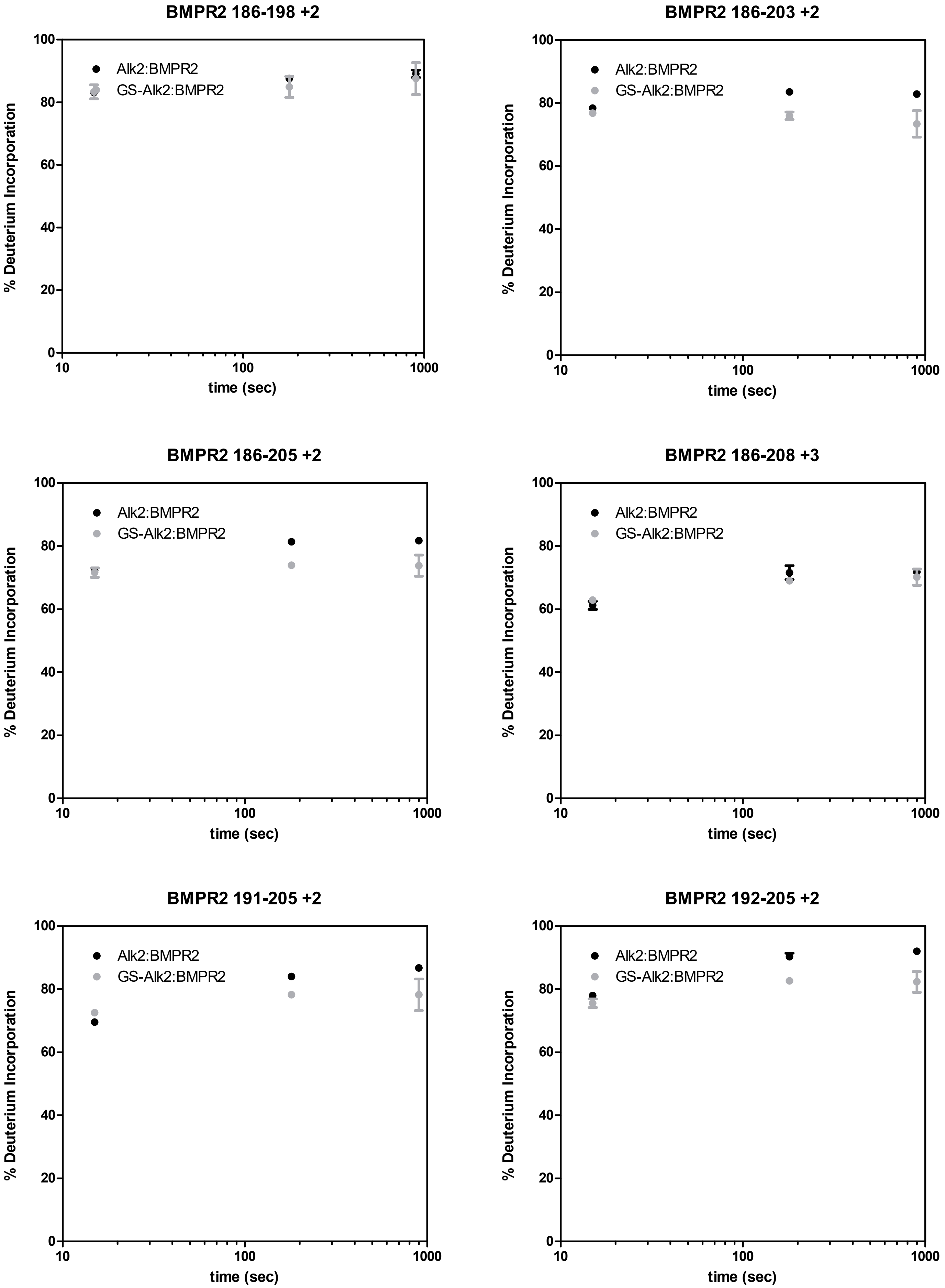

Alk2:BMPR2 complex vs. GS-Alk2-BMPR2 complex - HDX-MS Uptake Plots

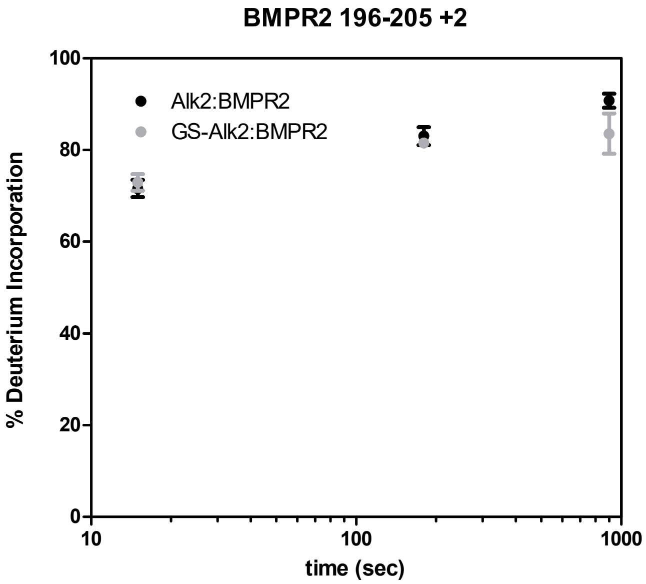

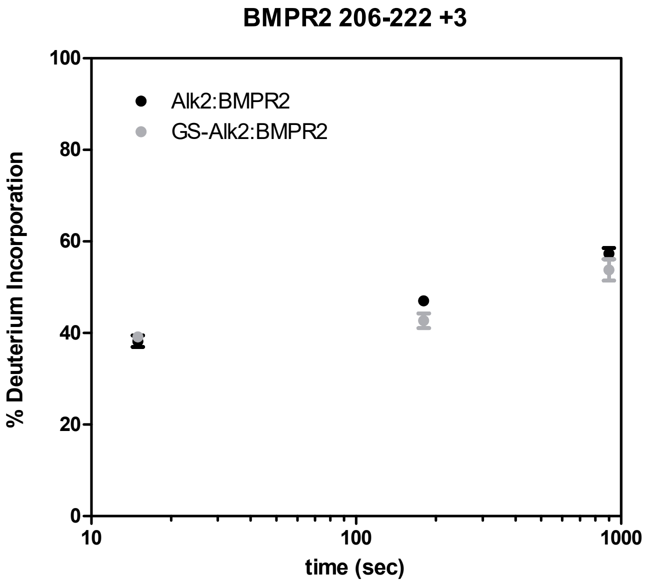

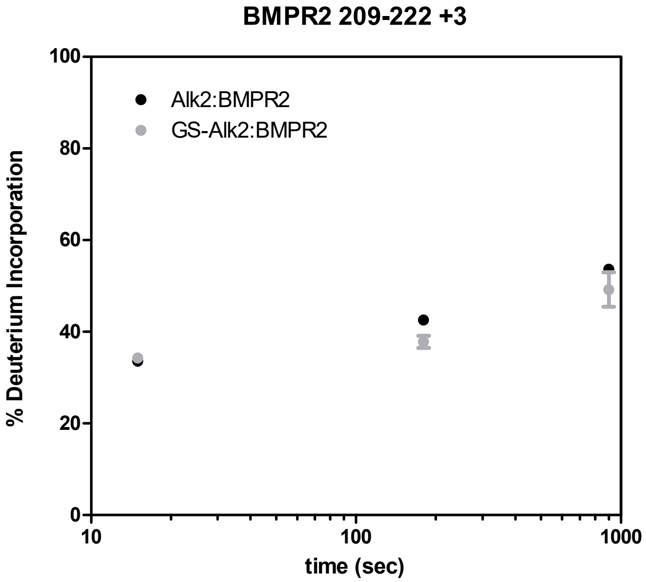

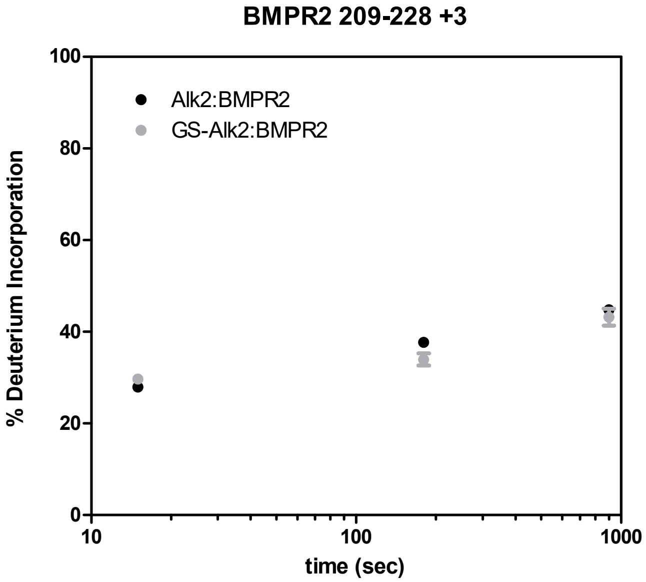

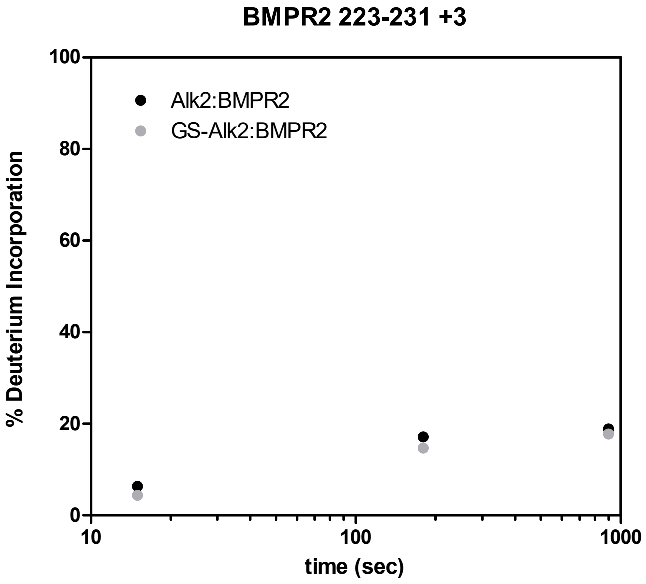

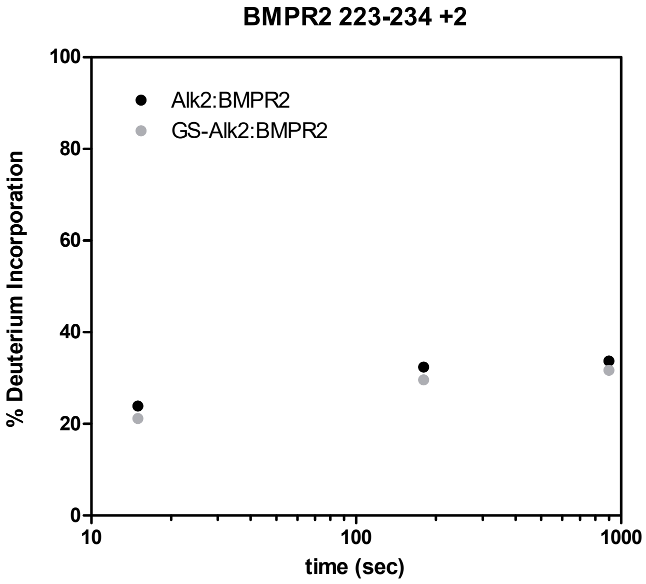

Alk2:BMPR2 complex vs. GS-Alk2-BMPR2 complex - HDX-MS Uptake Plots

BMPR2 223-243 +2

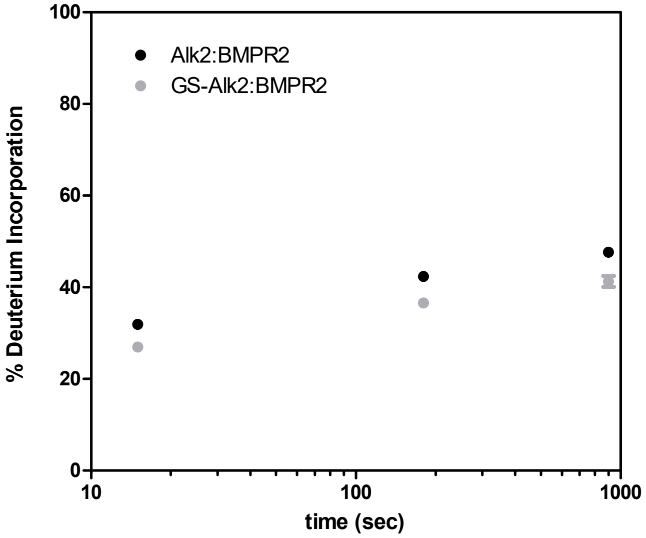

BMPR2 232-243 +2

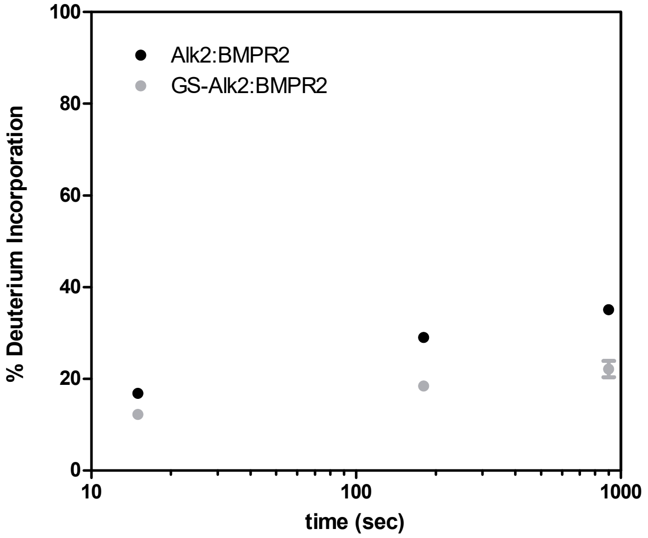

BMPR2 234-260 +5

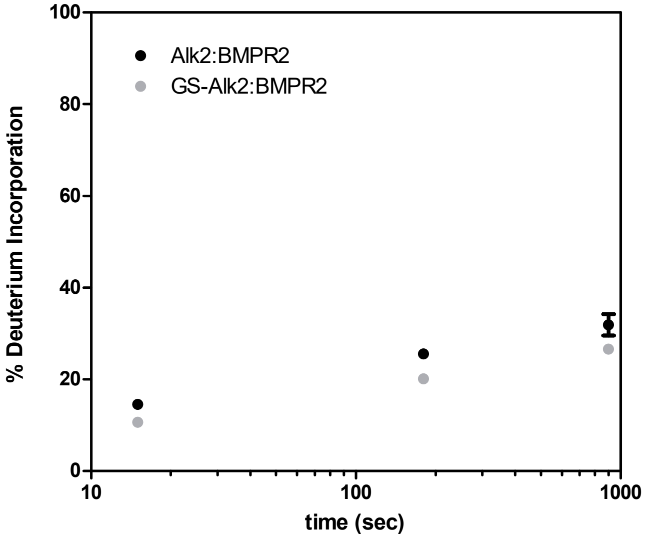

BMPR2 235-243 +2

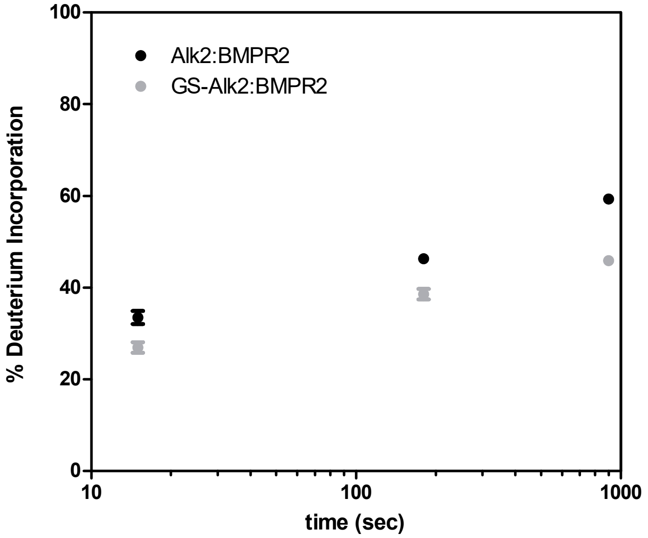

BMPR2 235-260 +5

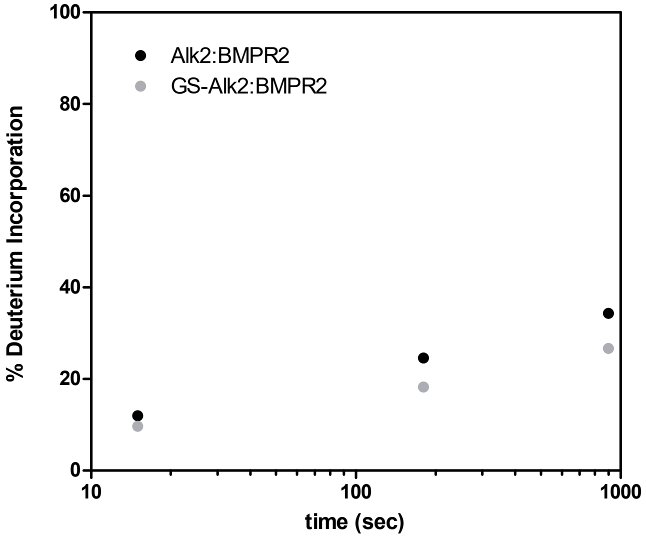

BMPR2 241-260 +3

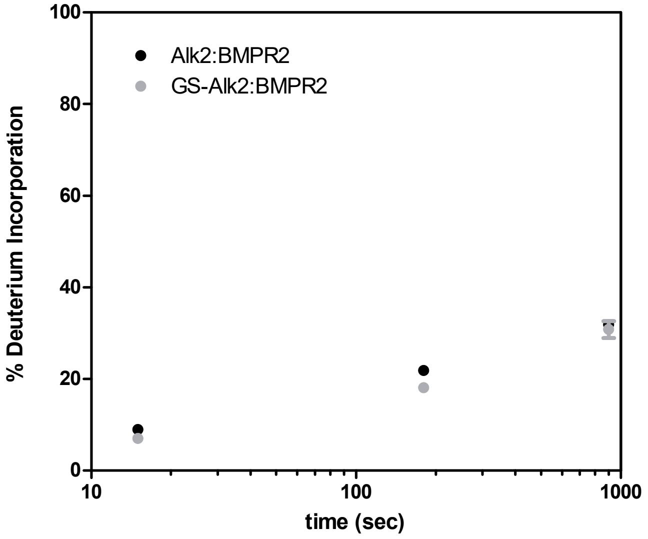

Alk2:BMPR2 complex vs. GS-Alk2-BMPR2 complex - HDX-MS Uptake Plots

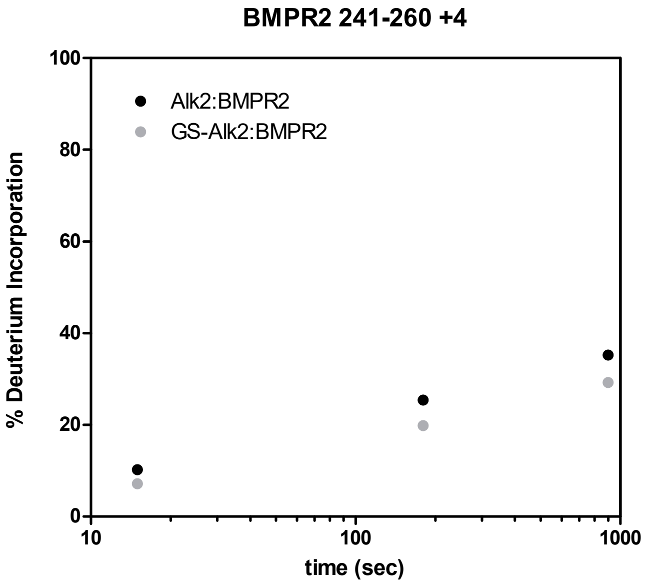

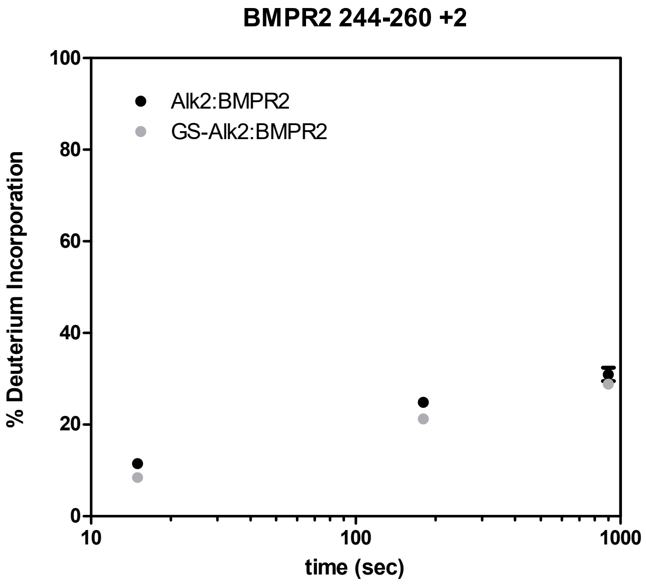

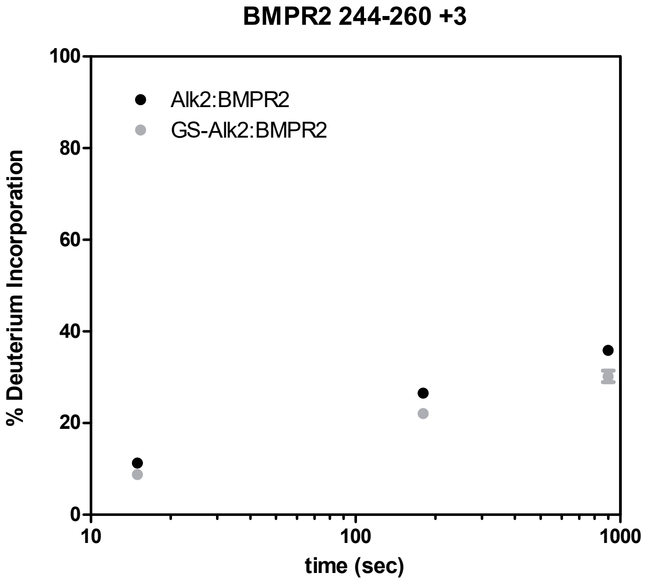

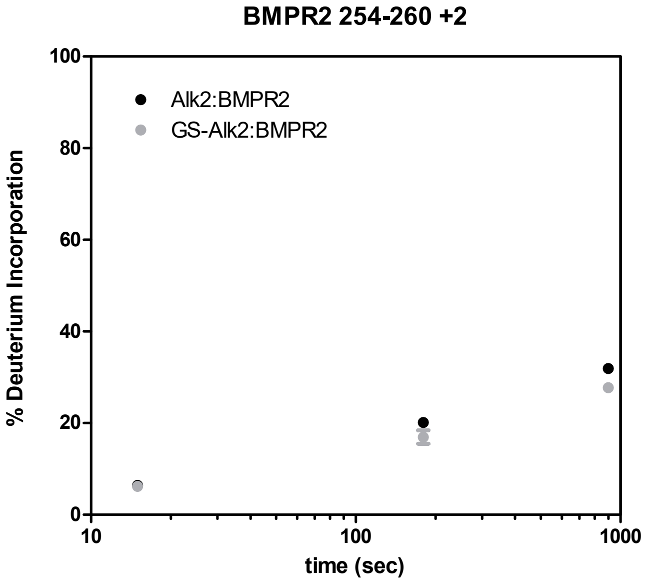

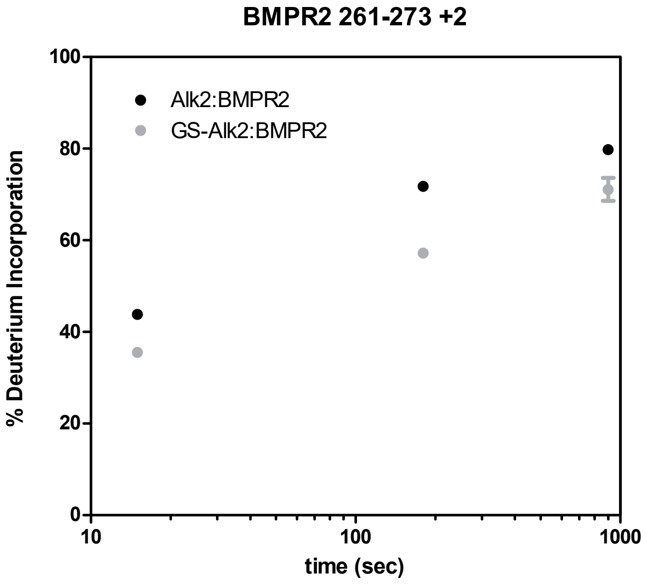

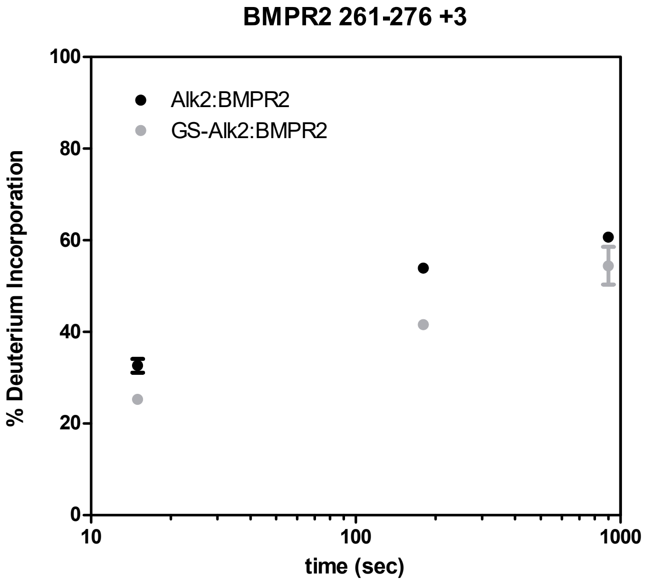

Alk2:BMPR2 complex vs. GS-Alk2-BMPR2 complex - HDX-MS Uptake Plots

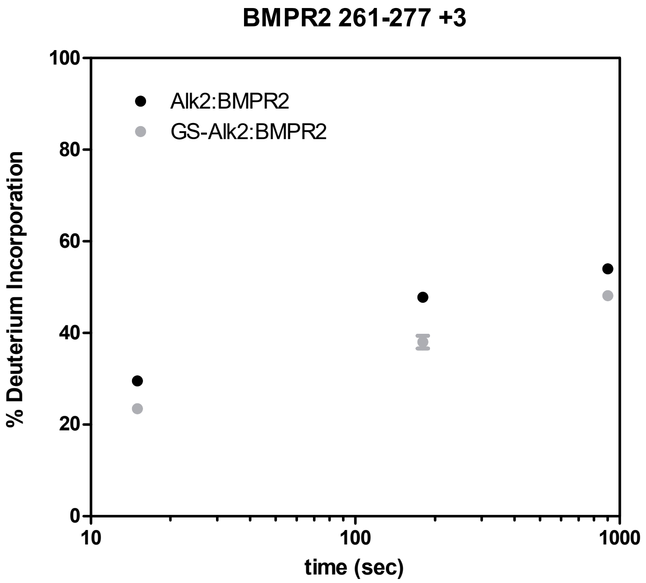

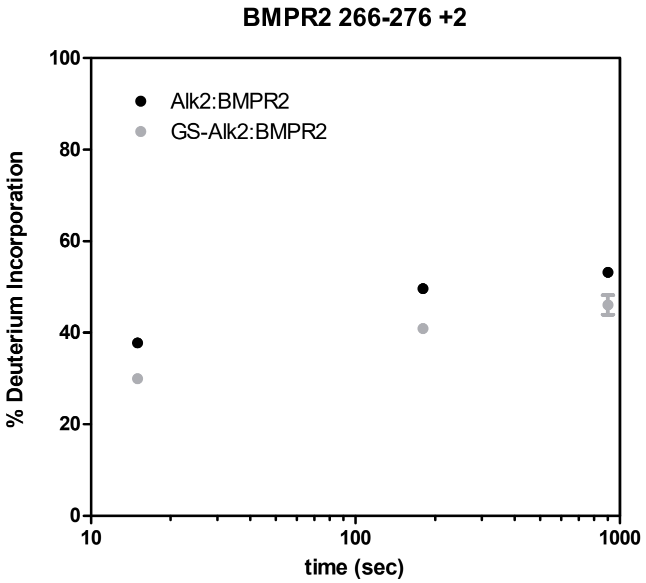

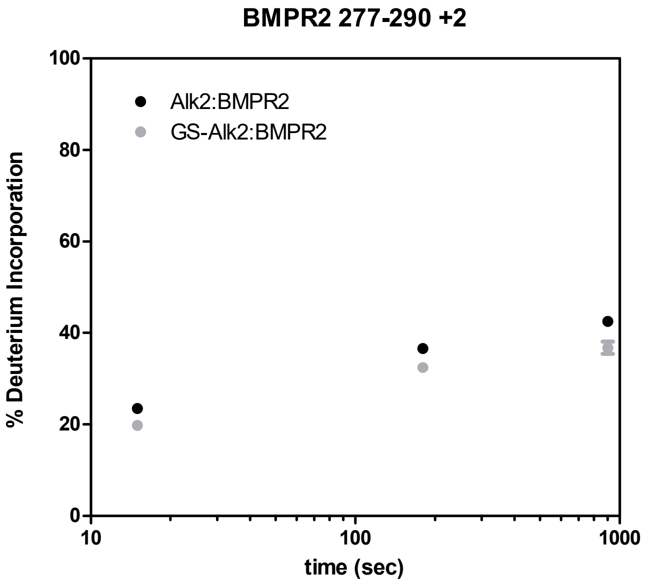

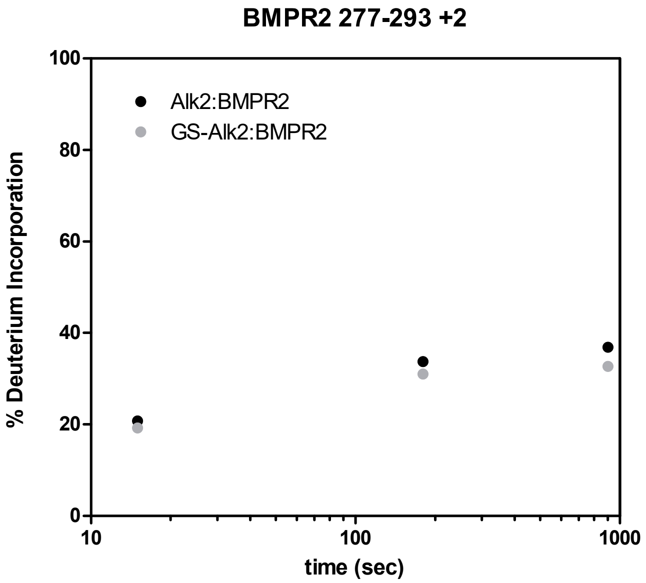

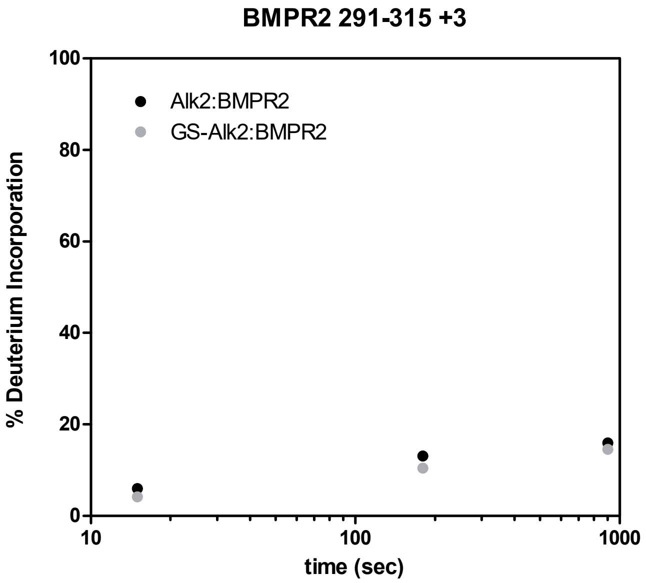

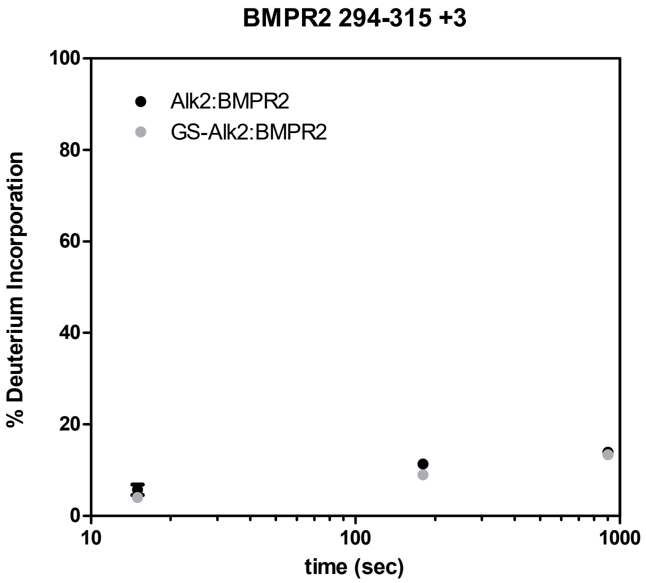

Alk2:BMPR2 complex vs. GS-Alk2-BMPR2 complex - HDX-MS Uptake Plots

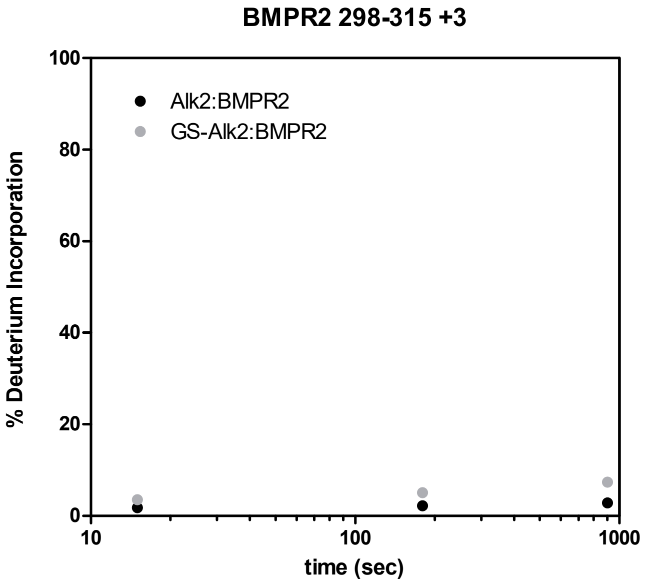

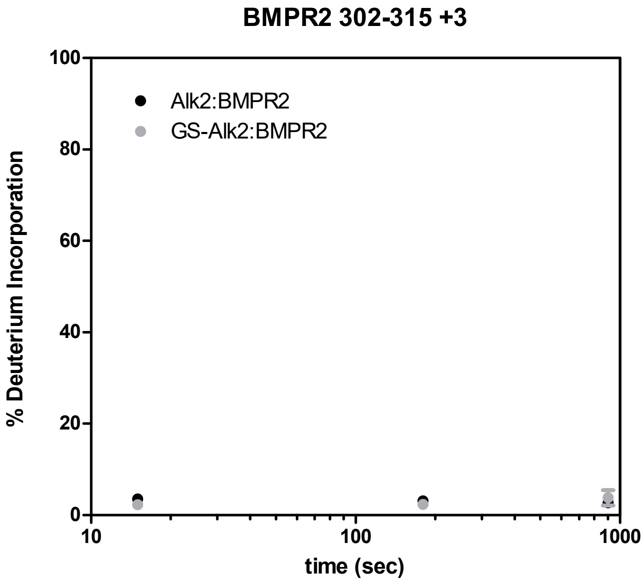

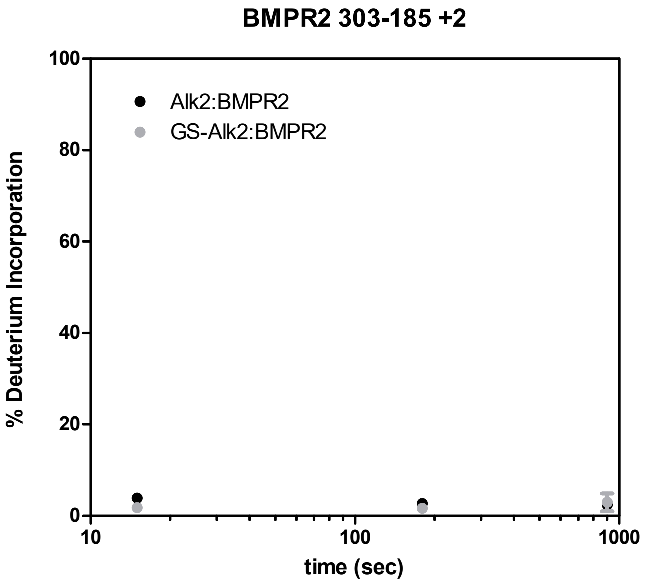

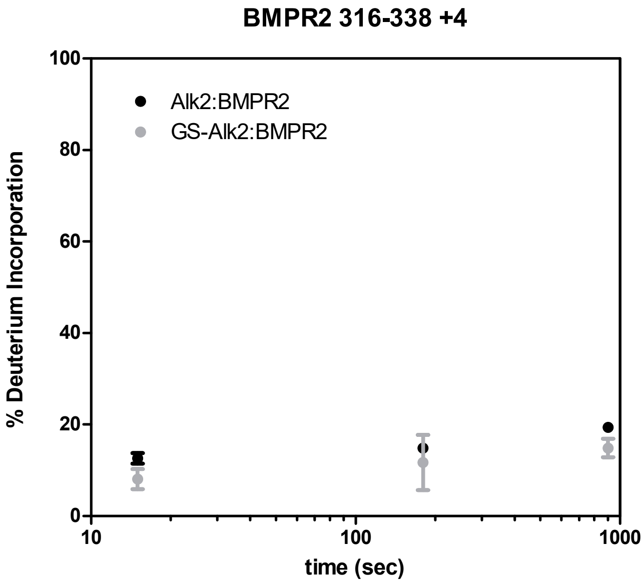

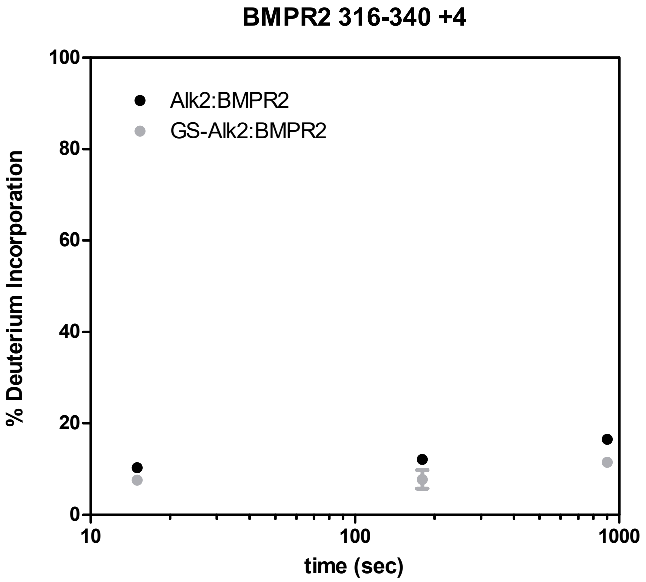

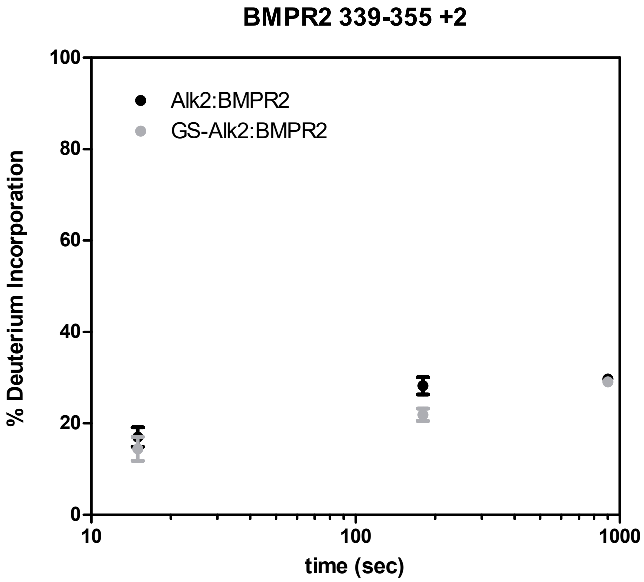

Alk2:BMPR2 complex vs. GS-Alk2-BMPR2 complex - HDX-MS Uptake Plots

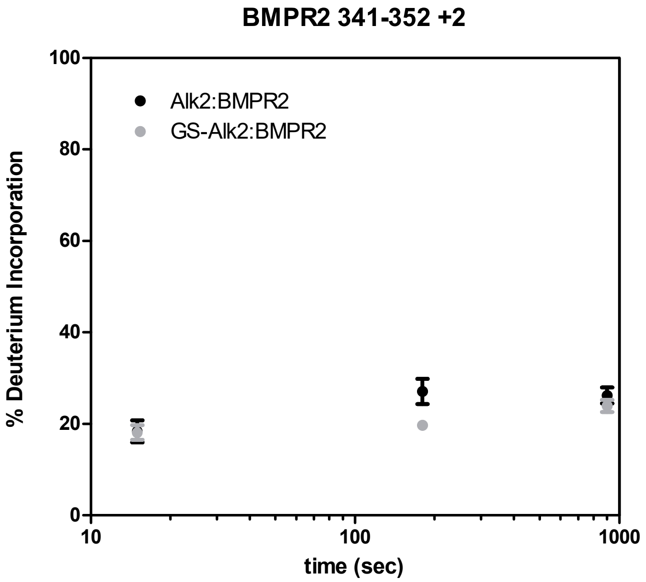

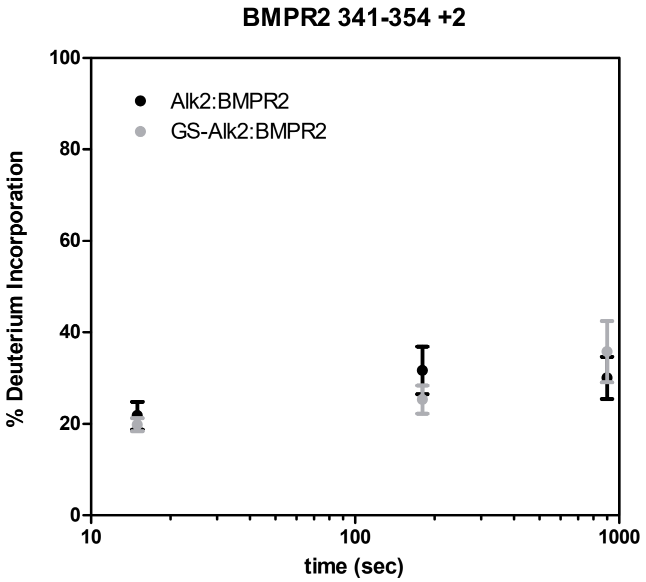

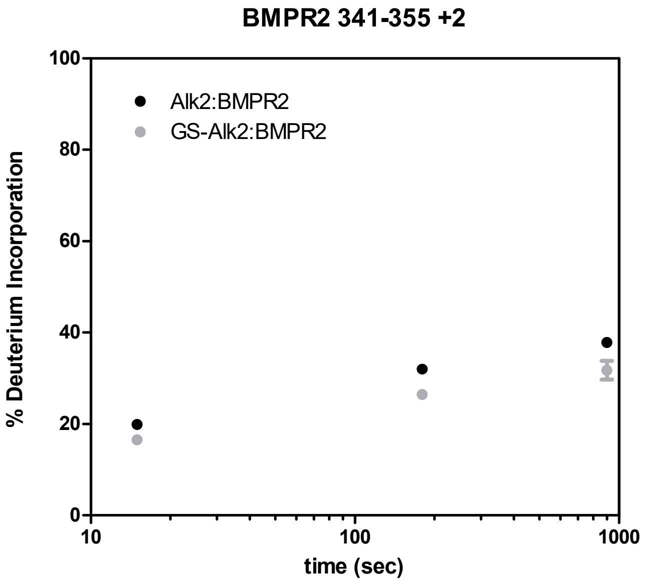

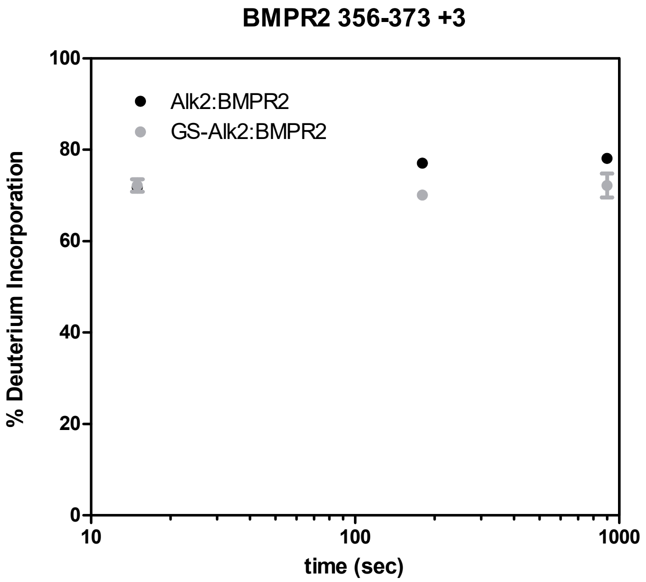

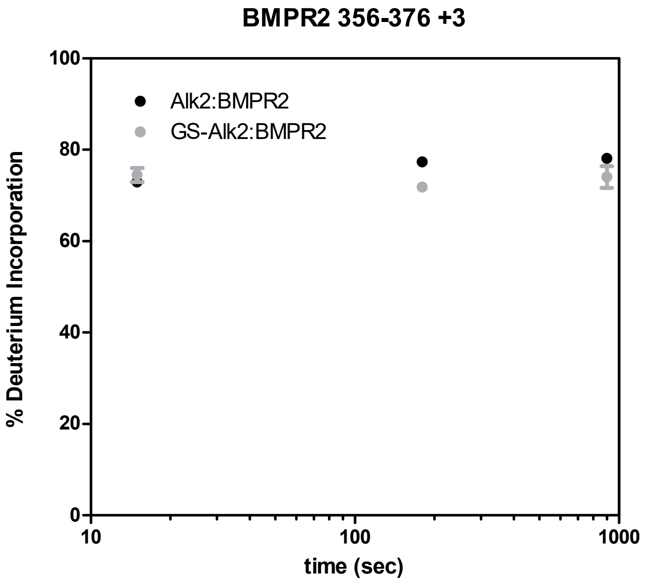

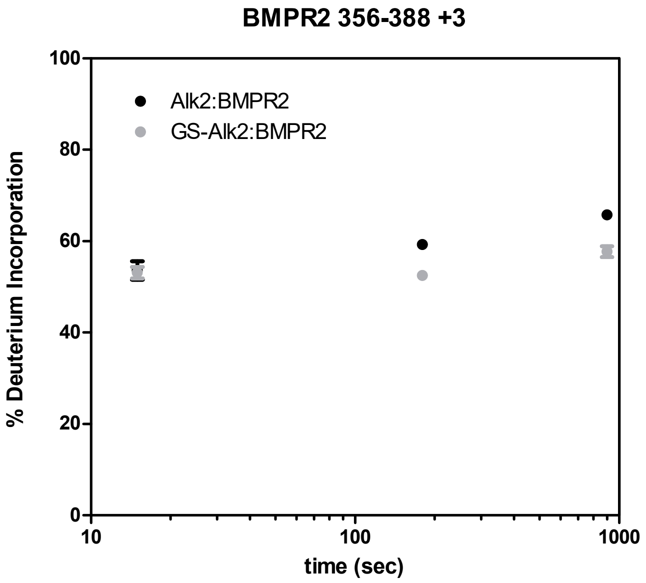

Alk2:BMPR2 complex vs. GS-Alk2-BMPR2 complex - HDX-MS Uptake Plots

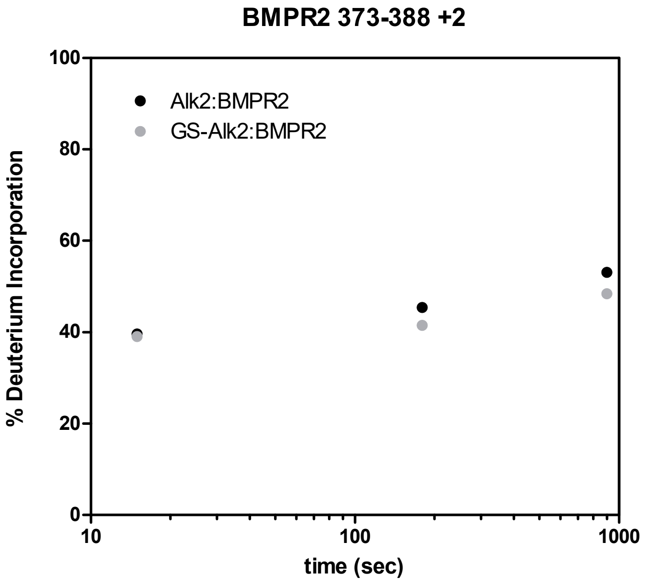

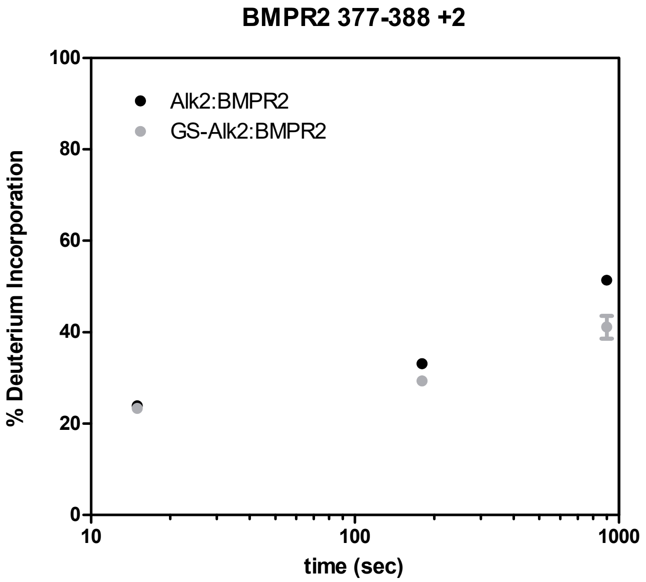

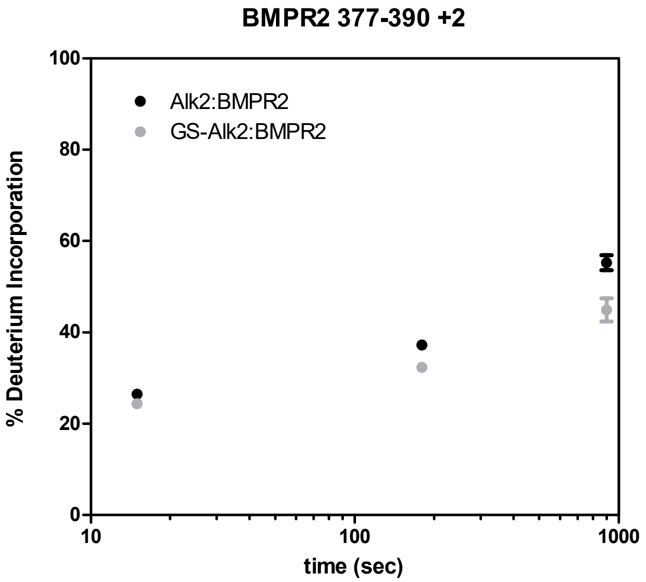

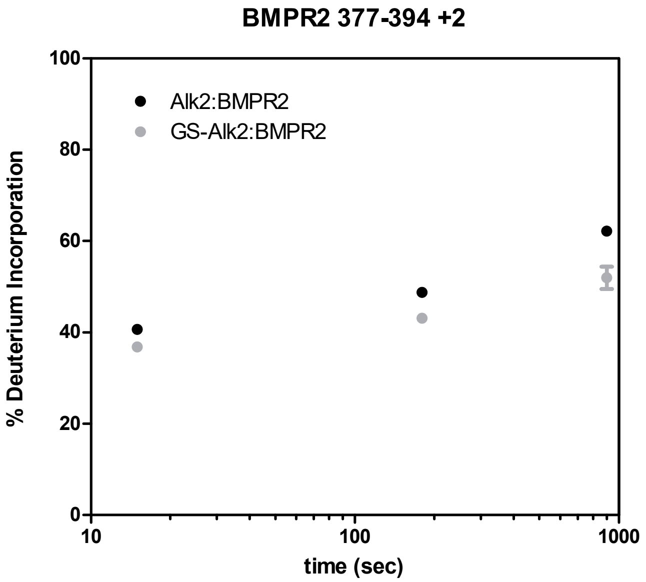

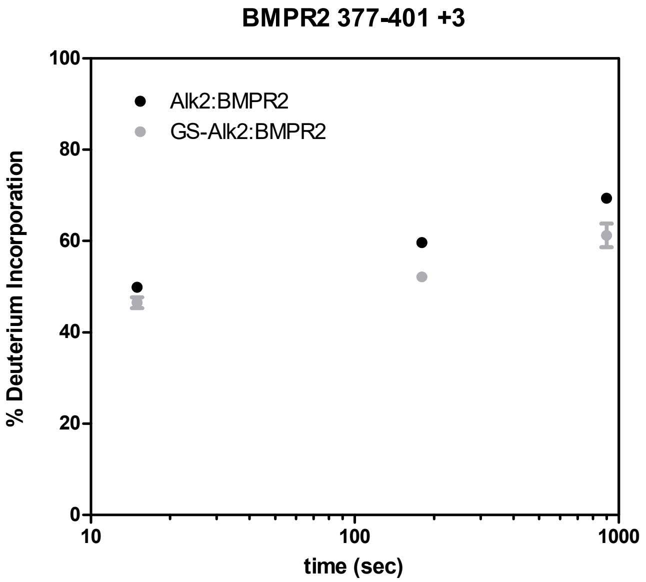

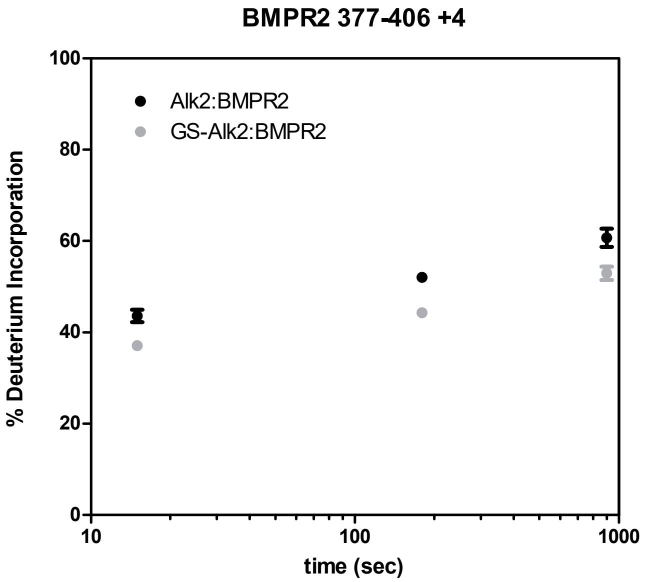

Alk2:BMPR2 complex vs. GS-Alk2-BMPR2 complex - HDX-MS Uptake Plots

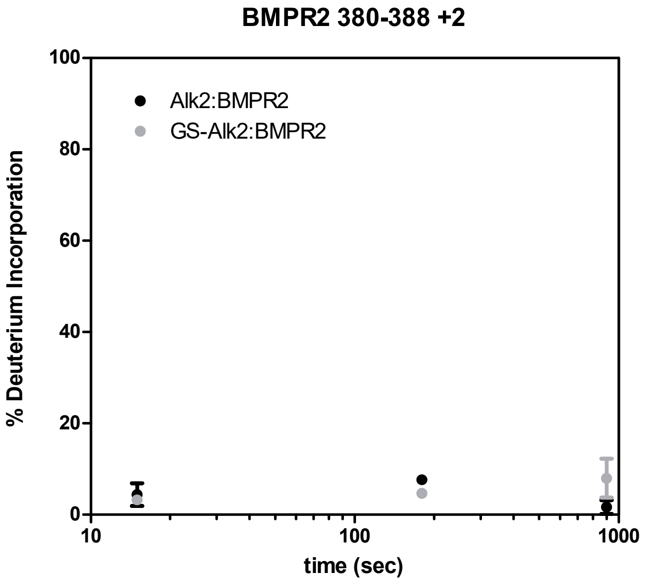

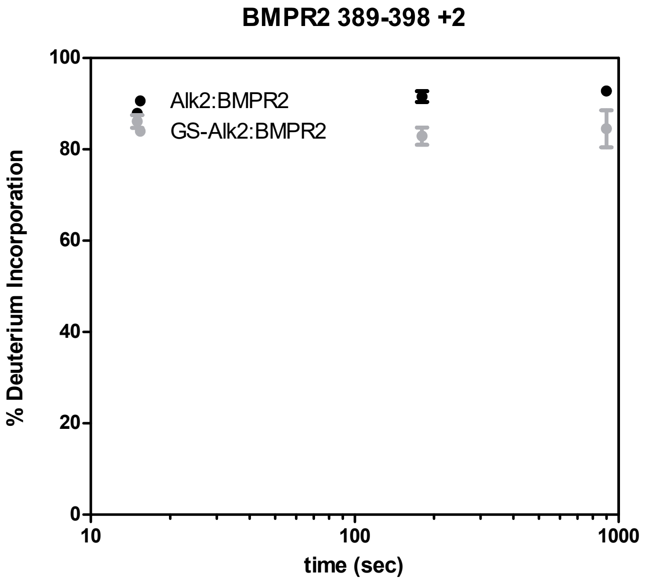

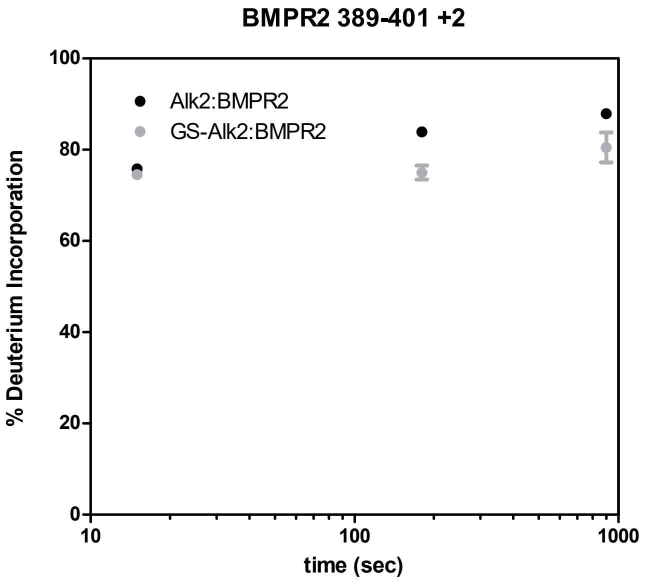

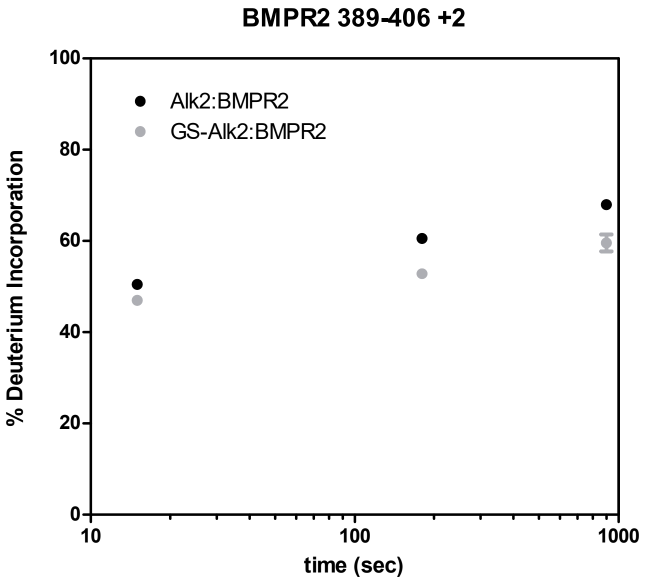

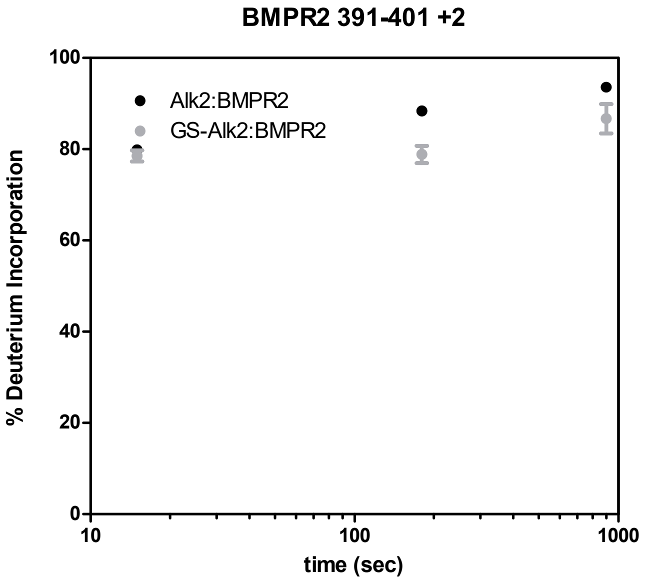

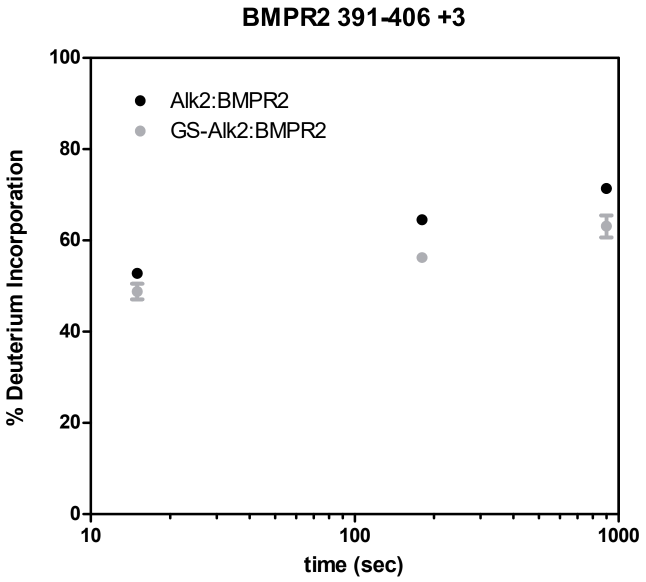

Alk2:BMPR2 complex vs. GS-Alk2-BMPR2 complex - HDX-MS Uptake Plots

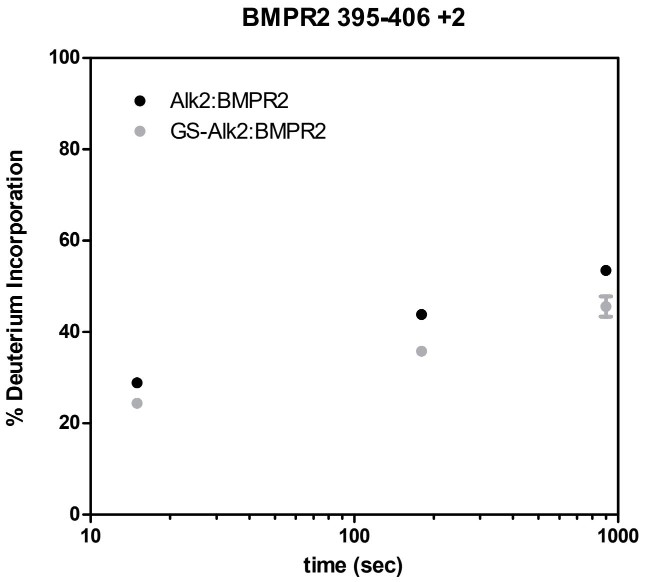

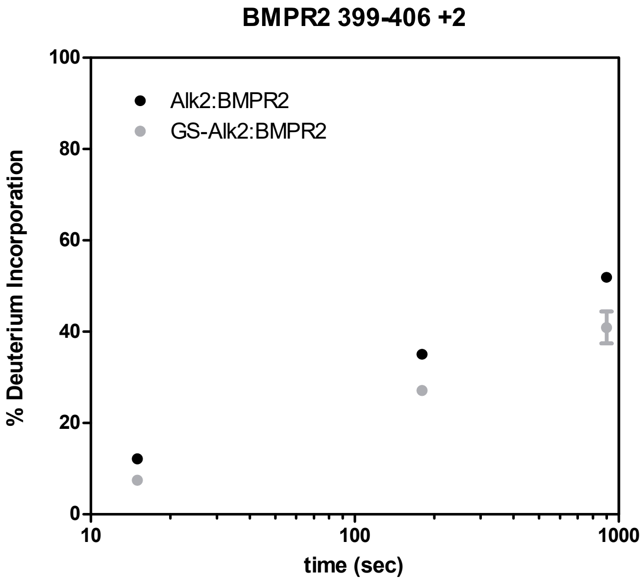

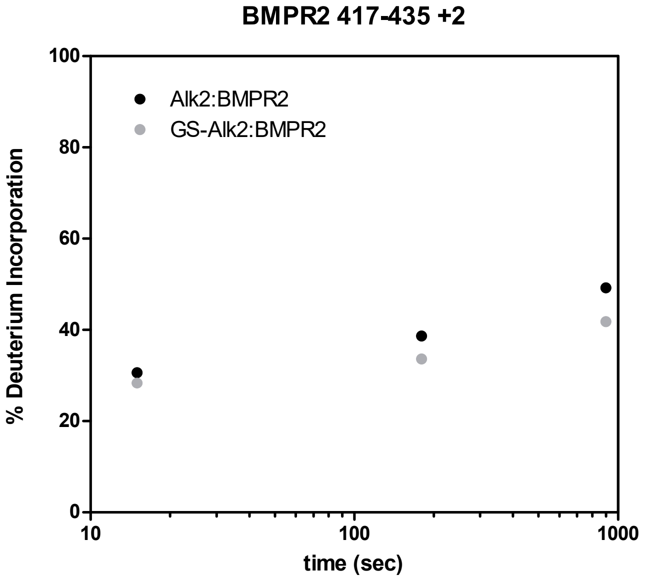

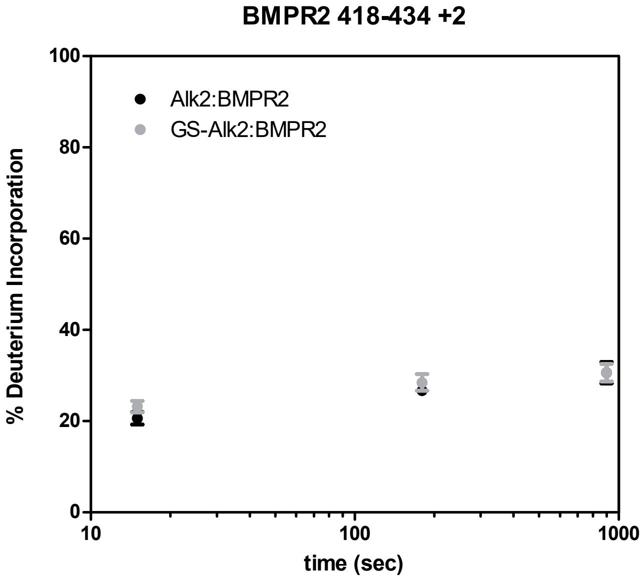

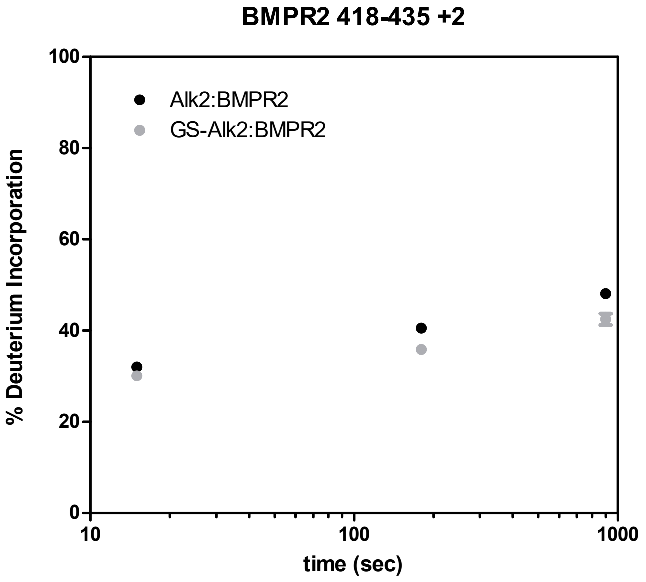

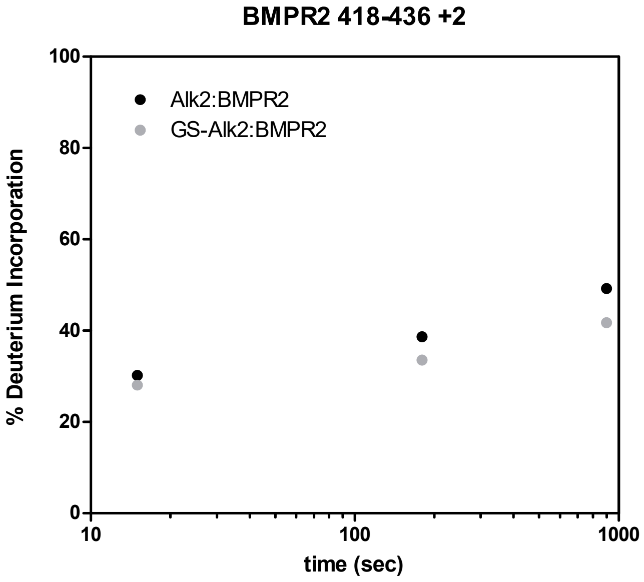

Alk2:BMPR2 complex vs. GS-Alk2-BMPR2 complex - HDX-MS Uptake Plots

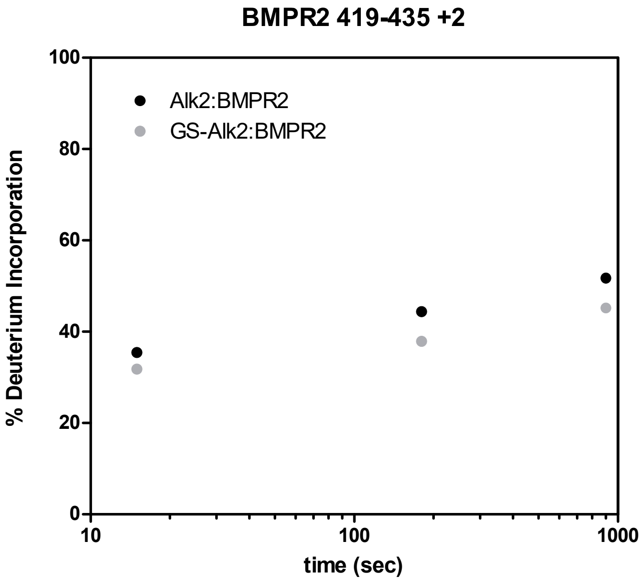

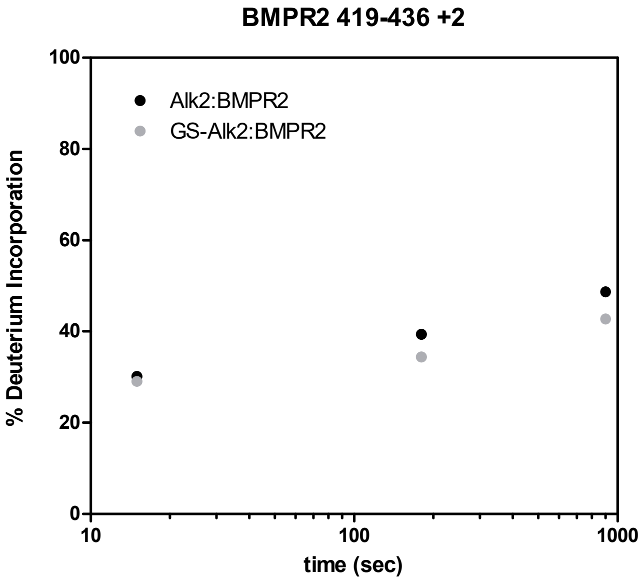

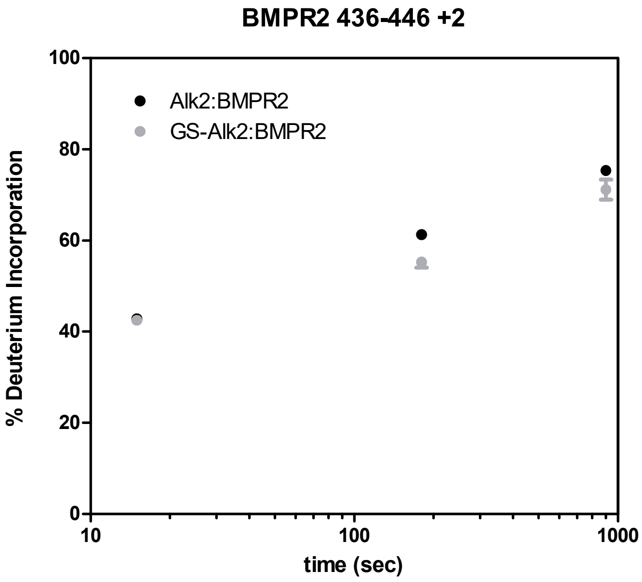

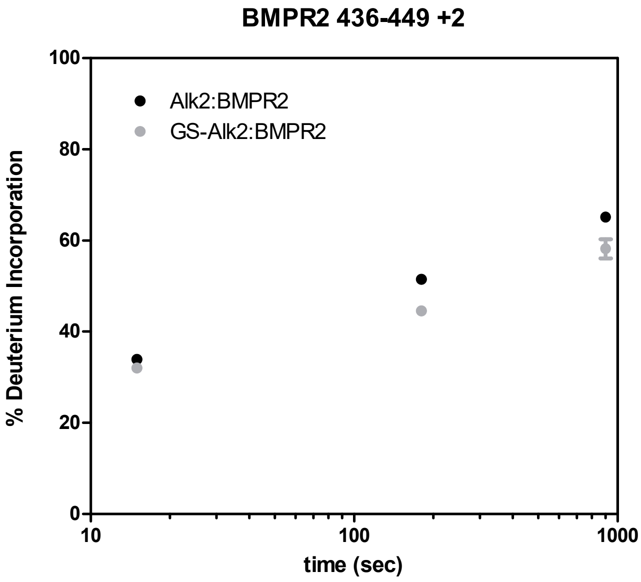

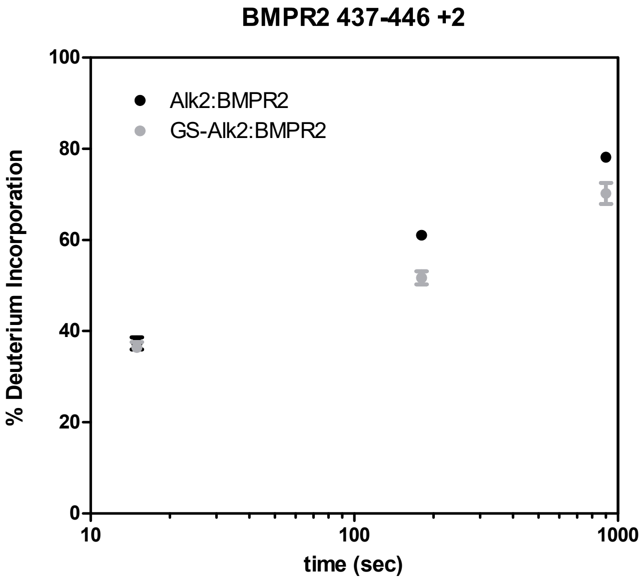

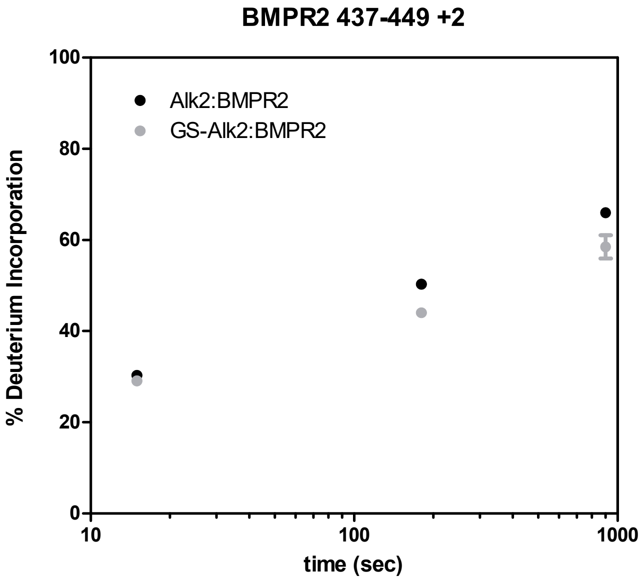

Alk2:BMPR2 complex vs. GS-Alk2-BMPR2 complex - HDX-MS Uptake Plots

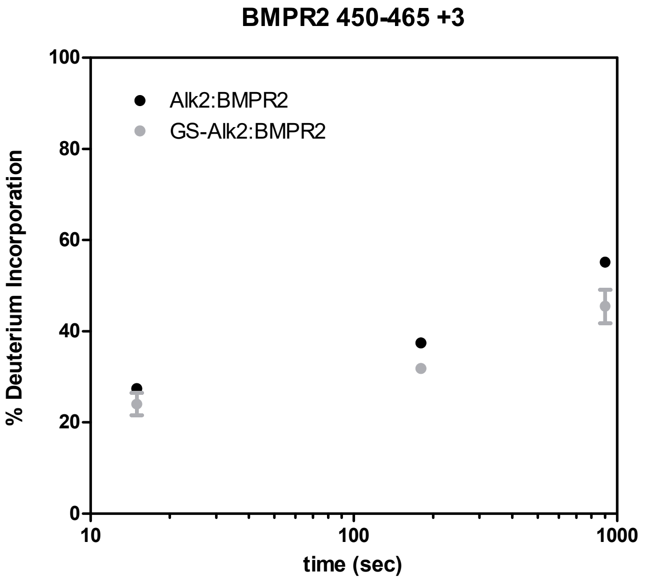

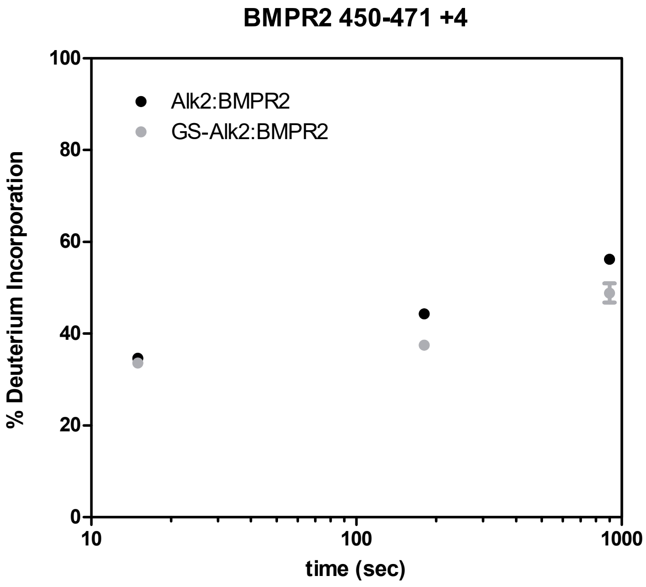

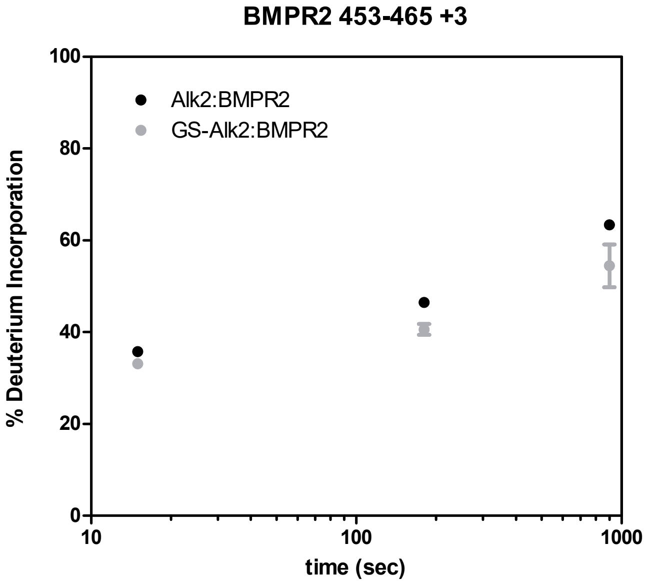

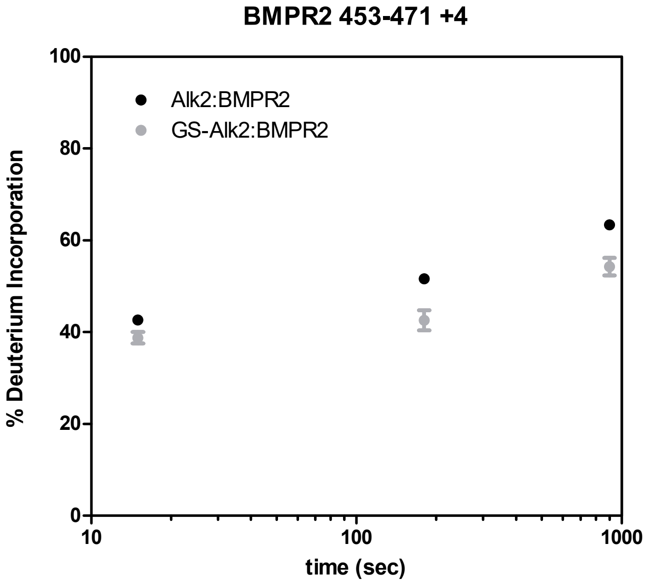

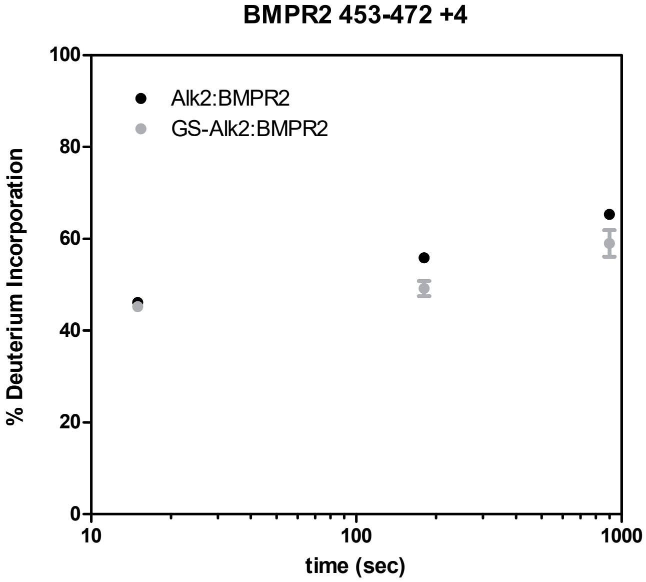

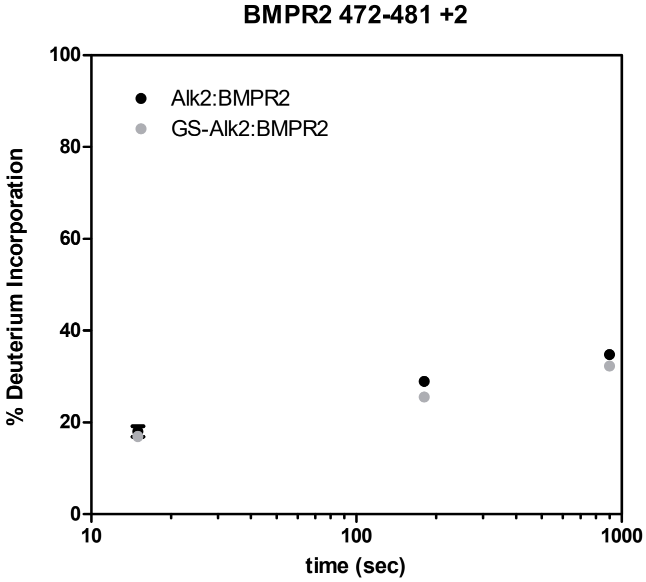

Alk2:BMPR2 complex vs. GS-Alk2-BMPR2 complex - HDX-MS Uptake Plots

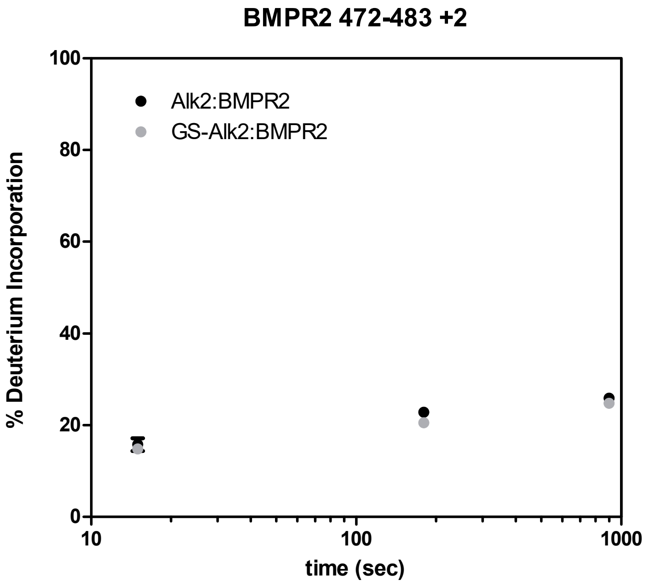

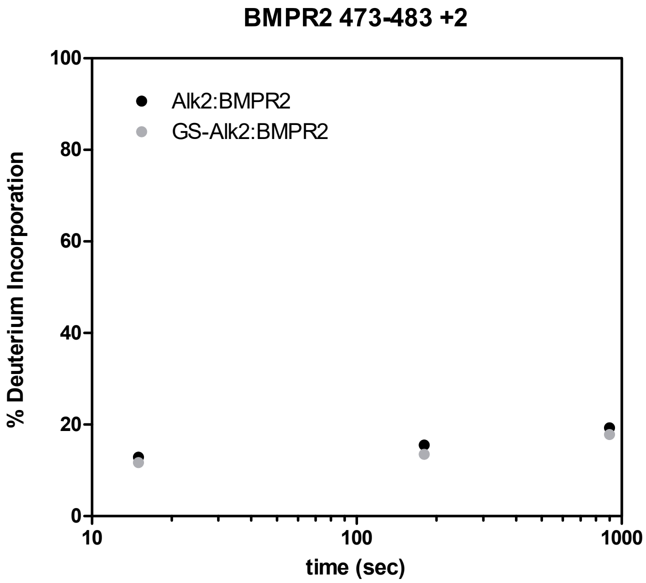

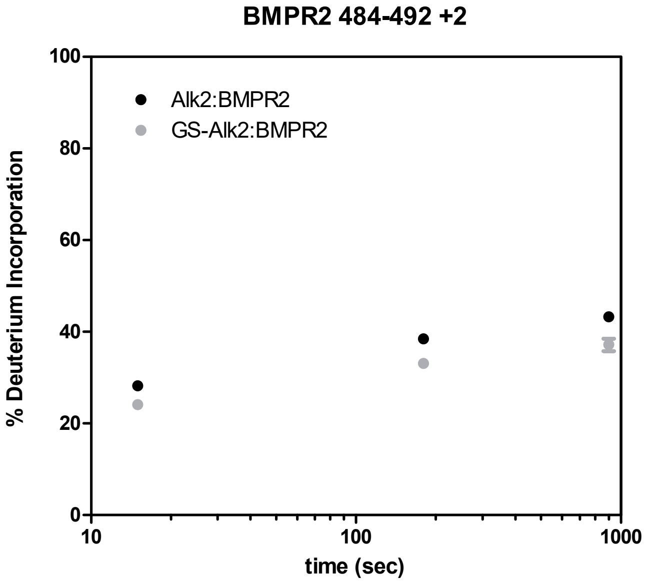

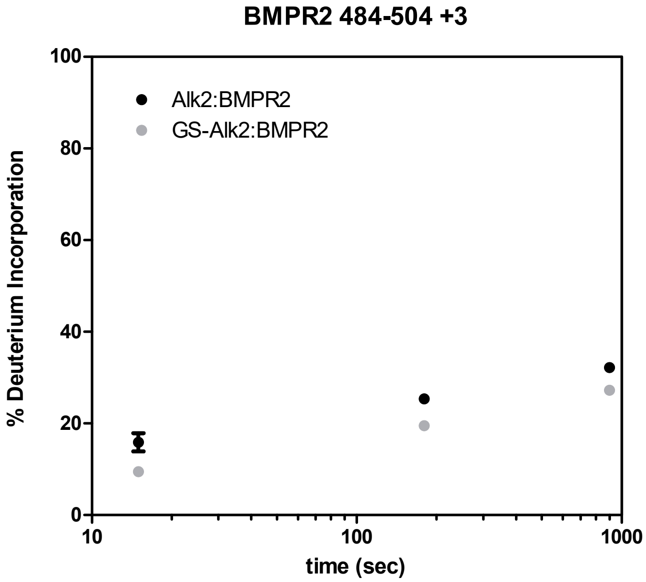

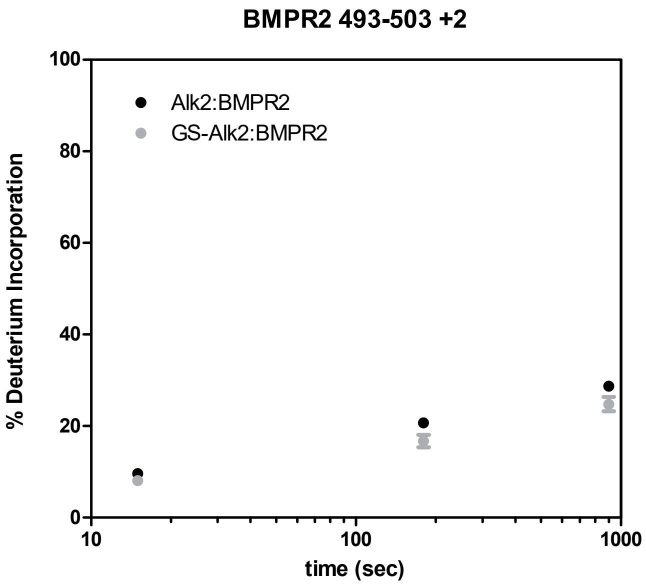

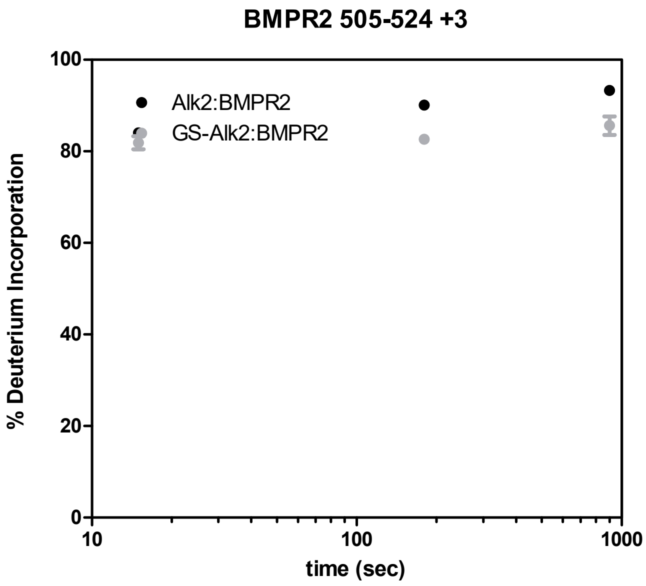

Alk2:BMPR2 complex vs. GS-Alk2-BMPR2 complex - HDX-MS Uptake Plots

BMPR2 505-529 +3

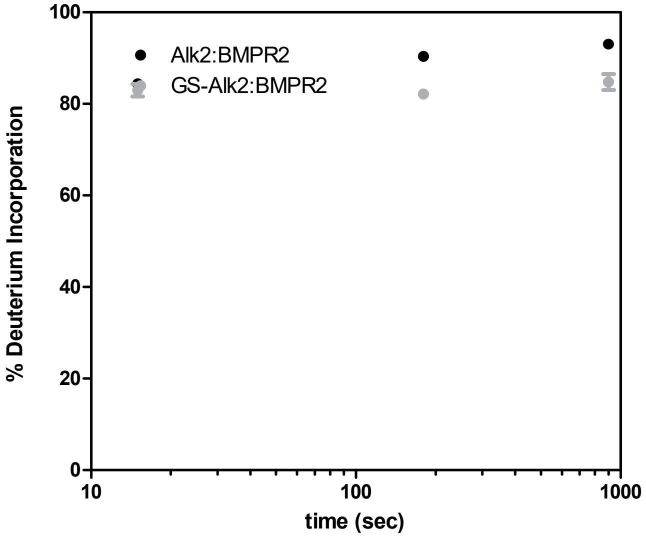

BMPR2 506-523 +2

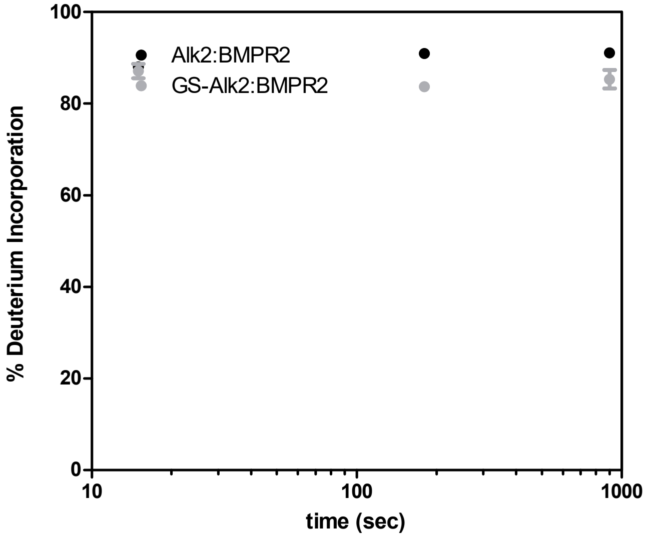

BMPR2 506-524 +3

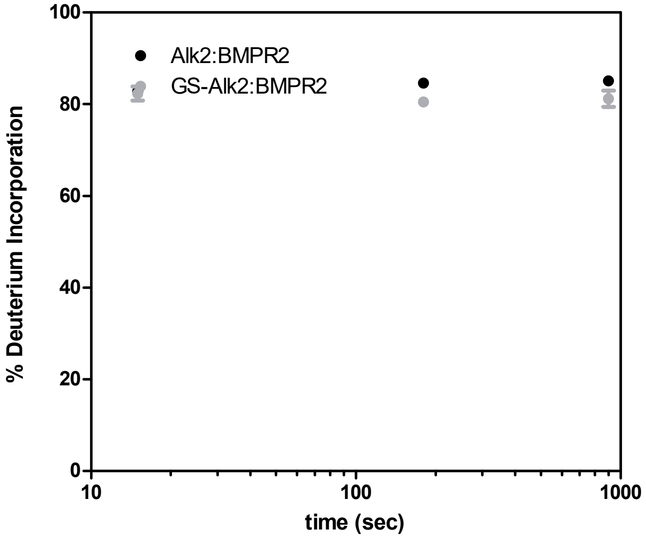

BMPR2 506-529 +3

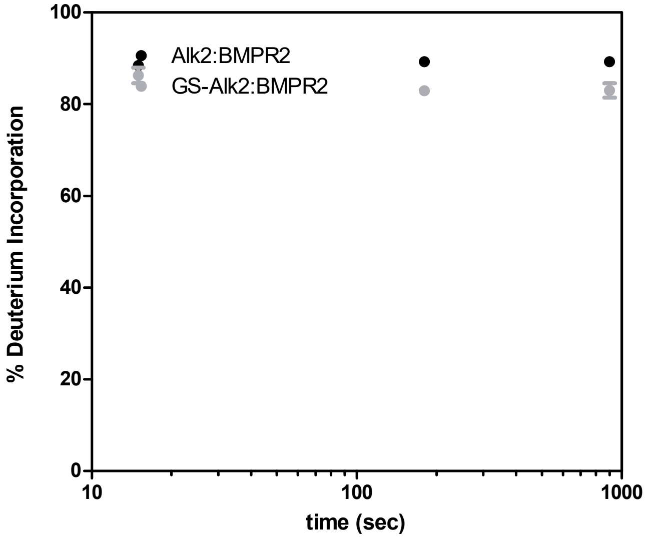

BMPR2 507-523 +3

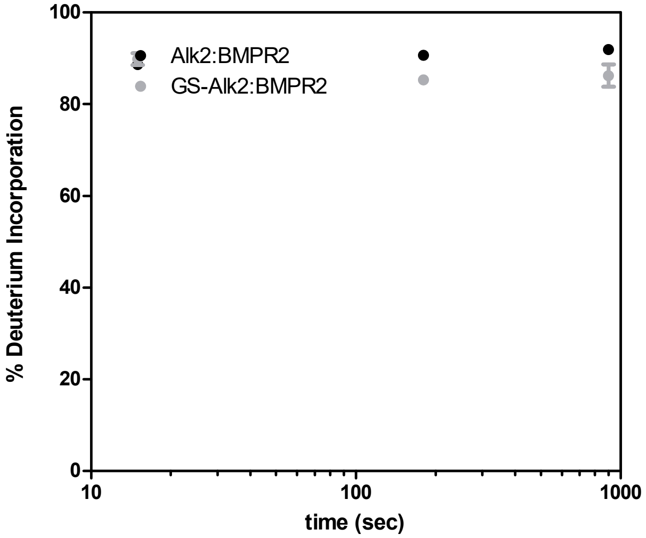

BMPR2 507-524 +3

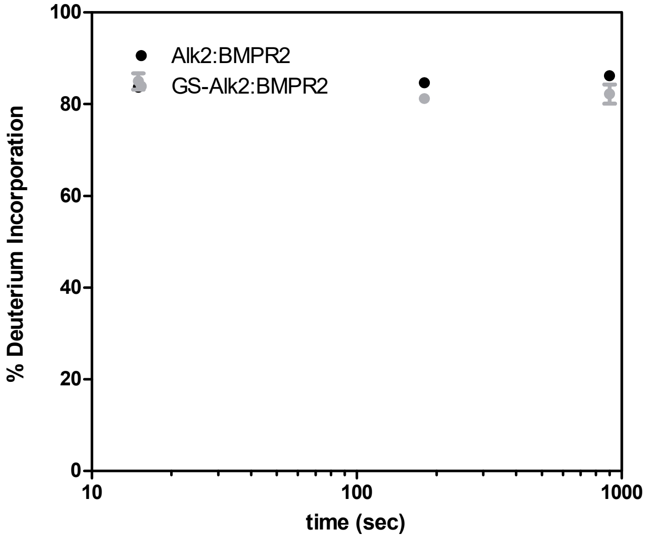

Alk2:BMPR2 complex vs. GS-Alk2-BMPR2 complex - HDX-MS Uptake Plots

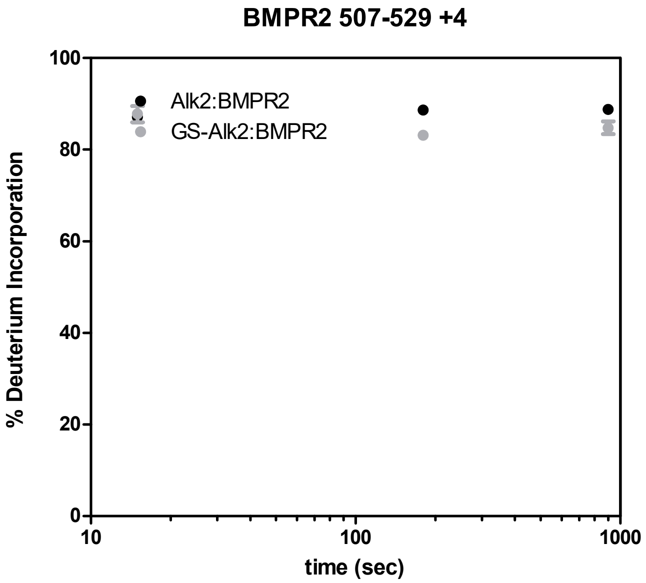

Supplement: Supplementary file 6 — Supplementary Data 3 [file 41467_2021_25248_MOESM6_ESM.zip › Suppl_Data_Set3_Agnew_Ayaz_rev.pdf]
