# Supplementary material for: Structural basis for ALK2/BMPR2 receptor complex signaling through kinase domain oligomerization
Source: Nat Commun. 2021 Aug 16;12:4950. doi: 10.1038/s41467-021-25248-5 (PMC8368100; doi:10.1038/s41467-021-25248-5)
Supplement: Supplementary file 7 — Reporting Summary [file 41467_2021_25248_MOESM7_ESM.pdf]

Corresponding author(s): Natalia Jura

Last updated by author(s): Jul 25, 2021

Reporting Summary

Nature Research wishes to improve the reproducibility of the work that we publish. This form provides structure for consistency and transparency in reporting. For further information on Nature Research policies, see our Editorial Policies and the Editorial Policy Checklist.

Statistics

For all statistical analyses, confirm that the following items are present in the figure legend, table legend, main text, or Methods section.

n/a	Confirmed	
☐	☒	The exact sample size (*n*) for each experimental group/condition, given as a discrete number and unit of measurement
☐	☒	A statement on whether measurements were taken from distinct samples or whether the same sample was measured repeatedly
☐	☒	The statistical test(s) used AND whether they are one- or two-sided<br>*Only common tests should be described solely by name; describe more complex techniques in the Methods section.*
☒	☐	A description of all covariates tested
☒	☐	A description of any assumptions or corrections, such as tests of normality and adjustment for multiple comparisons
☐	☒	A full description of the statistical parameters including central tendency (e.g. means) or other basic estimates (e.g. regression coefficient) AND variation (e.g. standard deviation) or associated estimates of uncertainty (e.g. confidence intervals)
☐	☒	For null hypothesis testing, the test statistic (e.g. F, t, r) with confidence intervals, effect sizes, degrees of freedom and P value noted<br>*Give P values as exact values whenever suitable.*
☒	☐	For Bayesian analysis, information on the choice of priors and Markov chain Monte Carlo settings
☒	☐	For hierarchical and complex designs, identification of the appropriate level for tests and full reporting of outcomes
☒	☐	Estimates of effect sizes (e.g. Cohen's *d*, Pearson's *r*), indicating how they were calculated

Our web collection on statistics for biologists contains articles on many of the points above.

Software and code

Policy information about availability of computer code

Data collection	Crystallographic data was collected at A.L.S. Beamline 8.3.1. The data was integrated in XDS (Kabsch et al, 2010), scaled and merged in AIMLESS (Evans et al, 2013), which is part of the CCP4 suite (CCP4 version 7.0) (Winn et al, 2011). The molecular dynamics (MD) simulations were performed using the Anton 2 Supercomputer. (The simulation code we used is specialized to Anton 2, but many codes performing MD simulation are widely available.) HDX-MS data were collected using Xcalibur ver. 3 (Thermo).
Data analysis	Crystallographic data was analyzed by molecular replacement performed in PHASER ver. 2-7.17 (McCoy et al, 2007) using the BMPR2 kinase domain (RCSB ID: 3G2F) or the ALK2 kinase domain (RCSB ID: 3MTF). The structures were refined by iterative rounds of refinement in Phenix.refine ver. 1.13-2998 (Liebschner et al, 2019) and manual model building with COOT ver. 08-8-8 (Emsley et al, 2010). HDX-MS data were analyzed using HDX Workbench ver. 2.9.8 (Pascal et al, 2012)

For manuscripts utilizing custom algorithms or software that are central to the research but not yet described in published literature, software must be made available to editors and reviewers. We strongly encourage code deposition in a community repository (e.g. GitHub). See the Nature Research guidelines for submitting code & software for further information.

Data

Policy information about availability of data

All manuscripts must include a data availability statement. This statement should provide the following information, where applicable:

- Accession codes, unique identifiers, or web links for publicly available datasets
- A list of figures that have associated raw data
- A description of any restrictions on data availability

Atomic coordinates have been deposited in the Protein Data Bank (PDB) under accession codes 6UNP (BMPR2KD-D485G), 6UNQ (ALK2KD-K493A with AMPPNP), 6UNR (ALK2KD-K492A/K493A with AMPPNP) and 6UNS (ALK2KD-K492A/K493A with LDN-193189). The molecular dynamics trajectories for data described in Figs. 2

and 7a,b,c and Supplementary Figs. 2, 10 and 12 are available for non-commercial use through contacting trajectories@deshawresearch.com. Atomic coordinates for the reported models are provided in Supplementary Datasets 1 and 2. All HDX-MS uptake plots for data presented in Fig. 3 and Supplementary Figs. 4, 6, 7 and 8 are included in the Supplementary Dataset 3. HDX-MS data files and source data for Supplementary Figures 4, 6, 7, and 8 are available in the ProteomeXchange Consortium via the PRIDE96 partner repository with the dataset identifier PXD022944.

Field-specific reporting

Please select the one below that is the best fit for your research. If you are not sure, read the appropriate sections before making your selection.

- [x] Life sciences
- [ ] Behavioural & social sciences
- [ ] Ecological, evolutionary & environmental sciences

For a reference copy of the document with all sections, see nature.com/documents/nr-reporting-summary-flat.pdf

Life sciences study design

All studies must disclose on these points even when the disclosure is negative.

Sample size	No sample size calculation was performed. The sample size was chosen based on the author's prior experiences with the experiments, as detailed in the following publications (Lagna et al, 2007, PMID: 17947237; Littlefield et al, 2014, PMID: 25468994; Loving and Underbakke, 2019 PMID:31403288)
Data exclusions	No data were excluded to generate graphs.
Replication	All attempts at replication were successful. Each experiment was repeated at least three times for the reproducibility. Each condition was quadruplicated with 4 independent biological samples for the luciferase assays, and 3 independent runs of samples in triplicates for the kinase assays. The enzymatic assays were performed on distinct samples from separate purifications. For the HDX-MS results, the sample size for each treatment and time point was n = 3 technical replicates. For purification of the BMPR2 and ALK2 kinase domain constructs, at least ten times of independent purification were carried out.
Randomization	This is not relevant for all the types of assays and analyses reported in this study because the measurements do not depend on human observations but are collected by the automated devices. Specifically, all assays and analyses generate numerical outputs that are then evaluated using software-based analysis standardized in the field and used by entire scientific community (such as, data collection and analysis software for X-ray crystallography, HDX-MS, SAXS and MD). The luciferase and kinase assay generate numerical values that are non-arbitrary, and they are later subject to comparison by statistical analyses. No animals or human research participants are involved in this study.
Blinding	We blinded samples during the measurement of the luciferase activity by applying random numbers to the samples. For HDX-MS analysis, the exchange experiments were done by one investigator (E. Underbakke) and MS analysis blindly by another (H. Loving). Blinding was not relevant to other aspects of this study, as the protein is not required to be allocated into experimental groups in protein structural studies, and no animals or human research participants are involved in this study.

Reporting for specific materials, systems and methods

We require information from authors about some types of materials, experimental systems and methods used in many studies. Here, indicate whether each material, system or method listed is relevant to your study. If you are not sure if a list item applies to your research, read the appropriate section before selecting a response.

Materials & experimental systems

n/a	Involved in the study	
[ ]	[x]	Antibodies
[ ]	[x]	Eukaryotic cell lines
[x]	[ ]	Palaeontology and archaeology
[x]	[ ]	Animals and other organisms
[x]	[ ]	Human research participants
[x]	[ ]	Clinical data
[x]	[ ]	Dual use research of concern

Methods

n/a	Involved in the study	
[x]	[ ]	ChIP-seq
[x]	[ ]	Flow cytometry
[x]	[ ]	MRI-based neuroimaging

Antibodies

Antibodies used	The monoclonal anti-BMPR2 carboxyl terminal domain antibody was purchased from BD Biosciences (BD Bioscience, Cat# 612292). anti-GAPDH antibody clone 6C5 (Millipore, Cat# MAB374), horseradish peroxidase (HRP)-conjugated anti-HA antibody (Roche clone 3F10, supplied by SIGMA-Aldrich, Cat# 12013819001). All antibodies were used at 1:1000 dilution.
Validation	All these antibodies are commercially available, generally used and have been extensively validated by the manufacturers. The validation data available online for these antibodies includes detection of specific epitopes by Western blot using a range of antibody dilutions. More detailed information is available per request from each vendor.

Eukaryotic cell lines

Policy information about cell lines

Cell line source(s)	In this study we use HEK-BRR-luc cells, which are clonal derivative of HEK293 in which pFAm.BRE.Luc.Neo construct was stably integrated. There is no commercial source of these cells. They can be obtained per request from the Economides laboratory (Regeneron) or the Hata laboratory (UCSF). Sf9 insect cells were obtained from a commercial vendor, Expression Systems (Davis, CA).
Authentication	The HEK-BRR-luc cell line has been characterized and authenticated as described in Hatsell and colleagues, Science Translational Medicine, 2015. The original cells were obtained from ATCC, expanded in the lab and transfected with the luciferase construct. PCR was used to confirm presence of the GC-rich BMP-responsive element (BRE; 5'-GCCGCCcgCAGC-3'). The inducible expression of Luciferase was confirmed with Bright-Glo Luciferase Assay System (Promega) upon BMP6 induction of cells.
Mycoplasma contamination	Mycoplasma contamination was tested monthly and cells used in the manuscript were tested negative.
Commonly misidentified lines (See ICLAC register)	No such cell lines were used in this study.
